# Ball‐Milling‐Enabled Reactivity of Manganese Metal**

**DOI:** 10.1002/anie.202108752

**Published:** 2021-09-16

**Authors:** William I. Nicholson, Joseph L. Howard, Giuseppina Magri, Alex C. Seastram, Adam Khan, Robert R. A. Bolt, Louis C. Morrill, Emma Richards, Duncan L. Browne

**Affiliations:** ^1^ School of Chemistry Cardiff University Main Building, Park Place Cardiff CF10 3AT UK; ^2^ Department of Pharmaceutical and Biological Chemistry University College London (UCL) School of Pharmacy 29–39 Brunswick Square London WC1N 1AX UK

**Keywords:** ball milling, electron paramagnetic spectroscopy, manganese, organometallics, reductive coupling

## Abstract

Efforts to generate organomanganese reagents under ball‐milling conditions have led to the serendipitous discovery that manganese metal can mediate the reductive dimerization of arylidene malonates. The newly uncovered process has been optimized and its mechanism explored using CV measurements, radical trapping experiments, EPR spectroscopy, and solution control reactions. This unique reactivity can also be translated to solution whereupon pre‐milling of the manganese is required.

## Graphical abstract

A manganese‐mediated reductive dimerization of arylidene malonates by ball milling is reported. The process has been optimised and its mechanism explored by CV measurements, radical trapping, and EPR spectroscopy. Control experiments identify the action of ball milling rather than mortar and pestle is necessary to realise the effective activation of manganese, which can then be used in either solution or a ball mill for subsequent reactions.

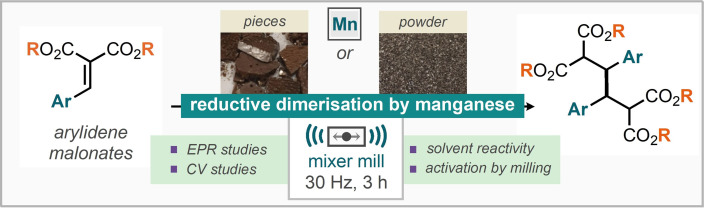

In recent times mechanochemistry by ball milling has been widely explored for a range of synthetic transformations including metal or organo‐catalyzed processes;^[1]^ heterocycle synthesis;^[2]^ construction of metal organic materials;^[3]^ and the generation and use of organometallic reagents.^[4]^ Often, the implementation of these reactions by ball‐milling techniques can lead to alternative selectivity, shorter reaction times, reduced waste and/or the ability to access reaction pathways not accessible in solution.^[5]^ Synthetic mechanochemistry has been the topic of several reviews to describe and collate these potential benefits in an effort to harness the technique and pave the way forward for this intriguing reactor technology.^[6]^

The improved robustness of air sensitive reaction processes is an emerging theme for reactions which can benefit from being conducted in a ball‐mill. Indeed, the mechanochemical ball‐milled versions of palladium catalysed cross‐couplings such as the Buchwald‐Hartwig reaction, thiol aryl/alkyl‐ation and palladocycle formation have all been demonstrated to provide this increased robustness and seemingly reduced sensitivity to air and moisture.^[7]^ C−H activation processes using rhodium have also been demonstrated to benefit in a similar manner.^[8]^ With regards to the generation of organometallic reagents, our group, as well as others, have explored the generation and use of organozinc species, whereby the reaction outcome is independent of the initial form of the zinc metal. Milling the zinc metal in its variety of forms in the presence of an alkyl/aryl/allyl‐halide has led to the successful generation and application of organozinc species in the ball‐milled Negishi, Reformatsky and Barbier‐type reactions (A, Scheme 1).[^4a^, ^4b^, ^4c^] Notably, these reactions require no additive for the activation of zinc (mechanical action is assumed to be responsible), no reaction solvent is required and no inert gas atmosphere is necessary. The materials are simply weighed into the milling jar on the bench (if standard chemical hazards permit), the jars are closed and milling is commenced. In a similar vein, the generation of organomanganese reagents from manganese metal is also reported as capricious, with transmetallation from an alternative organo‐metal to Mn^II^ salts being the preferred strategy. Nonetheless, the chemistry and reactivity of organomanganese reagents appears to be relatively unique compared to alternative organometallic species and harnessing a reliable method for the preparation of these materials could well pave the way to further developing these species.^[9]^ We therefore set out to translate our zinc metal conditions into those for manganese metal (B, Scheme 1), exploring a variety of electrophiles. To our surprise, when milling manganese pieces and ethyl 4‐bromobutyrate, **1**, as a pro‐nucleophile with diethyl 2‐benzylidenemalonate (**2**) as electrophile (B, Scheme 1), none of the targeted conjugate addition product **3** was observed. Rather the reductive coupling of the arylidene malonate **2**, leading to dimer **4** was instead isolated in 66 % yield. Putting this finding into context we have found very few examples of this particularly reductive dimerization process. Yamashita and co‐workers report that such a reaction can be affected using samarium (II) iodide leading to reductively dimerized material in good yield and short timescales (1 hour, C, Scheme 1).^[10]^

**Scheme 1:**
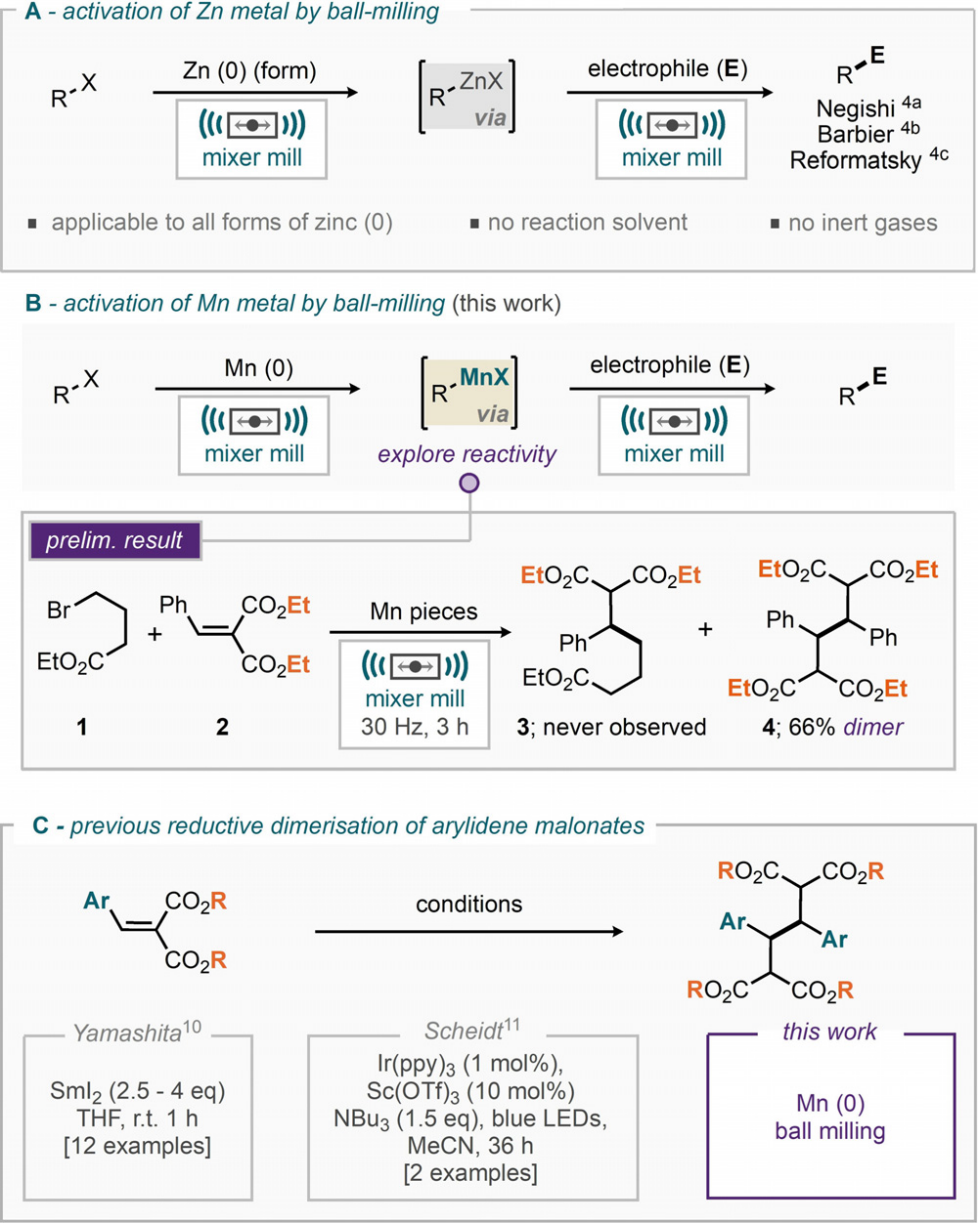
Examples of using mechanochemistry to alter reactivity by different methods.

McDonald and Scheidt reported a Lewis acid/photoredox cooperative catalysis approach to the reductive coupling of arylidene malonates with two examples of the dimerization process when oxidative quenching of the photocatalyst is employed.^[11]^ Previous work from Cahiez and co‐workers has also shown the β‐reductive dimerization of cyclohexenone by dibutyl manganese, although this dimerization process was only observed for cyclohexenone substrates and not arylidene malonates.^[12]^ Looking to develop an understanding of the ball milled manganese mediated process we opted to firstly optimize the reaction. Initial optimization was focused on the composition of reagents required for the new dimerization transformation. This found that Mn, LiCl, and a liquid assisted grinding (LAG) agent must be present for any reactivity (Table 1, entries 1–3). Previous reactions conducted under ball‐milling conditions have shown that altering the liquid additive can alter the product selectivity or improve on reaction yields, therefore, alternative liquid additives were screened.^[13]^ Our results indicate that more highly coordinating liquids induce the greatest reactivity, (Table 1, entries 4 and 7), which may be attributed to the greater stabilization of organometallic intermediates by these materials. As the ethyl 4‐bromobutyrate had not led to the anticipated reactivity, we also explored its necessity in the reaction. Upon omission of this substrate from the reaction, the yield dropped significantly; down to 10 % (Table 1, entry 10). Clearly the low yield initially suggests that 4‐bromobutyrate (**1**) is required for reactivity. It is already well observed in the literature that the composition of liquid and solid form reagents within the ball‐milling reactor can significantly alter the product distribution.^[14]^ Therefore, to correct for the smaller quantity of liquid with **1** omitted, an extra equivalent of THF was used, which led to an improved yield of 80 % (Table 1, entry 11), indicating that improved yield of product is obtained with increased liquid content within the mill. Further attempts to improve the reaction beyond this consisted of varying the amount of manganese metal in the reaction (0.55 equiv. and 2.0 equiv. Table 1 entries 12 and 13 respectively), and the addition of a range of grinding auxiliaries which can be used to moderate the texture and heat capacity of the reacting mixture.^[15]^

**Table 1:**
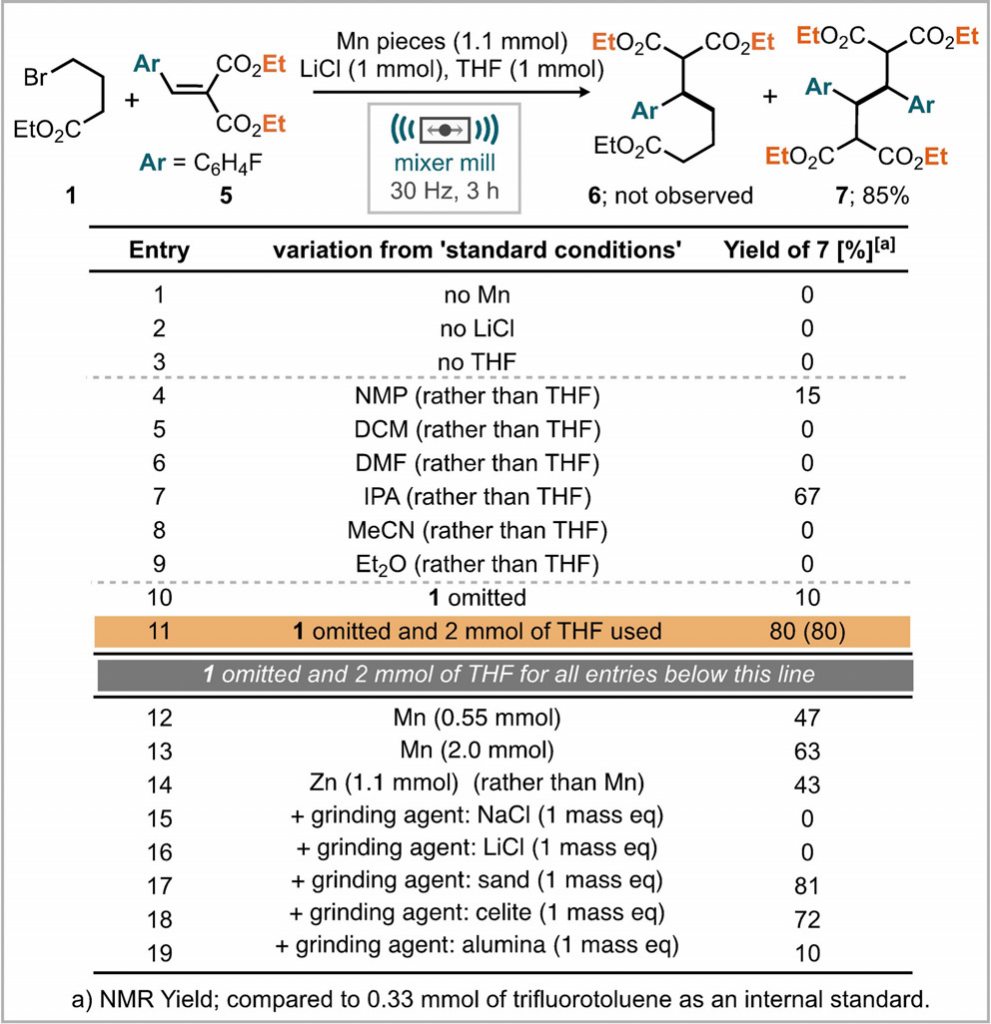
Optimisation of the manganese mediated reductive dimerization of arylidene malonates by ball‐milling.

Neither of these resulted in improvement, although the addition of sand led to a similar reaction yield (Table 1, entries 15–19). Notably, the reaction was also demonstrated possible with zinc metal as the reductant affording the reductively coupled dimer in 43 % yield (Table 1, entry 14). Having determined the optimum reaction conditions (Table 1, entry 11), we subsequently turned our attention to explore the substrate scope. Initially we investigated the propensity for other unsaturated aryl bearing electron‐deficient systems to participate in this process which was met with no success for ethyl cinnamate, cinnamonitrile, 2‐nitrovinyl benzene, 2‐benzylidene malonitrile and ethyl 3‐phenylpropiolate where starting material was returned (C, Scheme 2). Despite this, the reaction is applicable to a small range of arylidene malonates with para functionality, leading to products including trifluoromethyl (**8**), methyl (**9**), methoxy (**10**), methyl ester (**11**), nitrile (**12**) and chloro (**13**) functionality (A, Scheme 2). Notably, bromo substitution was not tolerated, potentially due to manganese insertion into the C‐Br bond as evidenced by an inseparable mixture of reaction products. Variation of the electron‐withdrawing group was also explored where replacement of ethyl esters for both methyl and *iso*‐propyl groups still permitted the reaction to proceed in moderate yield (**15** and **168**, B, Scheme 2). Allyl substituted esters furnished the reductively coupled product (**17**) in 77 % isolated yield and demonstrates a tolerance of the reaction mechanism to unactivated double bonds. The mixed nitrile and ester system, ethyl‐2‐cyano‐3‐phenylacrylate also underwent dimer formation to give the desired product (**14**) in 55 % yield. Notably, the nitrile malonate substrate led to a single diastereomer of product, in contrast to every other substrate which gave both syn and anti‐products. Substrate scope studies demonstrated that rather specific electronic activation is required to permit reductive coupling by manganese, including two strong electron withdrawing groups and benzylic stabilization. Further, substrates which could permit trapping of a radical by ring formation (such as allyl ester and citronellal derived malonates), or ring fragmentation (cyclopropyl) did not furnish any of the corresponding reductively coupled products. With this information in hand, together with prior methods for the reductive coupling of arylidene malonates we proposed and investigated plausible reaction mechanisms (Scheme 3). Notably, support from computational methods is absent, it appears that many computational methods for supporting mechanistic hypotheses rely on intrinsic and implicit solvent molecules. Our proposed mechanism starts with the Lewis acid activation of the malonate moiety with lithium ions (Scheme 3 a). We note that LiCl was essential for the observed reactivity (Table 1, entry 2). Furthermore, it was demonstrated by Scheidt and co‐workers that the addition of a Lewis acid to similar substrates lowers the reduction potential, leading to more facile reduction.^[11]^ To explore this further the reduction potentials of 4‐trifluoromethyl and 4‐methoxy substituents were measured in the presence and absence of LiCl against ferrocene (*E*_1/2_=0.382 V vs. SCE in MeCN, Scheme 3 b). In general, addition of LiCl results in the decrease of the reduction potential of the arylidene malonates explored. However, it is worth noting that the electrochemical measurements were performed in solution phase and it is unclear at this point how readily they translate to non‐solution conditions.

**Scheme 2:**
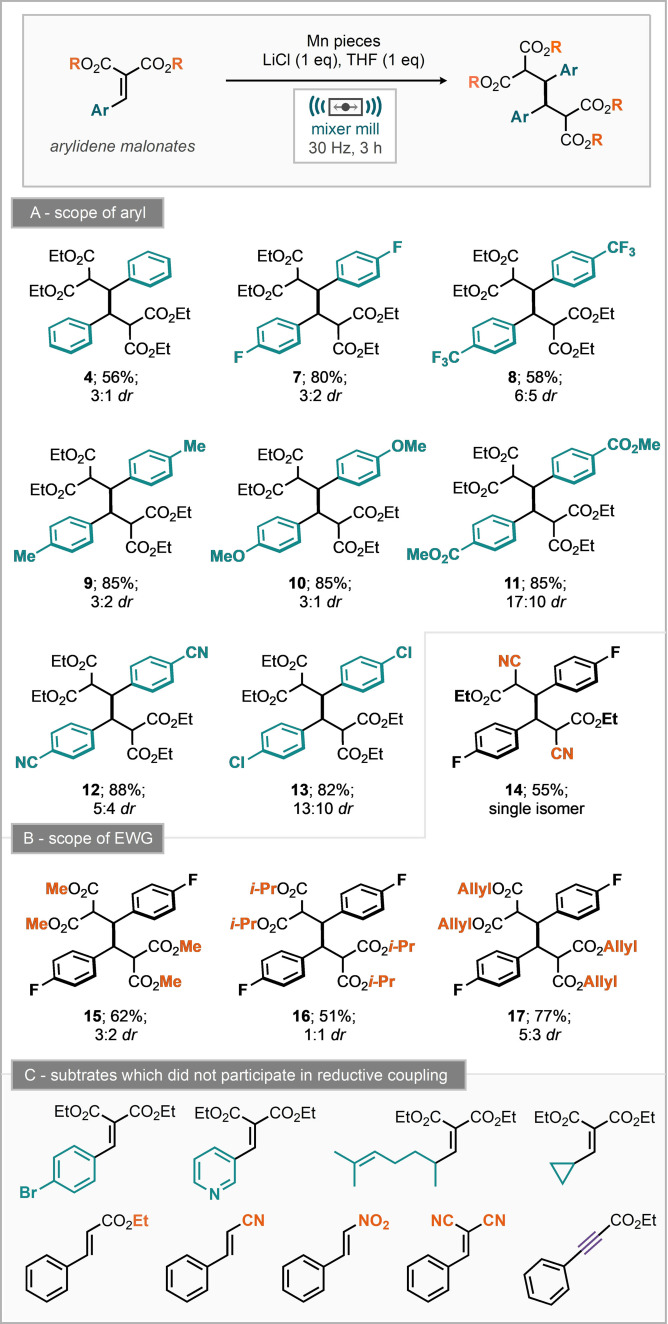
Substrate scope of the manganese mediated reductive dimerization of arylidene malonates by ball‐milling.

**Scheme 3:**
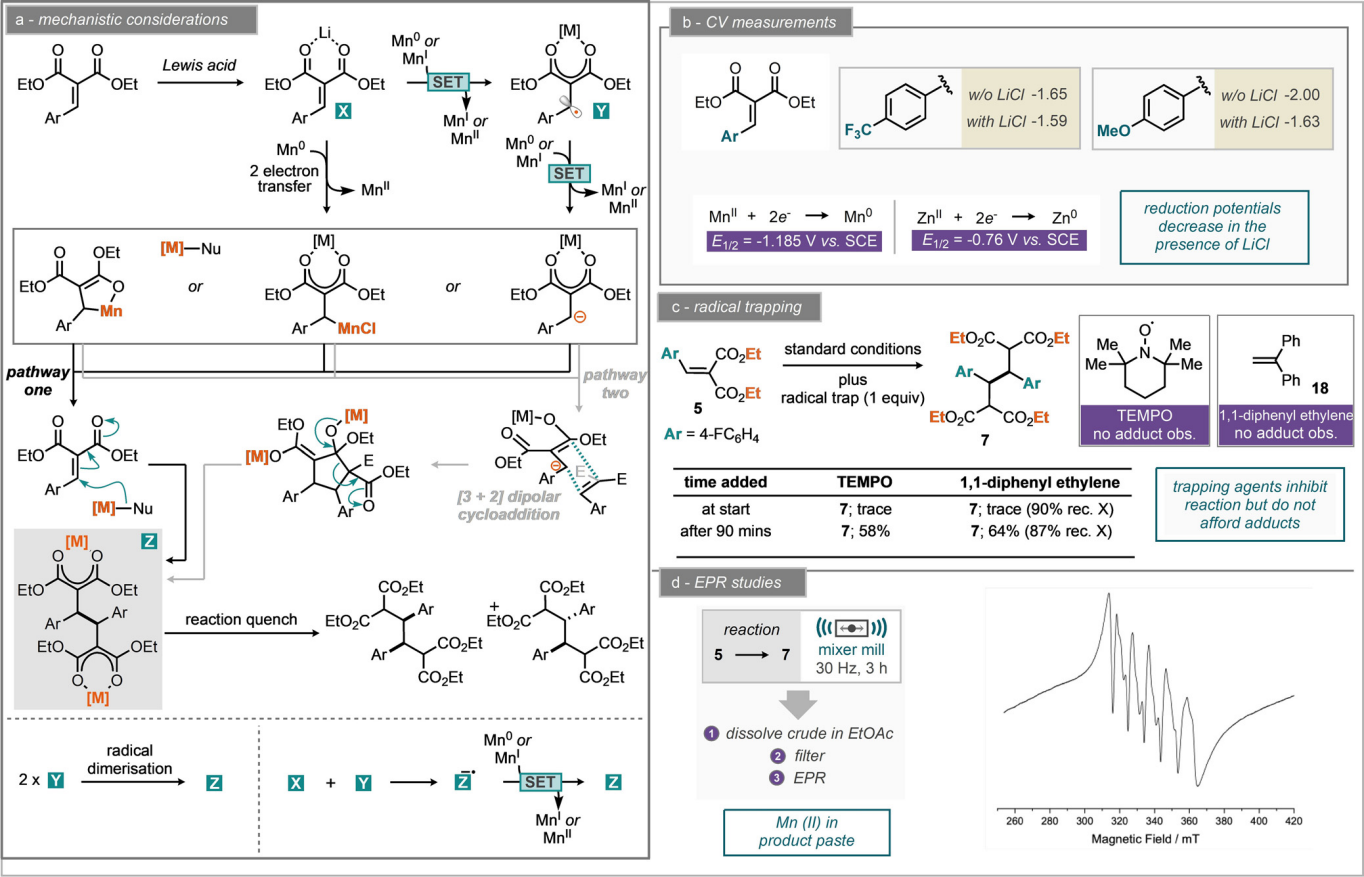
Mechanistic investigation of the manganese mediated reductive dimerization of arylidene malonates by ball‐milling.

Suprisingly, the measured reduction potentials are greater than the reducing power of Mn metal (*E*_1/2_=−1.185 V vs. SCE) suggesting that it is not possible for Mn to reduce these substrates without additional factors at play.^[16]^ Perhaps even more perplexing is that zinc metal is also seemingly a competent reductant in this ball milled reaction (Table 1, entry 14) yet the reducing power of Zn metal is *E*_1/2_=−0.76 V vs. SCE.^[16]^

From these experimental observations, we propose several paths forward towards the product from an initial Li ion chelated arylidene malonate. From this activated adduct the reduction by Mn could take place either i) in the form of a two‐electron reduction to generate the organometallic intermediate (which may exist in several forms), or ii) via a series of single electron transfer (SET) reduction events to generate radical intermediates that could themselves then act as the nucleophile before further reduction. As a test for the presence of radical intermediates (i.e. pathway (ii)), experiments were performed in the presence of the radical inhibiters TEMPO and 1,1‐diphenyl ethylene (Scheme 3 c). Reactions were run in two permutations. One with the radical trapping agent in place from the beginning of the reaction and the other with a pausing of milling after 90 mins and addition of the trap. When either radical trap was present from the start of the reaction, this caused an almost complete shutdown of reactivity with only trace product being detected by NMR and none of the corresponding adducts observed. In the case of 1,1‐diphenyl ethylene, 90 % of the trap was recovered. When the radical trap was added to the reaction after 90 mins, the reactions yielded reductively coupled product in 58 % for TEMPO and 64 % for 1,1‐diphenyl ethylene (cf ≈80 % yield in the absence of the radical trap). In none of the experiments were adducts of the trap with the substrate observed. The impact of these materials on the reaction may be due to changes in the mixing and rheological properties of the reaction mixtures. Clearly, these results indicate that there is some formation of the product prior to addition of the trap, but further reactivity is quenched immediately upon radical trap addition.

Interrogation of the reaction by electron paramagnetic resonance (EPR) spectroscopy, demonstrates a characteristic signal for Mn^II^ species. The EPR spectrum of an ethyl acetate solution of the crude paste obtained directly from the milling jar pre‐workup contained a sextet multiplet feature, with six equal‐intensity hyperfine lines separated by 260 MHz (9.3 mT), which is characteristic of a d^5^ Mn^II^ species with *I*(^55^Mn)=5/2 (Scheme 3 d). Observation of the two‐electron oxidized metal at the end of the reaction does not provide confirmation of a single two‐electron reduction pathway (i.e. pathway (i)), nor sequential single‐electron reduction steps of the substrate (i.e. pathway (ii)), but does confirm that manganese metal has been oxidised during the reaction process. Further EPR measurements were performed on the reaction product following work‐up to identify the origin of the Mn^II^ signal (detailed in the ESI). The possibility of a concerted [3+2] dipolar cycloaddition followed by ring opening is also proposed and electrophile trapping experiments have not resulted in any isolatable intermediates along this pathway. Finally, we were drawn to further explore the comminution of manganese by the action of milling to enable this newly observed reactivity (Scheme 4). This was explored with a number of control reactions. Firstly, treating the model substrate **5** to solution reaction stirred in THF at room temperature for 24 hours resulted in complete recovery of starting material when manganese pieces were used and trace product when employing manganese powder (Scheme 4). Performing the same reactions at reflux returned trace product with manganese pieces and 11 % yield of the reductively coupled product with manganese powder. Somewhat surprisingly, when manganese pieces and powder are each milled for 5 minutes at 30 Hz and then exposed to the arylidene malonate in THF solution at room temperature for 24 hours, excellent yields of the reductively coupled product (**7**) are returned in 86 % and 78 % respectively. This yield is comparable, if not marginally better than when the reaction is run purely by milling for three hours. As a final investigation, mortar and pestle grinding (or pre‐activation) for two minutes was compared to milling for two minutes at 30 Hz for both manganese pieces and powder. In the former case, pieces returned 3 % of the reductively coupled product following 24 hour reaction in THF at room temperature and powder returned 2 % yield. In the ball‐milled case the pre‐activated pieces afforded 69 % reductively coupled product and the powder afforded 65 % yield.^[17]^ Together these results show that the fine comminution of manganese metal by high energy ball milling is necessary to enable this mode of reactivity for manganese.

**Scheme 4:**
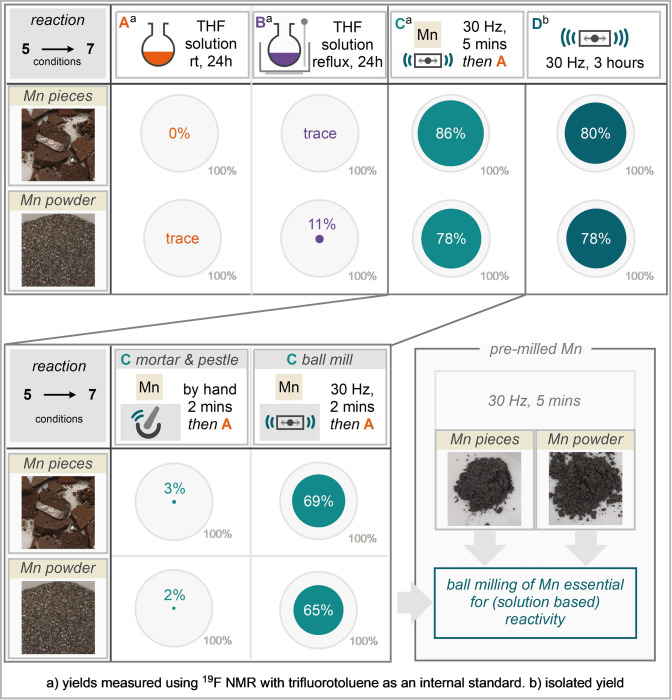
Control experiments of solution versus milling for manganese activation.

In conclusion, a novel dimerization of electron poor alkenes by manganese has been discovered. This new reaction has been optimized and explored to develop a deeper understanding. EPR measurements confirm the presence of Mn^II^ at the end of the reaction; measured reduction potentials point towards activation by addition of lithium chloride and radical trapping experiments rule out the intermediacy of interceptable radical species. The exact mechanism remains unclear. However, control experiments have confirmed that ball milling of manganese is essential to unlock this mode of reactivity, this finding delivers a particularly exciting prospect for the future of earth‐abundant manganese chemistry.

## Conflict of interest

The authors declare no conflict of interest.

## Supporting information

As a service to our authors and readers, this journal provides supporting information supplied by the authors. Such materials are peer reviewed and may be re‐organized for online delivery, but are not copy‐edited or typeset. Technical support issues arising from supporting information (other than missing files) should be addressed to the authors.

Supporting Information
