# Supplementary material for: Ball‐Milling‐Enabled Reactivity of Manganese Metal
Source: Angew Chem Int Ed Engl. 2021 Sep 16;60(43):23128–33. doi: 10.1002/anie.202108752 (PMC8596600; doi:10.1002/anie.202108752)

Angewandte Chemie
Eine Zeitschrift der Gesellschaft Deutscher Chemiker

Supporting Information

Ball-Milling-Enabled Reactivity of Manganese Metal**

*William I. Nicholson, Joseph L. Howard, Giuseppina Magri, Alex C. Seastram, Adam Khan, Robert R. A. Bolt, Louis C. Morrill, Emma Richards, and Duncan L. Browne**

anie_202108752_sm_miscellaneous_information.pdf

Supporting Information

Table of Contents

General Information.. 2

Initial investigation.. 4

Optimization of Reductive Dimerisation.. 6

Cyclic Voltammetry Study.. 9

EPR Experiments.. 15

Control Reactions.. 18

Unproductive Substrates.. 19

Characterization Data.. 20

Starting material Synthesis.. 20

Product Characterisation.. 33

NMR Spectra.. 42

HPLC Traces.. 122

General Information

All aldehydes were passed through a basic alumina plug directly before use, all other reagents purchased from commercial sources were used without further purification. Thin layer chromatography (TLC) was carried out using Merck TLC silica gel 60 sheet and visualized with ultraviolet light or potassium permanganate stain. Preparatory TLC was carried out using Analtech TLC uniplates: silica gel matrix (200 x 200 x 1.5 mm) and visualized with ultraviolet light.

Flash column chromatography was performed with Sigma Aldrich silica gel 40-60 Å as the stationary phase and solvents employed were analytical grade.

^{1}H, ^{13}C, and ^{19}F NMR spectra were recorded on a Bruker 400 Ultrashield or Bruker AVX500 (500 MHz) spectrometer at ambient temperature. The obtained chemical shifts; δ, are reported in ppm and are referenced to the residual solvent signal. Spin-spin coupling constants; J, are given in Hz. NMR yields were measured using 0.33 mmol of trifluorotoluene as an internal standard.

Melting points were measured on a Gallenkamp melting point apparatus and are reported corrected by linear calibration to benzophenone (47 - 49 °C) and benzoic acid (121 - 123 °C).

High resolution mass spectral (HRMS) data were obtained on a Waters MALDI-TOF mx in Cardiff University. Spectra were obtained using electron impact ionization (EI), chemical ionization (CI), positive electrospray (ES), pneumatically assisted electrospray (pNSI) or atmospheric solids analysis probe (ASAP+).

Infrared spectra were recorded on a Shimadzu IR-Affinity-1S FTIR spectrometer.

The ball mill used was an InSolido Technologies IST400 using 14 mL hardened stainless steel jars. The grinding was set to 30 Hz and milling time set to 0 and a stopwatch used to monitor reaction time.

The GC yield of products and conversion of substrates were determined using the internal standard method. The response factor (RF) of analytes was determined by analysing known quantities of internal standard (trifluorotoluene) against known quantities of substrate and product:

$$\mathrm{RF} = \frac{\mathrm{Area_{Internal\ standard}\ x\ Moles_{Analyte}}}{\mathrm{Area_{Analyte}\ x\ Moles_{Internal\ standard}}}$$

The quantity of an analyte was then calculated according to the following equation:

$$\mathrm{Moles_{Analyte}} = \frac{\mathrm{RF\ x\ Moles_{Internal\ standard}\ x\ Area_{Analyte}}}{\mathrm{x\ Area_{Internal\ standard}}}$$

The preparatory HPLC was carried out with a Japan Analytical Industry LC-9110 II Next equipped with a normal phase column (JAIGEL-SIL, 043-10), a UV-Vis detector and set to recycling with a 10 mLmin^{-1} flow rate.

Cyclic voltammetry (CV) experiments were conducted using an Autolab PGSTAT204, controlled using Nova 2.1 software. The working electrode was a GC disc (3 mm dia., BASi part number MF-2012), the counter electrode was a Pt-wire (BASi part number MW-4130) and a Ag/$AgNO_3$ reference electrode was used (BASi part number – MW-1085) in a 10 mL glass vial. The solution of interest was purged with N_2 for 10 minutes before data collection. After data collection, ferrocene (5 mM) was added and an additional scan was run. The parent data was referenced relative to the $Fc^{+/0}$ couple that was recorded ($E_{1/2}$ = 0.382 V).

All EPR spectra were recorded on a Bruker EMX utilising an ER4119_SHQE resonator, at 120 K using a Bruker Variable Temperature Unit, operating at 10 mW MW power, 100 kHz field modulation and 5 G modulation depth. Spin-trap samples were recorded at room temperature, utilising the ER4102_shqe resonator, operating at 0.64 mW MW power, 100 kHz field modulation and 0.5 G modulation depth. Simulations of the experimental data were performed using the garlic function in the Easyspin toolbox for Matlab, to determine the g_{iso} and a_{iso} spin Hamiltonian parameters.[1]

[1] S. Stoll, A. Schweiger, *J. Magn. Reason.* **2006**, 178, 42-55

Ball Milling Equipment Used

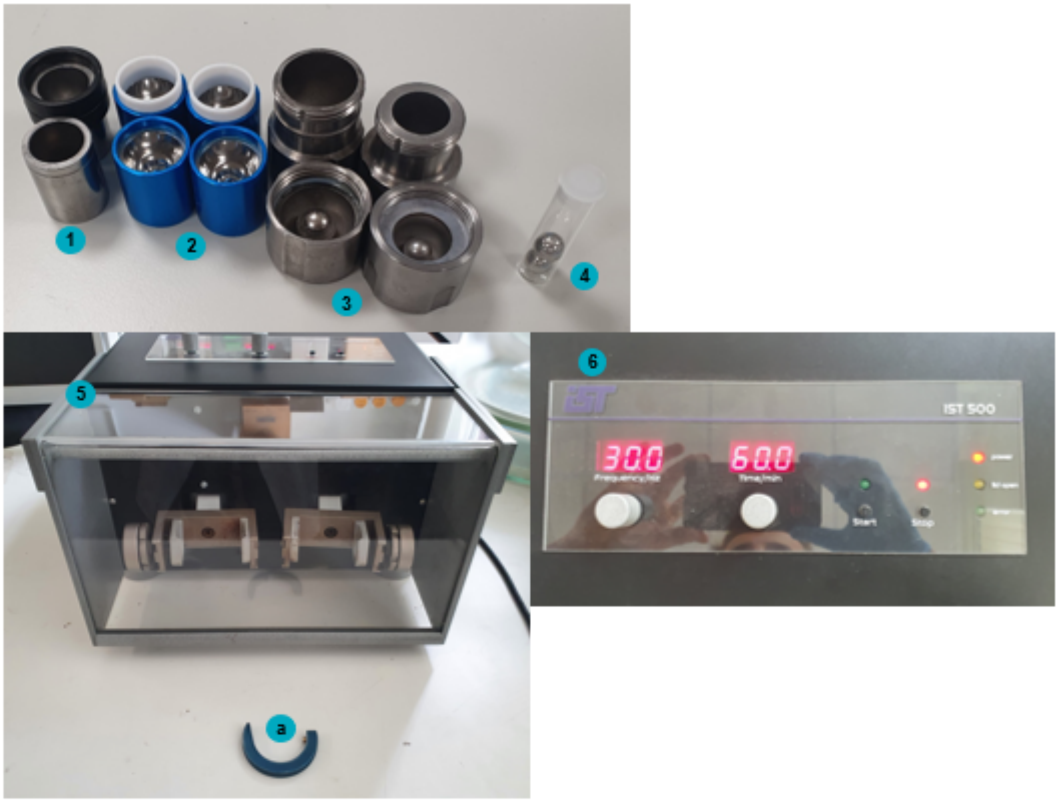

1) IST milling jar 14 mL stainless steel with retaining collar (http://www.insolidotech.org/accessories.html)

2) FTS Smartsnap™ grinding jars, 15 mL stainless steel with PTFE retaining washer (https://formtechscientific.com/fts-1000-shaker-mill/products.html?section=accessories&accessory=smartsnap-grinding-jars)

3) Retsch stainless steel milling jars 25 mL and 15 mL (https://www.retsch.com/products/milling/ball-mills/mixer-mill-mm-400/order-data-quote-request/)

4) 4 g stainless steel milling balls from Retsch (https://www.retsch.com/products/milling/ball-mills/mixer-mill-mm-400/order-data-quote-request/)

5) IST 500 ball mill jar mountings with the jar (a) removal tool (http://www.insolidotech.org/ist500.html)

6) IST 500 ball mill controls set to 30 Hz and 60 minutes

Initial investigation

Generation of Organomanganese

Initial investigation focussed on the development of organomanganese directly from manganese metal and alkyl halides.

To a 14 mL stainless steel jar was added ethyl-4-bromobutyrate (0.195 g, 1 mmol), manganese pieces, irregular (relevant quantity), additive(s) and a stainless-steel ball (10 mm, 4.1 g) added. The mixture was milled at 30 Hz for the appropriate time. The mixture was then washed into a flask with dichloromethane, aqueous hydrochloric acid (1 M, 25 mL) was added and the mixture stirred for ten minutes. Trifluorotoluene (41 μL, 0.33 mmol) was added as an internal standard, and a sample from the organic layer was passed through a silica plug, washing with diethyl ether.

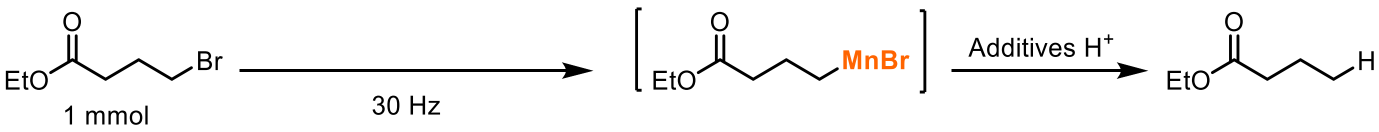

Scheme S1. Organomanganese generation optimisation

Entry	Mn equiv.	Time / h.	Additive (equiv.)	Conversion of XX[a]	GC Yield[a]
1	1.1	1	-	14%	0%
2	1.1	2	-	46%	2%
3	1.1	3	-	100%	28%
4	1.1	4	-	100%	33%
5	2	3	-	100%	43%
6	3	3	-	100%	26%
7	1.1	3	DMA (0.5)	77%	23%
8	1.1	3	DMA (0.75)	75%	25%
9	1.1	3	DMA (1.0)	80%	37%
10	1.1	3	DMA (1.25)	67%	30%
11	1.1	3	DMA (1.5)	84%	16%
12	1.1	3	DMA (2.0)	-	-
13	1.1	3	LiCl (1.0)	52%	21%
14	1.1	3	LiCl (2.0)	33%	12%
15	1.1	3	THF (1.0)	69%	20%
16	1.1	3	THF (2.0)	-	-
**17**	**1.1**	**3**	**THF (1.0) / LiCl (1.0)**	**75%**	**36%**
18	2	3	DMA (1.0)	82%	34%
19	1.1	3	DMF (1.0)	75%	28%
20	1.1	3	DMSO (1.0)	-	-
21	1.1	3	EtOAc (1.0)	0%	0%

22	1.1	3	NMP (1.0)	81%	23%
23	1.1	4	DMA (1.0)	88%	33%
24	1.1	4	THF (1.0) / LiCl (1.0)	97%	38%

Table S1. Initial screening for generation of organomanganese. [a] Measured by GC

Conditions which produced the best yield of the proposed organomanganese (table S1, entry 17) were applied to a one-pot two-step and one pot method for the generation of the organomanganese followed by its trapping with an electrophile. With these conditions various electrophiles were screened.

Electrophile Screen:

One pot two step method

To a 14 mL stainless steel jar was added ethyl-4-bromobutyrate (0.195 g, 1 mmol), manganese pieces, irregular (1.1 mmol, 60 mg), THF (1.0 mmol, 80 µL), LiCl (1.0 mmol, 42 mg) and a stainless-steel ball (10 mm, 4.1 g) added. The mixture was milled at 30 Hz 3 hours. The jar was then opened the relevant electrophile (1 mmol) was added and the mixture milled for a further 3 h. The mixture was then washed into a flask with EtOAc, aqueous hydrochloric acid (1 M, 25 mL) was added, and the mixture stirred for ten minutes. The organic phase was then separated, washed with brine, and dried with magnesium sulphate. The solvent was then removed *en vacuo* and the crude reaction mixture was purified by flash column chromatography.

One pot method

To a 14 mL stainless steel jar was added ethyl-4-bromobutyrate (0.195 g, 1 mmol), manganese pieces, irregular (1.1 mmol, 60 mg), THF (1.0 mmol, 80 µL), LiCl (1.0 mmol, 42 mg), the relevant electrophile (1 mmol) and a stainless-steel ball (10 mm, 4.1 g) added. The mixture was milled at 30 Hz 3 hours. The mixture was then washed into a flask with EtOAc, aqueous hydrochloric acid (1 M, 25 mL) was added, and the mixture stirred for ten minutes. The organic phase was then separated, washed with brine, and dried with magnesium sulphate. The solvent was then removed *en vacuo* and the crude reaction mixture was purified by flash column chromatography.

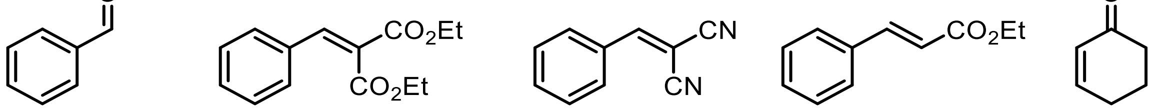

Scheme S2. Electrophile screen

From the one pot reaction using benzylidene malonate the dimer product was isolated as the only product of that reaction. All other reactions involving electrophiles failed to proceed to the desired products.

Optimization of Reductive Dimerisation

To a 14 mL stainless steel milling jar containing the milling ball (10 mm, 4.1 g) was added Manganese, irregular pieces, (1.1 mmol, 60 mg), diethyl 2-(4-fluorobenzylidene)malonate (1 mmol), THF (2 mmol, 160 μL), and anhydrous Lithium Chloride (1 mmol, 42 mg). The reaction mixture was then milled at 30 Hz for 3 h. The mixture was then washed into a flask with EtOAc, aqueous hydrochloric acid (1 M, 25 mL) was added and the mixture stirred for ten minutes. The organic phase was then separated, washed with brine and dried with magnesium sulphate. Trifluoro toluene (0.33 mmol, 41 μL) was added as an internal standard and crude ^{19}F NMR was taken.

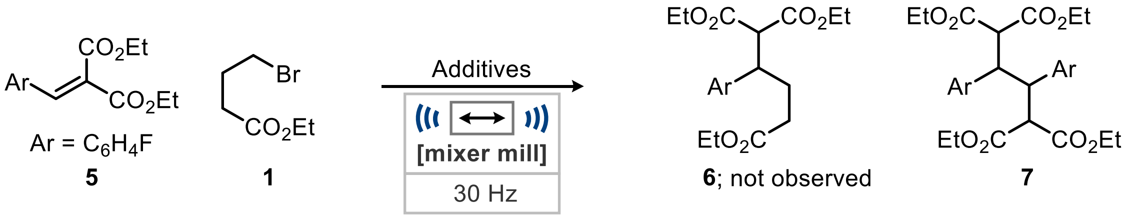

Scheme S3. Reductive dimerization optimisation

Entry	Reductant	Equiv.	LAG	Equiv.	Equiv. of LiCl	Equiv. of 1	GA mg	Time / h	Yield of 7
**1**	Mn	1.1	THF	1	1	1		3	85%
**2**	Mn	1.1	NMP	1	1	1		3	15%
**3**	Mn	1.1	DCM	1	1	1		3	0%
**4**	Mn	1.1	IPA	1	1	1		3	67%
**5**	Mn	1.1	DMF	1	1	1		3	0%
**6**	Mn	1.1	MeCN	1	1	1		3	0%
**7**	Mn	1.1	Et2O	1	1	1		3	0%
**8**	Mn	1.1	THF	1	1	1		3	85%
**9**	Mn	1.1	THF	1	1	0.5		3	86%
**10**	Mn	1.1	THF	1	1	0		3	10%
**11**	Mn	1.1	THF	1	1	0		3	10%
**12**	**Mn**	**1.1**	**THF**	**2**	**1**	**0**		**3**	**80% (80%)**
**13**	Mn	1.1	THF	3	1	0		3	43%
**14**	**Mn**	**1.1**	**THF**	**2**	**1**	**0**		**3**	**80% (80%)**

**15**	Mn	0.55	THF	2	1	0			3	47%
**16**	Mn	2	THF	2	1	0			3	63%
**17**	Zn	1.1	THF	2	1	0			3	43%
**18**	**Mn**	**1.1**	**THF**	**2**	**1**	**0**			**3**	**80% (80%)**
**19**	Mn	1.1	THF	1	0	0			3	0%
**20**	Mn	1.1	THF	2	1 (MgOTf2)	0			3	0%
**21**	Mn	1.1	THF	2	1 (ZnCl2)	0			3	63%
**22**	Mn	1.1	THF	2	1 (ScOTf3)	0			3	0%
**23**	Mn	1.1	THF	2	1 (ScOTf3)	0			3	0%
**24**	Mn	1.1	THF	2	1 (PyrOTf)	0			3	5%
**25**	Mn	1.1	THF	2	1 (LiBr)	0			3	66%
**26**	Mn	1.1	THF	2	1 (LiI)	0			3	0%
**27**	Mn	1.1	THF	2	1 (LiOTf)	0			3	0%
**28**	Mn	1.1	THF	2	1 (MnCl2)	0			3	0%
**29**	Mn	1.1	THF	2	1 (Mn(OTf)2)	0			3	0%
**30**	Zn	1.1	THF	2	1 (MnCl2)	0			3	11%
**31**	Zn	1.1	THF	2	1 (ZnCl2)	0			3	43%
**32**	Mn	1.1	THF	2	0.5	0			3	72%
**33**	Mn	1.1	THF	2	1.5	0			3	68%
**34**	**Mn**	**1.1**	**THF**	**2**	**1**	**0**			**3**	**80% (80%)**
**35**	Mn	1.1	THF	1	1	1	NaCl	266	3	0%
**36**	Mn	1.1	THF	1	6.3	1	LiCl	266	3	0%
**37**	Mn	1.1	THF	1	1	1	Sand	266	3	81%
**38**	Mn	1.1	THF	1	1	1	MgSO4	266	3	0%
**39**	Mn	1.1	THF	1	1	1	Celite	266	3	72%
**40**	Mn	1.1	THF	1	1	1	Alumina	266	3	10%
**41**	Mn	1.1	THF	2	1	0			0.08	0%
**42**	Mn	1.1	THF	2	1	0			0.25	4%
**43**	Mn	1.1	THF	2	1	0			0.5	9%
**44**	Mn	1.1	THF	2	1	0			0.75	16%
**45**	Mn	1.1	THF	2	1	0			1	30%
**46**	Mn	1.1	THF	2	1	0			1.5	58%
**47**	Mn	1.1	THF	2	1	0			2	84%
**48**	**Mn**	**1.1**	**THF**	**2**	**1**	**0**			**3**	**80% (80%)**

**49**	Mn	1.1	THF	2	1	0			4	72%

Table S2. Optimization of reductive dimerization of 4-fluorobenzylidene malonate

Cyclic Voltammetry Study

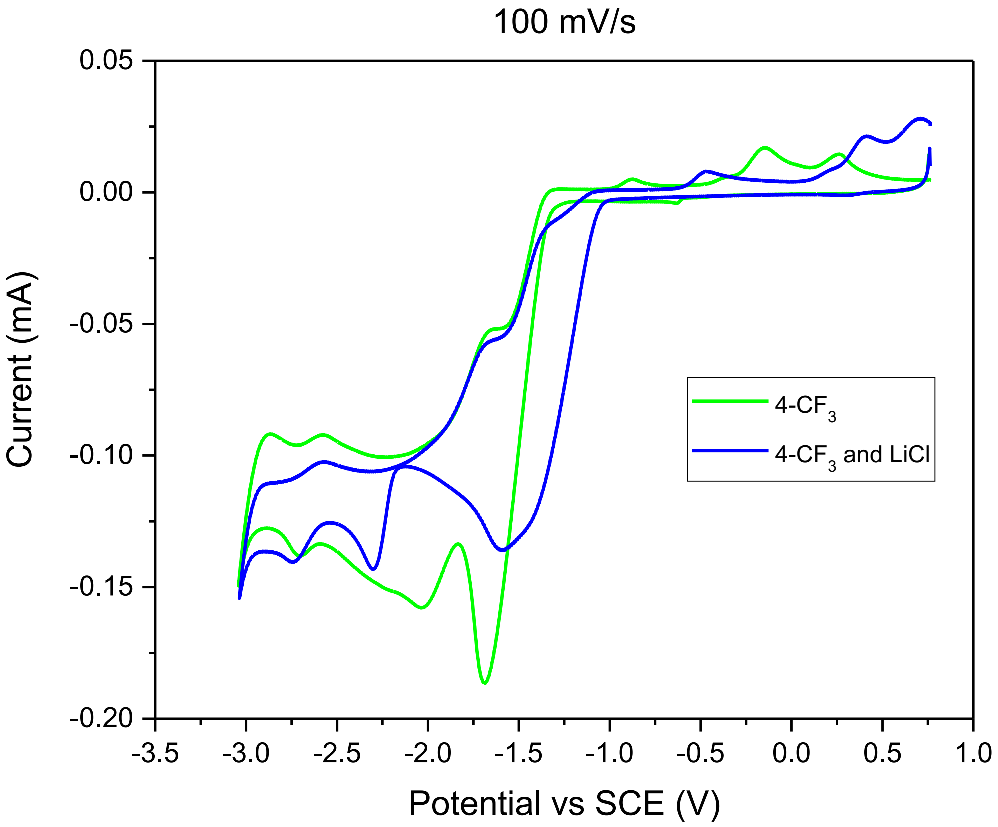

Figure S1. Cyclic voltammogram of diethyl 2-(4-(trifluoromethyl)benzylidene)malonate (5 mM) in MeCN as solvent and Tetra *n*-butyl ammonium hexafluorophosphate (0.1 M) as electrolyte without LiCl (1 mM) (light green line) and with LiCl (1 mM) (dark blue line).

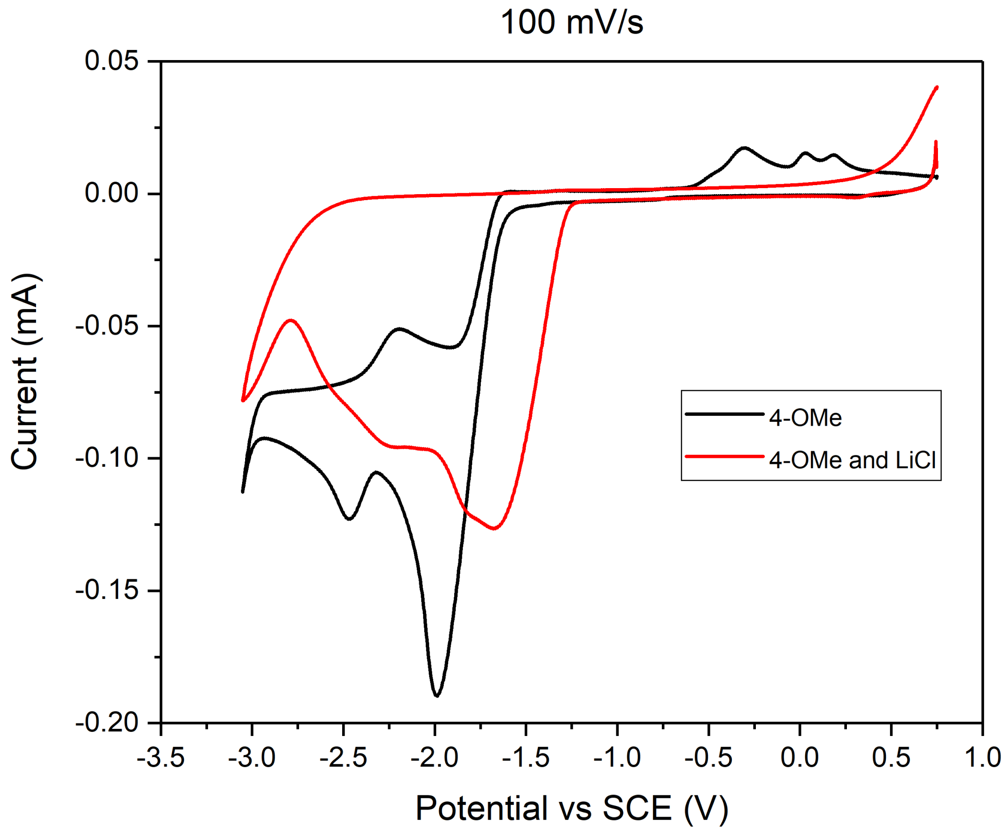

Figure S2. Cyclic voltammogram of diethyl 2-(4-methoxybenzylidene)malonate (5 mM) in MeCN as solvent and Tetra *n*-butyl ammonium hexafluorophosphate (0.1 M) as electrolyte without LiCl (1 mM) (black line) and with LiCl (1 mM) (red line).

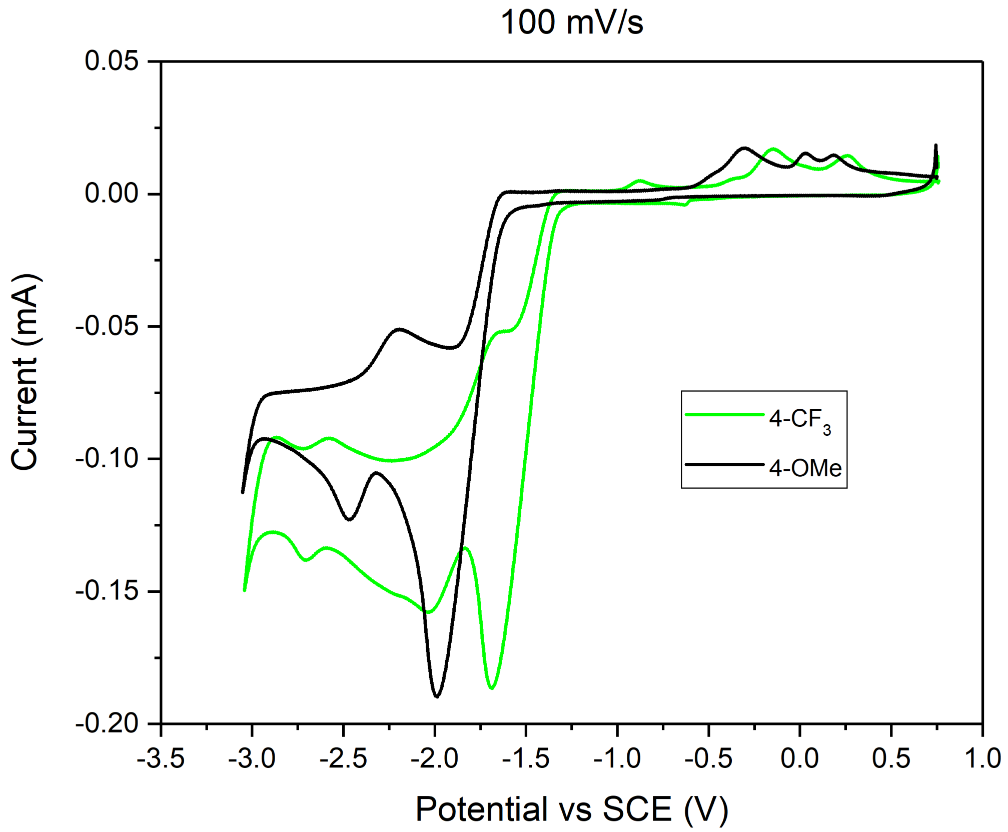

Figure S3. Cyclic voltammogram of diethyl 2-(4-methoxybenzylidene)malonate (black line), and diethyl 2-(4-(trifluoromethyl)benzylidene)malonate (light green line), (5 mM) in MeCN as solvent and Tetra *n*-butyl ammonium hexafluorophosphate (0.1 M) as electrolyte.

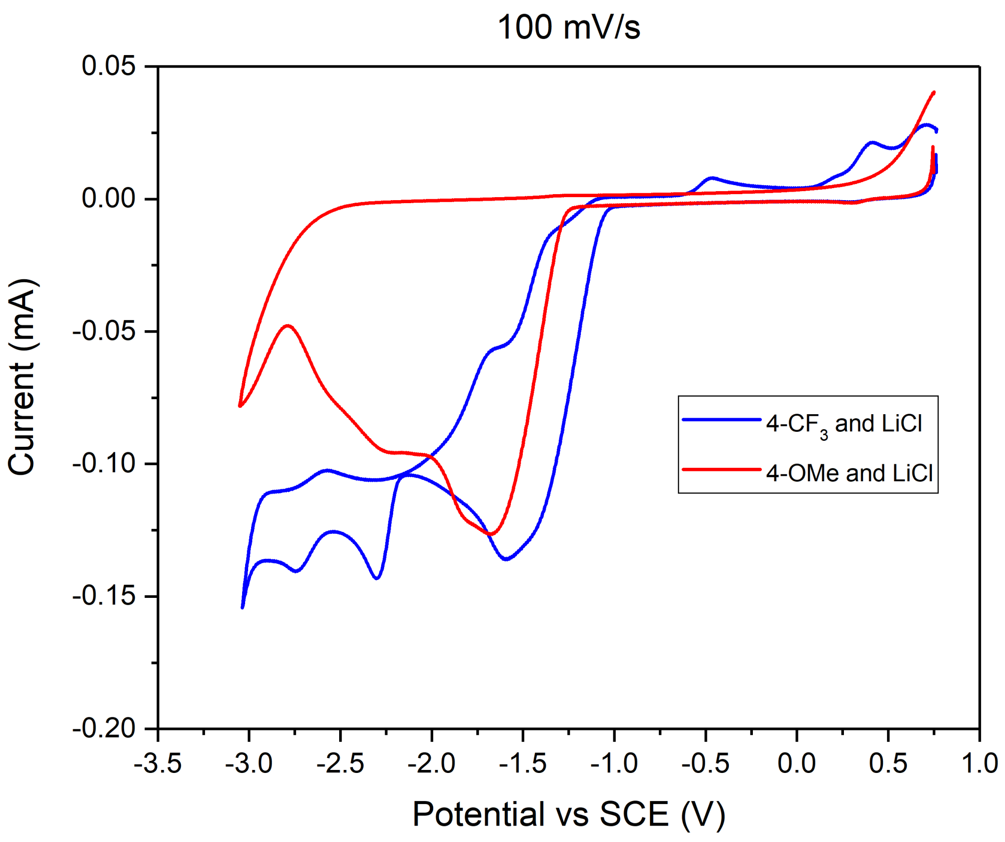

Figure S4. Cyclic voltammogram of diethyl 2-(4-methoxybenzylidene)malonate (red line), and diethyl 2-(4-(trifluoromethyl)benzylidene)malonate (dark blue line) (5 mM) in MeCN as solvent and Tetra *n*-butyl ammonium hexafluorophosphate (0.1 M) as electrolyte with LiCl (1 mM).

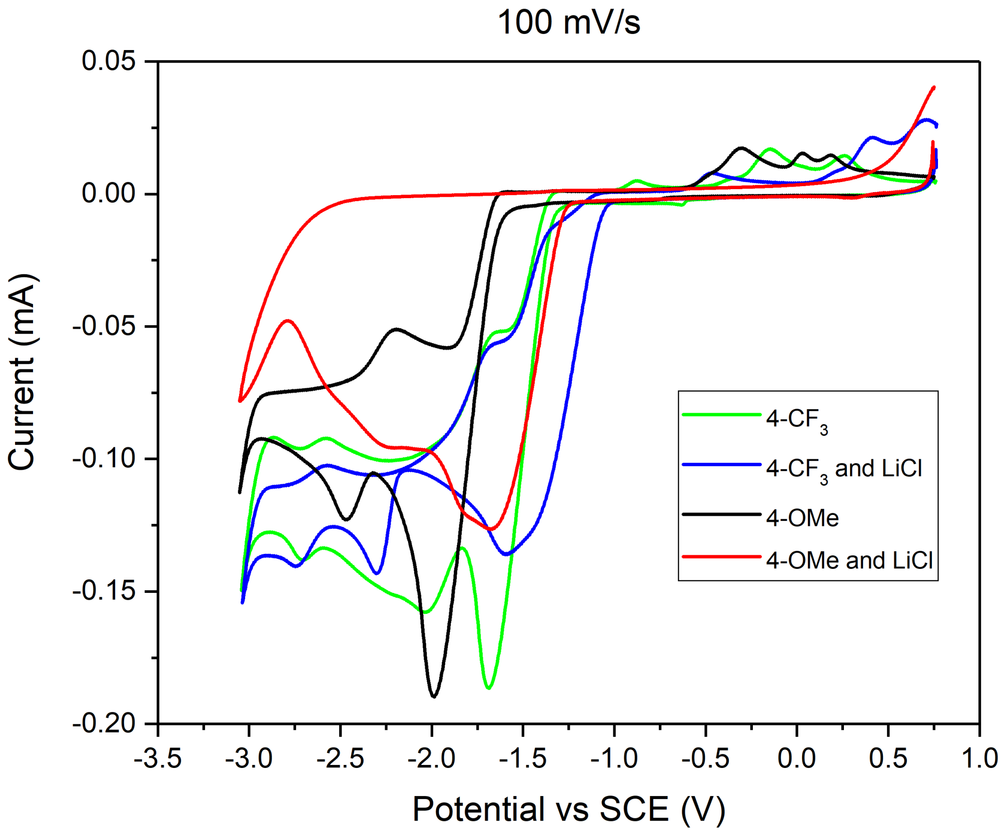

Figure S5. Cyclic voltammogram of, diethyl 2-(4-methoxybenzylidene)malonate (black line), diethyl 2-(4-methoxybenzylidene)malonate with LiCl (red line), diethyl 2-(4-(trifluoromethyl)benzylidene)malonate (light green line), and diethyl 2-(4-(trifluoromethyl)benzylidene)malonate with LiCl (dark blue line), (5 mM) in MeCN as solvent and Tetra *n*-butyl ammonium hexafluorophosphate (0.1 M) as electrolyte with and without LiCl (1 mM).

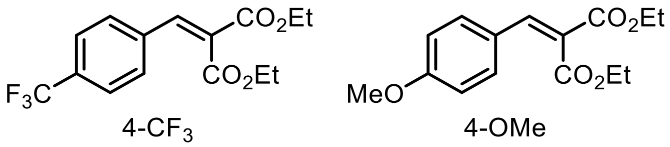

Scheme S4. Substrates selected for CV analysis

	Substrate reduction potential / V	
LiCl	4-CF_3	4-OMe
Without	-1.27	-1.62
With	-1.21	-1.25
Difference	0.06	0.37

Table S3. Reduction potential of a range of benzylidene malonates Vs. Fc/Fc$^+$

$Mn^{2+} + 2e^- \longrightarrow Mn(s)$ $E_{1/2} = -1.185$ vs SCE

$Zn^{2+} + 2e^- \longrightarrow Zn(s)$ $E_{1/2} = -0.76$ vs SCE

$Fc^+ + e^- \longrightarrow Fc(s)$ $E_{1/2} = 0.380$ vs SCE[2]

As the reduction potentials of the substrates were measured against Fc/Fc$^+$ they were converted to against SCE with the following equation. This was to make them directly comparable to that of the known value for manganese metal.

$$E_{1/2}(Substrate\ vs. SCE) = E_{1/2}(Substrate\ vs. Fc/Fc^+) - E_{1/2}(Fc/Fc^+\ vs.\ SCE)$$

	Substrate reduction potential / V	
LiCl	4-CF_3	4-OMe
Without	-1.65	-2.00
With	-1.59	-1.63
Difference	0.06	0.37

Table S3. Reduction potential of a range of benzylidene malonates Vs. SCE

[2] R. Francke, R. D. Little, *Chem. Soc. Rev.* **2014**, 43, 2492 – 2521

EPR Experiments

To a 14 mL stainless steel milling jar containing the milling ball (10 mm, 4.1 g) was added manganese, irregular pieces, (1.1 mmol, 60 mg), diethyl 2-(4-fluorobenzylidene)malonate (1 mmol, 266 mg), THF (2 mmol, 160 μL), and lithium chloride (1 mmol, 42 mg). The reaction mixture was milled at 30 Hz for 3 h. The resulting crude product mixture was washed with EtOAc, prior to full work-up using aqueous HCl (1 M, 25 mL) to induce separation of the product into organic and aqueous phases. The reaction mixture prior to milling resulted in the absence of an EPR spectrum, due to line broadening effects arising from the conduction properties of the Mn(0) pieces. Further confirmation of this was provided by a similar lack of observable EPR signal recorded on a solid manganese strip.

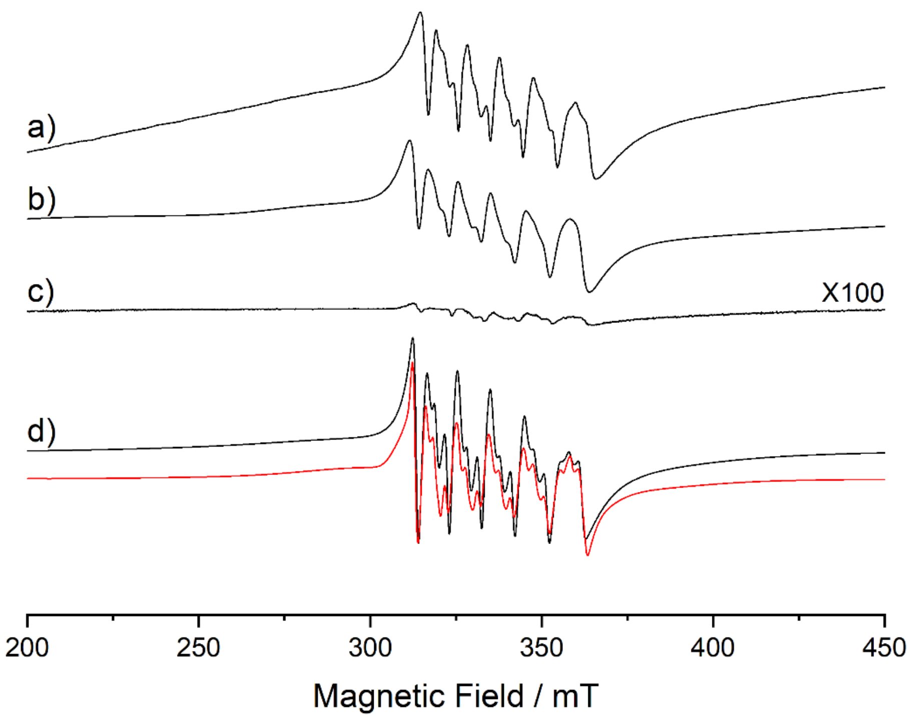

Figure S7. CW X-band EPR spectra (T = 120 K) of **a)** Crude reaction mixture dissolved in ethyl acetate, **b)** aqueous layer after work-up with HCl, **c)** organic layer after work-up; **d)** $Mn(OAc)_2$ (10 mM) in a water:glycerol (50:50) solvent, and red trace showing a simulation of $Mn(OAc)_2$. A simulation of $Mn(OAc)_2$ has been included above for comparative purposes, showing the extremely similarities in spectral appearance between the crude product and its Mn(OAc)2 counterpart.

The EPR spectra of the reaction products after various treatments are presented in Figure S7. As can be seen, there is evidence of a strong EPR signal of an ethyl acetate solution of the crude reaction product (Fig S7a). The well-resolved six hyperfine lines originate from a high spin d^5 Mn (II) centre with nuclear spin value $I(^{55}Mn) = {}^5/_2$ (100 % abundance). The broad baseline effects visible on either side of the main signal originate from zero-field splitting effects, and the additional splitting superimposed on each of the six main lines arises from fine structure, both of which are typical for high-spin systems (e.g. high-spin d^5).

It is anticipated that any source of oxidised Mn generated during the course of the reaction would remain in the aqueous layer. Hence, the crude reaction product underwent a full work-up with HCl to afford separation of the organic and aqueous layers. The EPR spectrum of the aqueous layer does indeed show a strong Mn (II) signal (Fig S7b), whereas a much weaker signal is observed in the EPR spectrum of the organic layer (Fig S7c), which most likely originates from incomplete separation of the phases during work-up. Given the potential reaction pathways (involving either double electron transfer, or sequential SET processes), the most plausible identity of the oxidised manganese species involves coordination of the malonate. Hence, the spectrum of a pure sample of $Mn(OAc)_2$ is presented in Fig S7d for comparison, which closely resembles that of the crude reaction product (Fig S7a).

The dominant factor in the spin Hamiltonian parameters of high-spin systems is the zero-field interaction, arising from interaction between the multiple unpaired electrons. Spin-orbit coupling is spin-forbidden in d^5 systems (in which all 3d-orbitals are singly occupied and therefore half-filled), therefore the g-tensor is typically predominantly isotropic and close to the free-electron g-value ($g_{iso} \sim 2.0023$). Unfortunately, in the case of Mn (II) systems, electron spin delocalisation onto surrounding ligand nuclei is typically small and therefore the resulting superhyperfine coupling is typically unresolved within the linewidth of the main hyperfine features. A simulation of the $Mn(OAc)_2$ experimental spectrum is presented in Fig S7e, performed using the Easyspin toolbox within Matlab, with the following spin Hamiltonian parameters:

$g_{iso} = 2.008$, A = [-283.74, -265.4 -268.2] MHz, D = [-603.8, -139.4] MHz. Values are consistent with highly isotropic Mn (II) systems.[3]

In addition to following the redox process taking place at the metal centre, we performed spin trap experiments with the aim of identifying any C-based radical intermediates that could prove

[3] a) T. A. Stich, S. Lahiri, G. Yeagle, M. Dicus, M. Brynda, A. Gunn, C. Aznar, V. J. DeRose and R. D. Britt, *Appl. Magn. Reson.*, 2007, **31**, 321–341; b) C. Duboc, M. N. Collomb and F. Neese, *Appl. Magn. Reson.*, 2010, **37**, 229–245; c) K. Keller, M. Zalibera, M. Qi, V. Koch, J. Wegner, H. Hintz, A. Godt, G. Jeschke, A.Savitsky and M. Yulikov, *Phys. Chem. Chem. Phys.*, 2016, **18**, 25120–25135.

the multiple sequential SET pathway. EPR spectra were recorded on the crude reaction performed in the presence of PBN spin trap, however unfortunately the data were inconclusive and are not reported here for brevity.

Control Reactions

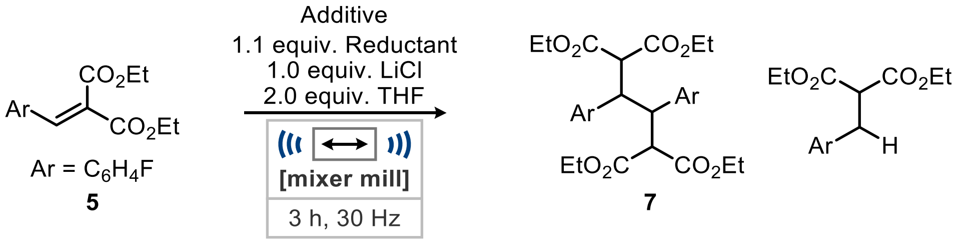

Scheme S5. Control reactions

Entry	Additive (equiv.)	Conversion of SM[a]	Yield of XX[a]	Yield of XX[a]
1	1.1 mmol Mn, 1mmol LiCl, 2 mmol THF, 1 mmol water	95%	20%	0%
2	1.1 mmol Zn 1 mmol LiCl, 1 mmol H_2O, 2 mmol THF	100%	4%	41%
3	1.1 mmol Zn, 2 mmol THF, 1 mmol LiCl	83%	43%	0%
4	1.1 mmol Zn, 1 mmol MnCl-$_2$4H_2O, 2 mmol THF, 1 mmol LiCl	100%	18%	51%
5	1.1 mmol Mn, 1 mmol MnCl-$_2$4H_2O, 2 mmol THF, 1 mmol LiCl	82%	19%	0%
6	1.1 mmol Mn, 2 mmol THF, 1 mmol LiCl, 1 mmol TEMPO	0%	0%	0%
7	1.1 mmol Mn, 2 mmol THF, 1 mmol LiCl, 1 mmol TEMPO	48%	37%	0%
8[b]	1.1 mmol Mn, 2 mmol THF, 1 mmol LiCl, 1 mmol 1,1-diphenlyethylene	8%	Trace	0%
9	1.1 mmol Mn, 2 mmol THF, 1 mmol LiCl, 1 mmol 1,1-diphenlyethylene	60%	53%	0%
10[b]	1.1 mmol Mn, 2 mmol THF, 1 mmol LiCl, 1 mmol TMSCl	5%	Trace	0%
11	1.1 mmol Mn, 2 mmol THF, 1 mmol LiCl, 1 mmol TMSCl	43%	32%	0%
12[b]	1.1 mmol Mn, 2 mmol d^8 THF, 1 mmol LiCl	100%	76%[c]	0%

Table S4. Control reactions [a] determined by ^{19}F NMR using 0.33 mmol of trifluorotoluene as an internal standard [b] TEMPO/1,1-diphenylethylene/TMSCl added after 1.5 h of milling and milling then continued for a further 1.5 h [c] no deuteration incorporation in product.

Through these control reactions it was established that when the reaction was mediated by zinc metal that the reduced intermediate could be easily protonated by water present in the reaction. Whereas the intermediate present when manganese metal was used as the reductant could not be protonated by the presence of water although the water in the reaction did lead to a decrease in conversion and yield of the dimer.

Unproductive Substrates

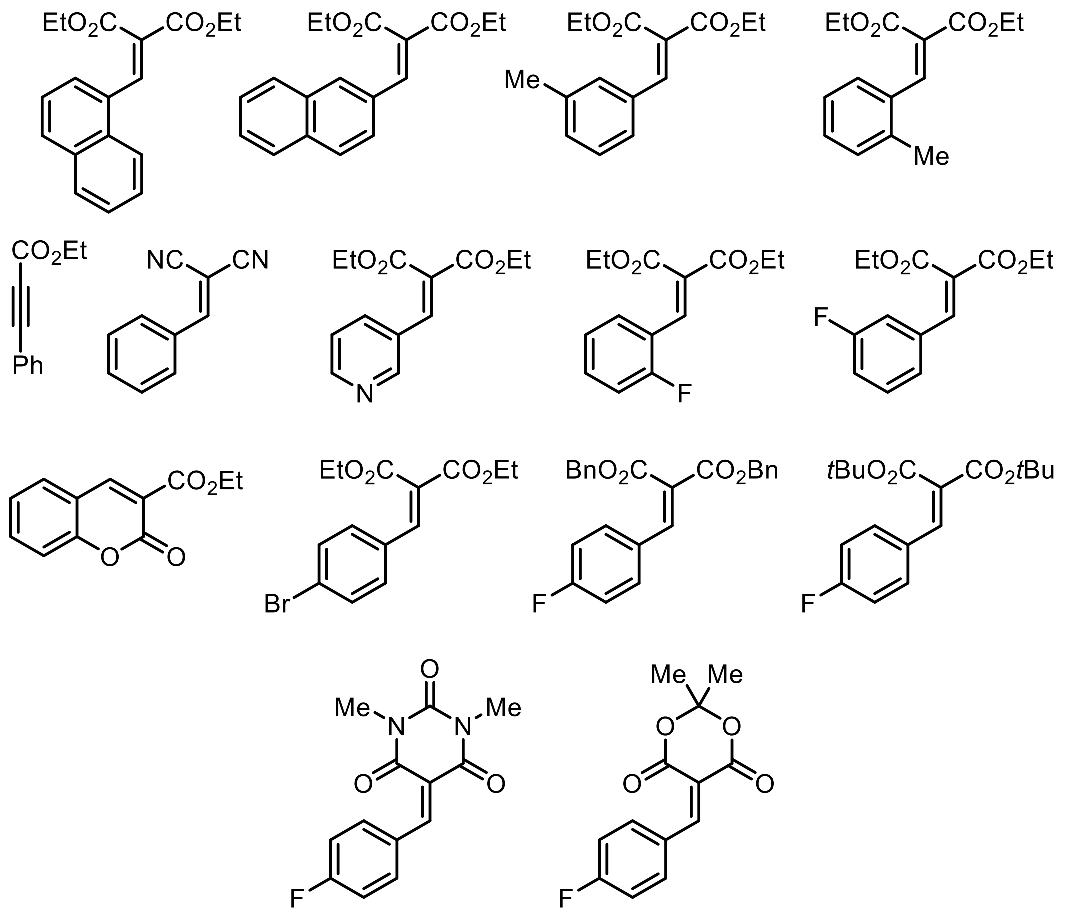

Many of the unproductive substrates in this reaction have higher reduction potentials than that of the optimized substrate meaning they may fall outside of the window of amenable compounds for the reaction. Furthermore, more sterically demanding substrates also failed to undergo the reaction showing its sensitivity to traditional steric demands. Finally, the substrate bearing the aryl bromide provided a complex mixture of products which could not be separated with some having been de-halogenated suggestive of manganese insertion into the C-Br bond followed by protonation.

Characterization Data

Starting material Synthesis

Diethyl 2-(4-fluorobenzylidene)malonate (5)

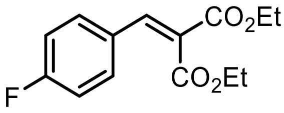

The title compound was prepared using a method modified from the literature.[4] To a 250 mL round bottom flask was added toluene (100mL, 0.5 M), diethyl malonate (50 mmol), 4-fluorobenzaldehyde (60 mmol), acetic acid (5 mmol), and pyrrolidine (5 mmol). The flask was then equipped with a Dean-Stark apparatus and vigorously refluxed for 16 h. The solvent was then removed *en vacuo* and the residual oil was dissolved in diethyl ether (100 mL) and washed with sat. bicarbonate (100 mL), water (100 mL), and brine (100 mL). The organic phase was then dried using magnesium sulphate and solvent removed *en vacuo.* The remaining volatile impurities were removed via kugelrohr distillation (120 ˚C, 80 mbar) giving the title compound as a pale-yellow oil (92%, 12.2 g). **^{1}H NMR** (500 MHz, $CDCl_3$) δ 7.68 (s, 1H), 7.49 – 7.41 (m, 2H), 7.10 – 7.02 (m, 2H), 4.34 (q, *J* = 7.1 Hz, 2H), 4.30 (q, *J* = 7.1 Hz, 2H), 1.33 (t, *J* = 7.1 Hz, 3H), 1.29 (t, *J* = 7.1 Hz, 3H). **^{13}C NMR** (126 MHz, $CDCl_3$) δ 166.7 (s), 164.2 (s), 164.0 (d, *J* = 252.6 Hz), 140.9 (s), 131.7 (d, *J* = 8.7 Hz), 129.3 (d, *J* = 3.4 Hz), 126.2 (s), 116.2 (d, *J* = 21.9 Hz), 61.9 (s), 61.8 (s), 14.3 (s), 14.1 (s). **^{19}F NMR** (376 MHz, $CDCl_3$) δ -108.7. **HRMS** (EI+) [$C_{14}H_{15}O_4F$] calc. 266.0954 found 266.0955. Characterization data is in accordance with prior reports.[5]

General Procedure A: Synthesis of Arylidene Malonates

The title compound was prepared using a method modified from the literature.[6] A 25 ml vial equipped with a stirrer bar and 250 wt % activated 3 Å molecular sieves (beads, 0.12 - 0.20 in) was flame dried. Aldehyde (6 mmol) and malonate (5 mmol) was added to the vial followed by dry CH_2Cl_2 (5 mL, 1.0 M). Stirring was started followed by the addition of acetic acid (0.5 mmol) and pyrrolidine (0.5 mmol). The mixture was stirred overnight to 4 days. The reaction

[4] S. Hajra, S. M. Aziz, R. Maji, *RSC Adv*. **2013**, 3, 10185-10188
[5] J. Wang, Y. Zhou, L. Zhang, Z. Li, X. Chen, H. Liu, *Org. Lett.* **2013**, 15, 1508-1511
[6] R. C. Betori, B. R. McDonald, K. A. Scheidt, *Chem. Sci.* **2019**, 10, 3353–3359

mixture was filtered followed by passing through a silica plug. The product was then either used without purification or purified by column chromatography/crystallization/distillation.

Diethyl 2-(4-methoxybenzylidene)malonate

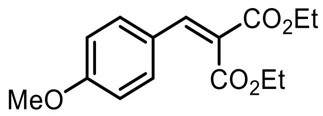

The title compound was synthesised by general procedure A stirring for 4 days and purified by kugelrohr distillation (95 ˚C, <1 mbar) (49%, 0.682 g) as a colourless oil. **^{1}H NMR** (500 MHz, $CDCl_3$) δ 7.67 (s, 1H), 7.45 – 7.40 (m, 2H), 6.92 – 6.86 (m, 2H), 4.36 (q, J = 7.1 Hz, 2H), 4.29 (q, J = 7.1 Hz, 2H), 3.83 (s, 3H), 1.36 – 1.27 (m, 6H). **^{13}C NMR** (126 MHz, $CDCl_3$) δ 167.3, 164.6, 161.7, 141.9, 131.7, 125.6, 123.8, 114.4, 61.8, 61.6, 55.5, 14.3, 14.1. **HRMS** (EI+) [$C_{15}H_{18}O_5$] calc. 278.1154 found 278.1153. Characterization data is in accordance with prior reports.[5]

Diethyl 2-(4-(methoxycarbonyl)benzylidene)malonate

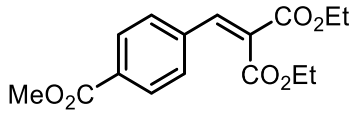

The title compound was synthesised by general procedure A stirring overnight and purified by column chromatography (Hexane:EtOAc) (44%, 0.671 g) as a colourless oil. **^{1}H NMR** (500 MHz, $CDCl_3$) δ 8.04 (d, *J* = 7.9 Hz, 1H), 7.75 (s, 1H), 7.51 (d, *J* = 7.9 Hz, 1H), 4.32 (q, *J* = 7.1 Hz, 2H), 3.93 (s, 2H), 1.34 (t, *J* = 7.1 Hz, 1H), 1.27 (t, *J* = 7.0 Hz, 2H). **^{13}C NMR** (126 MHz, $CDCl_3$) δ 166.5, 166.3, 163.9, 140.9, 137.4, 131.6, 130.1, 129.3, 128.5, 62.0, 52.5, 14.3, 14.0. **HRMS** (EI+) [$C_{16}H_{18}O_6$] calc. 306.1103 found 306.1102.

Diethyl 2-(4-cyanobenzylidene)malonate

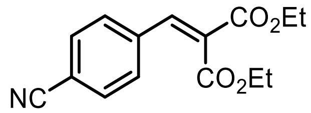

The title compound was synthesised by general procedure A stirring overnight and purified by recrystallization from Et_2O (55%, 0.743 g) as a yellow solid. **MP** 69-72 ˚C. **^{1}H NMR** (500 MHz, $CDCl_3$) δ 7.71 (s, 1H), 7.69 – 7.65 (m, 2H), 7.54 (dd, *J* = 8.7, 0.6 Hz, 2H), 4.32 (q, *J* = 7.1 Hz, 4H), 4.32 (q, *J* = 7.1 Hz, 4H), 1.34 (t, *J* = 7.1 Hz, 3H), 1.27 (t, *J* = 7.1 Hz, 3H). **^{13}C NMR** (126 MHz, $CDCl_3$) δ 165.9, 163.6, 139.7, 137.6, 132.6, 129.8, 129.7, 118.3, 113.8, 62.2, 62.2, 14.2,

14.0. **HRMS** (EI+) [$C_{15}H_{15}NO_4$] calc. 273.1001 found 273.0998. Characterization data is in accordance with prior reports. [5]

Diethyl 2-(4-bromobenzylidene)malonate

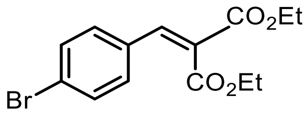

The title compound was synthesised by general procedure A stirring overnight and purified by recrystallization from hexane (78%, 1.278 g) as a colourless solid. **MP** 39-41 ˚C. **^{1}H NMR** (500 MHz, $CDCl_3$) δ 7.65 (s, 1H), 7.54 – 7.47 (m, 2H), 7.34 – 7.29 (m, 2H), 4.33 (q, *J* = 7.1 Hz, 3H), 4.30 (q, *J* = 7.1 Hz, 3H), 1.33 (t, *J* = 7.1 Hz, 3H), 1.29 (t, *J* = 7.1 Hz, 3H). **^{13}C NMR** (126 MHz, $CDCl_3$) δ 166.4, 163.9, 140.7, 132.1, 131.9, 130.8, 127.0, 125.0, 61.9, 61.8, 14.1, 13.9. **HRMS** (ES+) [$C_{14}H_{15}O_4Br$ + Na^+] calc. 349.0051 found 349.0052. Characterization data is in accordance with prior reports. [5]

Diethyl 2-(4-chlorobenzylidene)malonate

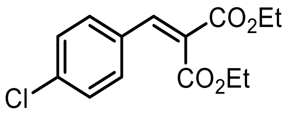

The title compound was synthesised by general procedure A stirring overnight (90%, 1.29 g) as a colourless oil. **^{1}H NMR** (500 MHz, $CDCl_3$) δ 7.67 (s, 1H), 7.41 – 7.37 (m, 2H), 7.37 – 7.33 (m, 2H), 4.33 (q, *J* = 7.1 Hz, 3H), 4.30 (q, *J* = 7.1 Hz, 3H), 1.33 (t, *J* = 7.1 Hz, 3H), 1.29 (t, *J* = 7.1 Hz, 3H). **^{13}C NMR** (126 MHz, $CDCl_3$) δ 166.6, 164.1, 140.8, 136.8, 131.5, 130.8, 129.3, 127.0, 62.0, 61.9, 14.3, 14.1. **HRMS** (ES+) [$C_{14}H_{15}O_4$ + Na^+] calc. 305.0557 found305.0556. Characterization data is in accordance with prior reports. [5]

Diethyl 2-(4-(trifluoromethyl)benzylidene)malonate

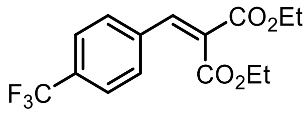

The title compound was synthesised by general procedure A stirring overnight (quant., 1.58 g) as a colourless solid that went brown over time and was stored under nitrogen at 0 ˚C. **MP** 44 - 47 ˚C. **^{1}H NMR** (400 MHz, $CDCl_3$) δ 7.74 (s, 1H), 7.64 (d, *J* = 8.3 Hz, 1H), 7.56 (d, *J* = 8.3 Hz, 1H), 4.33 (q, *J* = 7.1 Hz, 2H), 4.32 (q, *J* = 7.1 Hz, 2H), 1.34 (t, *J* = 7.1 Hz, 1H), 1.28 (t, *J* = 7.1 Hz, 2H). **^{13}C NMR** (126 MHz, $CDCl_3$) δ 166.1 (s), 163.8 (s), 140.4 (s), 136.6 (s), 132.0 (q, *J* = 32.7 Hz), 129.6 (s, *J* = 17.7 Hz), 128.9 (s), 125.9 (q, *J* = 3.7 Hz), 123.8 (q, *J* = 272.3 Hz),

62.1 (s, *J* = 3.2 Hz), 62.1 (s), 14.2 (s, *J* = 29.1 Hz), 14.0 (s). **^{19}F NMR** (376 MHz, $CDCl_3$) δ -63.0 (s). **HRMS** (ES+) [$C_{15}H_{15}O_4F_3$ + Na^+] calc. 339.0820 found 339.0814. Characterization data is in accordance with prior reports.[7]

Diethyl 2-(4-methylbenzylidene)malonate

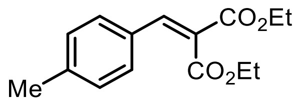

The title compound was synthesised by general procedure A stirring overnight and purified by recrystallization from from Et_2O and hexane (82%, 1.08 g) as a colourless solid. **MP** 41 - 43 ˚C. **^{1}H NMR** (500 MHz, $CDCl_3$) δ 7.70 (s, 1H), 7.35 (d, *J* = 8.3 Hz, 1H), 7.18 (d, *J* = 8.1 Hz, 1H), 4.34 (q, *J* = 7.1 Hz, 1H), 4.30 (q, *J* = 7.1 Hz, 1H), 2.37 (s, 2H), 1.33 (t, *J* = 6.1 Hz, 1H), 1.30 (t, *J* = 6.1 Hz, 1H). **^{13}C NMR** (126 MHz, $CDCl_3$) δ 167.1, 164.5, 142.3, 141.3, 130.2, 129.7, 129.7, 125.3, 61.8, 61.7, 21.6, 14.3, 14.1. **HRMS** (ES+) [$C_{15}H_{18}O_4$ + Na^+] calc. 285.1103 found 285.1105. Characterization data is in accordance with prior reports.[5]

Diethyl 2-(2-fluorobenzylidene)malonate

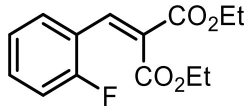

The title compound was synthesised by general procedure A stirring overnight and purified by kugelrohr distillation (90 ˚C, <1 mbar) (48%, 1.28 g) as a pale-yellow oil. **^{1}H NMR** (500 MHz, $CDCl_3$) δ 7.91 (s, 1H), 7.44 (td, *J* = 7.6, 1.7 Hz, 1H), 7.41 – 7.35 (m, 1H), 7.17 – 7.05 (m, 2H), 4.31 (q, *J* = 7.1 Hz, 2H), 4.30 (q, *J* = 7.1 Hz, 2H), 1.34 (t, *J* = 7.1 Hz, 3H), 1.26 (t, *J* = 7.1 Hz, 3H). **^{13}C NMR** (126 MHz, $CDCl_3$) δ 166.3 (s), 163.0 (s), 161.0 (d, *J* = 253.3 Hz), 135.0 (d, *J* = 5.2 Hz), 132.5 (d, *J* = 8.7 Hz), 129.5 (d, *J* = 2.1 Hz), 128.2 (d, *J* = 1.5 Hz), 124.4 (d, *J* = 3.7 Hz), 121.4 (d, *J* = 12.5 Hz), 116.1 (d, *J* = 21.7 Hz), 62.0 (s), 61.9 (s), 14.3 (s), 14.0 (s). **^{19}F NMR** (376 MHz, $CDCl_3$) δ -112.70. Characterization data is in accordance with prior reports.[8]

Diethyl 3-(3-fluorobenzylidene)malonate

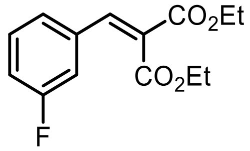

[7] A. Kazia, R. Melngaile, A. Mishnev, J. Velkis, *Org. Biomol. Chem.* **2020**, 18, 1384-1388
[8] S. Ghosh, C. K. Jana, *Org. Biomol. Chem.* **2019**, 17, 10153-10157

The title compound was synthesised by general procedure A stirring overnight and purified by kugelrohr distillation (90 ˚C, <1 mbar) (68%, 1.81 g) as a pale yellow oil. **^{1}H NMR** (500 MHz, $CDCl_3$) δ 7.68 (s, 1H), 7.35 (td, *J* = 8.0, 5.9 Hz, 1H), 7.23 (dd, *J* = 7.7, 0.7 Hz, 1H), 7.18 – 7.14 (m, 1H), 7.10 (tdd, *J* = 8.3, 2.5, 0.8 Hz, 1H), 4.34 (q, *J* = 7.1 Hz, 1H), 4.31 (q, *J* = 7.1 Hz, 1H), 1.33 (t, *J* = 7.1 Hz, 1H), 1.30 (t, *J* = 7.1 Hz, 1H). **^{13}C NMR** (126 MHz, $CDCl_3$) δ 166.4 (s), 163.9 (s), 162.8 (d, J = 247.0 Hz), 140.7 (d, J = 2.6 Hz), 135.1 (d, J = 7.9 Hz), 130.5 (d, J = 8.3 Hz), 127.7 (s), 125.5 (d, J = 3.0 Hz), 117.6 (d, J = 21.3 Hz), 115.9 (d, J = 22.5 Hz), 62.0 (s), 62.0 (s), 14.3 (s), 14.0 (s). **^{19}F NMR** (376 MHz, $CDCl_3$) δ -112.09.

Diethyl 2-(2-methylbenzylidene)malonate

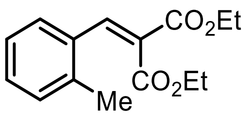

The title compound was synthesised by general procedure A stirring overnight and purified by kugelrohr distillation (95 ˚C, <1 mbar) (37%, 0.969 g) as a colourless oil. **^{1}H NMR** (500 MHz, $CDCl_3$) δ 7.97 (s, 1H), 7.33 (d, *J* = 7.7 Hz, 1H), 7.27 (td, *J* = 7.5, 1.3 Hz, 1H), 7.22 – 7.18 (m, 1H), 7.18 – 7.12 (m, 1H), 4.31 (q, *J* = 7.1 Hz, 2H), 4.22 (q, *J* = 7.1 Hz, 2H), 2.38 (s, 3H), 1.34 (t, *J* = 7.1 Hz, 3H), 1.16 (t, *J* = 7.1 Hz, 3H). **^{13}C NMR** (126 MHz, $CDCl_3$) δ 166.5, 164.2, 141.9, 137.7, 132.7, 130.5, 130.2, 127.9, 127.8, 126.1, 61.8, 61.6, 20.1, 14.3, 14.0. Characterization data is in accordance with prior reports.[4]

Diethyl 3-(3-methylbenzylidene)malonate

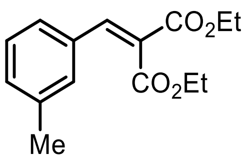

The title compound was synthesised by general procedure A stirring overnight and purified by kugelrohr distillation (95 ˚C, <1 mbar) (63%, 1.65 g) as a colourless oil. **^{1}H NMR** (500 MHz, $CDCl_3$) δ 7.71 (s, 1H), 7.30 – 7.24 (m, 3H), 7.23 – 7.18 (m, 1H), 4.34 (q, J = 7.1 Hz, 2H), 4.30 (q, J = 7.1 Hz, 2H), 2.35 (s, 3H), 1.33 (t, J = 7.1 Hz, 3H), 1.30 (t, J = 7.1 Hz, 3H). **^{13}C NMR** (126 MHz, $CDCl_3$) δ 166.9, 164.3, 142.5, 138.6, 132.9, 131.5, 130.3, 128.8, 126.7, 126.1, 61.8, 61.8, 21.5, 14.3, 14.0.

Diethyl 2-(pyridin-3-ylmethylene)malonate

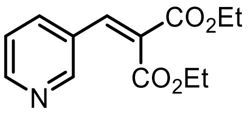

The title compound was synthesised by general procedure A stirring overnight and purified by kugelrohr distillation (114 ˚C, <1 mbar) (54%, 0.682 g) as an orange oil. **^{1}H NMR** (500 MHz, $CDCl_3$) δ 8.68 (d, *J* = 2.3 Hz, 1H), 8.61 (dd, *J* = 4.8, 1.6 Hz, 1H), 7.77 (dddd, *J* = 8.0, 2.3, 1.6, 0.6 Hz, 1H), 7.70 (s, 1H), 7.31 (dddd, *J* = 8.0, 4.8, 0.8, 0.4 Hz, 1H), 4.34 (q, *J* = 7.1 Hz, 1H), 4.32 (q, *J* = 7.1 Hz, 1H), 1.34 (t, *J* = 7.1 Hz, 1H), 1.29 (t, *J* = 7.1 Hz, 1H). **^{13}C NMR** (126 MHz, $CDCl_3$) δ 166.1, 163.7, 151.2, 150.8, 138.6, 135.8, 129.1, 128.7, 123.7, 62.1, 62.1, 14.3, 14.1. **HRMS** (ES+) [$C_{13}H_{16}NO_4$] calc. 250.1079 found 250.1089. Characterization data is in accordance with prior reports.[9]

Diethyl 2-(naphthalen-2-ylmethylene)malonate

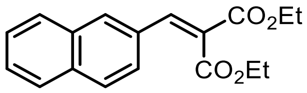

The title compound was synthesised by general procedure A stirring for 2 days and purified by kugelrohr distillation (132 ˚C, <1 mbar) (59%, 0.8923 g) as a colourless oil. **^{1}H NMR** (500 MHz, $CDCl_3$) δ 7.99 – 7.95 (m, 1H), 7.90 (s, 1H), 7.83 (ddd, *J* = 8.7, 6.6, 2.9 Hz, 1H), 7.58 – 7.45 (m, 1H), 4.38 (q, *J* = 7.2 Hz, 1H), 4.34 (q, *J* = 7.2 Hz, 1H), 1.36 (t, *J* = 7.1 Hz, 1H), 1.30 (t, *J* = 7.1 Hz, 1H). **^{13}C NMR** (126 MHz, $CDCl_3$) δ 167.0, 164.4, 142.3, 134.2, 133.2, 131.1, 130.6, 128.9, 128.7, 127.9, 127.8, 126.9, 126.5, 125.4, 61.9, 61.8, 14.3, 14.1. **HRMS** (ES+) [$C_{18}H_{18}O_4 + Na^+$] calc. 321.1103 found 321.1103

Diethyl 2-(naphthalen-1-ylmethylene)malonate

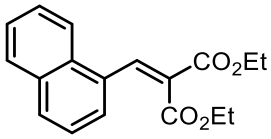

The title compound was synthesised by general procedure A stirring for 2 days and purified by kugelrohr distillation (138 ˚C, <1 mbar) (67%, 1.012 g) as an orange oil. **^{1}H NMR** (500 MHz, $CDCl_3$) δ 8.47 (s, 1H), 8.00 (d, *J* = 8.1 Hz, 1H), 7.90 – 7.84 (m, 2H), 7.60 – 7.51 (m, 3H), 7.46 – 7.40 (m, 1H), 4.37 (q, *J* = 7.1 Hz, 2H), 4.16 (q, *J* = 7.1 Hz, 2H), 1.38 (t, *J* = 7.1 Hz, 3H), 1.06 (t, *J* = 7.1 Hz, 3H). **^{13}C NMR** (126 MHz, $CDCl_3$) δ 166.3, 164.1, 141.4, 133.5, 131.5, 131.0, 130.6, 129.4, 128.8, 127.1, 126.6, 126.5, 125.3, 124.2, 61.9, 61.6, 14.3, 13.9. **HRMS** (ES+) [$C_{18}H_{18}O_4 + Na^+$] calc. 321.1103 found 321.1105. Characterization data is in accordance with prior reports.[4]

Diethyl 2-(cyclopropylmethylene)malonate

[9] M. M. Ramaiah, N. S. Shivananju, P. B. Shubha, *Lett. Org. Chem.* **2020**, 17, DOI : 10.2174/1570178616666190401194641

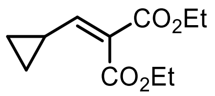

The title compound was synthesised by general procedure A stirring for 2 days and purified by kugelrohr distillation (75 ˚C, <1 mbar) (73%, 1.55 g) as a colourless oil. **^{1}H NMR** (500 MHz, $CDCl_3$) δ 6.35 (d, *J* = 11.3 Hz, 1H), 4.31 (q, *J* = 7.1 Hz, 1H), 4.21 (q, *J* = 7.1 Hz, 1H), 1.96 (tdt, *J* = 12.4, 7.9, 4.5 Hz, 1H), 1.33 (t, *J* = 7.1 Hz, 2H), 1.27 (t, *J* = 7.1 Hz, 2H), 1.08 (qd, *J* = 4.9, 0.7 Hz, 1H), 0.75 (tt, *J* = 5.0, 2.5 Hz, 1H). **^{13}C NMR** (126 MHz, $CDCl_3$) δ 166.0, 164.5, 156.2, 125.7, 61.3, 61.2, 14.3, 14.3, 13.1, 10.0. **HRMS** (AS+) [$C_{11}H_{16}O_4$ + H] calc. 213.127 found 213.1120. Characterization data is in accordance with prior reports.[9]

Diethyl 2-(2,6-dimethylhept-5-en-1-ylidene)malonate

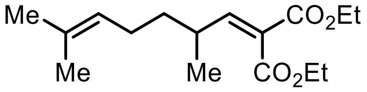

The title compound was synthesised by general procedure A stirring for 2 days and purified column chromatography (77%, 1.04 g) as a colourless oil. **^{1}H NMR** (500 MHz, $CDCl_3$) δ 6.75 (d, *J* = 10.8 Hz, 1H), 5.03 (t, *J* = 7.0 Hz, 1H), 4.27 (qd, *J* = 7.1, 3.3 Hz, 2H), 4.22 (q, *J* = 7.1 Hz, 2H), 2.55 (ddd, *J* = 13.6, 11.0, 6.8 Hz, 1H), 1.94 (d, *J* = 7.5 Hz, 1H), 1.91 (d, *J* = 7.4 Hz, 1H), 1.65 (s, 3H), 1.56 (s, 3H), 1.44 – 1.34 (m, 2H), 1.29 (dt, *J* = 15.3, 7.1 Hz, 6H), 1.04 (d, *J* = 6.6 Hz, 3H). **^{13}C NMR** (126 MHz, $CDCl_3$) δ 165.8, 164.2, 154.3, 132.1, 127.5, 123.9, 61.3, 61.2, 36.7, 34.5, 26.0, 25.8, 19.9, 17.8, 14.3, 14.2. **HRMS** (AP+) [$C_{16}H_{27}O_4$] calc. 283.1909 found 283.1914

Dimethyl 2-(4-fluorobenzylidene)malonate

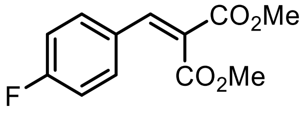

The title compound was synthesised by general procedure A stirring for 2 days and purified by kugelrohr distillation (90 ˚C, <1 mbar) (65%, 0.771 g) as a colourless oil. **^{1}H NMR** (400 MHz, $CDCl_3$) δ 7.73 (s, 1H), 7.43 (dd, *J* = 8.5, 5.3 Hz, 2H), 7.08 (t, *J* = 8.6 Hz, 2H), 3.85 (s, 3H), 3.85 (s, 3H). **^{13}C NMR** (101 MHz, $CDCl_3$) δ 167.2 (s), 164.6 (s), 164.1 (d, *J* = 253.0 Hz), 141.8 (s), 131.7 (d, *J* = 8.7 Hz), 129.2 (d, *J* = 3.4 Hz), 125.4 (s), 116.3 (d, *J* = 21.9 Hz), 52.9 (s), 52.9 (s). **^{19}F NMR** (376 MHz, $CDCl_3$) δ -108.3. Characterization data is in accordance with prior reports.[10]

[10] Y. Matsumoto, D. Nakatake, R. Yazaki, T. Ohshima, *Chem. Eur. J.* **2018**, 24, 6062-6066

Diisopropyl 2-(4-fluorobenzylidene)malonate

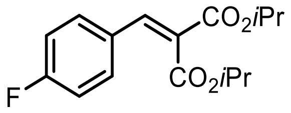

The title compound was synthesised by general procedure A stirring for 2 days and purified by kugelrohr distillation (98 ˚C, <1 mbar) (57%, 0.841 g) as an orange oil. ^{1}**H NMR** (400 MHz, $CDCl_3$) 7.63 (s, 3H), 7.47 (dd, *J* = 8.5, 5.3 Hz, 6H), 7.06 (t, *J* = 8.6 Hz, 6H), 5.31 – 5.20 (m, 3H), 5.20 – 5.09 (m, 3H), 1.31 (d, *J* = 6.4 Hz, 18H), 1.29 (d, *J* = 6.5 Hz, 19H).^{13}**C NMR** (101 MHz, $CDCl_3$) δ 166.3 (s), 164.0 (d, *J* = 252.3 Hz), 163.8 (s), 140.2 (s), 131.7 (d, *J* = 8.6 Hz), 129.4 (d, *J* = 3.3 Hz), 127.0 (s), 116.1 (d, *J* = 21.8 Hz), 69.5 (s), 21.9 (s), 21.7 (s). ^{19}**F NMR** (376 MHz, $CDCl_3$) δ -108.9.

Di-tert-butyl 2-(4-fluorobenzylidene)malonate

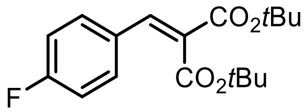

The title compound was synthesised by general procedure A stirring for 2 days and purified by kugelrohr distillation (105 ˚C, <1 mbar) (42%, 0.676 g) as a yellow oil. ^{1}**H NMR** (500 MHz, $CDCl_3$) δ 7.53 – 7.47 (m, 3H), 7.08 – 7.01 (m, 2H), 1.53 (s, 9H), 1.52 (s, 9H). ^{13}**C NMR** (126 MHz, $CDCl_3$) δ 166.0 (s), 163.8 (d, *J* = 251.6 Hz), 163.6 (s), 138.7 (s), 131.6 (d, *J* = 8.5 Hz), 129.7 (d, *J* = 3.4 Hz), 129.1 (d, *J* = 2.0 Hz), 115.9 (d, *J* = 21.8 Hz), 82.6 (s), 82.3 (s), 28.2 (s), 28.0 (s). ^{19}**F NMR** (376 MHz, $CDCl_3$) δ -109.6.

Diallyl 2-(4-fluorobenzylidene)malonate

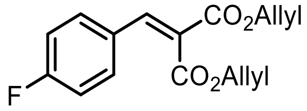

The title compound was synthesised by general procedure A stirring for 2 days and purified by kugelrohr distillation (95 ˚C, <1 mbar) (65%, 0.944 g) as a colourless oil. ^{1}**H NMR** (400 MHz, $CDCl_3$) δ 7.73 (s, 1H), 7.46 (dd, *J* = 8.5, 5.3 Hz, 2H), 7.07 (t, *J* = 8.6 Hz, 2H), 5.94 (tddd, *J* = 16.3, 11.9, 10.4, 5.7 Hz, 2H), 5.42 – 5.31 (m, 2H), 5.31 – 5.22 (m, 2H), 4.76 (ddt, *J* = 7.9, 5.6, 1.4 Hz, 4H). ^{13}**C NMR** (101 MHz, $CDCl_3$) δ 166.2 (s), 164.1 (d, *J* = 252.9 Hz), 163.8 (s), 141.8 (s), 131.9 (d, *J* = 8.7 Hz), 131.7 (s), 131.3 (s), 129.1 (d, *J* = 3.4 Hz), 125.6 (d, *J* = 2.0 Hz), 119.6 (s), 118.7 (s), 116.2 (d, *J* = 21.9 Hz), 66.5 (s), 66.3 (s). ^{19}**F NMR** (376 MHz, $CDCl_3$) δ -108.3.

Dibenzyl 2-(4-fluorobenzylidene)malonate

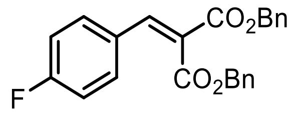

The title compound was synthesised by general procedure A stirring for 2 days and purified by kugelrohr distillation (136 ˚C, <1 mbar) (70%, 1.37 g) as a yellow oil. **^{1}H NMR** (400 MHz, $CDCl_3$) δ 7.63 (s, 1H), 7.39 – 7.11 (m, 13H), 6.81 (t, *J* = 8.6 Hz, 2H), 5.20 (s, 2H), 5.18 (s, 2H). **^{13}C NMR** (101 MHz, $CDCl_3$) δ 166.3 (s), 164.0 (d, *J* = 252.8 Hz), 163.9 (s), 142.0 (s), 135.6 (s), 134.9 (s), 131.8 (d, *J* = 8.7 Hz), 129.1 (s), 129.0 (d, *J* = 3.2 Hz), 128.7 (s), 128.5 (s), 128.2 (s), 125.5 (s), 125.5 (s, *J* = 2.0 Hz), 116.1 (d, *J* = 21.9 Hz), 67.8 (s), 67.4 (s). **^{19}F NMR** (376 MHz, $CDCl_3$) δ -108.3.

Ethyl (E)-2-cyano-3-(4-fluorophenyl)acrylate

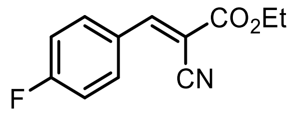

The title compound was synthesised by general procedure A stirring for 1 days and purified by recrystallization in ethanol (90%, 0.996 g) as a yellow solid. **MP** 94 - 97 ˚C. **^{1}H NMR** (500 MHz, $CDCl_3$) δ 8.21 (s, 1H), 8.07 – 7.99 (m, 1H), 7.22 – 7.16 (m, 1H), 4.39 (q, *J* = 7.1 Hz, 1H), 1.40 (t, *J* = 7.1 Hz, 2H). **^{13}C NMR** (126 MHz, $CDCl_3$) δ 165.6 (d, *J* = 257.6 Hz), 162.6 (s), 153.7 (s), 133.7 (d, *J* = 9.2 Hz), 128.0 (d, *J* = 3.3 Hz), 116.9 (d, *J* = 22.1 Hz), 115.6 (s), 102.7 (d, *J* = 2.4 Hz), 63.0 (s), 14.3 (s). **^{19}F NMR** (376 MHz, None) δ -104.8.

5-(4-Fluorobenzylidene)-1,3-dimethylpyrimidine-2,4,6(1H,3H,5H)-trione

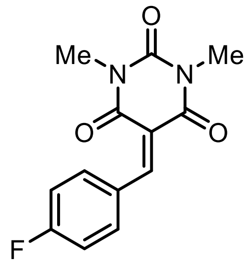

The title compound was synthesised by general procedure A stirring for 1 days and purified by recrystallization in ethanol (95%, 1.245 g) as a yellow solid. **MP** 161 - 164 ˚C. **^{1}H NMR** (500 MHz, $CDCl_3$) δ 8.52 (s, 1H), 8.23 – 8.14 (m, 2H), 7.20 – 7.11 (m, 2H), 3.42 (s, 3H), 3.38 (s, 3H). **^{13}C NMR** (126 MHz, $CDCl_3$) δ 165.6 (d, *J* = 257.7 Hz), 162.7 (s), 160.7 (s), 158.0 (s), 151.3 (s), 136.9 (d, *J* = 9.3 Hz), 129.0 (d, *J* = 3.1 Hz), 117.1 (d, *J* = 1.7 Hz), 115.8 (d, *J* = 21.8

Hz), 29.3 (s), 28.6 (s). **^{19}F NMR** (471 MHz, $CDCl_3$) δ -103.3. Characterization data is in accordance with prior reports.[11]

5-(4-Fluorobenzylidene)-2,2-dimethyl-1,3-dioxane-4,6-dione

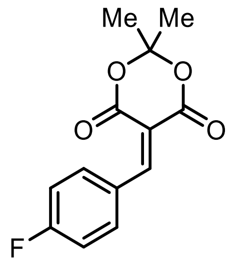

The title compound was synthesised by general procedure A stirring for 2 days and purified by recrystallization from diethyl ether and petroleum ether (98%, 1.225 g) as a white solid. **MP** 135 - 137 ˚C. **^{1}H NMR** (500 MHz, $CDCl_3$) δ 8.39 (s, 1H), 8.17 (dd, *J* = 8.1, 5.7 Hz, 2H), 7.17 (t, *J* = 8.3 Hz, 2H), 1.80 (s, 6H). **^{13}C NMR** (126 MHz, $CDCl_3$) δ 165.8 (d, *J* = 258.9 Hz), 163.3 (s), 159.9 (s), 156.7 (s), 136.9 (d, *J* = 9.5 Hz), 128.1 (d, *J* = 3.3 Hz), 116.2 (d, *J* = 21.9 Hz), 114.2 (d, *J* = 1.8 Hz), 104.6 (s), 27.6 (s). **^{19}F NMR** (376 MHz, $CDCl_3$) δ -101.9.

Ethyl 2-oxo-2H-chromene-3-carboxylate

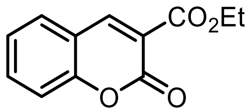

The title compound was prepared using a method modified from the literature.[12] To a flame dried 25 mL microwave vial was added salicylaldehyde (1.2 g, 10 mmol) and diethylmalonate (2.2 g, 14 mmol), the vial was then sealed and put under nitrogen. Ethanol (5 mL ,2 M) that had been dried over 3 Å molecular sieves was then added followed by piperidine (0.125 mL, 1 mmol) and acetic acid (0.050 mL 1 mmol). The vial was then heated to 125 ˚C for 3 hours. After which the reaction was cooled to 0 ˚C and water (10 mL) was added. This mixture was then filtered and washed with water ethanol (1:1) solution. The remaining solid was recrystalized from the minimum amount of boiling water ethanol (3:1). This gave the title compound as a white solid (82%, 1.89 g). **MP** 61 - 65 ˚C. **^{1}H NMR** (500 MHz, $CDCl_3$) δ 8.52 (s, 1H), 7.68 – 7.58 (m, 2H), 7.39 – 7.30 (m, 2H), 4.42 (q, *J* = 7.1 Hz, 2H), 1.41 (t, *J* = 7.1 Hz,

---

[11] E. Fillion, A. Kavoosi, K. Nguyen, C. Ieritano, *Chem. Commun.* **2016**, 52, 12813-12816

[12] Z. Zhang, Z.-W. Bai, Y. Ling, L.-Q. He, P. Huang, H.-X. Gu, R.-F. Hu, *Med. Chem. Res.* **2018**, 27, 1198-1205

3H). **^{13}C NMR** (126 MHz, $CDCl_3$) δ 163.2, 156.9, 155.3, 148.7, 134.5, 129.6, 125.0, 118.5, 118.0, 117.0, 62.1, 14.4. **HRMS** (ES+) [$C_{12}H_{10}O_4$ + Na^+] calc. 241.477 found 241.079

2-benzylidenemalononitrile

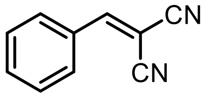

Following a modified literature procedure[13], benzaldehyde (1.061 g, 10 mmol), malononitrile (0.661 g, 10 mmol) and piperidine (0.099 mL, 1 mmol) were dissolved in ethanol (10 mL). The mixture was stirred at room temperature overnight. The solvent was removed under reduced pressure, dichloromethane (50 mL) and HCl (0.1 M, 50mL) were added and the mixture transferred to a separating funnel. The layers were separated and the aqueous layer further extracted with dichloromethane (2 x 50 mL). The combined organic phase was dried ($MgSO_4$), filtered and the solvent removed under reduced pressure. The residue was then recrystallized from hot EtOH/H_2O to yield the product as yellow crystals (0.941 g, 6.11 mmol, 61%). **MP** 80 - 81 °C. **^{1}H NMR** (500 MHz; $CDCl_3$): δ 7.91 (d, J = 7.8 Hz, 2H), 7.78 (s, 1H), 7.65-7.62 (m, 1H), 7.55 (t, J = 7.6 Hz, 2H). **^{13}C NMR** (126 MHz; $CDCl_3$): δ 160.0, 134.8, 131.1, 130.9, 129.8, 113.8, 112.7, 83.1.

Ethyl 3-phenylpropiolate

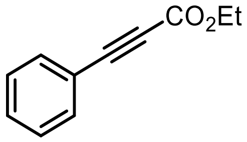

Following a modified literature procedure[14]. To an oven-dried flask was added potassium carbonate (2.073 g, 15 mmol) and the flask flushed with nitrogen. Ethyl propiolate (0.507 mL, 5 mmol), iodobenzene (0.839 mL, 7.5 mmol) and dry THF (40 mL) were added and stirred. To this mixture was added $Pd(PPh_3)_2Cl_2$ (0.070 g, 0.1 mmol) and CuI (0.018 g, 0.1 mmol) and the flask flushed with nitrogen. The mixture was heated to reflux, stirred for 22 hours, cooled and filtered through cotton wool into a separating funnel. Dichloromethane (50 mL) and water (50 mL) were added, the layers separated and the aqueous layer further extracted with dichloromethane (2 x 50 mL). The combined organic phase was washed (brine), dried ($MgSO_4$), filtered and the solvent removed under reduced pressure. The product was purified by flash column chromatography with gradient elution (0 - 10% ethyl acetate in petroleum

[13] J. R. Xin, J. T. Guo, D. Vigliaturo, Y. H. He and Z. Guan, *Tetrahedron*, 2017, **73**, 4627–4633.

[14] J. Li, J. Zhang, H. Tan and D. Z. Wang, *Org. Lett.*, 2015, **17**, 2522–2525

ether) and the product obtained as a yellow oil (0.325 g, 1.87 mmol, 37%). ^{1}**H NMR** (500 MHz; $CDCl_3$): δ 7.59 (d, *J* = 8.0 Hz, 2H), 7.46-7.43 (m, 1H), 7.37 (t, *J* = 7.6 Hz, 2H), 4.32-4.28 (m, 2H), 1.36 (t, *J* = 7.1 Hz, 3H). ^{13}**C NMR** (126 MHz; $CDCl_3$): δ 154.2, 133.1, 130.7, 128.7, 119.8, 86.2, 80.8, 62.3, 14.3.

Diethyl 2-(4-fluorobenzyl)malonate

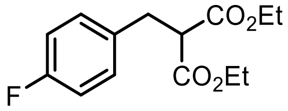

The title compound was prepared using a method modified from the literature.[15] To a flame dried 10 mL microwave vial was added sodium hydride (60% suspension in mineral oil) (42 mg, 1.05 mmol) and the vial placed under nitrogen. Dry THF (5 mL, 0.2 M) was then added and the vial was cooled to 0 ˚C. Diethyl malonate (0.160 mL, 1.05 mmol) was then added dropwise and the mixture was stirred for 15 mins followed by the addition of 4-fluorobenzyl bromide (0.125 mL, 1 mmol). The vial was then heated to 75 ˚C for 1 hour. Water (2 mL) was then added and the solvent was removed *en vacuo* and the remaining oil was dissolved in diethyl ether 20 mL and washed with water (20 mL). The water layer was then extracted twice more with diethyl ether and the combined organic phases were dried over magnesium sulphate. The solvent was then removed *en vacuo* this oil was then purified using flash column chromatography (hexane:ethyl acetate). The title compound was isolated as a colourless oil (52.6 mg, 20%). ^{1}**H NMR** (500 MHz, $CDCl_3$) δ 7.17 (dd, *J* = 8.3, 5.4 Hz, 1H), 6.96 (t, *J* = 8.7 Hz, 1H), 4.25 – 4.06 (m, 2H), 3.60 (td, *J* = 7.9, 1.1 Hz, 1H), 3.18 (d, *J* = 7.9 Hz, 1H), 1.30 – 1.11 (m, 3H). ^{13}**C NMR** (126 MHz, $CDCl_3$) δ 168.9 (s), 161.9 (d, *J* = 244.8 Hz), 133.7 (d, *J* = 3.3 Hz), 130.5 (d, *J* = 8.0 Hz), 115.5 (d, *J* = 21.3 Hz), 61.7 (s), 54.1 (s), 54.1 (s), 34.0 (s), 14.2 (s). ^{19}**F NMR** (471 MHz, $CDCl_3$) δ -116.3. **HRMS** (ES+) [$C_{14}H_{17}O_4F + Na^+$] calc. 291.1009 found 291.1016.

Methyl 4-formylbenzoate

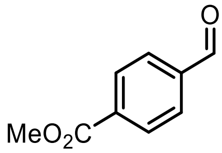

The title compound was prepared using the following procedure. To an oven dried 100 mL round bottom flask that had been placed under nitrogen was added, 4-carboxybenzaldehyde (2.25 g, 15 mmol), imidazole (2.04 g, 30 mmol), DMAP (0.366 g, 3 mmol), CH_2Cl_2 (30 mL, 0.5

[15] S. Herold, D. Bafaluy, K. Muñiz, *Green Chem.* **2018**, 20, 3191-3196

M) and methanol (2 mL, 45 mmol). This solution was then cooled to 0 ˚C. A solution containing DCC (1 M, 17 mL) in CH_2Cl_2 was prepared in a dried flask. This DCC solution was then added dropwise to the cooled reaction flask after which it was left to increase to room temperature and continued to stir for a further 3 hours. The reaction mixture was then filtered, and the filtrate collected. The filtrate was then washed with 0.5 M HCl (30 mL), saturated $NaHCO_3$ (30 mL), and brine (30 mL). The organic phase was then dried over magnesium sulfate and solvent removed *en vacuo*. This gave the title compound as a white solid and was used without further purification (quant., 2.62 g). **MP** 50 - 52 ˚C. **^{1}H NMR** 10.10 (s, 1H), 8.20 (d, *J* = 7.6 Hz, 2H), 7.95 (d, *J* = 7.6 Hz, 2H), 3.96 (s, 3H). **^{13}C NMR** (126 MHz, $CDCl_3$) δ 191.8, 166.2, 139.3, 135.2, 130.3, 129.7, 52.7.

General Procedure B: Synthesis of malonates

The title compounds were prepared using a method modified from the literature.[16] To a dried 100 mL round bottom flask was added diethyl ether (25 mL), malonic acid (10 mmol), and desired alcohol coupling partner (30 mmol). This mixture was cooled to 0 ˚C and a solution of DCC in diethyl ether (1 M, 30 mL) was added slowly. Once addition was complete the reaction mixture was allowed to rise to room temperature and stirred for a further 1 hour. Once the reaction was complete the mixture was filtered and the filtrate collected. The filtrate was then washed with saturated sodium bicarbonate followed by brine and dried using magnesium sulphate. The solvent was removed *en vacuo* and the title compound was used without further purification or purified by flash column chromatography.

Di-*iso*-propyl malonate

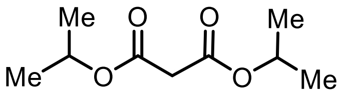

The title compound was prepared by general procedure B (93%, 1.72 g) as a colourless oil. **^{1}H NMR** (500 MHz, $CDCl_3$) δ 5.05 (hept, *J* = 6.3 Hz, 1H), 3.29 (s, 1H), 1.25 (d, *J* = 6.3 Hz, 6H). **^{13}C NMR** (126 MHz, $CDCl_3$) δ 166.4, 69.1, 42.5, 21.8.

Di-*tert*-butyl malonate

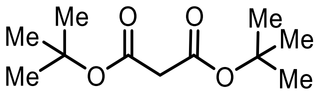

[16] R. Shelkov, M. Nahmany, A. Melman, *J. Org. Chem.* **2002**, 67, 8975-8982

The title compound was prepared by general procedure B exchanging diethyl ether as solvent for acetonitrile (97%, 2.10 g) as a yellow oil. **^{1}H NMR** (500 MHz, $CDCl_3$) δ 3.18 (s, 2H), 1.47 (s, 18H). **^{13}C NMR** (126 MHz, $CDCl_3$) δ 166.3, 81.8, 44.5, 28.1.

Diallyl malonate

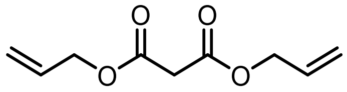

The title compound was prepared by general procedure B (93%, 1.76 g) as a colourless oil. **^{1}H NMR** (500 MHz, $CDCl_3$) δ 5.91 (ddt, *J* = 17.2, 10.4, 5.8 Hz, 1H), 5.34 (ddd, *J* = 17.2, 3.0, 1.5 Hz, 1H), 5.26 (ddd, *J* = 10.4, 1.3 Hz, 1H), 4.65 (ddd, 2H), 3.44 (s, 1H). **^{13}C NMR** (126 MHz, $CDCl_3$) δ 166.2, 131.6, 119.0, 66.2, 41.6.

Product Characterisation

General Procedure C: Reductive Dimerization of Alkenes

To a 14 mL stainless steel milling jar containing one milling ball (10 mm, 4.1 g) was added manganese, irregular pieces, (1.1 mmol, 60 mg), the appropriate arylidene malonate (1 mmol), THF (2 mmol, 160 µL), and lithium chloride (1 mmol, 42 mg). The reaction mixture was then milled at 30 Hz for 3 h. The mixture was then washed into a flask with EtOAc, HCl (1 M, 25 mL) was added and the mixture stirred for ten minutes and the organic phase was separated, washed with brine and dried with magnesium sulphate. This crude mixture was then purified using flash column chromatography. If the diastereomers of the product could not be separated the columned material was then further purified using recycling preparative HPLC.

General Procedure D: Reductive Dimerization of Alkenes in solution

If required, manganese of the appropriate form (275 mg) was placed in a 14 mL stainless steel milling jar containing one milling ball (10 mm, 4.1 g) and milled at 30 Hz for 5 mins.

To a 5 mL vial containing a magnetic stirrer bar was added manganese, (0.55 mmol, 30 mg) diethyl 2-(4-fluorobenzylidene)malonate (0.5 mmol, 133 mg), lithium chloride (0.5 mmol, 21 mg), and THF (2 mL, 0.25 M). The reaction mixture was left to stir and upon completion trifluorotoluene (0.167 mmol, 21 µL) was added as an internal standard followed by aq. HCl (1.5 mL, 1 M) and EtOAc (1.5 mL). The biphasic mixture was left to stir until the remaining manganese had been quenched and effervescing had ceased. The organic phase was then analysed by ^{19}F NMR spectroscopy.

Tetraethyl 2,3-bis(4-fluorophenyl)butane-1,1,4,4-tetracarboxylate (7)

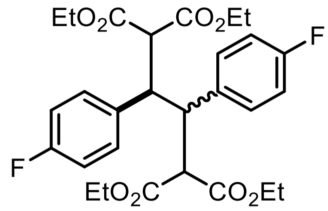

The title compound was prepared by general procedure C as a mixture of diastereomers (88%, 3:2, 235 mg) as a white solid. The anti diastereomer was separated by column chromatography and the syn diastereomer was separated by recycling preparative HPLC using 80:20 hexane:EtOAc. **MP** 144 - 145 ˚C. **Anti ^{1}H NMR** (500 MHz, $CDCl_3$) δ 7.32 – 7.27 (m, 1H), 6.96 (t, *J* = 8.6 Hz, 1H), 4.07 (dd, *J* = 5.6, 2.0 Hz, 1H), 3.95 (ddq, *J* = 14.6, 7.4, 3.6 Hz, 1H), 3.86 (q, *J* = 7.1 Hz, 1H), 3.58 (dd, *J* = 5.6, 2.1 Hz, 1H), 1.11 (t, *J* = 7.1 Hz, 1H), 0.97 (t, *J* = 7.1 Hz, 2H). **Anti ^{13}C NMR** (126 MHz, $CDCl_3$) δ 168.3 (s), 167.7 (s), 162.3 (d, *J* = 246.6 Hz), 133.8 (d, *J* = 3.3 Hz), 131.9 (d, *J* = 7.9 Hz), 115.1 (d, *J* = 21.2 Hz), 61.7 (s), 61.4 (s), 55.5 (s), 47.9 (s), 14.0 (s), 13.8 (s). **Anti ^{19}F NMR** (376 MHz, $CDCl_3$) δ -114.4. **IR**: ν_{max}: 1749, 1722, 1219, 1157, 1106 cm^{-1} **Anti HRMS** (AP+) [$C_{28}H_{33}F_2O_8$] calc. 535.2143 found 535.2144

Syn ^{1}H NMR (400 MHz, $CDCl_3$) δ 6.88 (br s, 4H), 4.49 – 4.29 (m, 2H), 3.90 – 3.72 (m, 3H), 3.65 (q, *J* = 1.7 Hz, 1H), 1.41 (t, *J* = 7.1 Hz, 3H), 0.85 (t, *J* = 7.1 Hz, 3H). **Syn ^{13}C NMR** (126 MHz, $CDCl_3$) δ 167.9 (s), 167.1 (s), 162.3 (d, *J* = 246.7 Hz), 131.6 (d, *J* = 3.3 Hz), 114.8 (d, *J* = 18.7 Hz), 62.3 (s), 61.5 (s), 55.7 (s), 45.9 (s), 14.3 (s), 13.7 (s). **Syn ^{19}F NMR** (376 MHz, $CDCl_3$) δ -114.6 (s). **Syn HRMS** (ES+) [$C_{28}H_{32}O_8F_2 + Na^+$] calc. 557.1963 found 557.1943

Tetraethyl 2,3-diphenylbutane-1,1,4,4-tetracarboxylate (4)

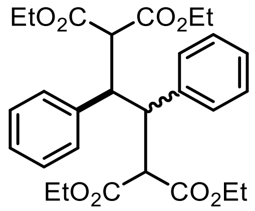

The title compound was prepared by general procedure C as a mixture of diastereomers (56%, 3:5, 140 mg) as a white solid. The diastereomers were separated by preparatory TLC, 5 runs at 80:20 hexane:EtOAc. **MP** 89 - 90 ˚C. **Anti ^{1}H NMR** (500 MHz, $CDCl_3$) δ 7.37 – 7.07 (m, 16H), 4.09 (dd, *J* = 6.0, 2.2 Hz, 3H), 3.98 – 3.85 (m, 6H), 3.81 (qd, *J* = 7.1, 1.0 Hz, 6H), 3.67 (dd, *J* = 6.0, 2.2 Hz, 3H), 1.10 (t, *J* = 7.1 Hz, 9H), 0.91 (t, *J* = 7.1 Hz, 9H). **Anti ^{13}C NMR** (500 MHz, $CDCl_3$) δ 7.4 – 7.1 (m, 16H), 4.1 (dd, *J* = 6.0, 2.2 Hz, 3H), 4.0 – 3.9 (m, 6H), 3.8 (qd, *J* = 7.1, 1.0 Hz, 6H), 3.7 (dd, *J* = 6.0, 2.2 Hz, 3H), 1.1 (t, *J* = 7.1 Hz, 9H), 0.9 (t, *J* = 7.1 Hz, 9H). **IR**: ν_{max}: 1759, 1736, 1203, 1165 cm^{-1} **Anti HRMS** (AP+) [$C_{28}H_{35}O_8$] calc. 499.2332 found 499.2309

Syn ^{1}H NMR (500 MHz, $CDCl_3$) δ 9.94 (s, enol 1H), 7.79 (d, *J* = 8.0 Hz, enol 2H), 7.48 (d, *J* = 8.1 Hz, enol 2H), 7.35 – 7.27 (m, 7H), 7.19 (m, 7H), 4.84 (d, *J* = 12.1 Hz, enol 1H), 4.50 – 4.31 (m, 4H), 4.02 (m, 2H), 3.91 (s, 1H), 3.88 (s, 1H), 3.84 – 3.69 (m, 6H), 1.42 (t, *J* = 7.1 Hz, 6H), 1.04 (t, *J* = 7.1 Hz, enol 6H), 1.02 (t, *J* = 7.1 Hz, enol 6H), 0.79 (t, *J* = 7.1 Hz, 6H). **Syn ^{13}C NMR** (126 MHz, $CDCl_3$) δ 191.8 (enol), 168.1, 167.5 (enol), 167.4 (enol), 167.4, 148.6 (enol), 140.3 (enol), 136.0, 135.3 (enol), 130.3, 129.0, 128.6, 128.0, 127.7, 127.7, 127.5, 127.5 (enol), 62.2, 61.9 (enol), 61.8 (enol), 61.3, 57.2 (enol), 55.7, 51.3 (enol), 46.6, 14.3, 14.0 (enol), 13.9 (enol), 13.6. **Syn HRMS** (AP+) [$C_{28}H_{35}O_8$] calc. 499.2332 found 499.2322

Single crystal X-Ray diffraction structure of anti:

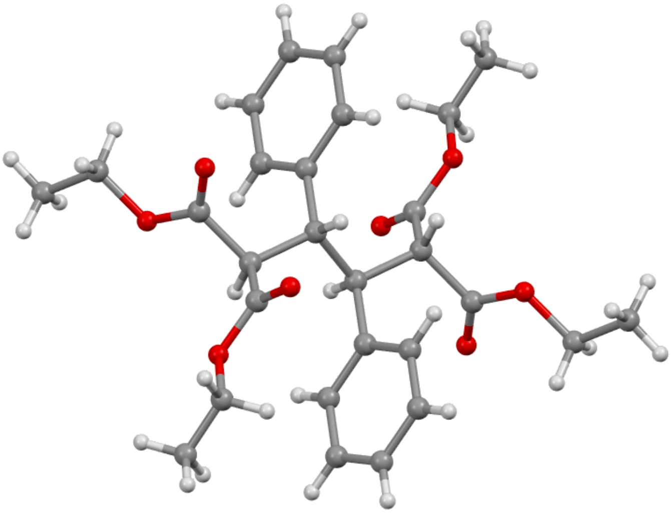

Tetraethyl (2,3-bis(4-(trifluoromethyl)phenyl)butane-1,1,4,4-tetracarboxylate (8)

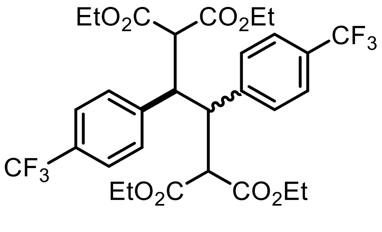

The title compound was prepared by general procedure C as a mixture of diastereomers (58%, 6:5, 368 mg) as a white solid. The diastereomers were separated by column chromatography. **MP** 186 – 188 ˚C. **Anti ^{1}H NMR** (500 MHz, $CDCl_3$) δ 7.55 (d, *J* = 8.1 Hz, 2H), 7.44 (d, *J* = 8.1 Hz, 2H), 4.20 (dd, *J* = 5.6, 2.2 Hz, 1H), 3.99 – 3.90 (m, 2H), 3.86 (qd, *J* = 7.1, 2.8 Hz, 2H), 3.60 (dd, *J* = 5.6, 2.2 Hz, 1H), 1.10 (t, *J* = 7.1 Hz, 3H), 0.94 (t, *J* = 7.1 Hz, 3H). **Anti ^{13}C NMR** (126 MHz, $CDCl_3$) δ 167.9 (s), 167.2 (s), 130.5 (s), 130.0 (q, *J* = 32.5 Hz), 128.6 (s), 127.8 (q), 125.1 (q, *J* = 3.6 Hz), 61.8 (s), 61.4 (s), 55.1 (s), 47.9 (s), 13.8 (s), 13.6 (s). **Anti ^{19}F NMR** (471

MHz, $CDCl_3$) δ -62.7. **IR**: ν_{max}: 2970, 1740, 1716, 1325, 1126 cm^{-1} **Anti HRMS** (ES+) [$C_{28}H_{35}O_8$] calc. 499.2332 found 499.2309

MP 100 – 102 ˚C. **Syn ^{1}H NMR** (500 MHz, $CDCl_3$) δ 7.61 – 7.35 (br, m, 1H), 6.83 (br, s, 1H), 4.51 – 4.34 (m, 1H), 3.99 (q, *J* = 1.9 Hz, 1H), 3.89 – 3.74 (m, 1H), 3.70 (q, *J* = 1.8 Hz, 1H), 1.42 (t, *J* = 7.1 Hz, 1H), 0.84 (t, *J* = 7.1 Hz, 1H). **Syn ^{13}C NMR** (126 MHz, $CDCl_3$) δ 167.6 (s), 166.8 (s), 140.1 (s), 130.7 (s), 130.2 (q, *J* = 32.6 Hz), 124.9 (s), 124.1 (q, *J* = 272.1 Hz), 62.6 (s), 61.7 (s), 55.3 (s), 46.3 (s), 14.3 (s), 13.6 (s). **Syn ^{19}F NMR** (471 MHz, $CDCl_3$) δ -62.6. **IR**: ν_{max}: 2980, 1749, 1722, 1323, 1118, 1068 cm^{-1} **Syn HRMS** (ES+) [$C_{30}H_{32}O_8F_6$ + Na^+] calc. 657.1899 found 657.1899

Tetraethyl 2,3-di-p-tolylbutane-1,1,4,4-tetracarboxylate (9)

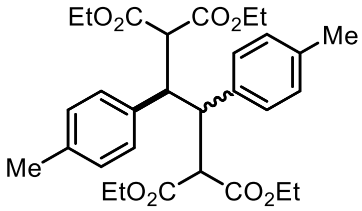

The title compound was prepared by general procedure C as a mixture of diastereomers (85%, 3:2, 223 mg) as a white solid. The diastereomers were separated by column chromatography. **MP** 92 – 94 ˚C. **Anti ^{1}H NMR** (500 MHz, $CDCl_3$) δ 7.17 (d, *J* = 8.1 Hz, 1H), 7.08 – 7.03 (m, 1H), 4.07 – 4.00 (m, 1H), 3.96 – 3.78 (m, 2H), 3.69 – 3.60 (m, 1H), 2.29 (s, 2H), 1.10 (t, *J* = 7.1 Hz, 2H), 0.94 (t, *J* = 7.1 Hz, 2H). **Anti ^{13}C NMR** (126 MHz, $CDCl_3$) δ 168.6, 167.9, 137.0, 135.2, 130.1, 128.8, 61.5, 61.1, 56.0, 48.4, 21.2, 13.9, 13.8. **IR**: ν_{max}: 1751, 1722, 1251, 1146 cm^{-1} **Anti HRMS** (ES+) [$C_{30}H_{38}O_8$ + Na^+] calc. 549.2464 found 549.2466

Syn MP 62 – 64 ˚C. **^{1}H NMR** (500 MHz, $CDCl_3$) δ 6.96 (s, 1H), 4.52 – 4.27 (m, 1H), 3.93 – 3.58 (m, 1H), 2.29 (s, 1H), 1.41 (t, *J* = 7.1 Hz, 1H), 0.81 (t, *J* = 7.1 Hz, 1H). **Syn ^{13}C NMR** (126 MHz, $CDCl_3$) δ 168.2, 167.5, 137.0, 132.9, 130.3, 128.4, 62.0, 61.2, 55.8, 46.1, 21.2, 14.3, 13.7. **IR**: ν_{max}: 1740, 1721, 1228, 1143, 1034 cm^{-1} **Syn HRMS** (ES+) [$C_{30}H_{38}O_8$ + Na^+] calc. 549.2464 found 549.2465

Tetraethyl 2,3-bis(4-methoxyphenyl)butane-1,1,4,4-tetracarboxylate (10)

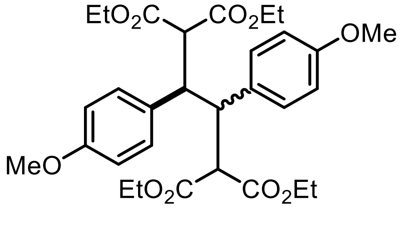

The title compound was prepared by general procedure C as a mixture of diastereomers (85%, 3:1, 237 mg) as a white solid. The anti-diastereomer was separated by recycling preparative HPLC using 80:20 hexane:EtOAc. **Anti ^{1}H NMR** (500 MHz, $CDCl_3$) δ 7.21 (d, *J* = 8.7 Hz, 1H), 6.79 (d, *J* = 8.8 Hz, 1H), 4.01 (dd, *J* = 5.6, 2.2 Hz, 1H), 3.97 – 3.81 (m, 2H), 3.78 (s, 2H), 3.61 (dd, *J* = 5.6, 2.2 Hz, 1H), 1.11 (t, *J* = 7.1 Hz, 2H), 0.97 (t, *J* = 7.1 Hz, 2H). **Anti ^{13}C NMR** (126 MHz, $CDCl_3$) δ 168.6, 168.0, 158.9, 131.4, 130.3, 113.5, 61.5, 61.2, 55.9, 55.3, 48.2, 14.0, 13.9. **Anti HRMS** (ES+) [$C_{30}H_{38}O_{10}$ + Na^+] calc. 581.2363 found 581.2379

Mixed ^{1}H NMR (500 MHz, $CDCl_3$) δ 6.71 (s, 4H), 4.47 – 4.30 (m, 1H), 3.96 – 3.89 (m, 2H), 3.78 (s, 1H), 1.41 (t, *J* = 7.1 Hz, 1H), 0.84 (t, *J* = 7.1 Hz, 1H). **Mixed ^{13}C NMR** ^{13}C NMR (126 MHz, $CDCl_3$) δ 168.62, 168.16, 167.99, 167.49, 158.92, 158.89, 131.40, 131.35, 130.28, 127.95, 113.49, 113.05, 62.08, 61.50, 61.26, 61.14, 55.93, 55.89, 55.32, 55.30, 48.14, 45.93, 14.32, 13.98, 13.88, 13.76.

Tetraethyl 2,3-bis(4-(methoxycarbonyl)phenyl)butane-1,1,4,4-tetracarboxylate (11)

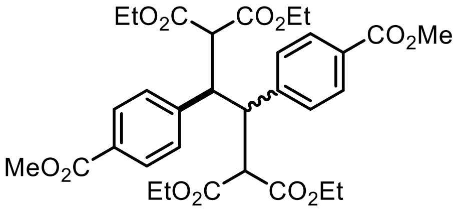

The title compound was prepared by general procedure C as a mixture of diastereomers (85%, 17:10, 522 mg) as a white solid. The anti diastereomer was separated by column chromatography. **MP** 156 – 158 ˚C. **Anti ^{1}H NMR** (500 MHz, $CDCl_3$) δ 7.91 (d, *J* = 8.5 Hz, 1H), 7.29 (d, *J* = 8.5 Hz, 1H), 4.15 (dd, *J* = 6.2, 2.1 Hz, 1H), 3.96 (q, *J* = 7.1 Hz, 1H), 3.88 (s, 1H), 3.80 (q, *J* = 7.1 Hz, 1H), 3.61 (dd, *J* = 6.2, 2.2 Hz, 1H), 1.11 (t, *J* = 7.1 Hz, 1H), 0.89 (t, *J* = 7.1 Hz, 2H).**Anti ^{13}C NMR** (126 MHz, $CDCl_3$) δ 168.1, 167.4, 166.9, 143.4, 130.2, 129.6, 129.5, 61.9, 61.5, 55.1, 52.3, 48.2, 14.0, 13.8. **IR**: ν_{max}: 1744, 1728, 1718, 1265, 1118 cm^{-1} **Anti HRMS** (AP+) [$C_{32}H_{39}O_{12}$] calc. 615.2442 found 615.2441

Mixed ^{1}H NMR (500 MHz, $CDCl_3$) δ 7.84 (s, 16H), 7.04 (t, *J* = 83.0 Hz, 10H), 4.51 – 4.30 (m, 11H), 4.04 – 3.94 (m, 8H), 3.90 (d, *J* = 8.7 Hz, 20H), 3.84 – 3.75 (m, 11H), 3.73 (dt, *J* = 4.4, 1.7 Hz, 7H), 1.42 (t, *J* = 7.1 Hz, 16H), 0.83 (t, *J* = 7.1 Hz, 17H). **Mixed ^{13}C NMR** (126 MHz, $CDCl_3$) δ 167.61, 166.75, 166.73, 141.20, 129.49, 129.41, 129.02, 62.34, 61.46, 55.15, 52.18, 46.35, 14.17, 13.61.

Tetraethyl bis(4-cyanophenyl)butane-1,1,4,4-tetracarboxylate (12)

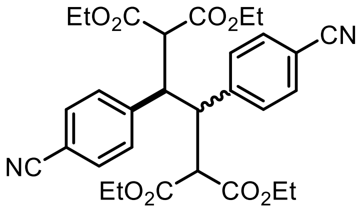

The title compound was prepared by general procedure C as a mixture of diastereomers (88%, 5:4, 242 mg) as a white solid. The diastereomers were separated by column chromatography. **MP** 204 – 207 ˚C. **Anti ^{1}H NMR** (500 MHz, $CDCl_3$) δ 7.59 (d, *J* = 8.5 Hz, 1H), 7.42 (d, *J* = 8.5 Hz, 1H), 4.19 (dd, *J* = 5.5, 2.1 Hz, 1H), 4.00 (qd, *J* = 7.1, 2.0 Hz, 1H), 3.89 (q, *J* = 7.1 Hz, 1H), 3.54 (dd, *J* = 5.5, 2.2 Hz, 1H), 1.13 (t, *J* = 7.1 Hz, 1H), 0.98 (t, *J* = 7.1 Hz, 1H). **Anti ^{13}C NMR** (126 MHz, $CDCl_3$) δ 167.8, 167.2, 143.3, 132.1, 131.0, 118.5, 112.0, 62.1, 61.8, 54.7, 47.9, 14.0, 13.9. **IR**: ν_{max}: 2226, 1748, 1717, 1310, 1146 cm^{-1} **Anti HRMS** (ES+) [$C_{30}H_{31}N_2O_8$] calc. 547.2080 found 547.2079

Syn MP 104 – 106 ˚C. **^{1}H NMR** (500 MHz, $CDCl_3$) δ 7.50 (br, s, 1H), 6.81 (br, s, 1H), 4.56 – 4.27 (m, 1H), 3.96 (q, *J* = 1.9 Hz, 1H), 3.90 – 3.76 (m, 1H), 3.68 (q, *J* = 1.8 Hz, 1H), 1.41 (t, *J* = 7.1 Hz, 1H), 0.89 (t, *J* = 7.1 Hz, 1H). **Syn ^{13}C NMR** (126 MHz, $CDCl_3$) δ 167.4, 166.5, 141.5, 131.8, 130.6 (br), 118.4, 112.0, 62.8, 61.9, 55.0, 46.5, 14.2, 13.8. **IR**: ν_{max}: 2224, 1757, 1736, 1204, 1165 cm^{-1} **Syn HRMS** (ES+) [$C_{30}H_{31}N_2O_8$] calc. 547.2080 found 547.2078

Diethyl 2,5-dicyano-3,4-bis(4-fluorophenyl)hexanedioate (14)

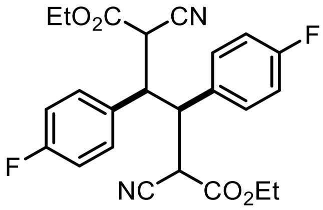

The title compound was prepared by general procedure C as a single diastereomers (55%, 121 mg) as a white solid. **MP** 173 – 176 ˚C. **^{1}H NMR** (500 MHz, $CDCl_3$) δ 7.60 (s, 1H), 7.16 (t, *J* = 8.4 Hz, 1H), 4.10 – 3.91 (m, 2H), 3.49 (dd, *J* = 2.4, 1.8 Hz, 1H), 1.04 (t, *J* = 7.1 Hz, 2H). **^{13}C NMR** (126 MHz, $CDCl_3$) δ 164.2 (s), 163.2 (d, *J* = 249.7 Hz), 130.2 (d, *J* = 3.4 Hz), 116.8 (d, *J* = 21.8 Hz), 114.6 (s), 63.1 (s), 47.3 (s), 42.3 (s), 13.8 (s). **^{19}F NMR** (376 MHz, $CDCl_3$) δ -111.3. **IR**: ν_{max}: 2232, 1749, 1510, 1269, 1228 1167 cm^{-1} **HRMS** (AP+) [$C_{24}H_{23}N_2F_2O_4$] calc. 441.1626 found 441.1626.

Tetraethyl 2,3-bis(4-chlorophenyl)butane-1,1,4,4-tetracarboxylate (13)

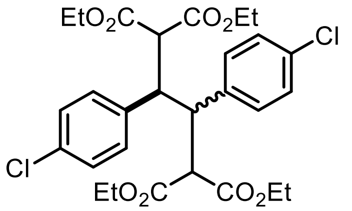

The title compound was prepared by general procedure C as a mixture of diastereomers (82%, 13:10, 231 mg) as a white solid. The anti diastereomer was separated by column chromatography and the syn diastereomer was separated using recycling preparative HPLC using 80:20 hexane:EtOAc. **MP** 170 - 173 ˚C. **Anti ^{1}H NMR** (500 MHz, $CDCl_3$) δ 7.25 – 7.19 (m, 1H), 4.05 (dd, *J* = 5.7, 2.2 Hz, 1H), 4.02 – 3.91 (m, 1H), 3.87 (qt, *J* = 4.8, 2.5 Hz, 1H), 3.58 (dd, *J* = 5.7, 2.2 Hz, 1H), 1.13 (t, *J* = 7.1 Hz, 1H), 0.98 (t, *J* = 7.1 Hz, 1H). **Anti ^{13}C NMR** (126 MHz, $CDCl_3$) δ 168.2, 167.6, 136.6, 133.7, 131.6, 128.5, 61.8, 61.5, 55.3, 47.9, 29.9, 14.0, 13.8. **IR**: ν_{max}: 1746, 1719, 1308, 1171, 1152, 1011 cm^{-1} **Anti HRMS** (ES+) [$C_{28}H_{32}O_8Cl_2$ + Na^+] calc. 589.1372 found 583.1376

Syn ^{1}H NMR (500 MHz, $CDCl_3$) δ 7.17 (s, 1H), 4.46 – 4.32 (m, 1H), 3.90 – 3.75 (m, 2H), 3.64 (d, *J* = 11.9 Hz, 1H), 1.41 (t, *J* = 7.1 Hz, 2H), 0.87 (t, *J* = 7.1 Hz, 2H). **Syn ^{13}C NMR** (126 MHz, $CDCl_3$) δ 167.8, 167.0, 134.4, 133.7, 128.1, 62.4, 61.6, 55.5, 45.9, 14.3, 13.8. **Syn HRMS** (ES+) [$C_{28}H_{32}O_8Cl_2$ + Na^+] calc. 589.1372 found 583.1376

Tetramethyl 2,3-bis(4-fluorophenyl)butane-1,1,4,4-tetracarboxylate (15)

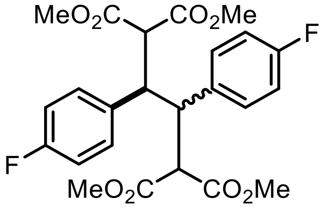

The title compound was prepared by general procedure C as a mixture of diastereomers (62%, 3:2, 149 mg) as a white solid. The diastereomers were separated by column chromatography. **MP** 161 – 162 ˚C. **Anti ^{1}H NMR** (500 MHz, $CDCl_3$) δ 7.32 – 7.27 (m, 1H), 6.99 (t, *J* = 8.7 Hz, 1H), 4.09 (dd, *J* = 5.1, 2.3 Hz, 1H), 3.60 (dd, *J* = 5.1, 2.3 Hz, 1H), 3.48 (s, 1H), 3.41 (s, 1H). **Anti ^{13}C NMR** (126 MHz, $CDCl_3$) δ 168.71 (s), 168.14 (s), 162.43 (d, *J* = 246.9 Hz), 133.64 (d, *J* = 3.4 Hz), 131.80 (d, *J* = 6.7 Hz), 115.43 (d, *J* = 21.2 Hz), 55.47 (s), 52.75 (s), 52.47 (s), 47.96 (s).j **Anti ^{19}F NMR** (471 MHz, $CDCl_3$) δ -114.2 (s). **IR**: ν_{max}: 1747, 1504, 1433, 1258, 1157 cm^{-1} **Anti HRMS** (ES+) [$C_{24}H_{24}F_2O_8$ + Na^+] calc. 501.1337 found 501.1334

Syn MP 107 – 108 ˚C **^{1}H NMR** (500 MHz, $CDCl_3$) δ 6.88 (s, 1H), 3.94 (s, 1H), 3.80 (q, *J* = 1.6 Hz, 1H), 3.72 (q, *J* = 1.5 Hz, 1H), 3.35 (s, 1H). **Syn ^{13}C NMR** (126 MHz, $CDCl_3$) δ 168.2 (s), 167.5 (s), 162.3 (d, *J* = 247.0 Hz), 131.3 (d, *J* = 3.3 Hz), 114.9 (d, *J* = 21.6 Hz), 55.4 (s), 53.4

(s), 52.6 (s), 46.1 (s). **IR**: ν_{max}: 1755, 1734, 1508, 1258, 1229, 1153 cm^{-1} **Syn ^{19}F NMR** (471 MHz, $CDCl_3$) δ -114.3 (s). **Syn HRMS** (ES+) [$C_{24}H_{24}F_2O_8$ + Na^+] calc. 501.1337 found 501.1331

Tetraisopropyl 2,3-bis(4-fluorophenyl)butane-1,1,4,4-tetracarboxylate (16)

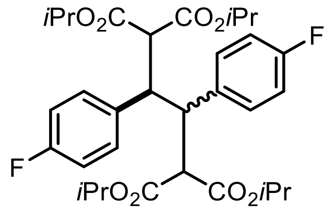

The title compound was prepared by general procedure C as a mixture of diastereomers (51%, 1:1,126 mg) as a white solid. The anti diastereomer was separated by column chromatography and the syn diastereomer was separated using recycling preparative HPLC using 80:20 hexane:EtOAc. **MP** 167 – 170 ˚C. **Anti ^{1}H NMR** (500 MHz, $CDCl_3$) δ 7.22 – 7.16 (m, 3H), 6.87 (t, *J* = 8.7 Hz, 2H), 4.77 (hept, *J* = 6.3 Hz, 1H), 4.66 (hept, *J* = 6.3 Hz, 1H), 4.04 – 3.89 (m, 1H), 3.46 (dd, *J* = 5.8, 2.2 Hz, 1H), 1.07 (d, *J* = 6.3 Hz, 3H), 0.95 (d, *J* = 6.3 Hz, 3H), 0.92 (d, *J* = 6.3 Hz, 3H), 0.86 (d, *J* = 6.3 Hz, 3H). **Anti ^{13}C NMR** (126 MHz, $CDCl_3$) δ 167.96 (s), 167.30 (s), 162.26 (d, *J* = 246.1 Hz), 134.07 (d, *J* = 3.3 Hz), 132.01 (s), 115.03 (d, *J* = 21.1 Hz), 69.30 (s), 68.79 (s), 55.50 (s), 47.72 (s), 21.60 (s), 21.49 (s), 21.44 (s), 21.30 (s). **Anti ^{19}F NMR** (471 MHz, $CDCl_3$) δ -115.16. **IR**: ν_{max}: 1740, 1717, 1506, 1296, 1159, 1123 cm^{-1} **Anti HRMS** (AP+) [$C_{32}H_{41}F_2O_8$] calc. 591.2769 found 591.2776.

Syn ^{1}H NMR (500 MHz, $CDCl_3$) δ 6.88 (s, br, 1H), 1.42 (d, *J* = 6.3 Hz, 1H), 1.36 (d, *J* = 6.3 Hz, 1H), 0.90 (d, *J* = 6.3 Hz, 1H), 0.83 (d, *J* = 6.2 Hz, 1H). **Syn ^{13}C NMR** (126 MHz, $CDCl_3$) δ 167.42 (s), 166.73 (s), 162.22 (d, *J* = 246.4 Hz), 131.98 (d, *J* = 3.3 Hz), 114.70 (d, *J* = 18.2 Hz), 69.86 (s), 68.92 (s), 55.93 (s), 45.49 (s), 21.94 (s), 21.69 (s), 21.34 (s), 21.16 (s). **Syn ^{19}F NMR** (471 MHz, $CDCl_3$) δ -114.83. **Syn HRMS** (AP+) [$C_{32}H_{41}F_2O_8$] calc. 591.2769 found 591.2776.

Tetraallyl 2,3-bis(4-fluorophenyl)butane-1,1,4,4-tetracarboxylate (17)

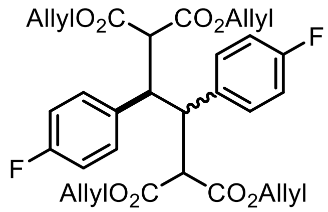

The title compound was prepared by general procedure C as a mixture of diastereomers (77%, 5:3, 151 mg) as a white solid. The anti diastereomer was separated by column

chromatography. **MP** 90 – 92 ˚C. **Anti ^{1}H NMR** (500 MHz, $CDCl_3$) δ 7.29 (dd, *J* = 8.7, 5.3 Hz, 1H), 6.96 (t, *J* = 8.7 Hz, 1H), 5.72 (ddt, *J* = 17.2, 10.4, 5.8 Hz, 1H), 5.64 – 5.53 (m, 1H), 5.19 (ddq, *J* = 10.0, 7.1, 1.4 Hz, 1H), 5.15 – 5.08 (m, 1H), 4.41 – 4.23 (m, 2H), 4.11 (dd, *J* = 5.3, 2.2 Hz, 1H), 3.64 (dd, *J* = 5.3, 2.2 Hz, 1H). **Anti ^{13}C NMR** (126 MHz, $CDCl_3$) δ 167.8 (s), 167.2 (s), 162.4 (d, *J* = 246.8 Hz), 133.5 (d, *J* = 3.3 Hz), 131.9 (s), 131.9 (s), 131.3 (d, *J* = 10.7 Hz), 119.0 (s), 119.0 (s), 115.3 (d, *J* = 21.2 Hz), 66.3 (s), 66.0 (s), 55.5 (s), 47.9 (s). **Anti ^{19}F NMR** (376 MHz, $CDCl_3$) δ -114.4. **IR**: ν_{max}: 1746, 1722, 1504, 1219, 1167, 1126 cm^{-1} **Anti HRMS** (AP+) [$C_{32}H_{33}F_2O_8$] calc. 583.2134 found 583.2151

Mixed ^{1}H NMR (500 MHz, $CDCl_3$) δ 6.87 (s, 3H), 6.03 (ddt, *J* = 17.1, 10.5, 5.8 Hz, 1H), 5.51 – 5.37 (m, 2H), 5.32 (ddd, *J* = 10.5, 2.4, 1.1 Hz, 1H), 5.08 – 4.96 (m, 2H), 4.93 – 4.81 (m, 1H), 4.77 (ddt, *J* = 13.1, 5.9, 1.3 Hz, 1H), 4.27 – 4.15 (m, 2H), 3.89 (t, *J* = 6.7 Hz, 1H), 3.72 (t, *J* = 6.7 Hz, 1H). **Mixed ^{13}C NMR** (126 MHz, $CDCl_3$) δ 167.41 (s), 166.66 (s), 162.30 (d, *J* = 246.9 Hz), 131.57 (s), 131.38 (d, *J* = 3.4 Hz), 131.15 (s), 119.44 (s), 118.96 (s), 118.95 (d, *J* = 6.8 Hz), 114.88 (d, *J* = 20.9 Hz), 66.79 (s), 66.13 (s), 55.60 (s), 45.89 (s). **Mixed ^{19}F NMR** (376 MHz, $CDCl_3$) δ -114.4.

NMR Spectra

Diethyl 2-(4-fluorobenzylidene)malonate

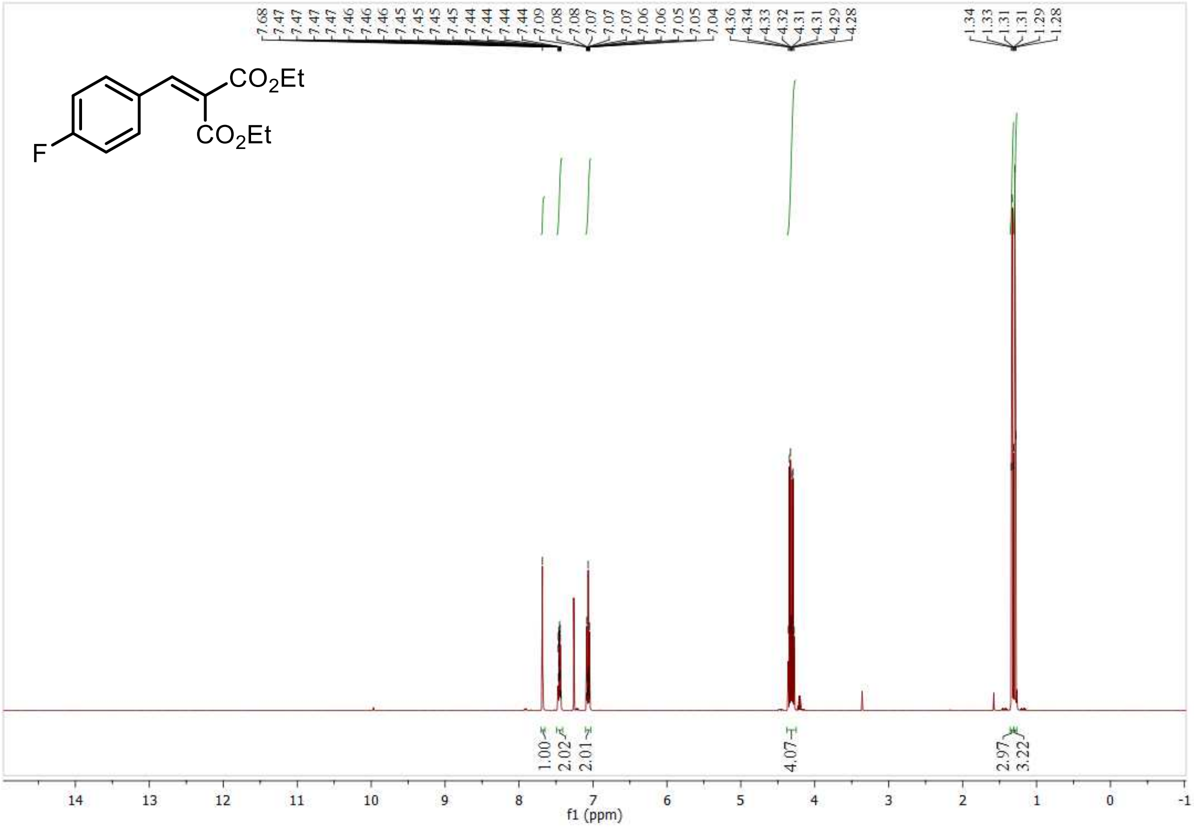

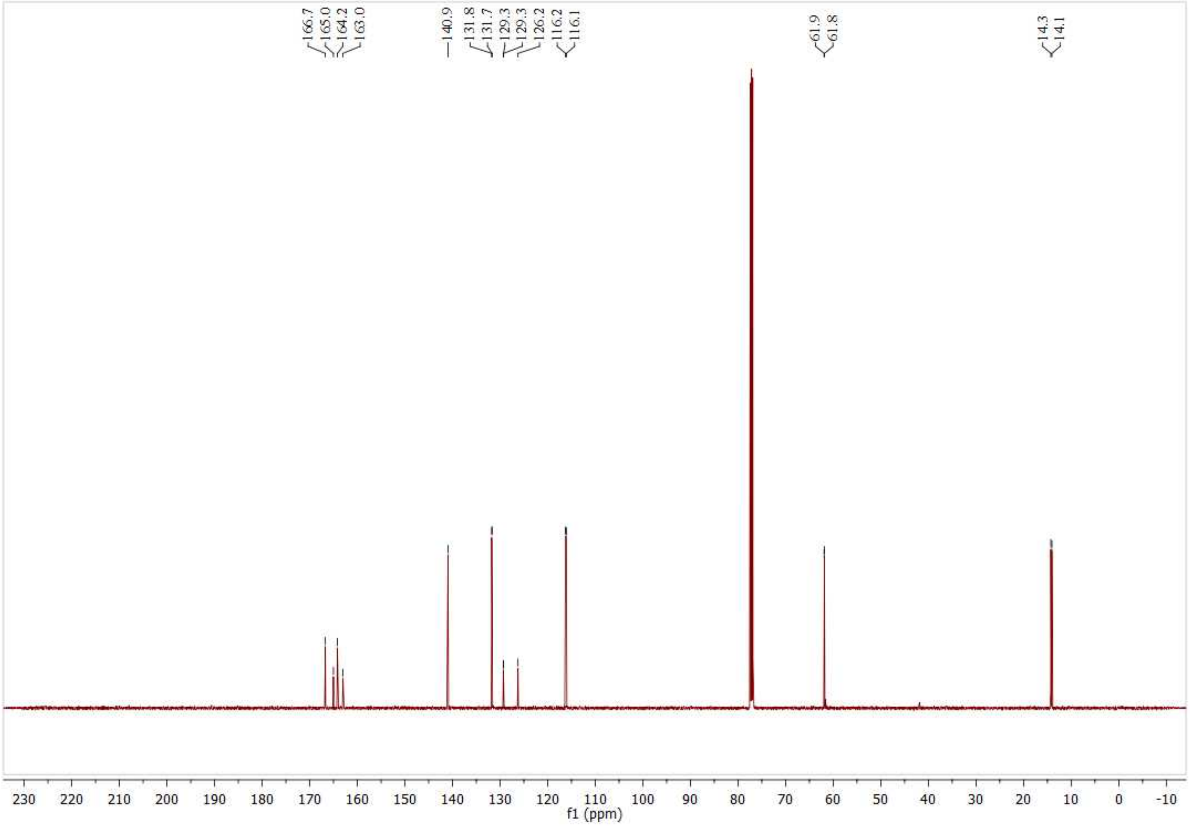

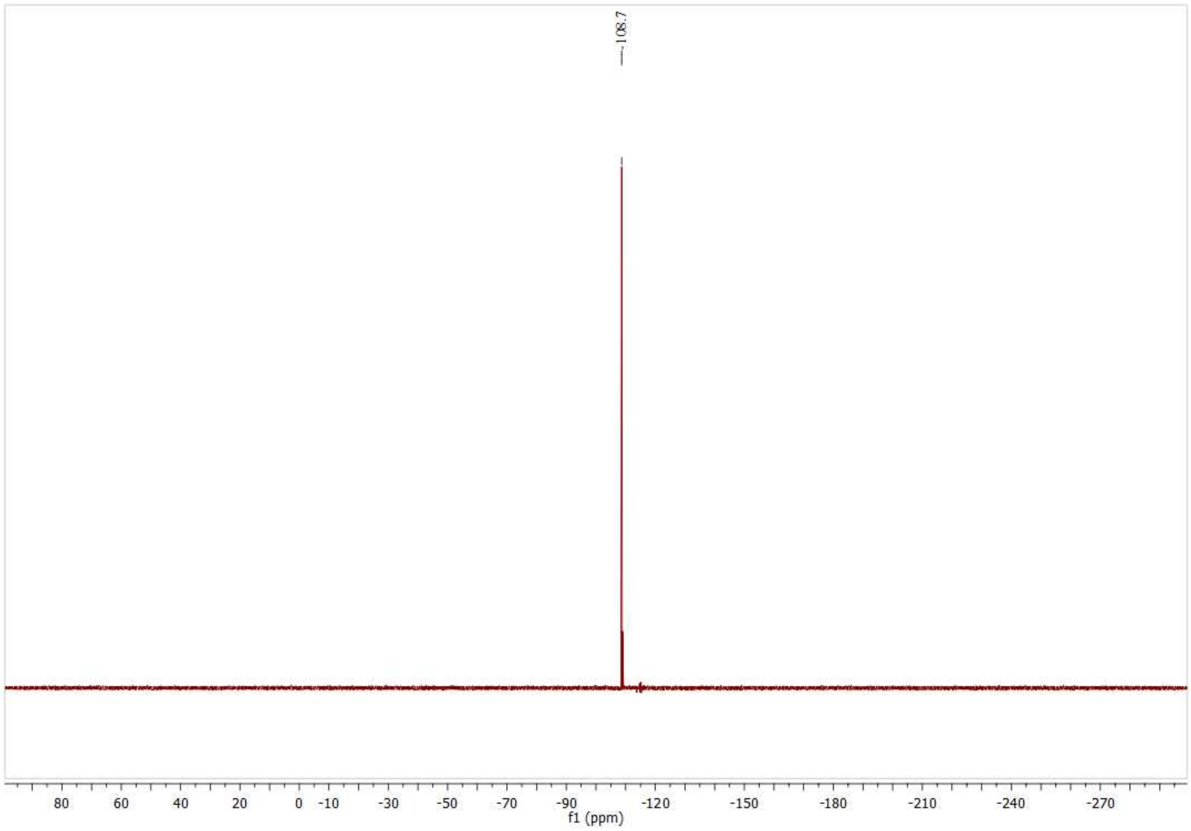
-108.7
80
60
40
20
0
-10
-30
-50
-70
-90
-120
-150
-180
-210
-240
-270
f1 (ppm)

Diethyl 2-(4-methoxybenzylidene)malonate

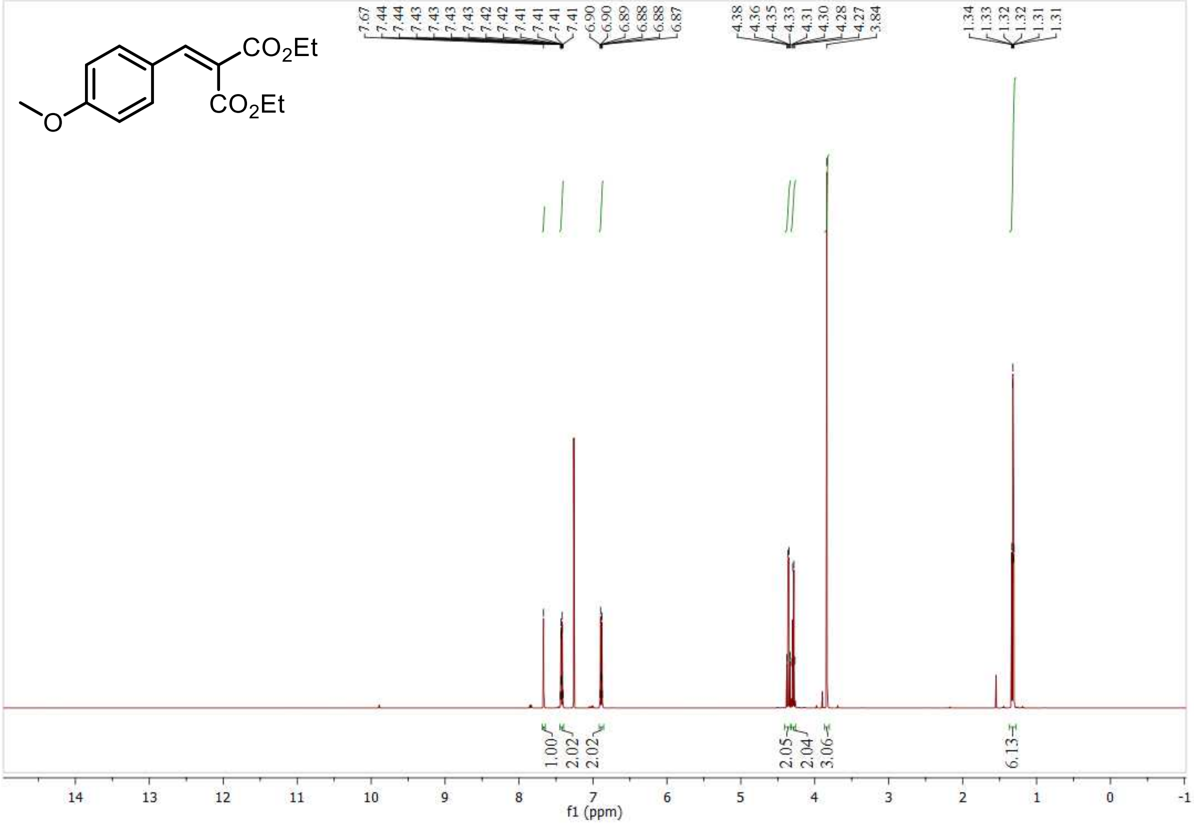

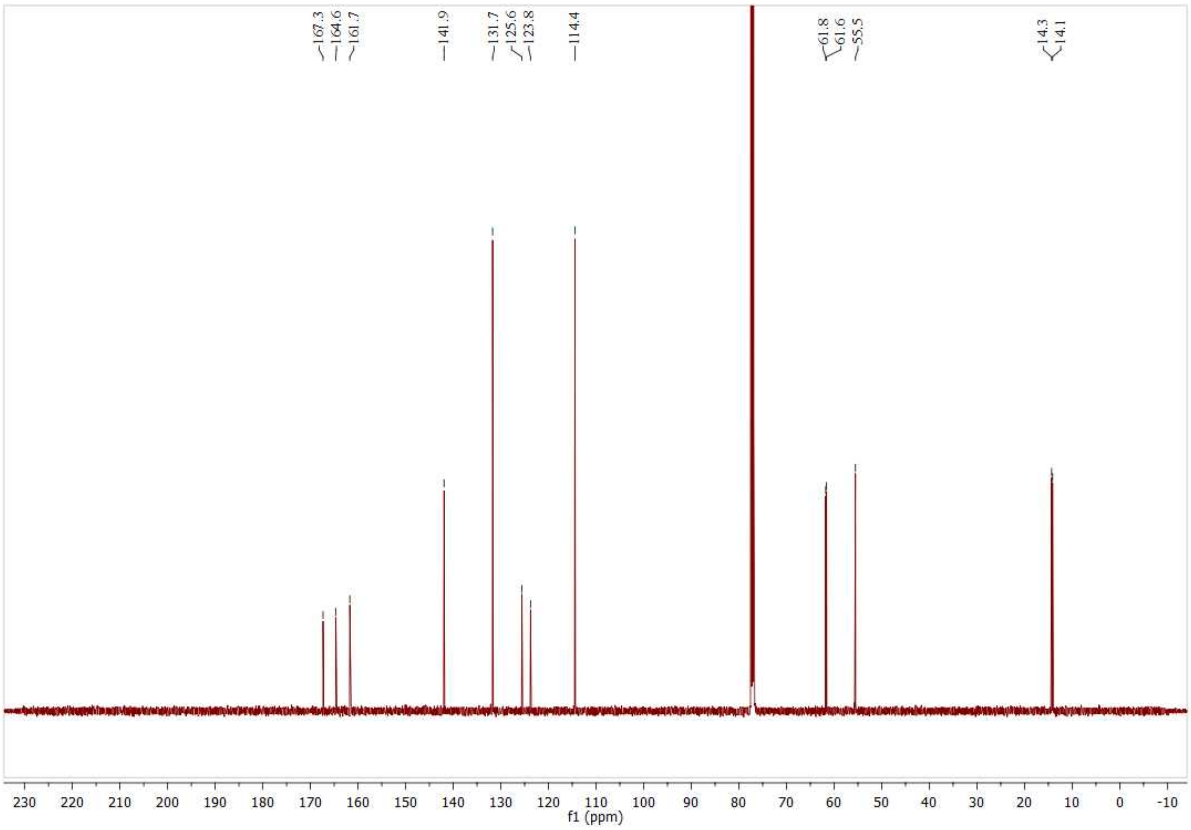

Diethyl 2-(4-(methoxycarbonyl)benzylidene)malonate

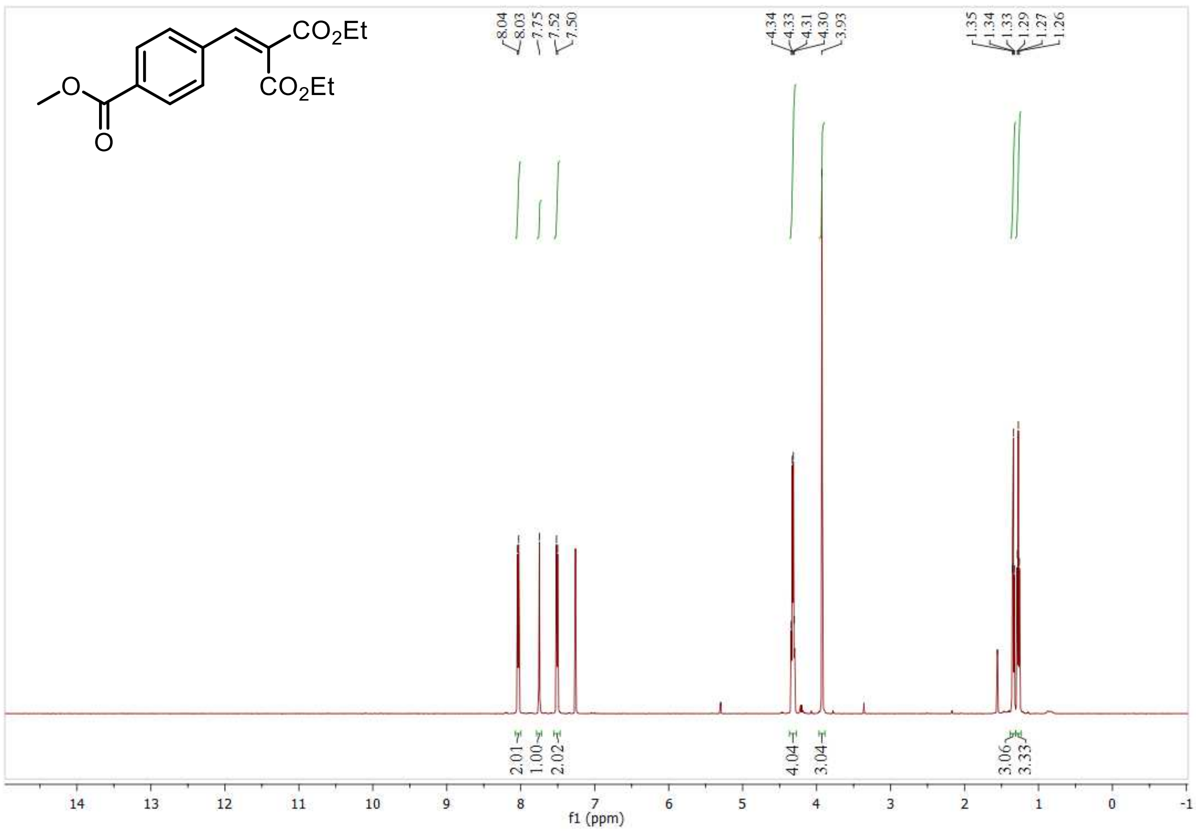

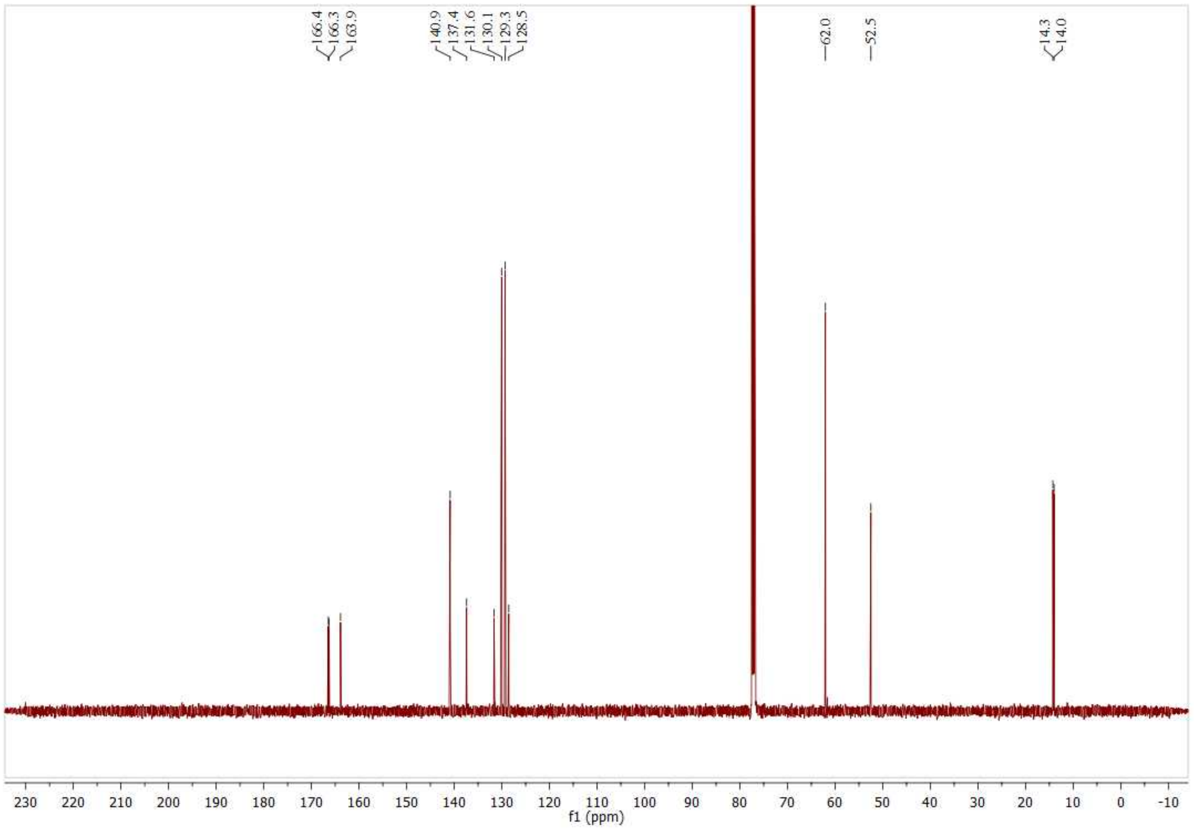

Diethyl 2-(4-cyanobenzylidene)malonate

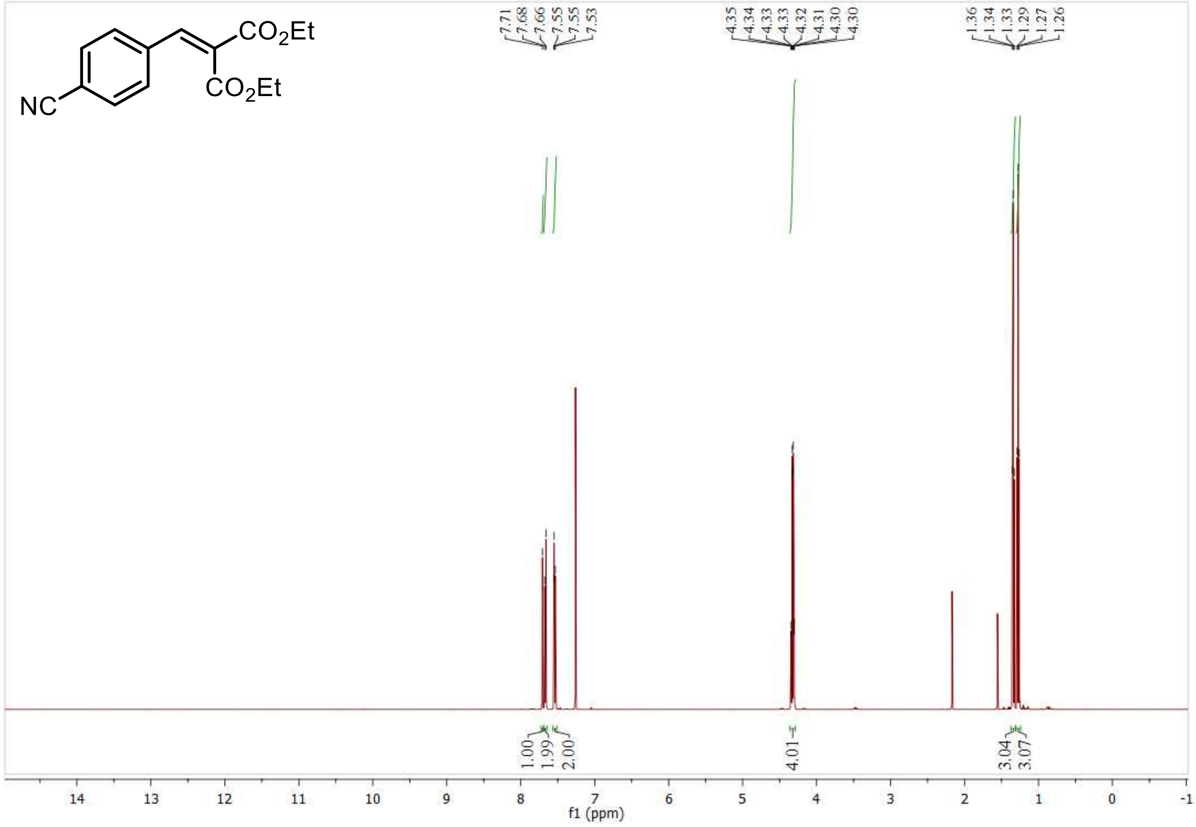

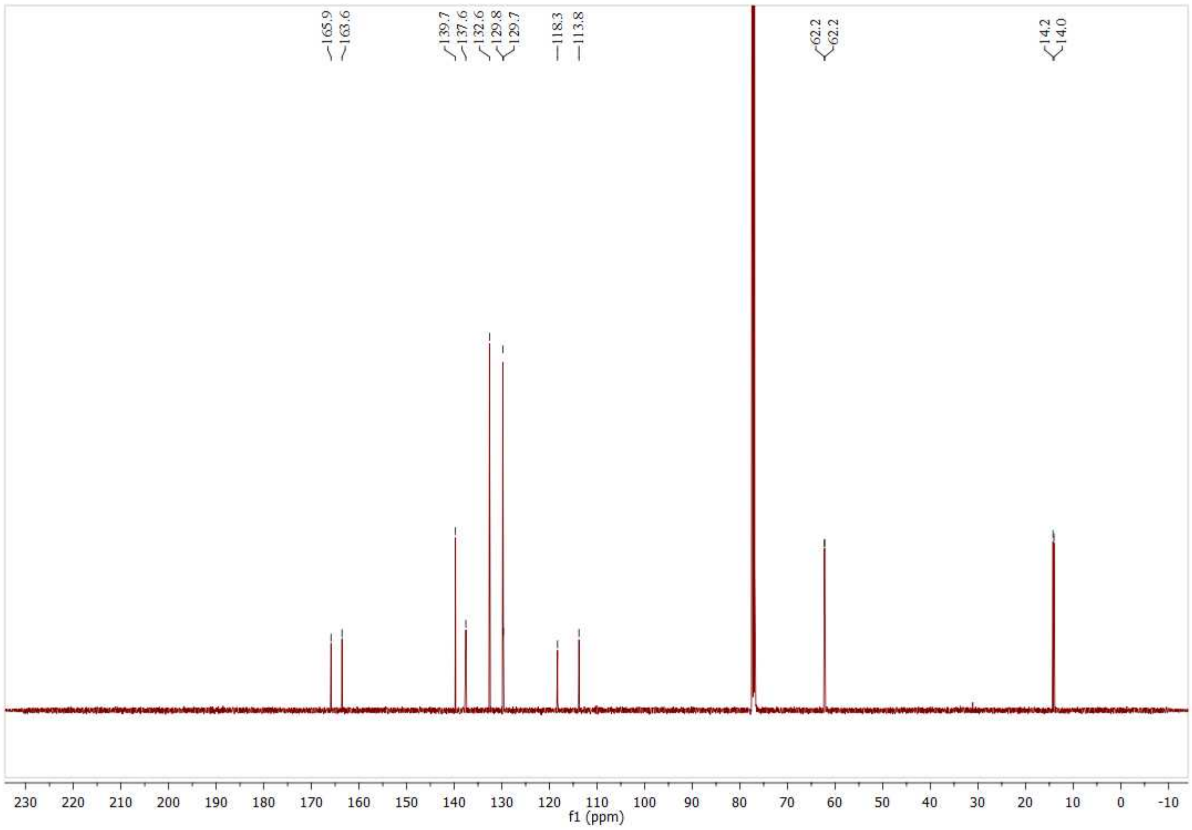

Diethyl 2-(4-bromobenzylidene)malonate

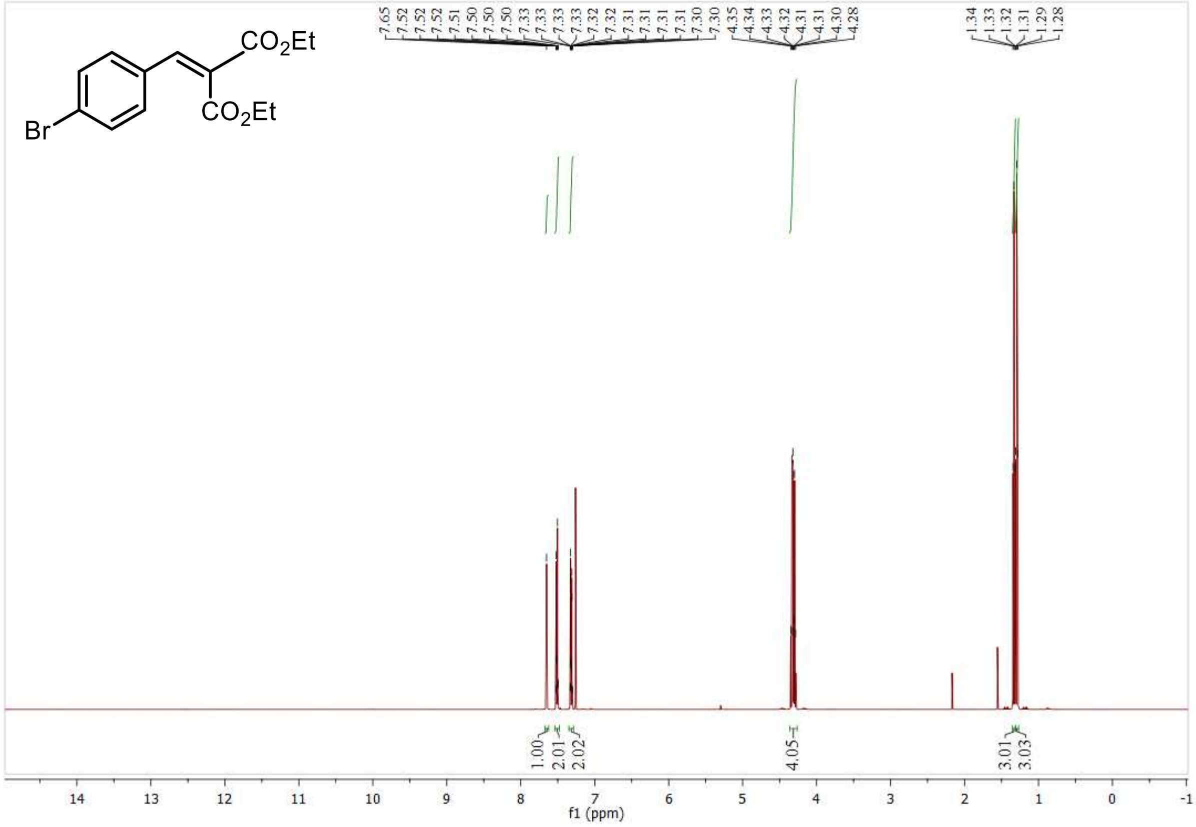

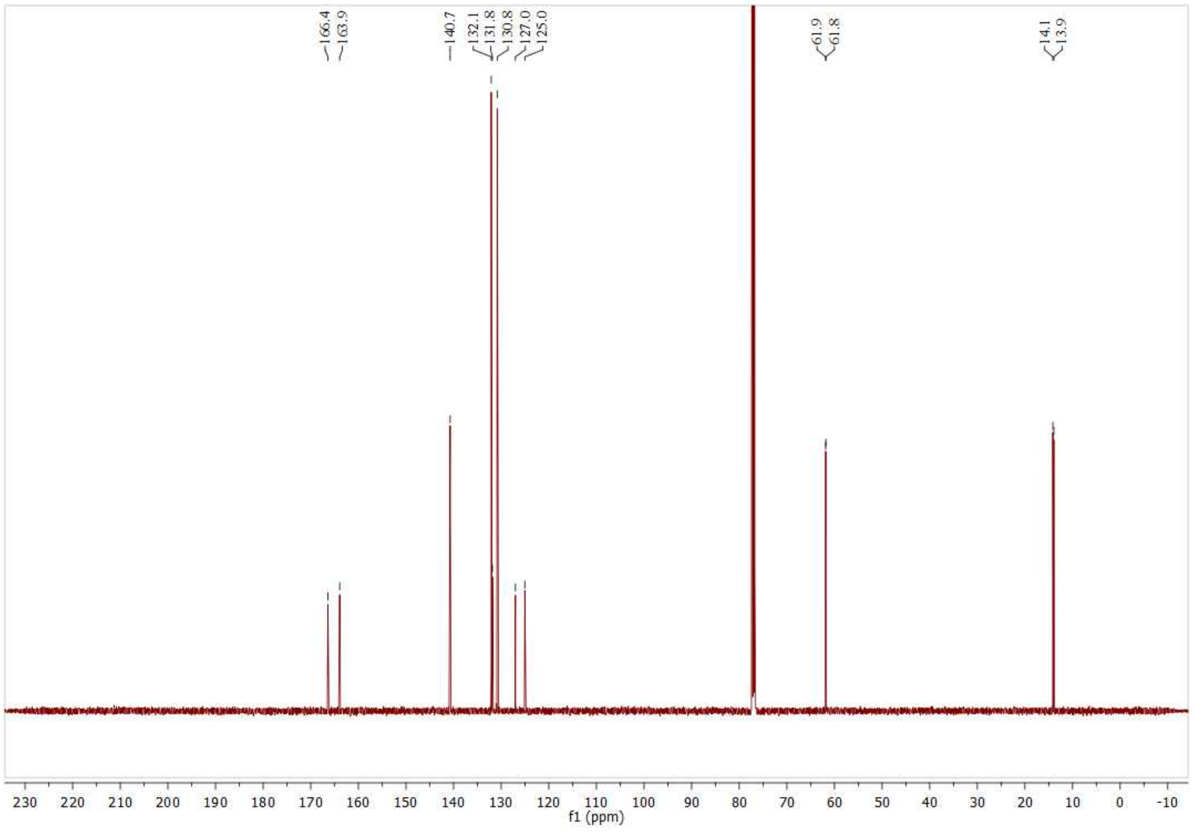

Diethyl 2-(4-chlorobenzylidene)malonate

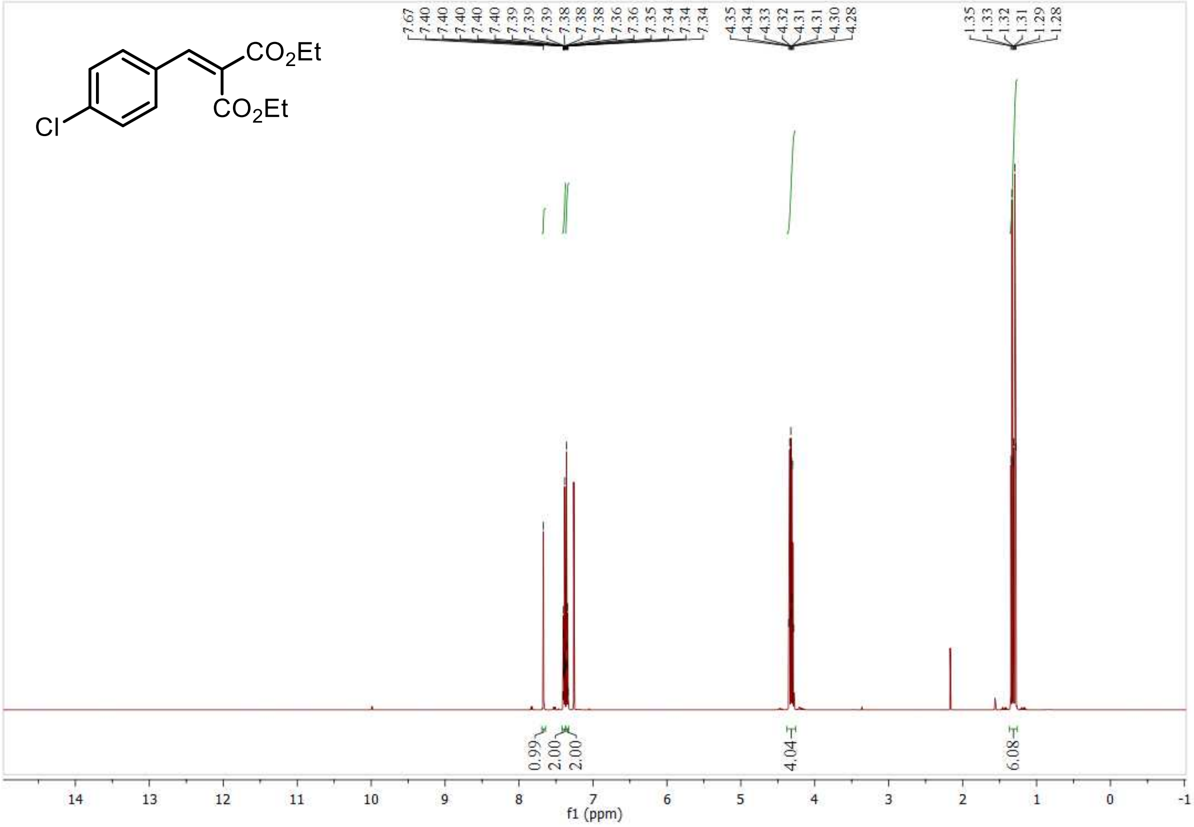

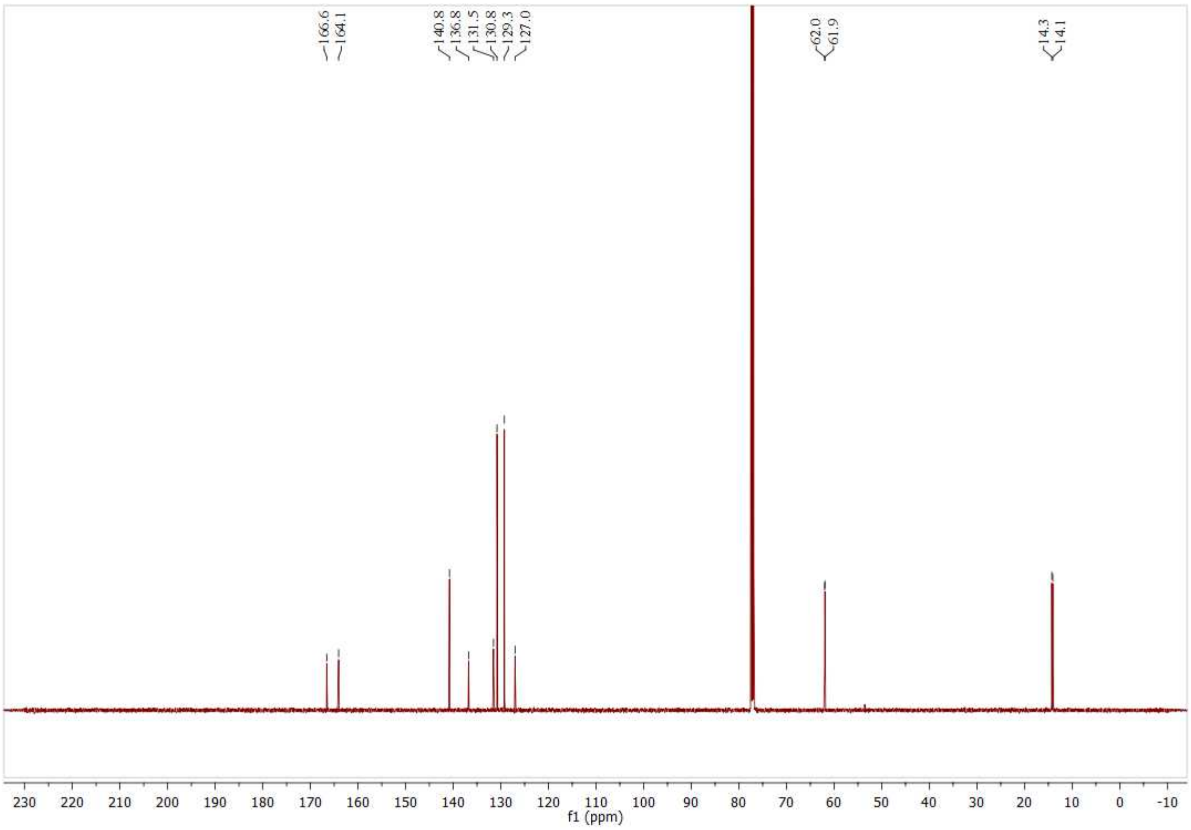

Diethyl 2-(4-(trifluoromethyl)benzylidene)malonate

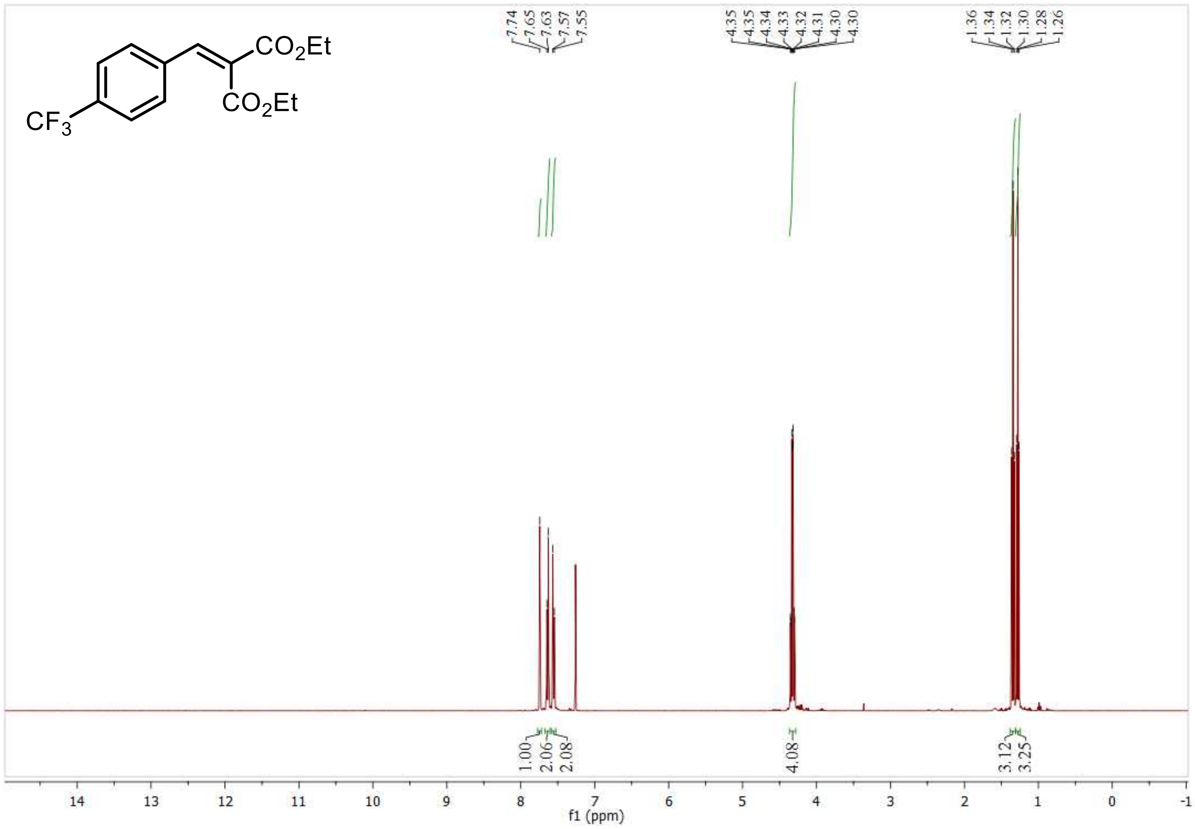

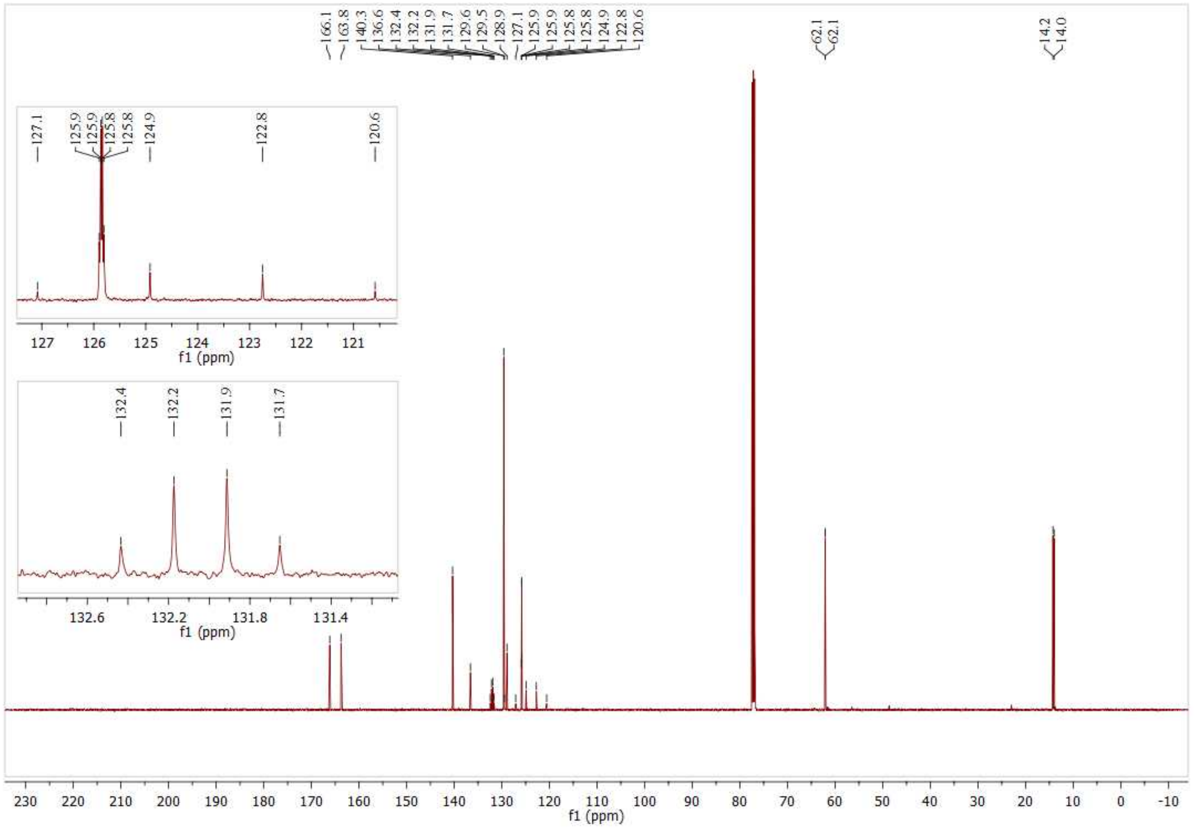

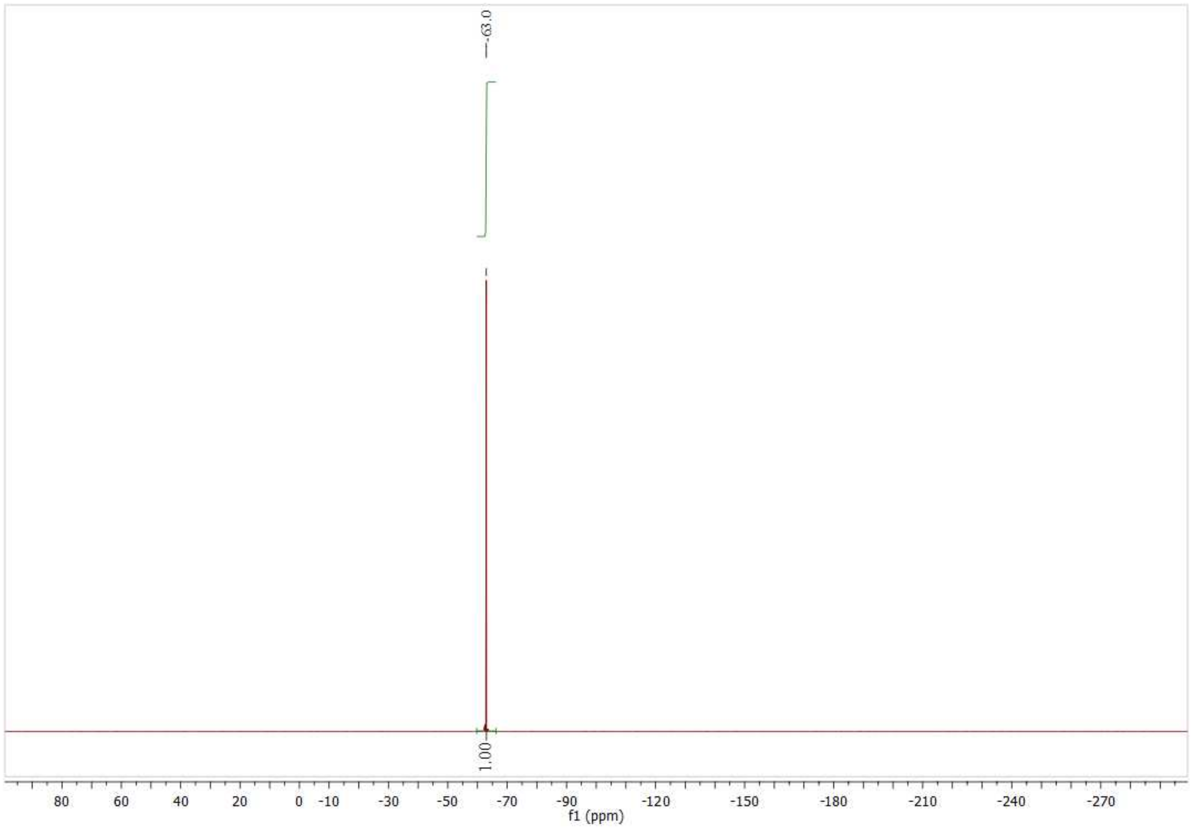
-63.0
1.00
80 60 40 20 0 -10 -30 -50 -70 -90 -120 -150 -180 -210 -240 -270
f1 (ppm)

Diethyl 2-(4-methylbenzylidene)malonate

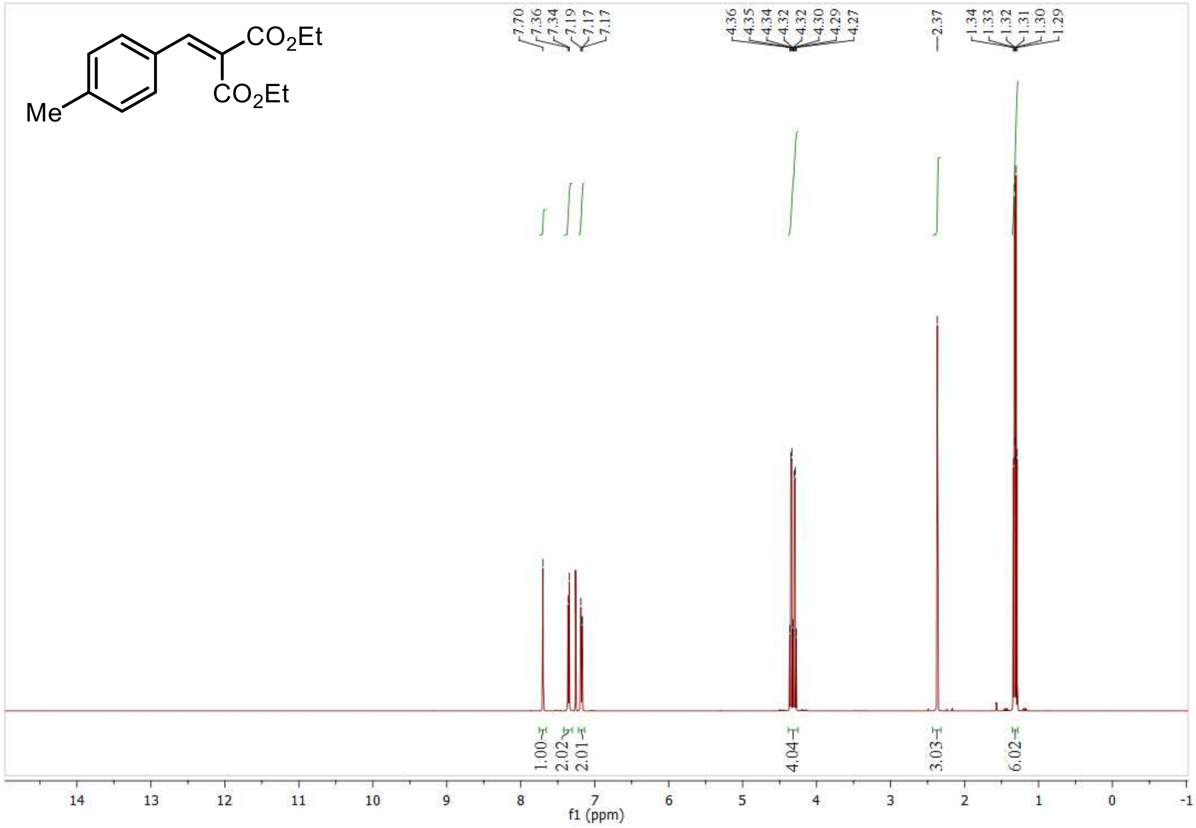

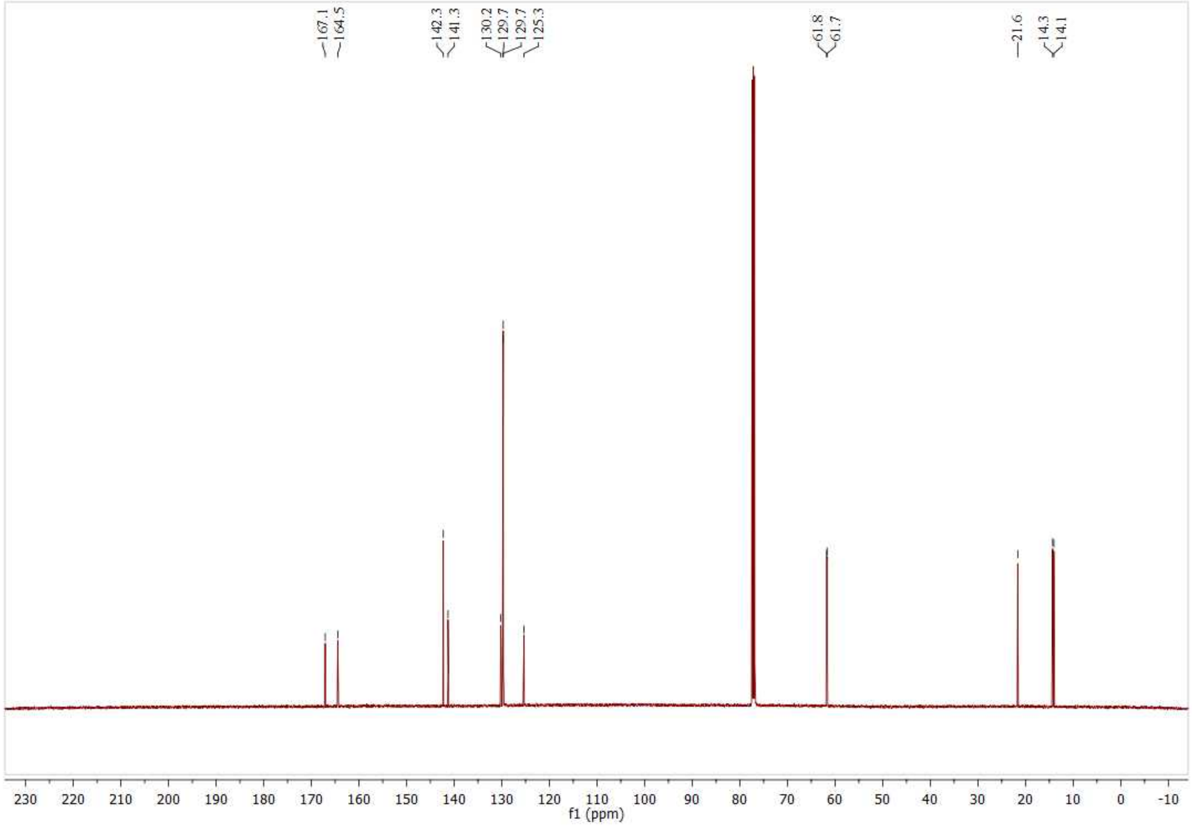

Diethyl 2-(2-fluorobenzylidene)malonate

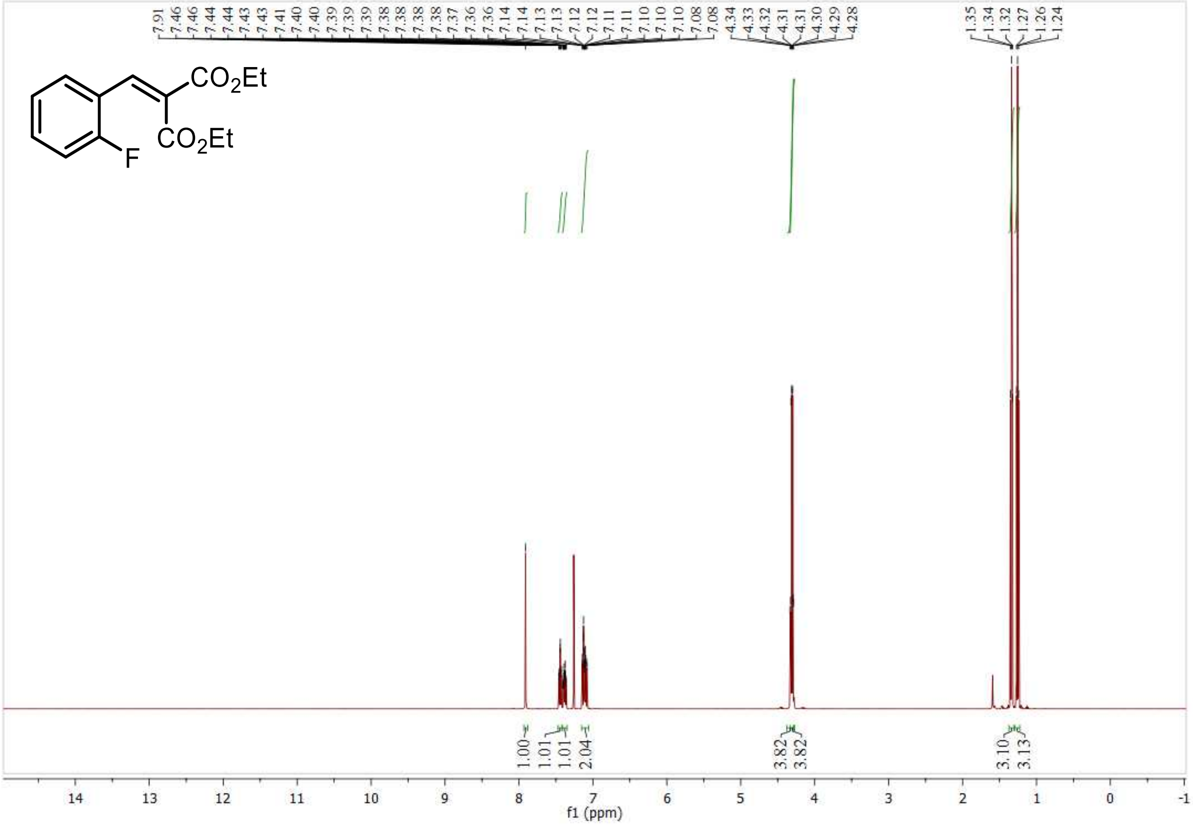

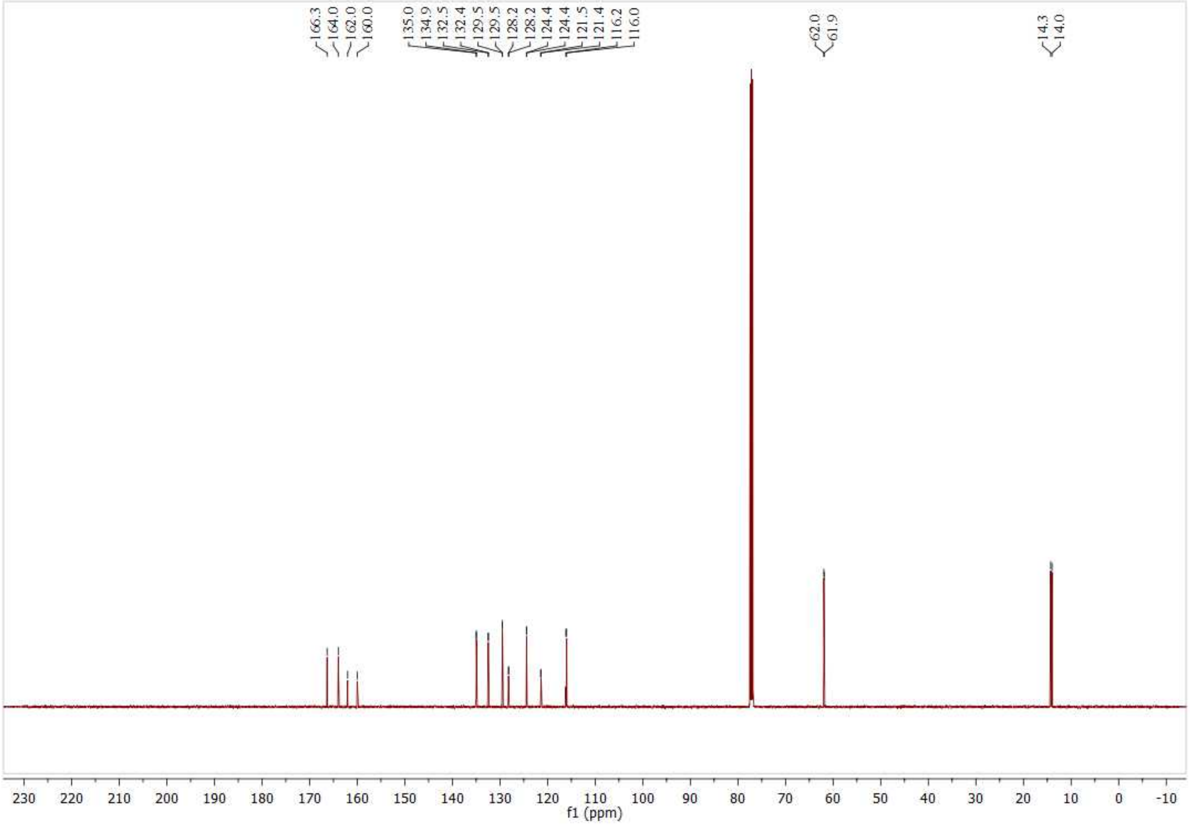

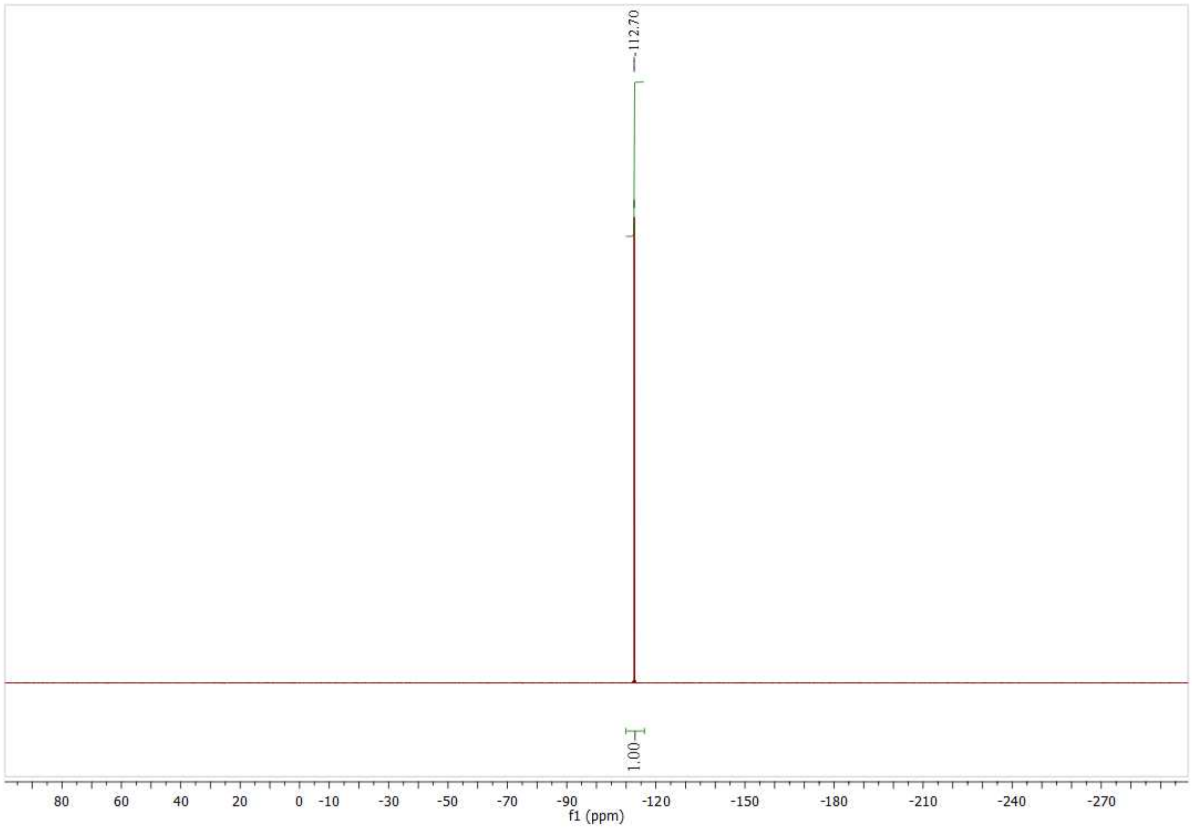
-112.70
1.00
80
60
40
20
0
-10
-30
-50
-70
-90
-120
-150
-180
-210
-240
-270
f1 (ppm)

Diethyl 3-(3-fluorobenzylidene)malonate

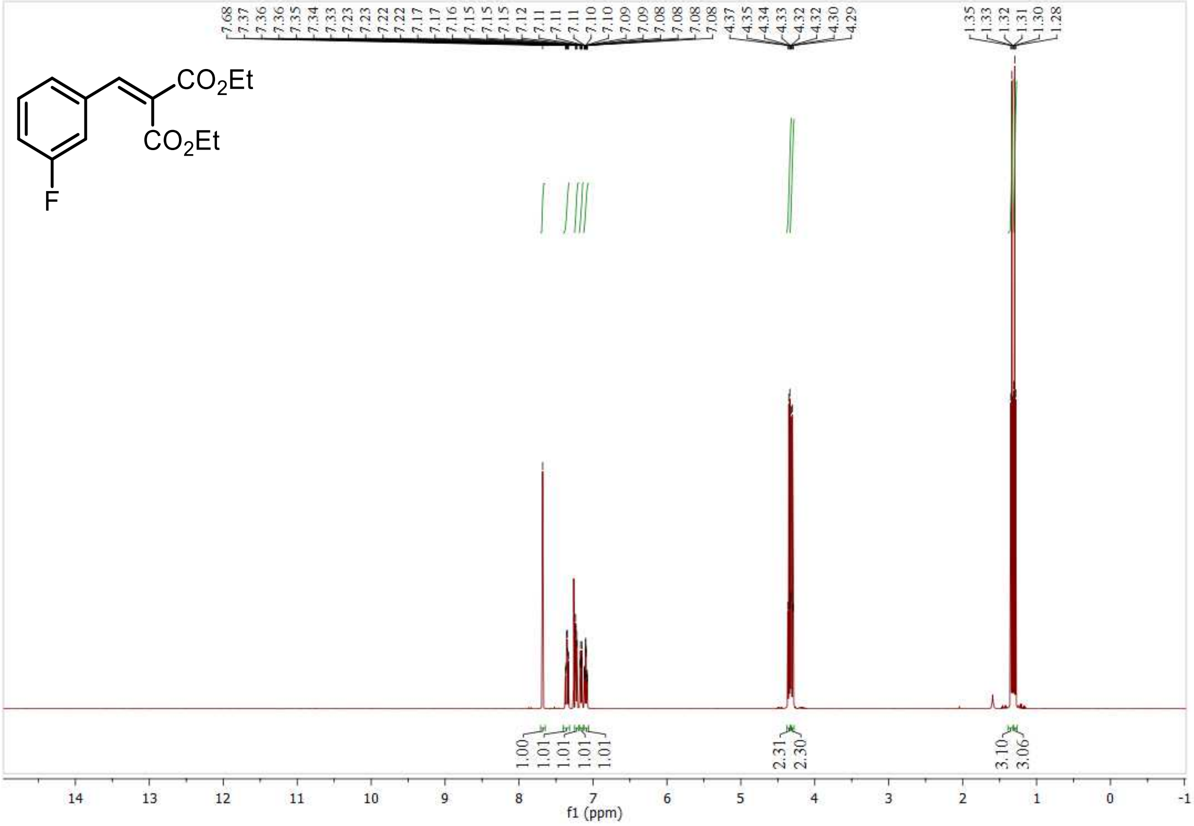

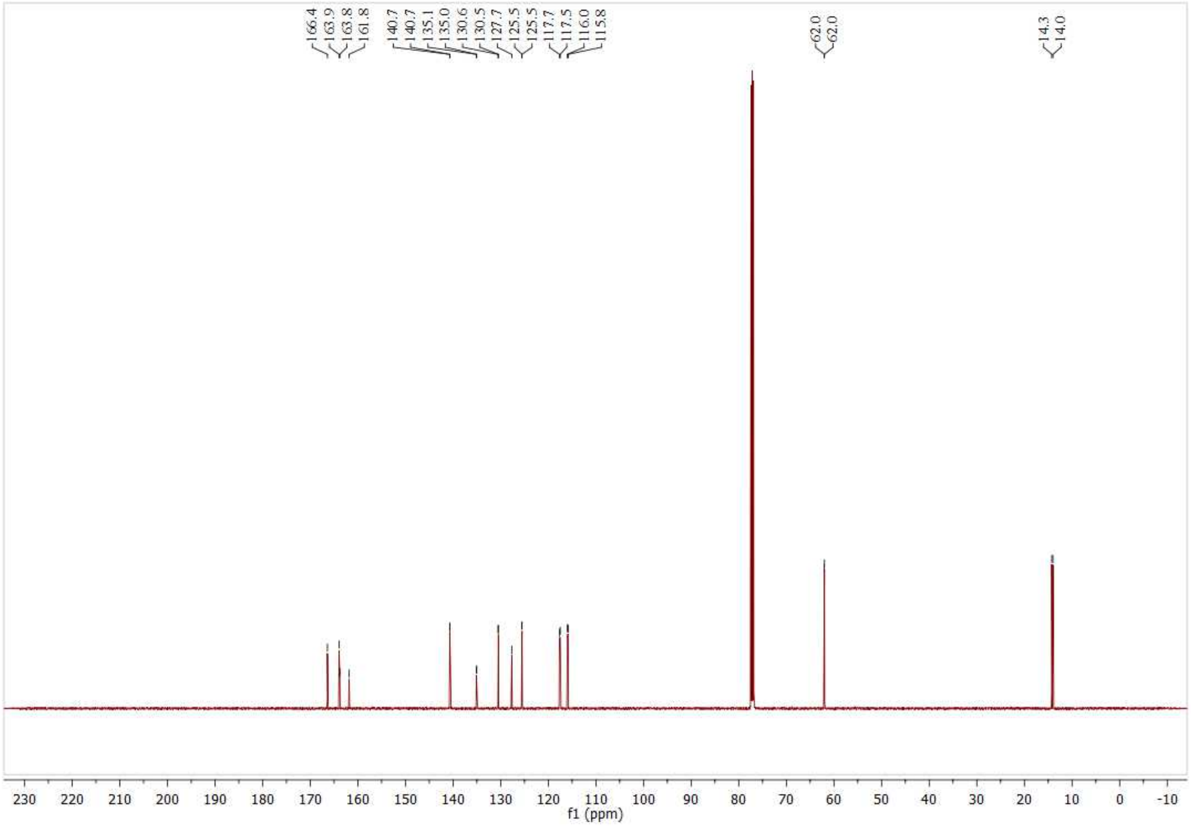

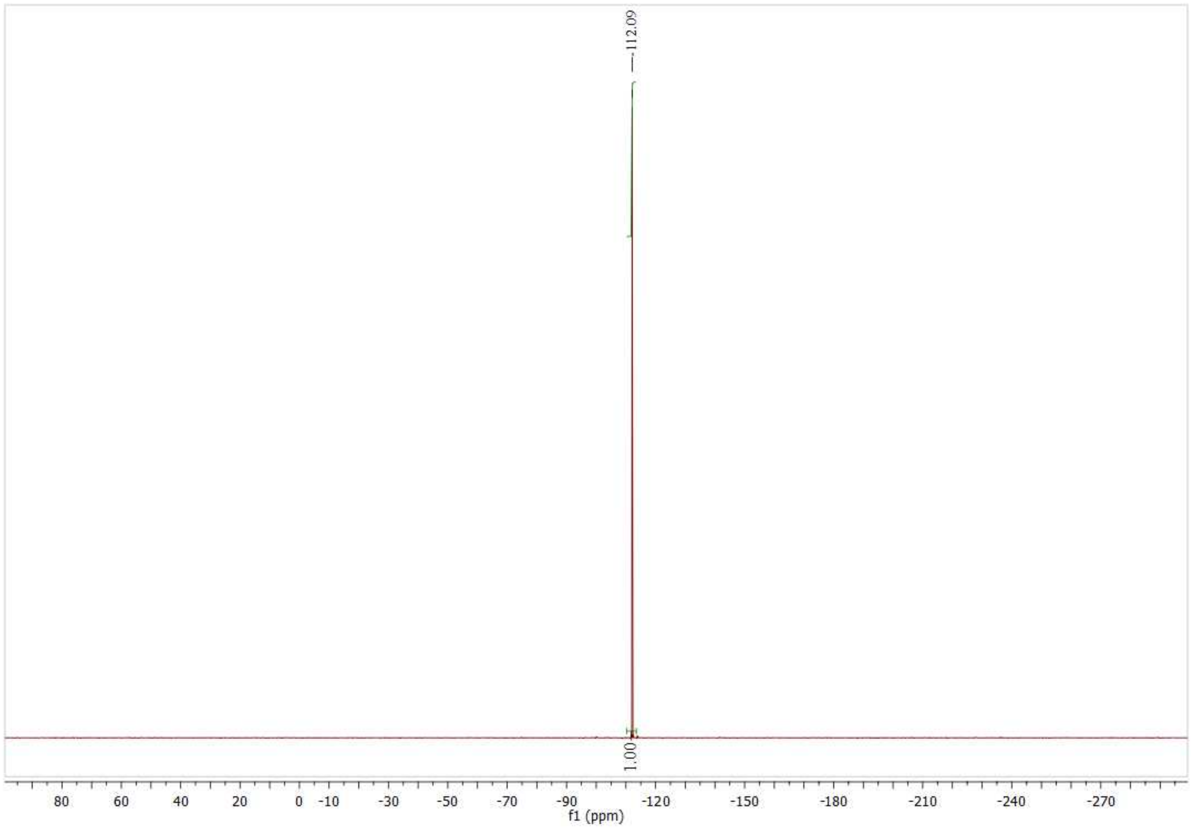

-112.09
1.00
80
60
40
20
0
-10
-30
-50
-70
-90
-120
-150
-180
-210
-240
-270
f1 (ppm)

Diethyl 2-(2-methylbenzylidene)malonate

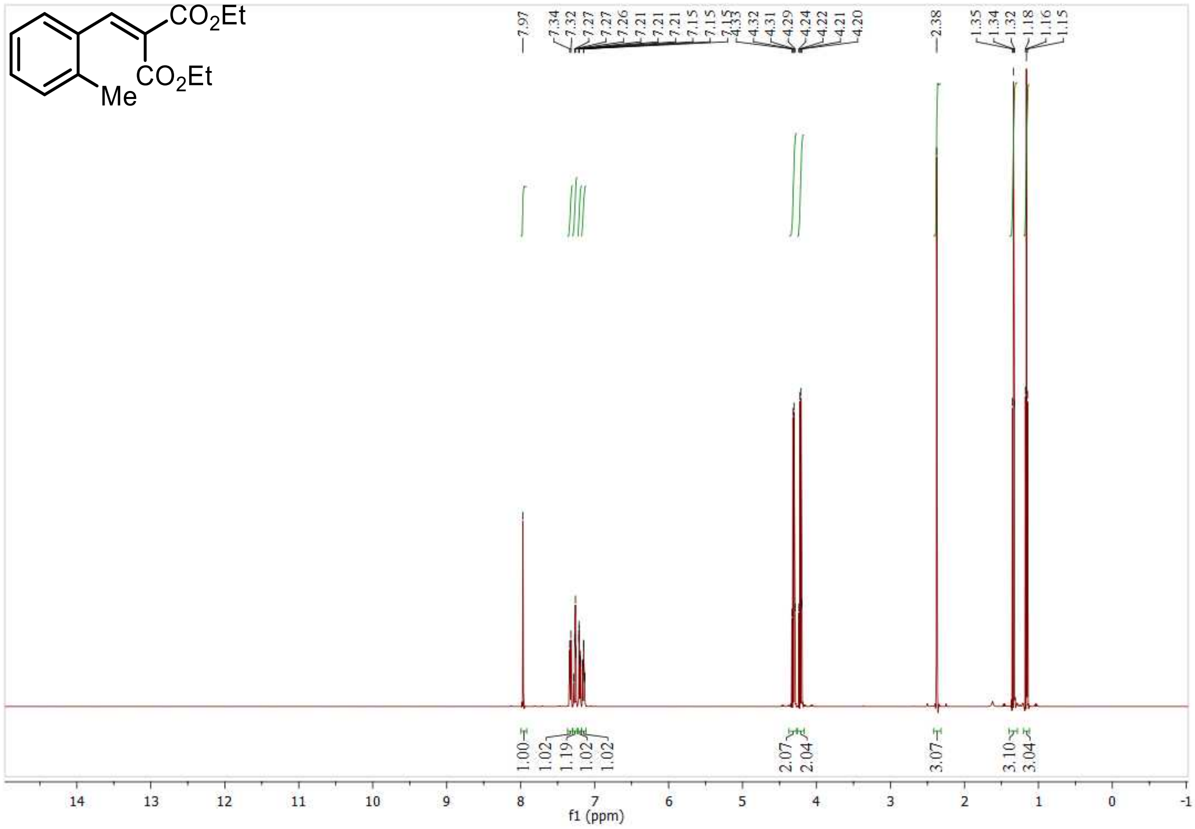

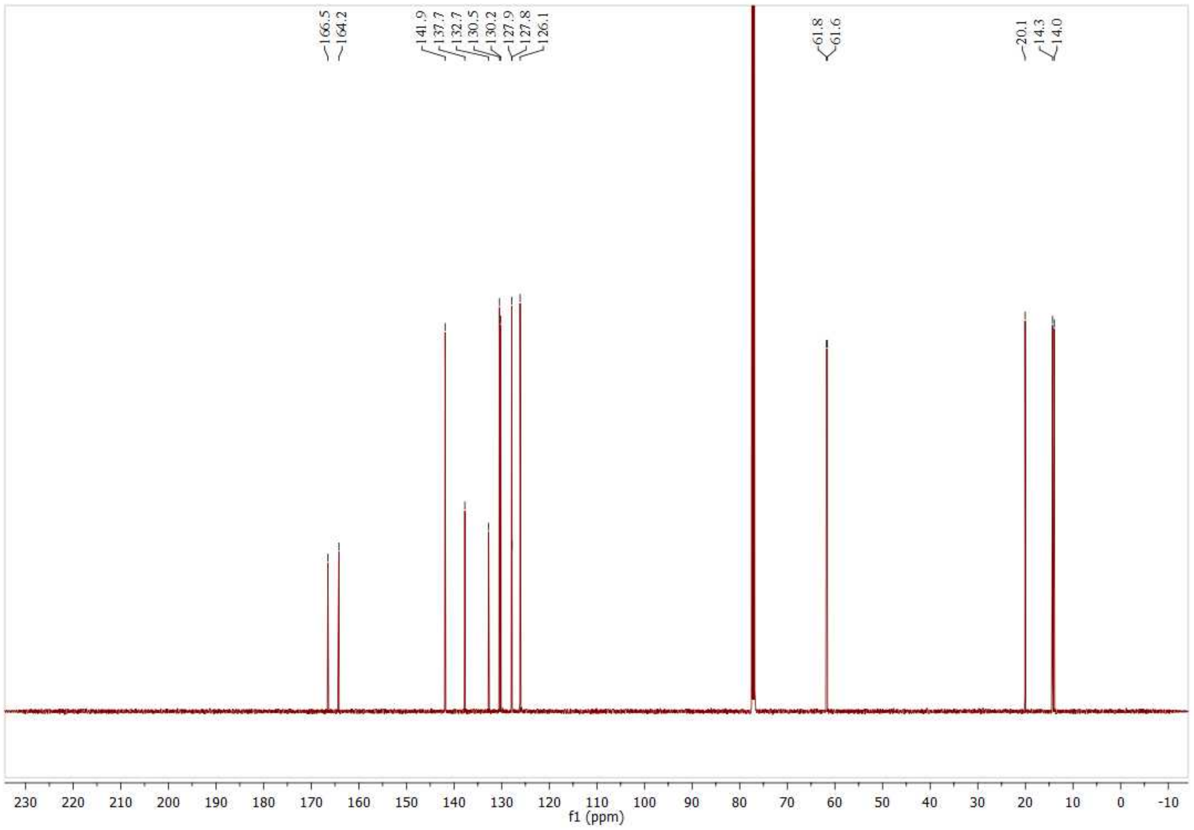

Diethyl 3-(3-methylbenzylidene)malonate

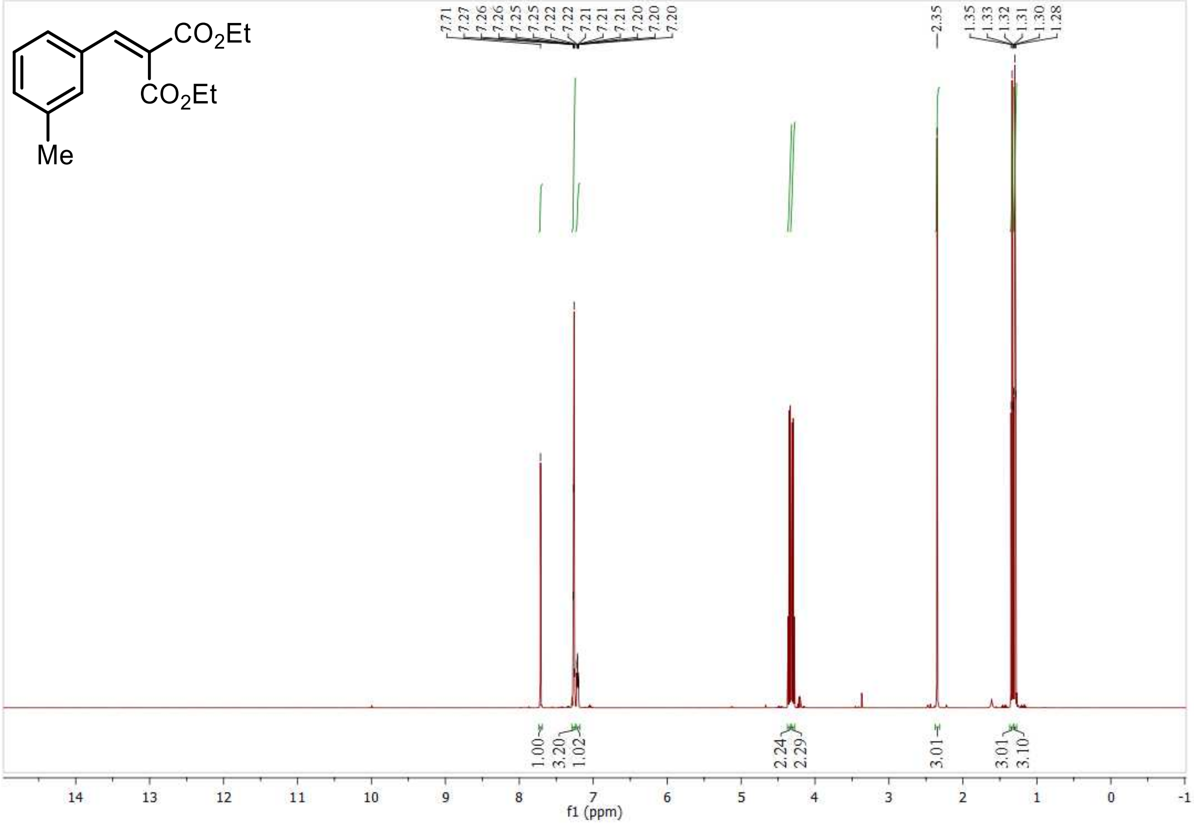

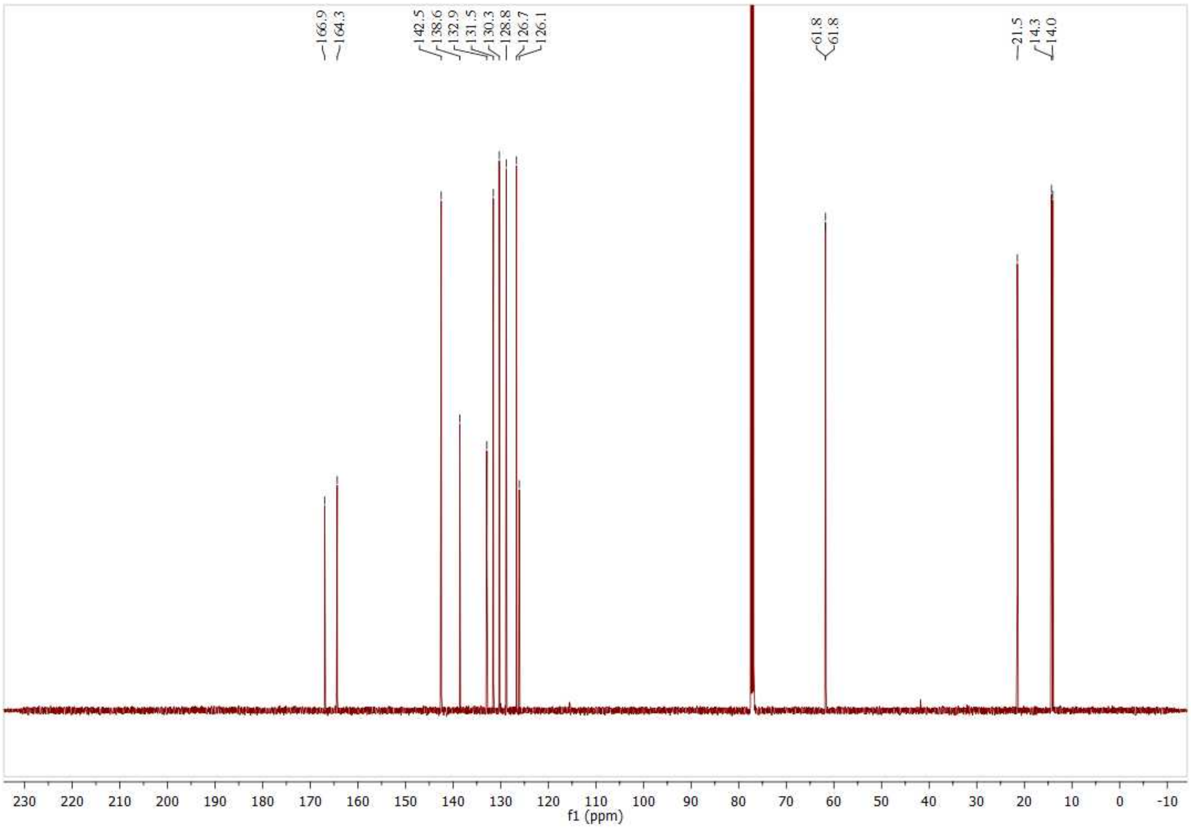

Diethyl 2-(pyridin-3-ylmethylene)malonate

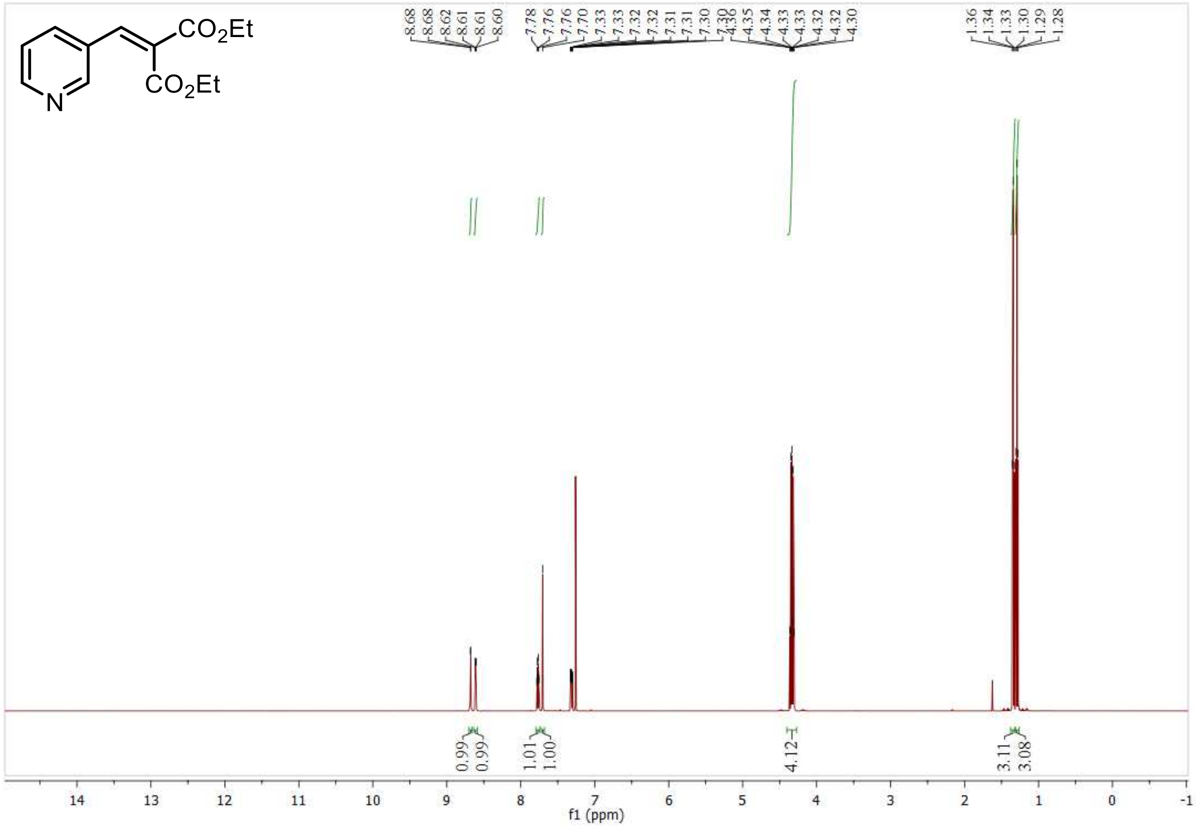

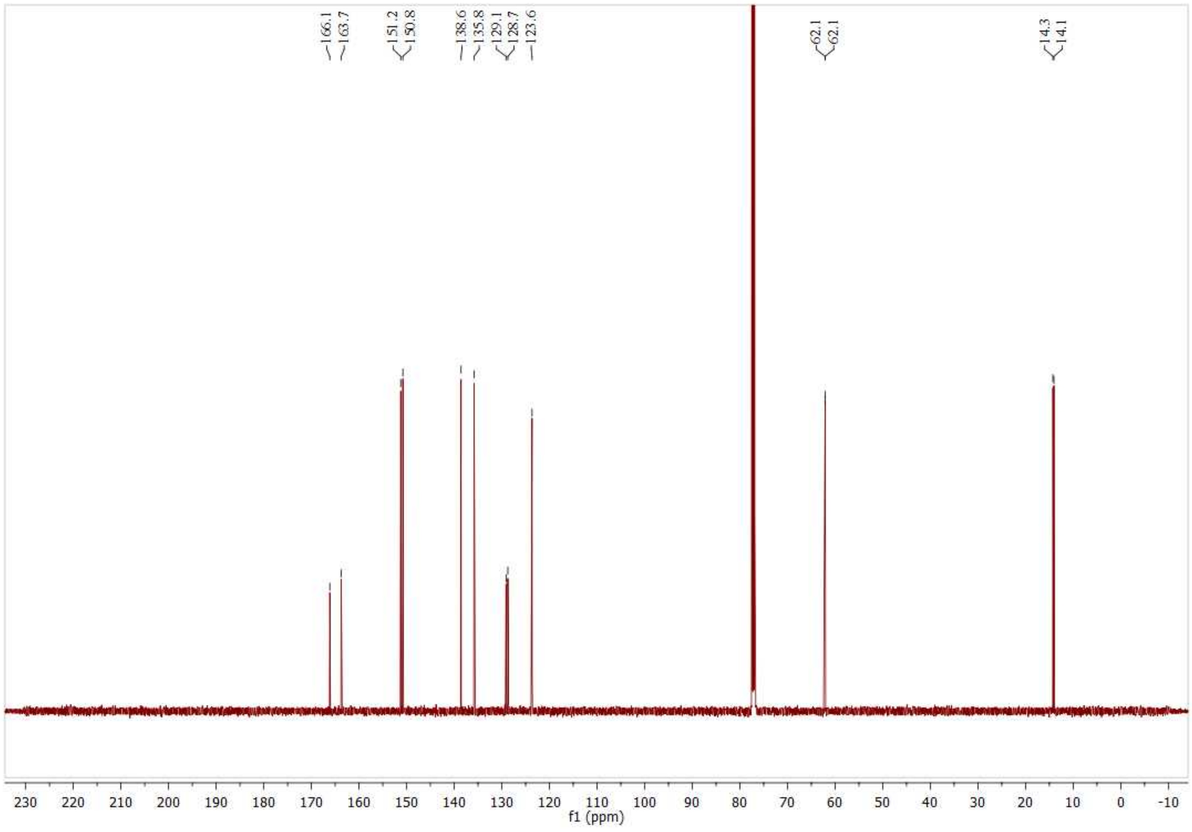

Diethyl 2-(naphthalen-2-ylmethylene)malonate

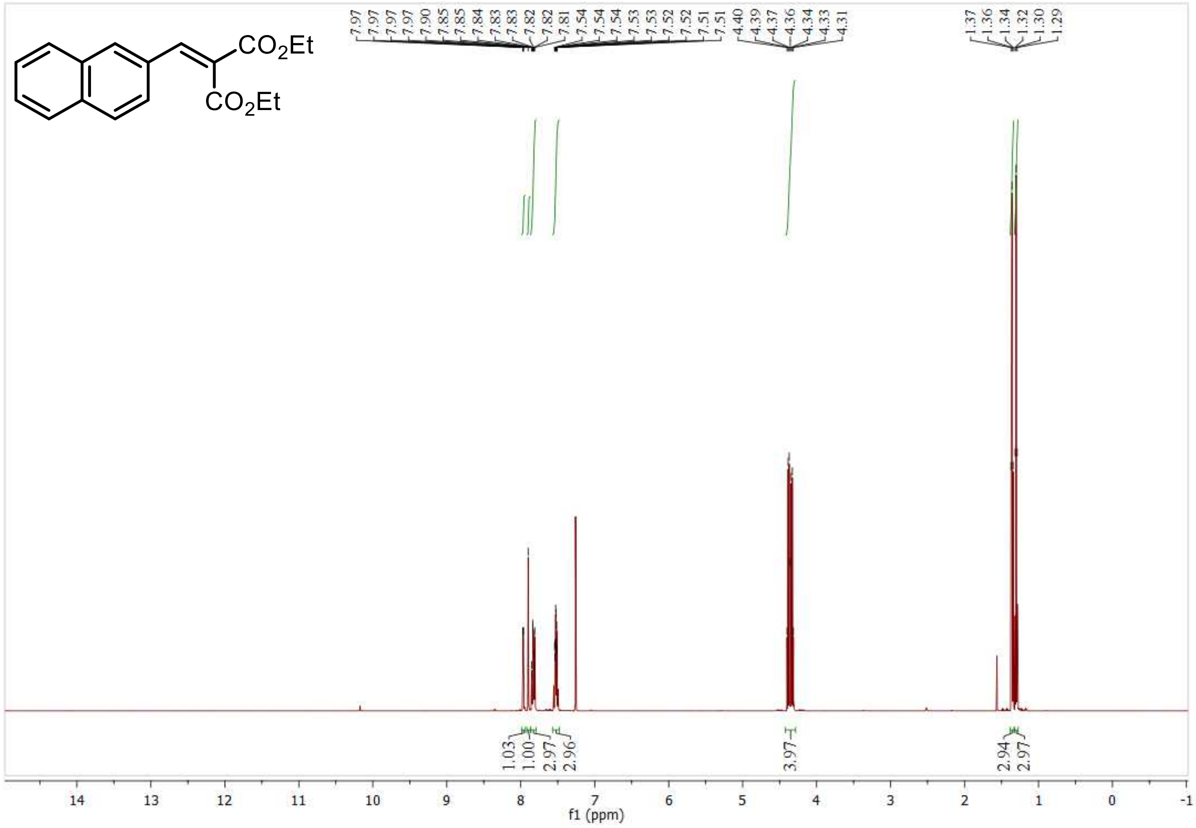

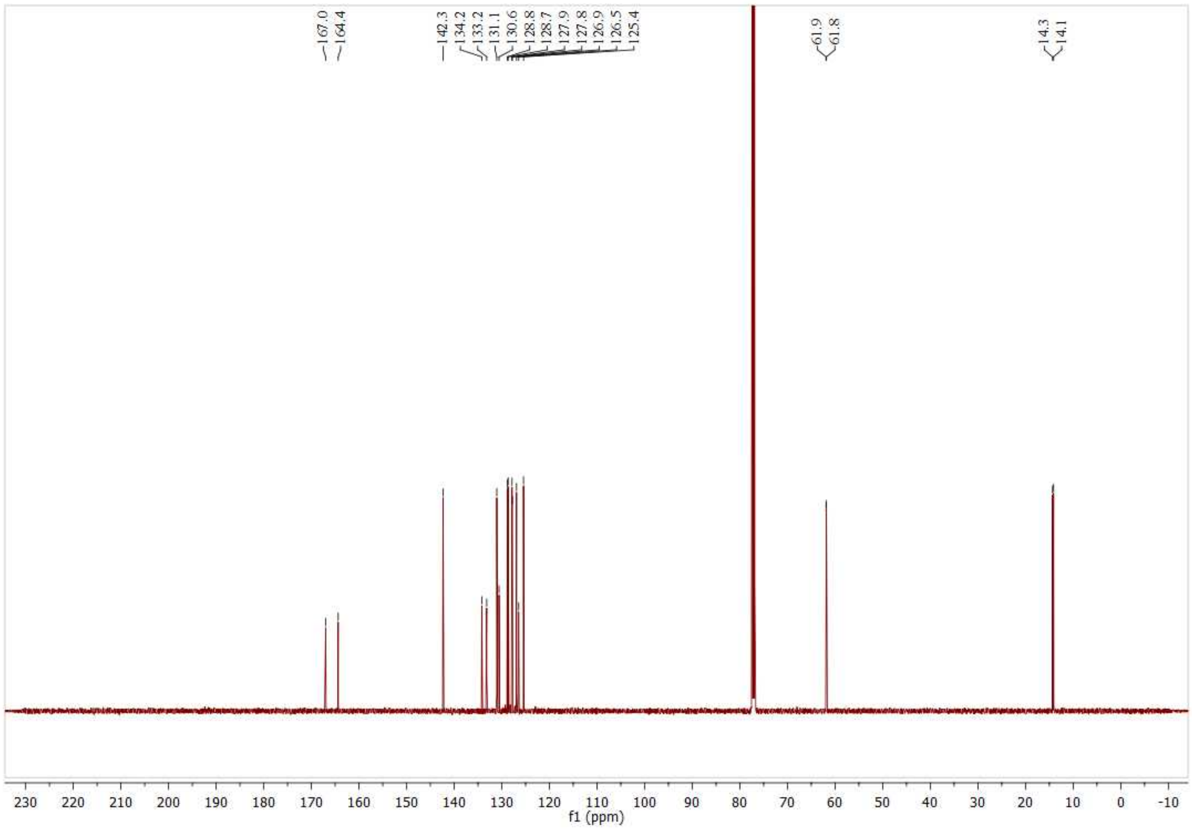

Diethyl 2-(naphthalen-1-ylmethylene)malonate

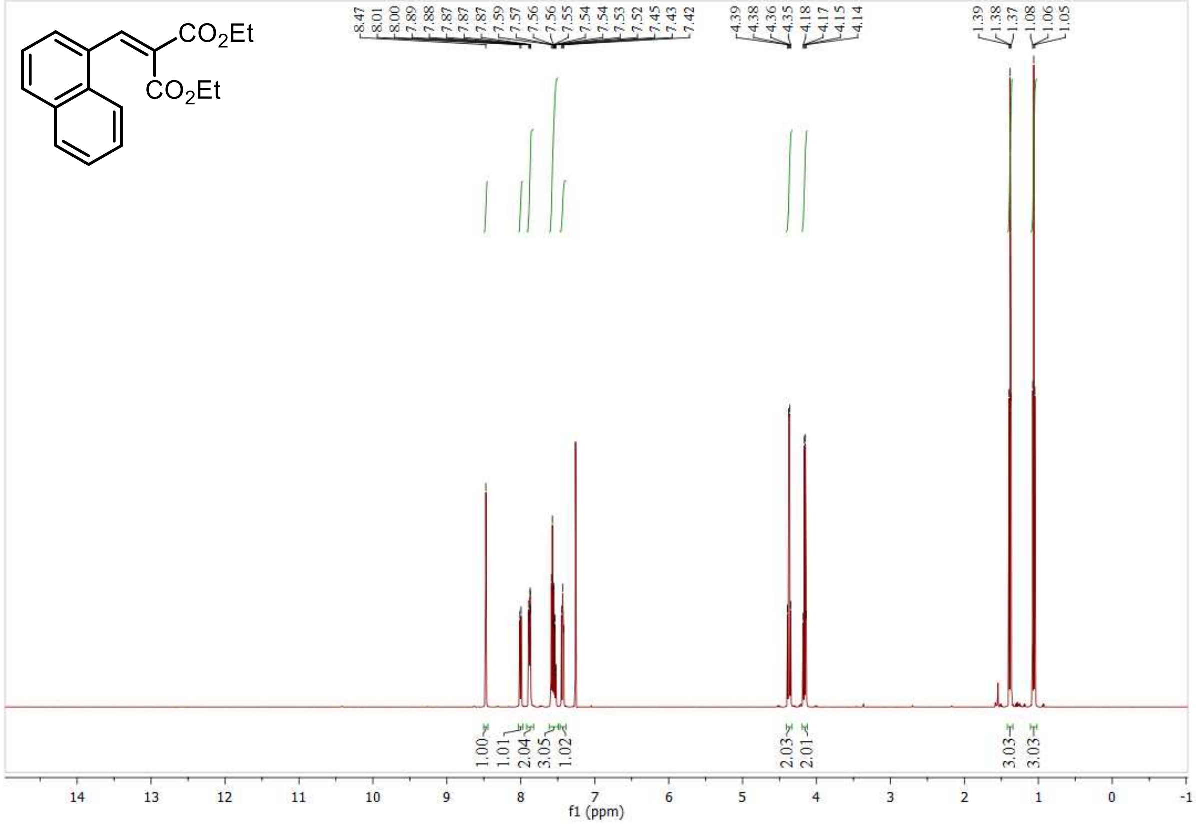

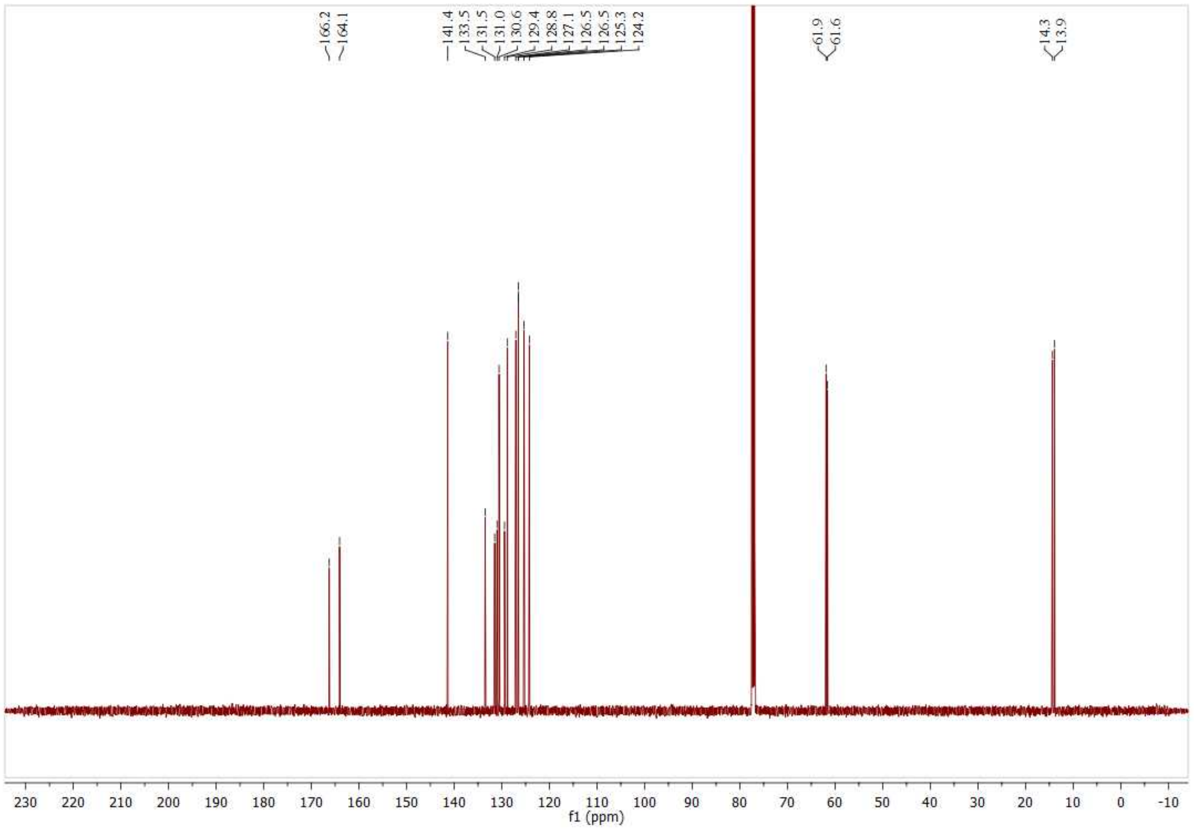

Diethyl 2-(cyclopropylmethylene)malonate

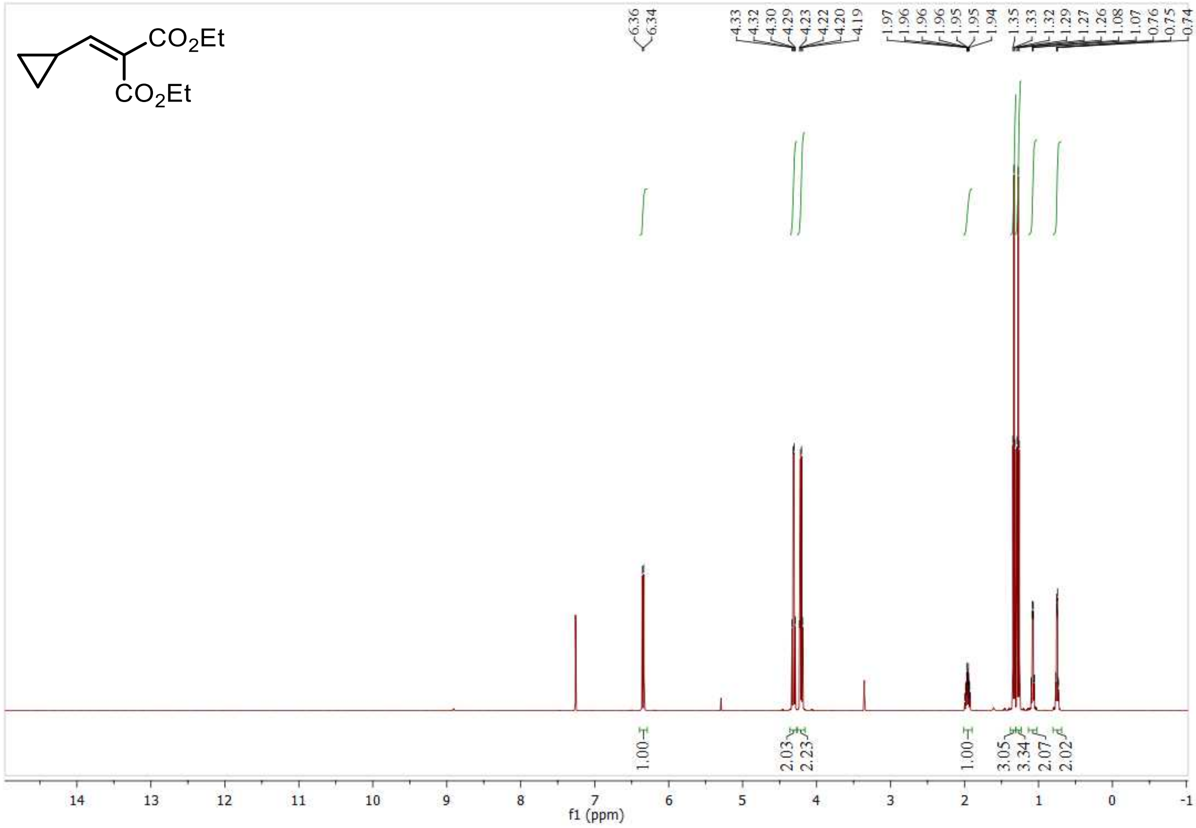

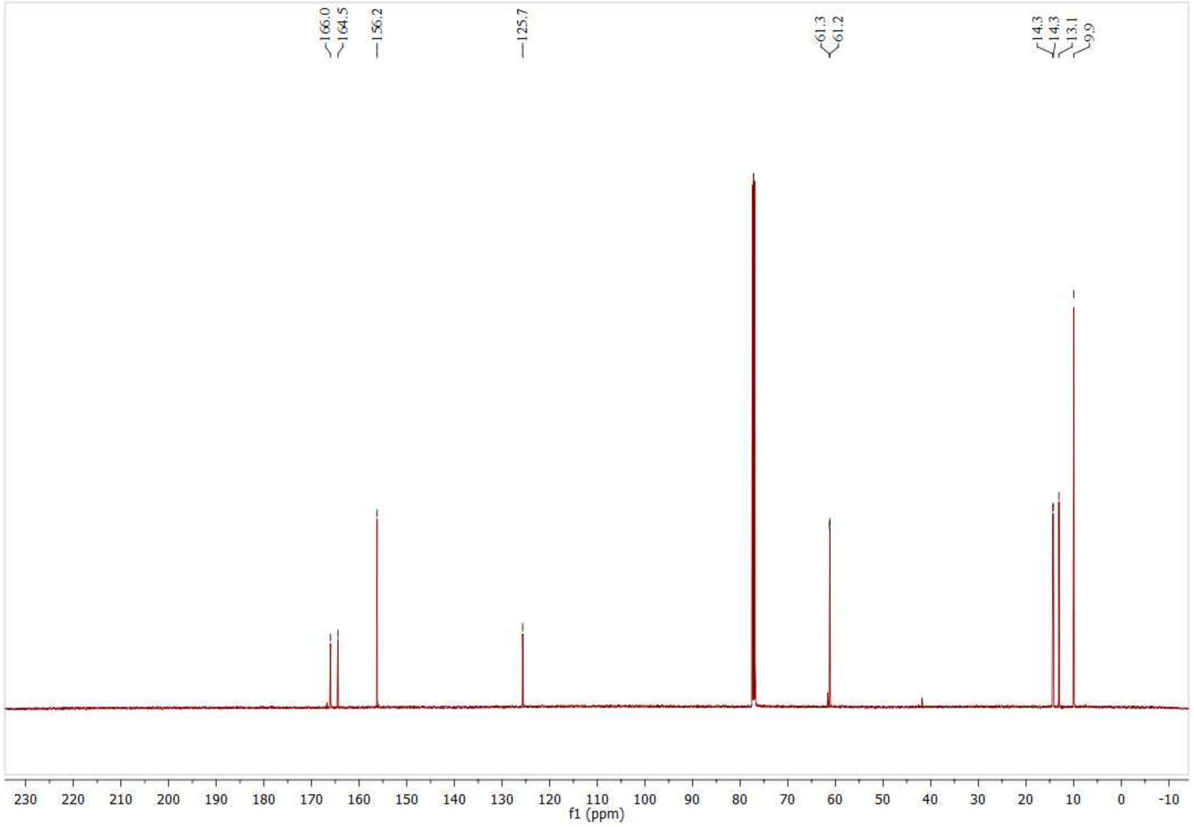

Diethyl 2-(2,6-dimethylhept-5-en-1-ylidene)malonate

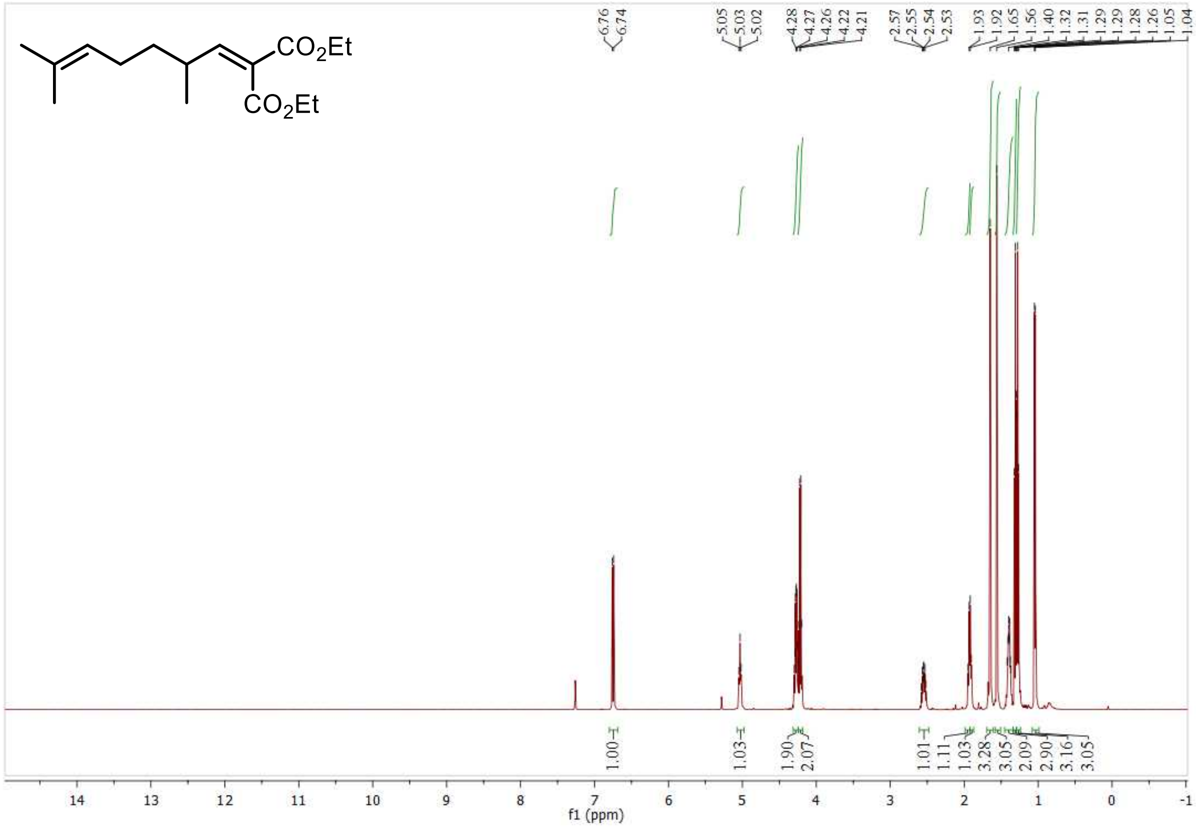

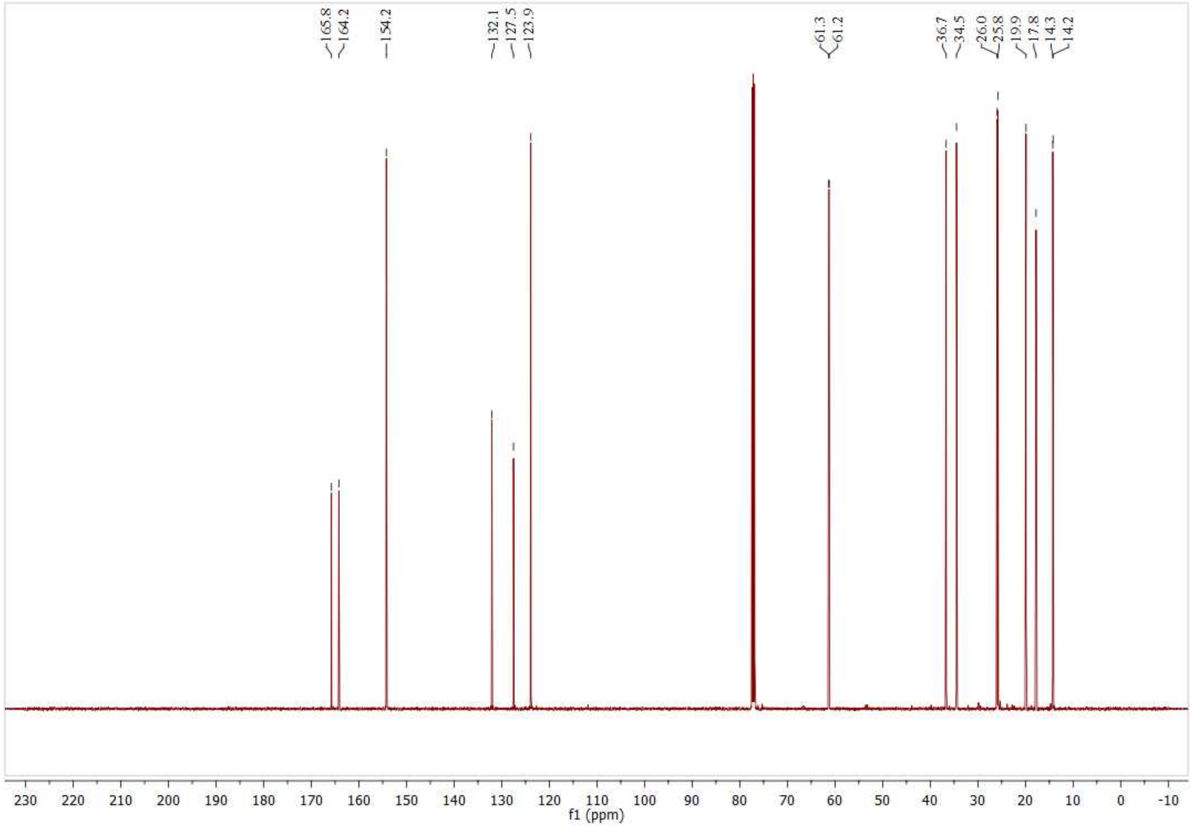

Dimethyl 2-(4-fluorobenzylidene)malonate

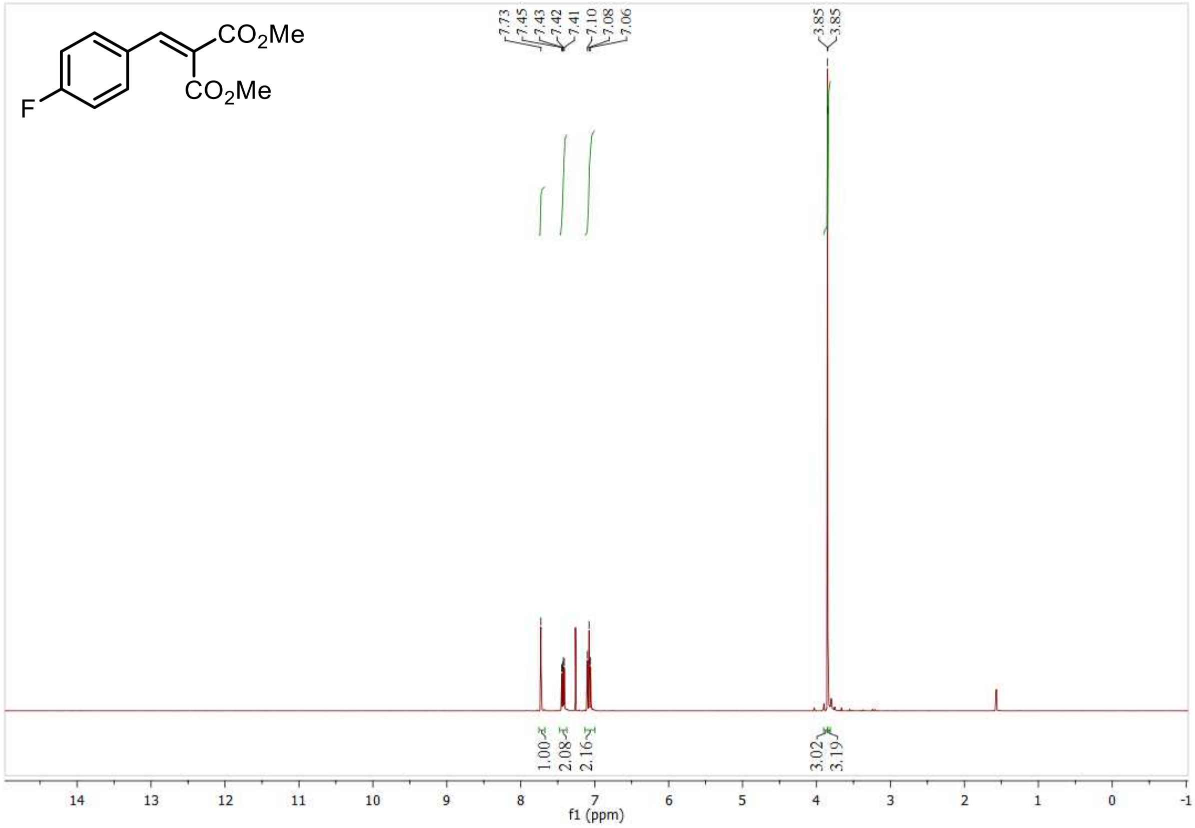

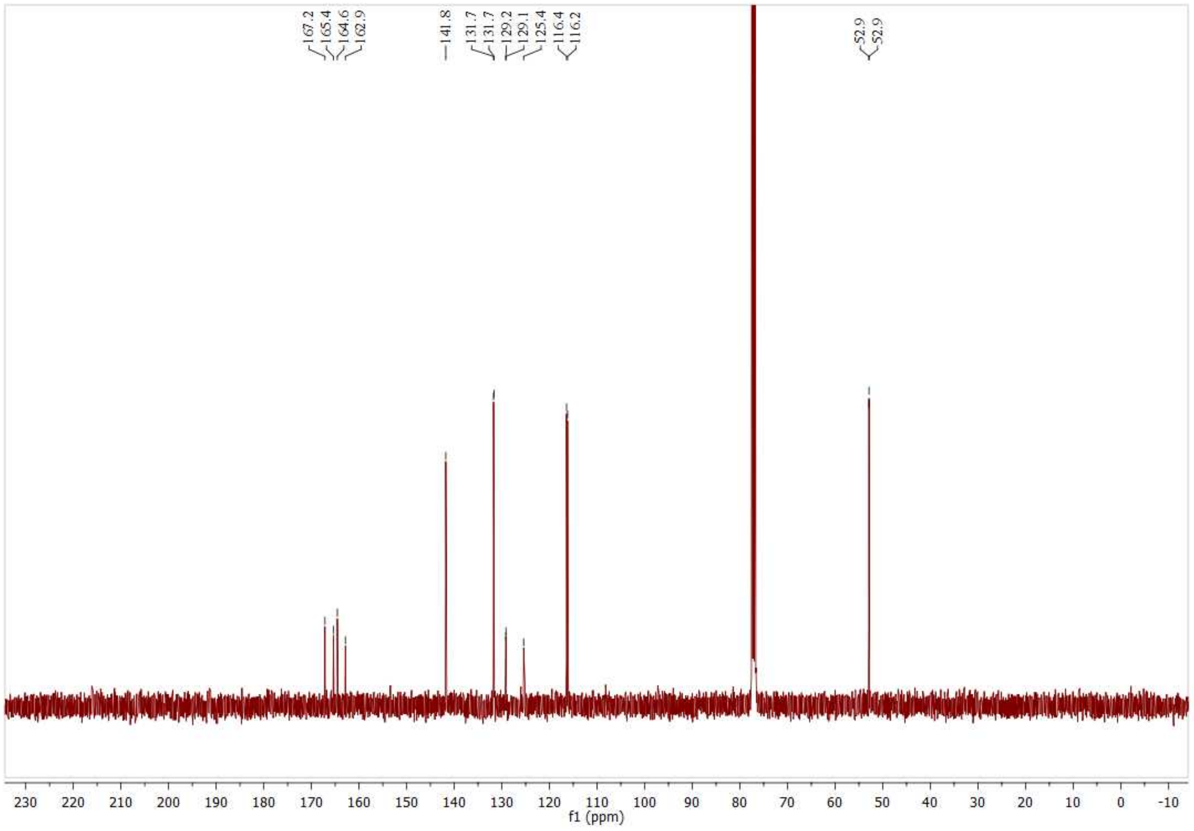

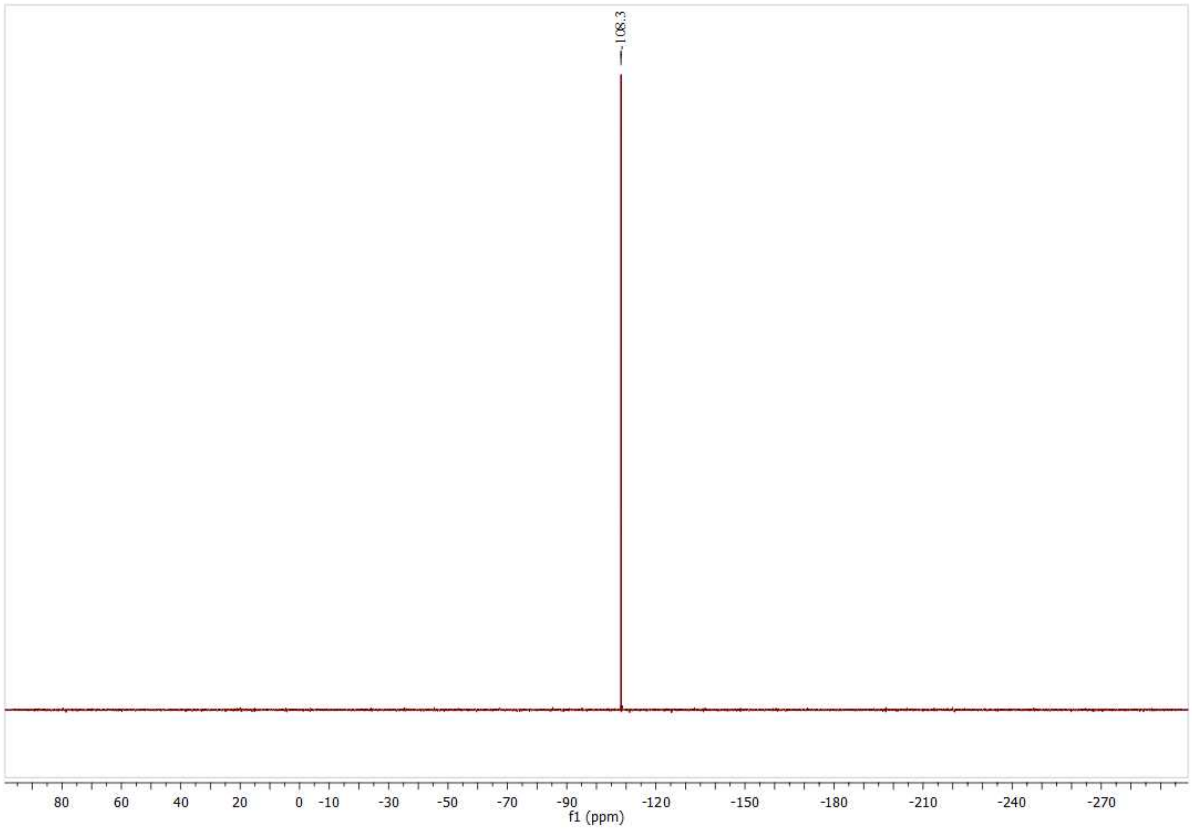
-108.3
80
60
40
20
0
-10
-30
-50
-70
-90
-120
-150
-180
-210
-240
-270
f1 (ppm)

Diisopropyl 2-(4-fluorobenzylidene)malonate

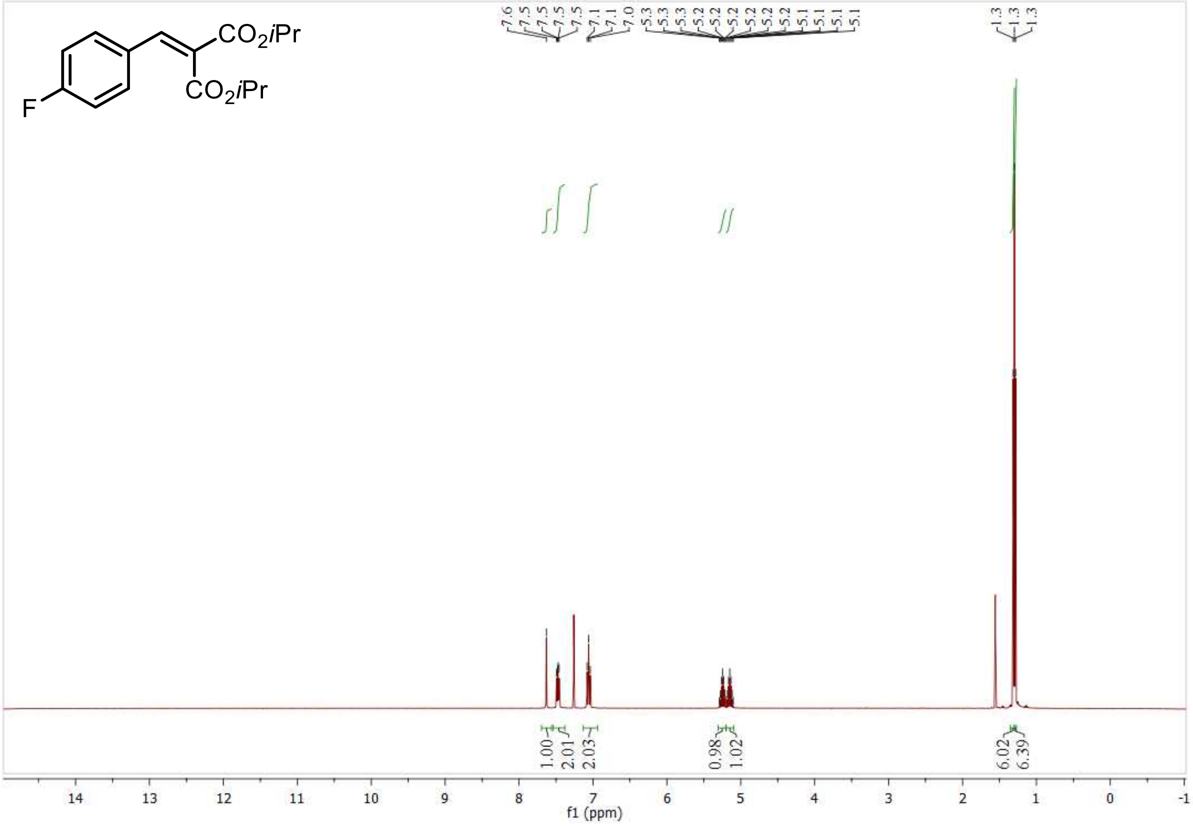

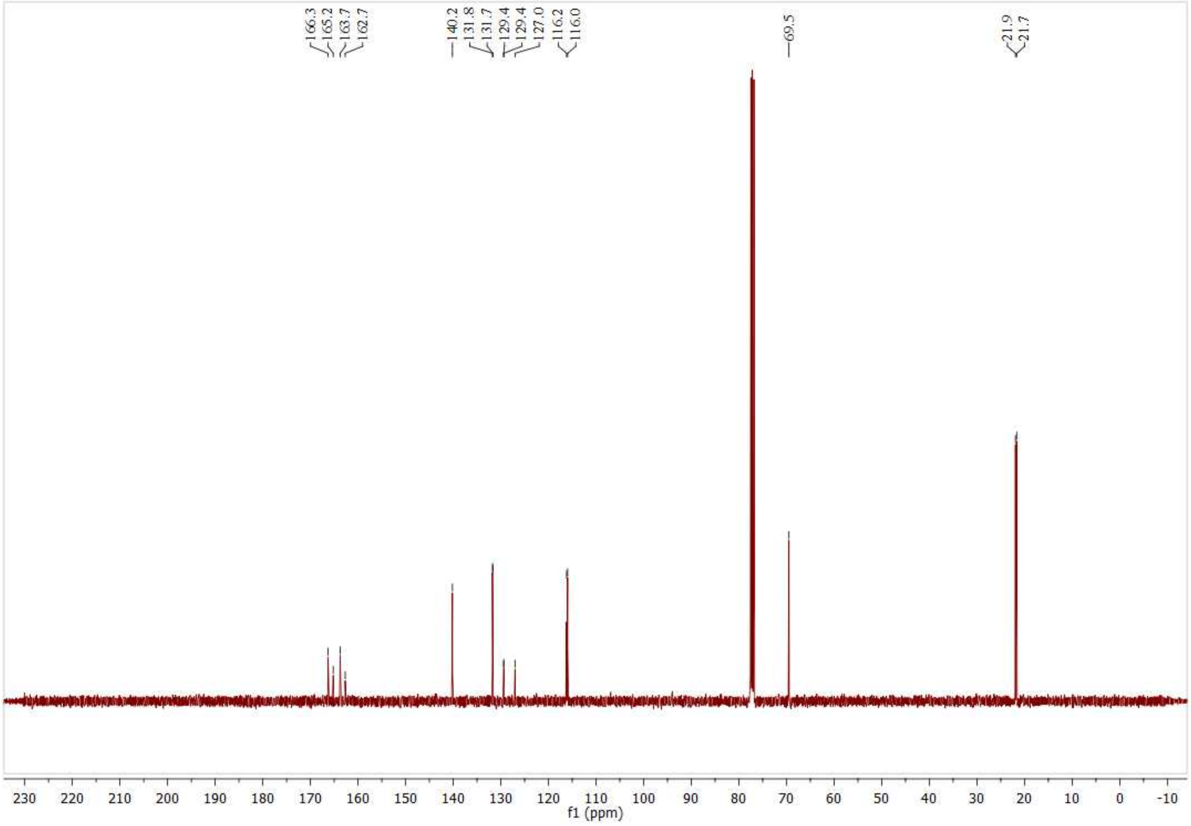

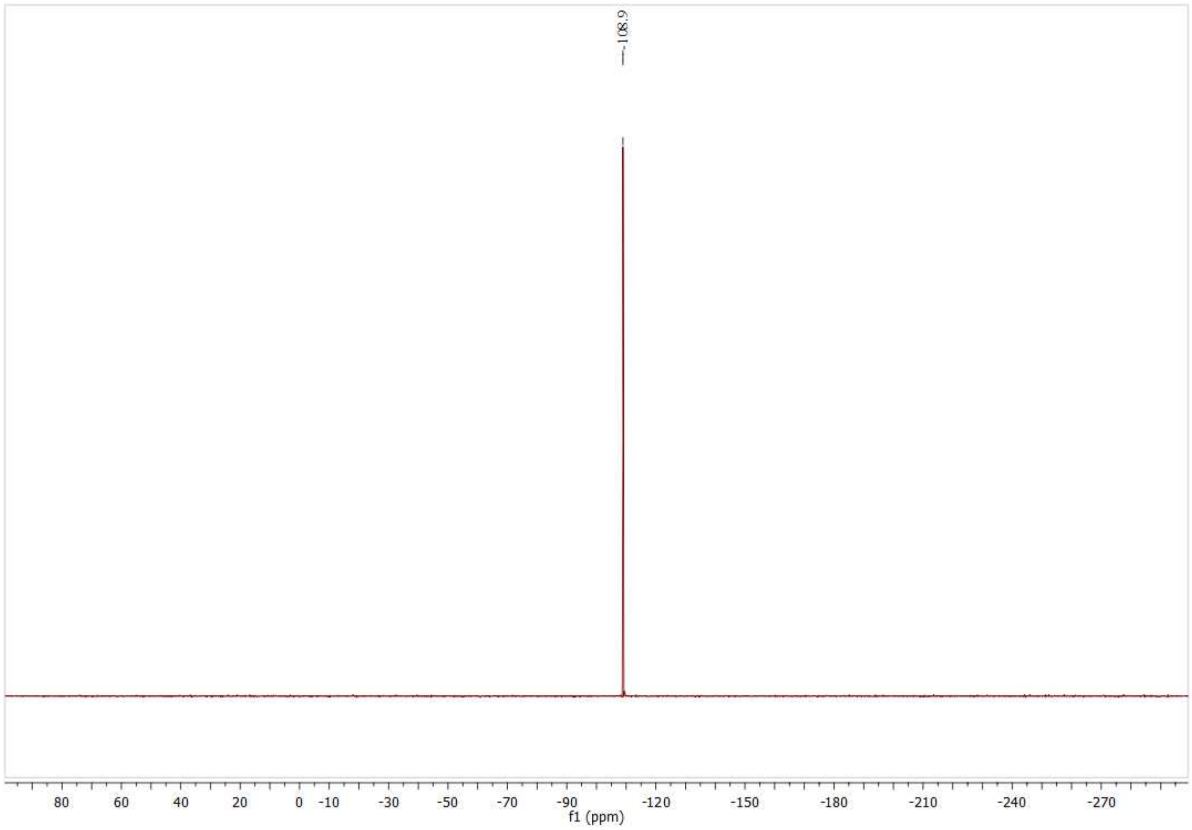
-108.9
80
60
40
20
0
-10
-30
-50
-70
-90
-120
-150
-180
-210
-240
-270
f1 (ppm)

Di-tert-butyl 2-(4-fluorobenzylidene)malonate

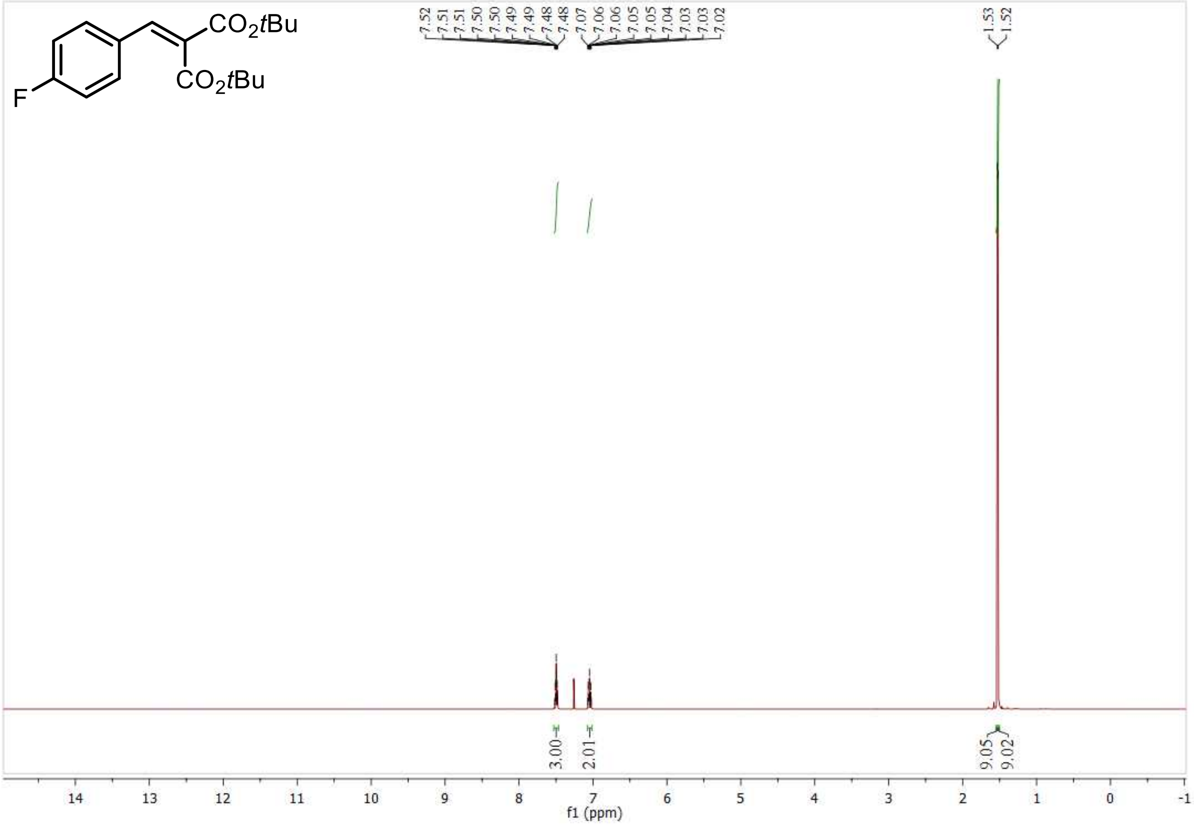

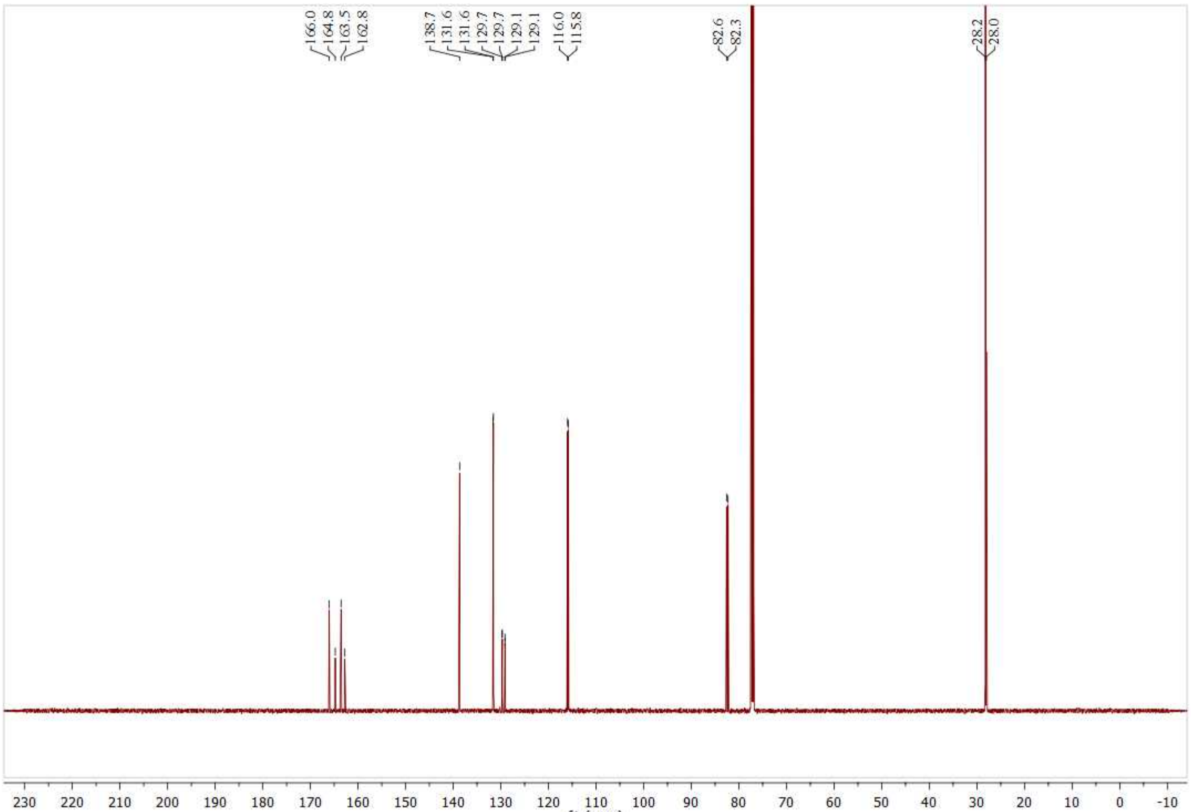

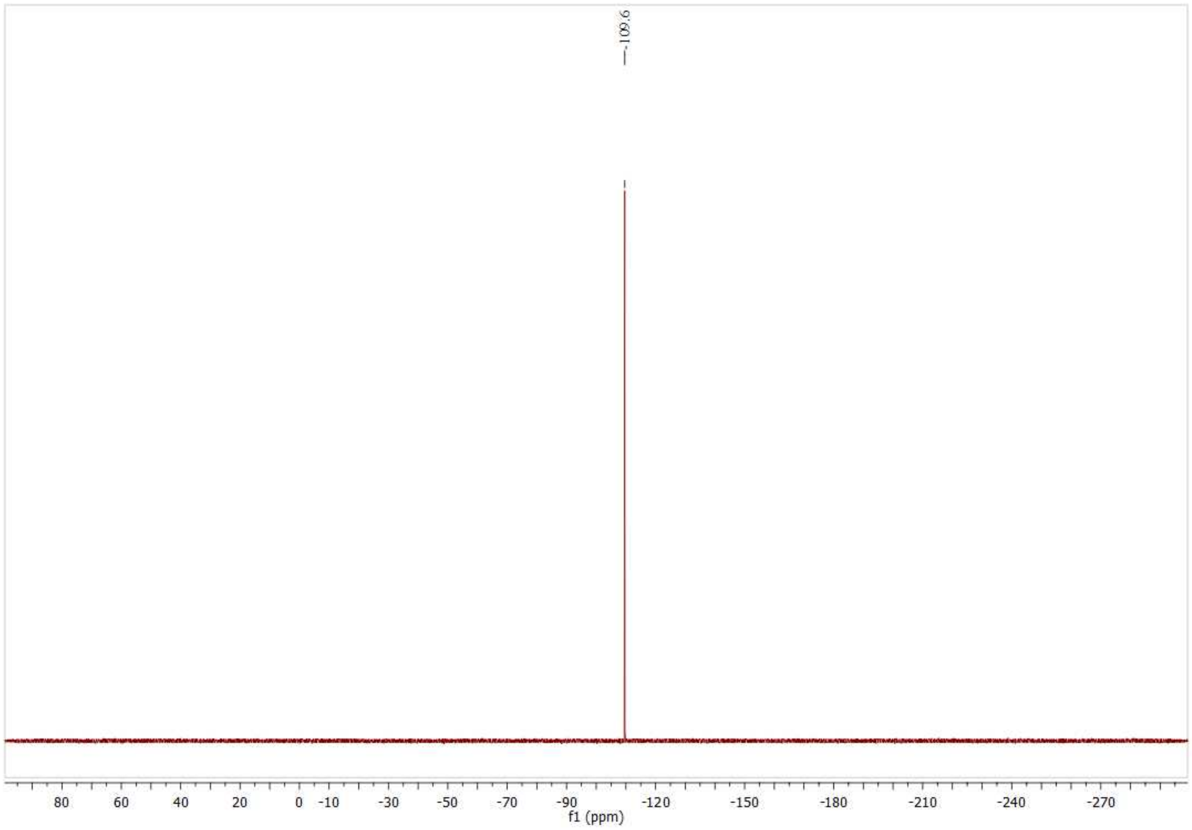
-109.6
80
60
40
20
0
-10
-30
-50
-70
-90
-120
-150
-180
-210
-240
-270
f1 (ppm)

Diallyl 2-(4-fluorobenzylidene)malonate

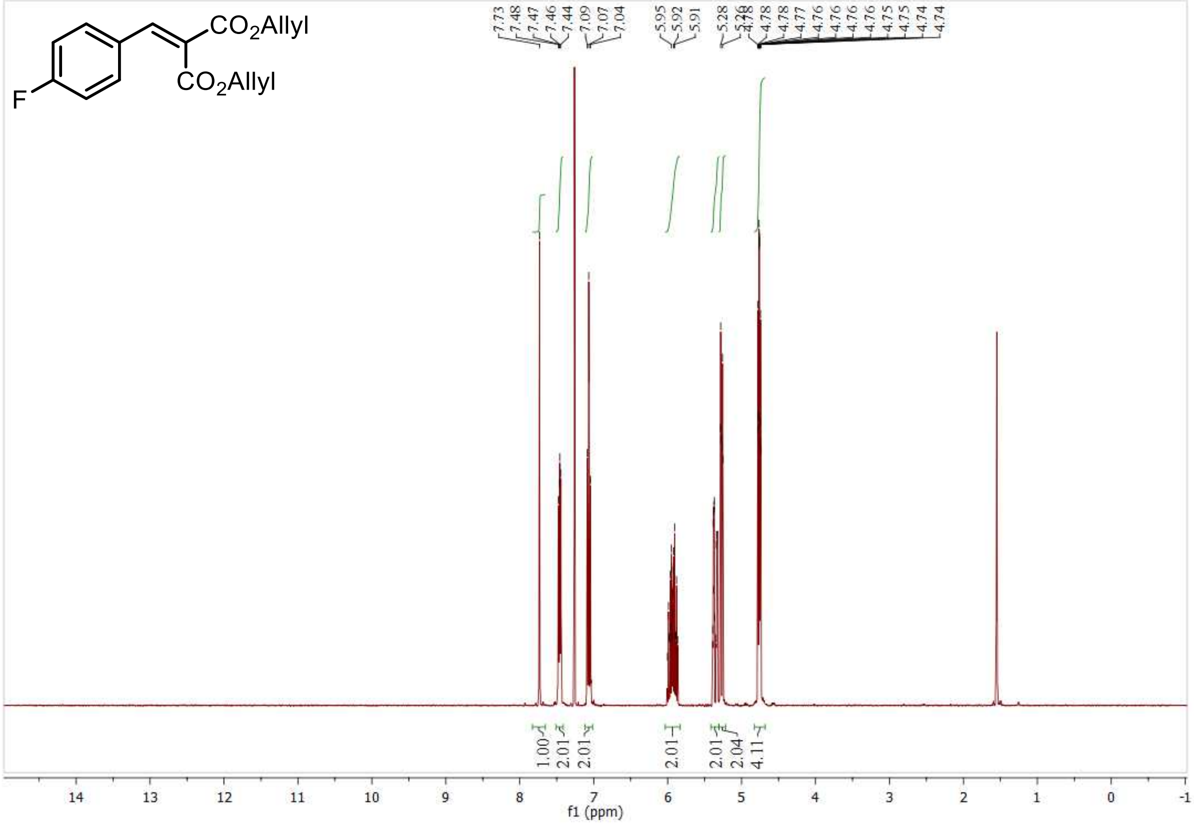

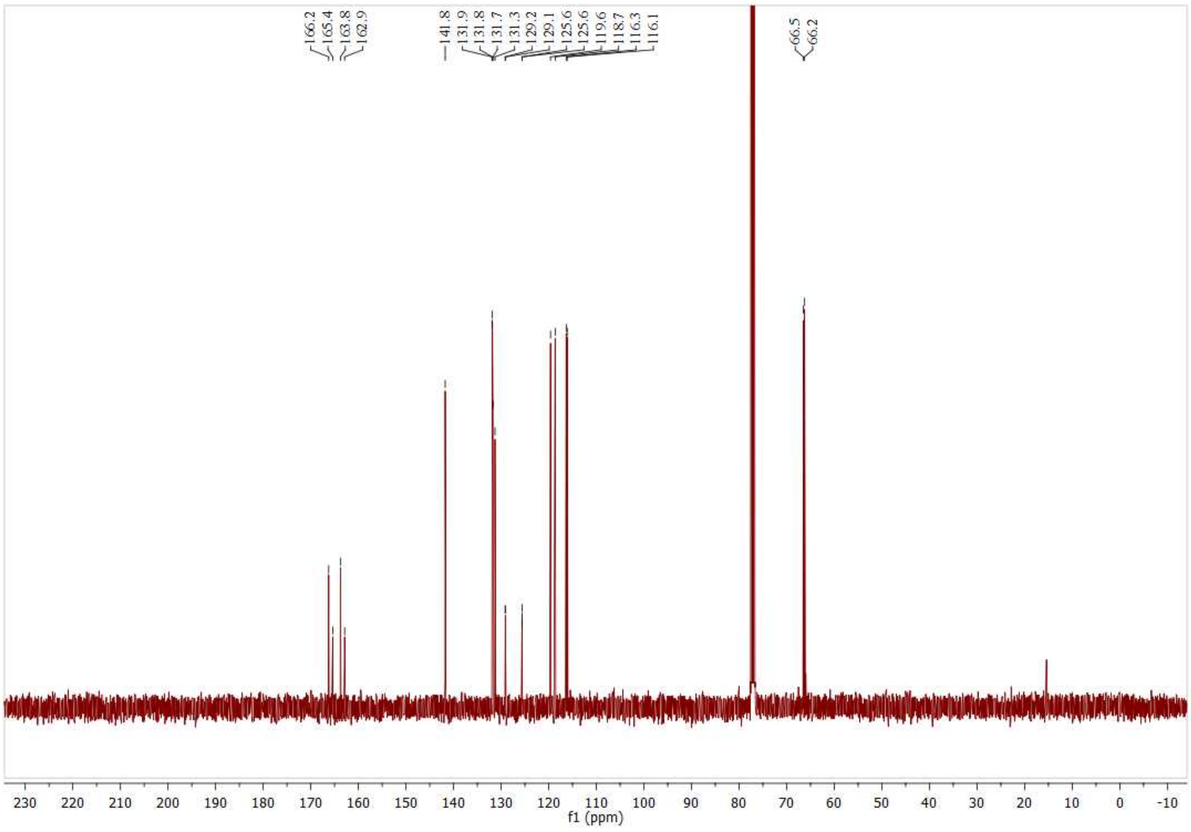

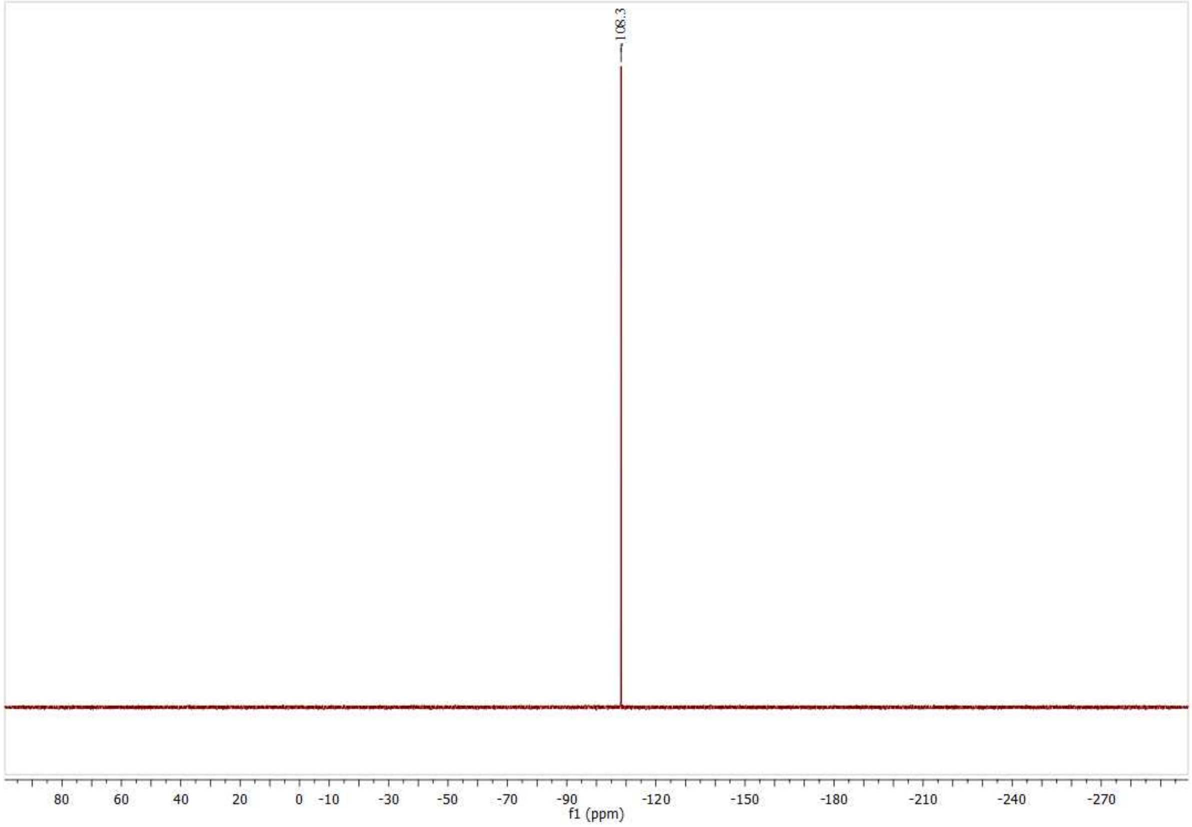
-108.3
80
60
40
20
0
-10
-30
-50
-70
-90
-120
-150
-180
-210
-240
-270
f1 (ppm)

Dibenzyl 2-(4-fluorobenzylidene)malonate

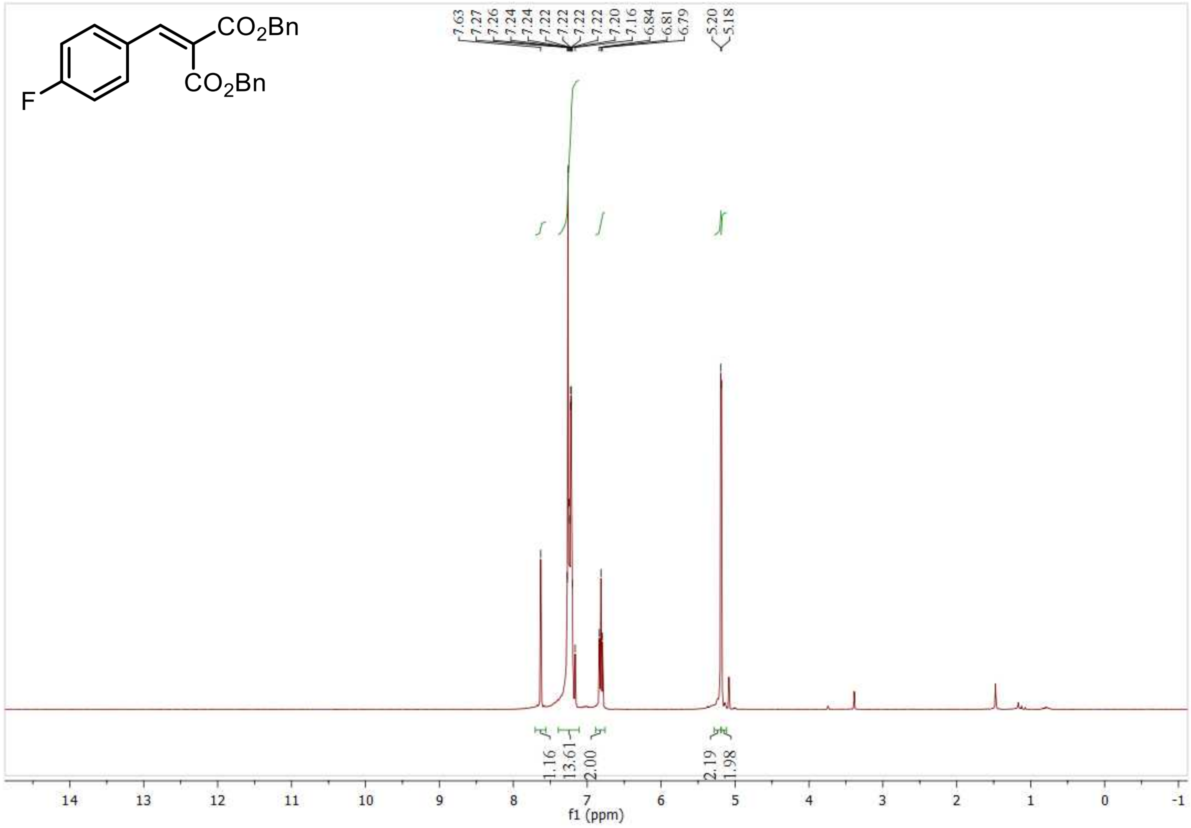

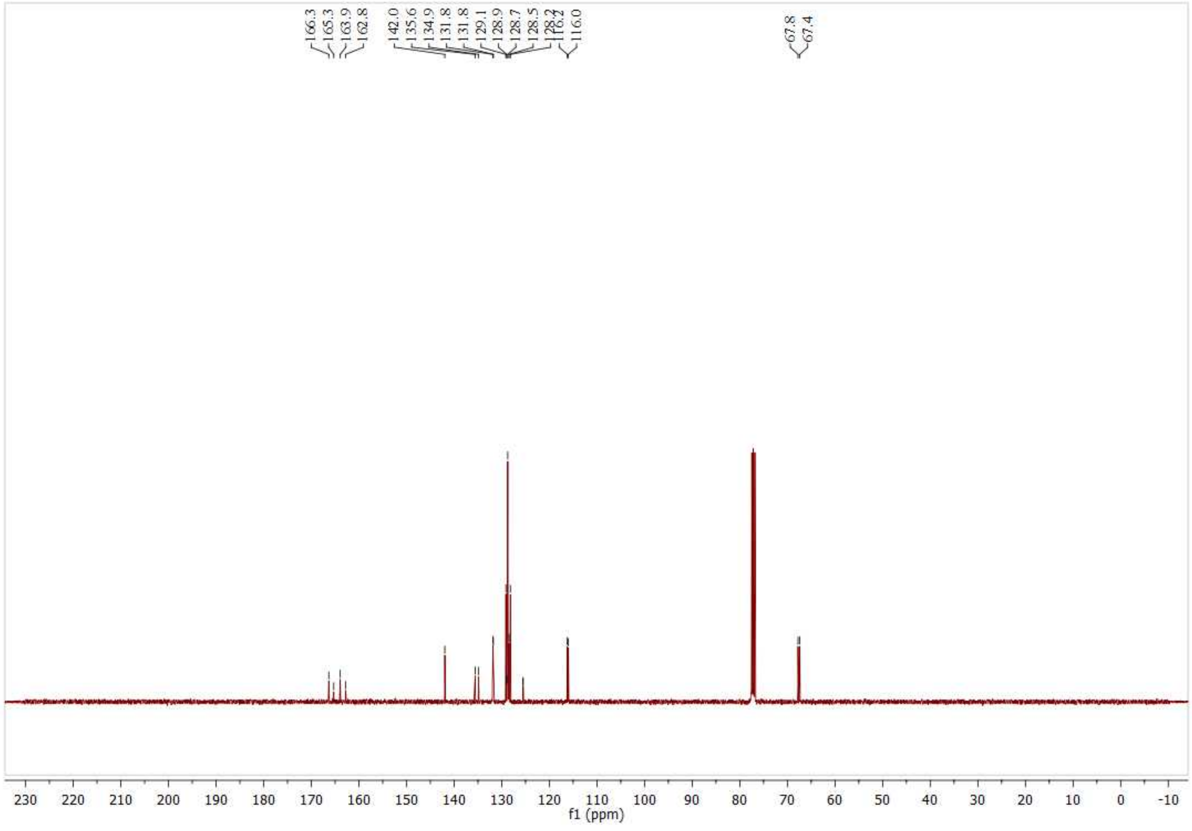

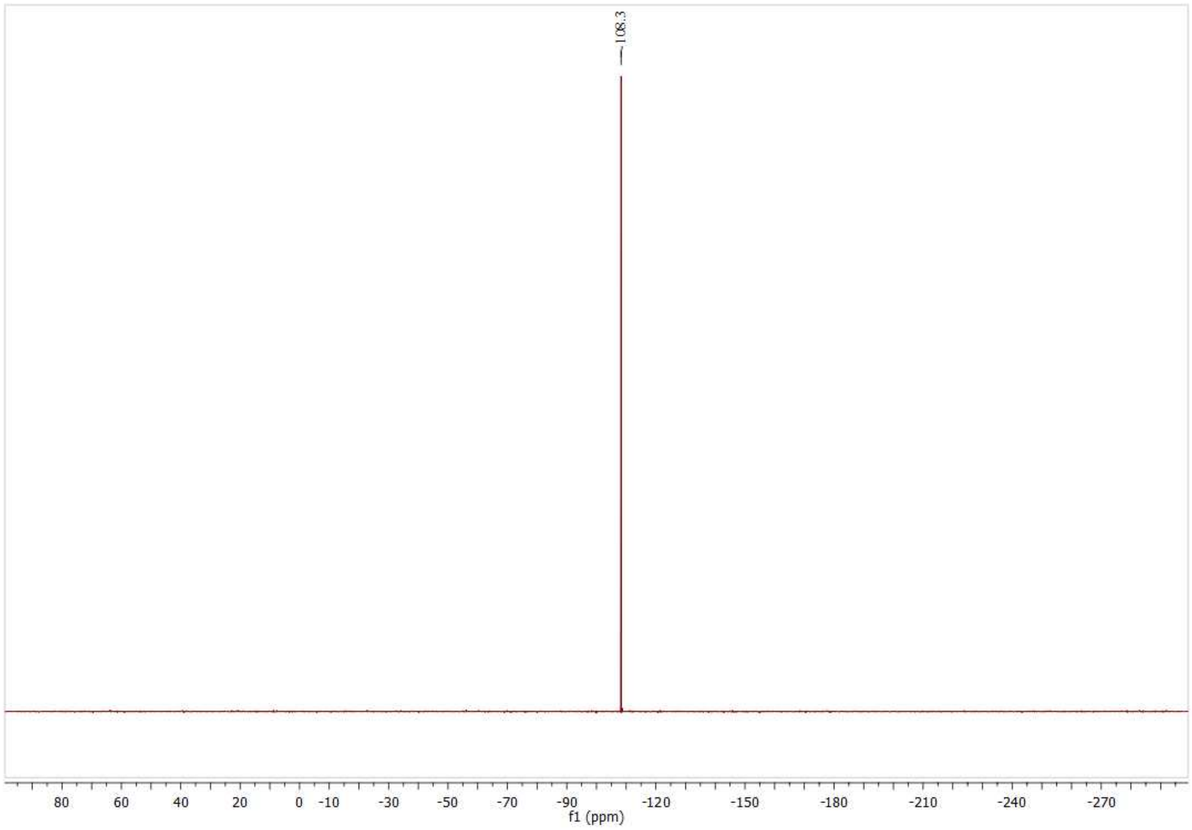
-108.3
80
60
40
20
0
-10
-30
-50
-70
-90
-120
-150
-180
-210
-240
-270
f1 (ppm)

Ethyl (E)-2-cyano-3-(4-fluorophenyl)acrylate

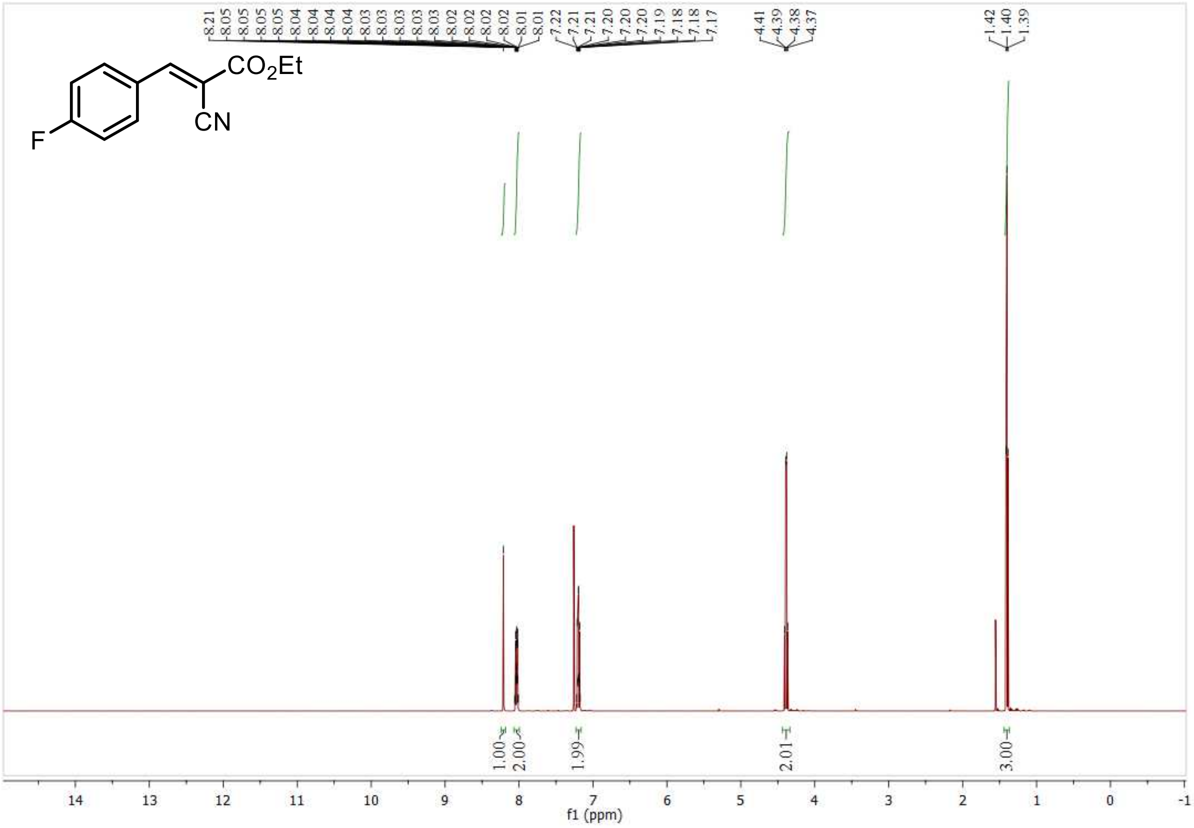

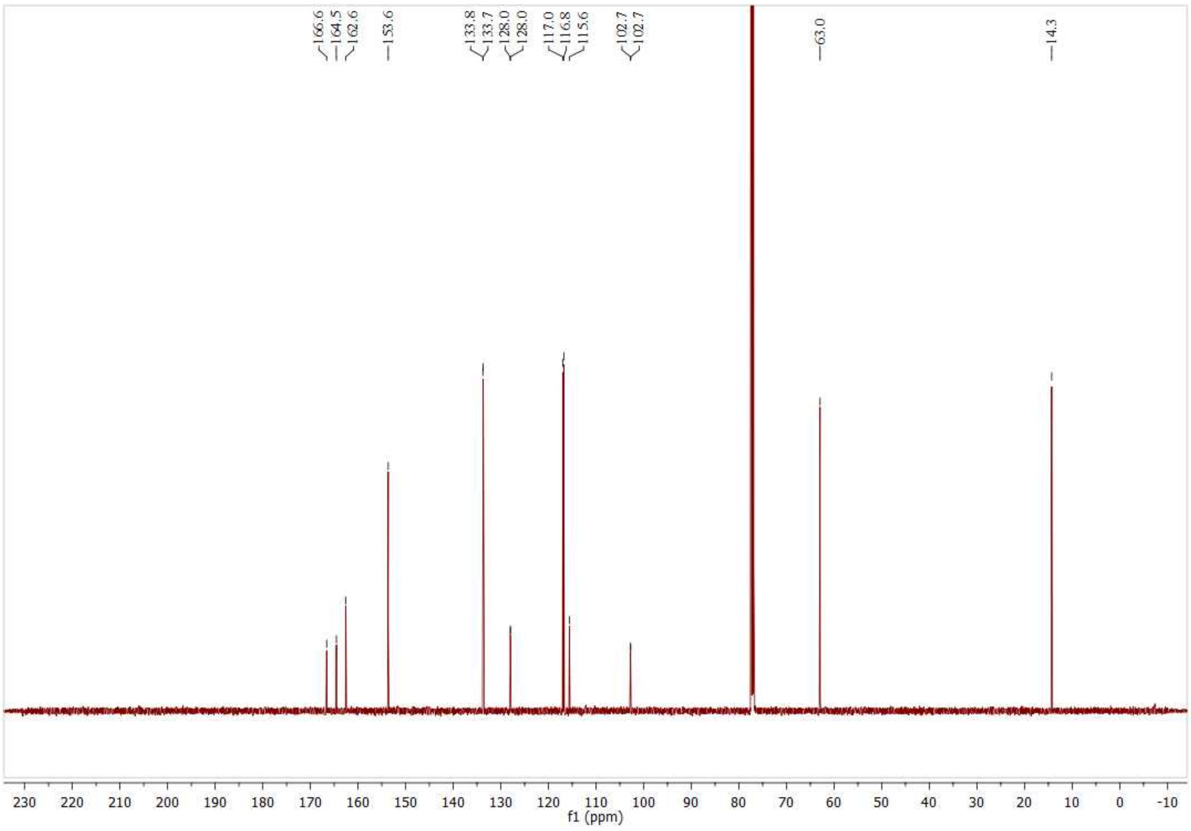

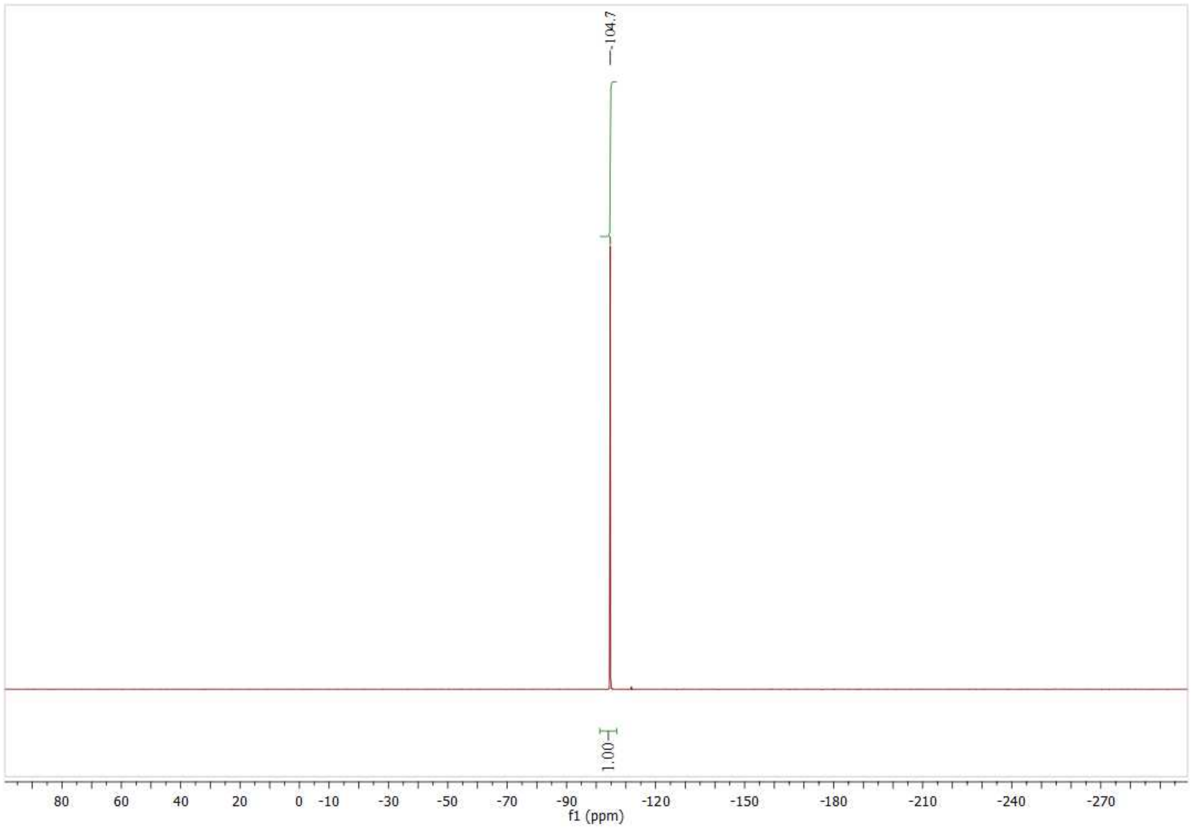

-104.7
1.00
80
60
40
20
0
-10
-30
-50
-70
-90
-120
-150
-180
-210
-240
-270
f1 (ppm)

5-(4-Fluorobenzylidene)-1,3-dimethylpyrimidine-2,4,6(1H,3H,5H)-trione

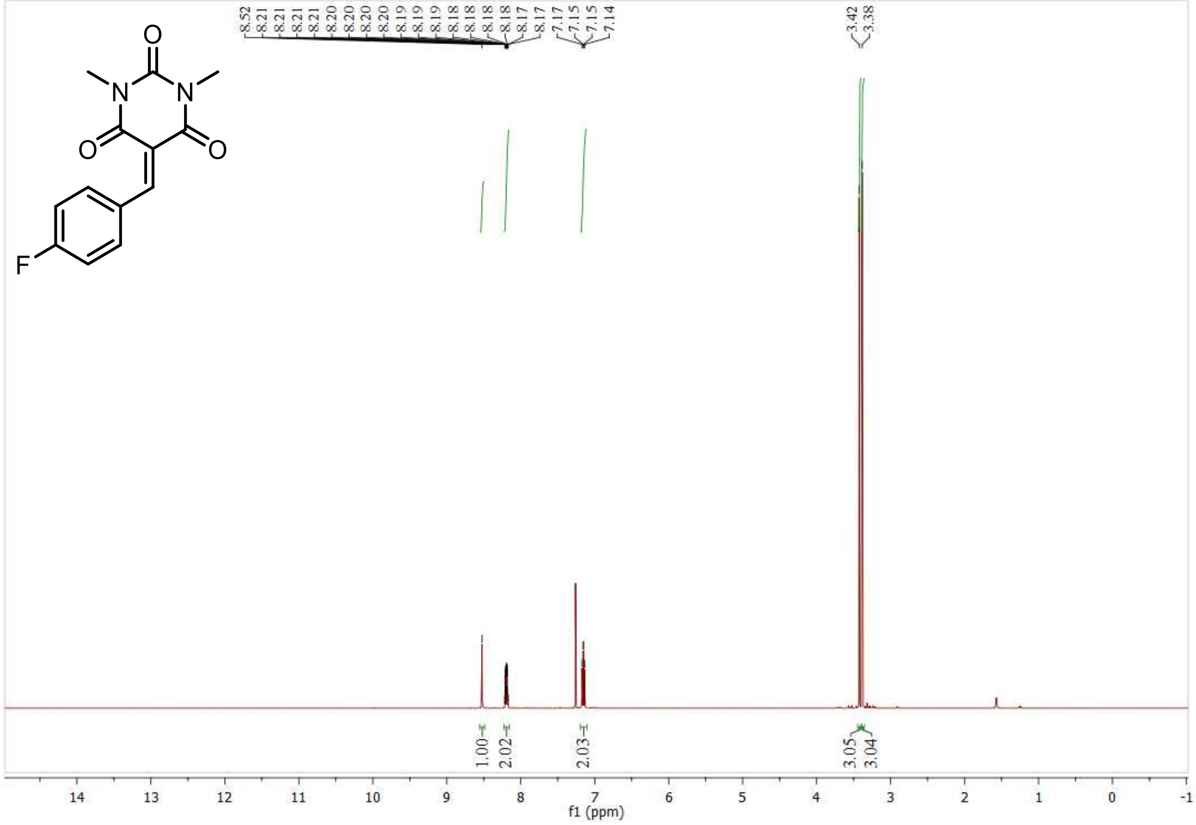

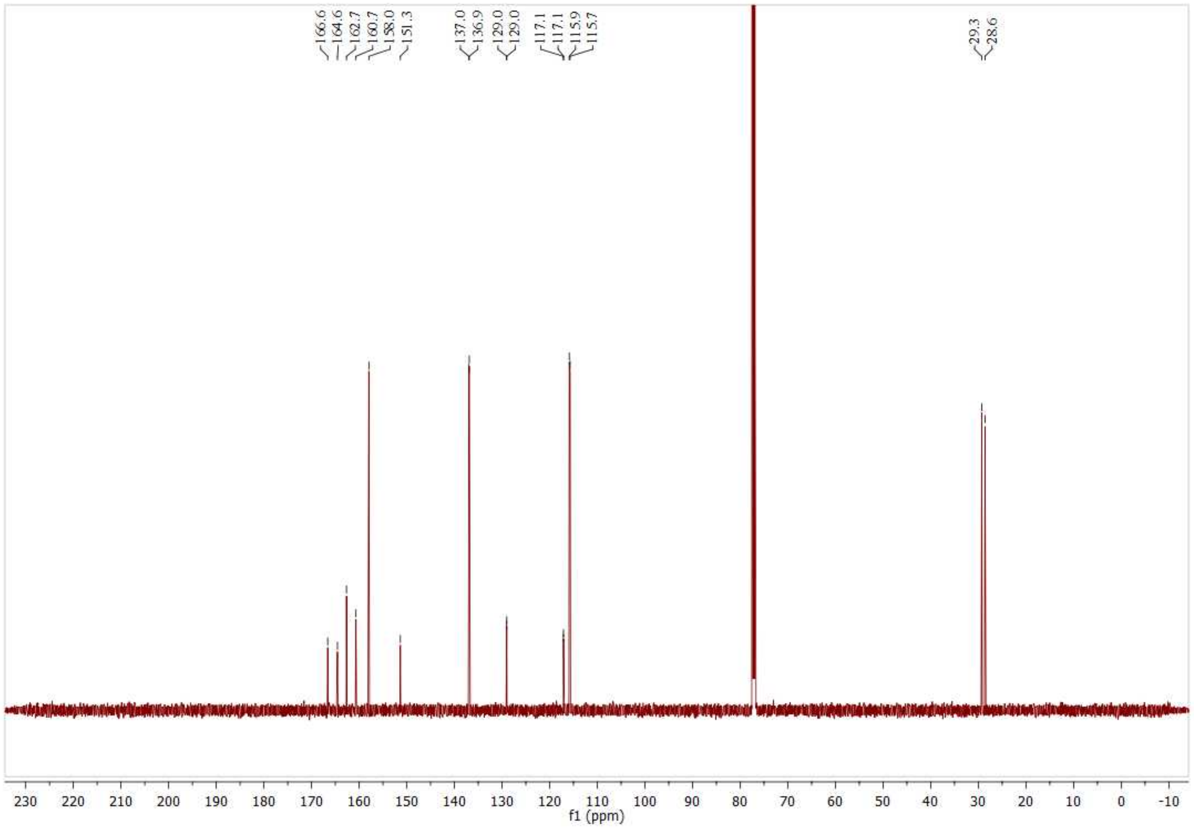

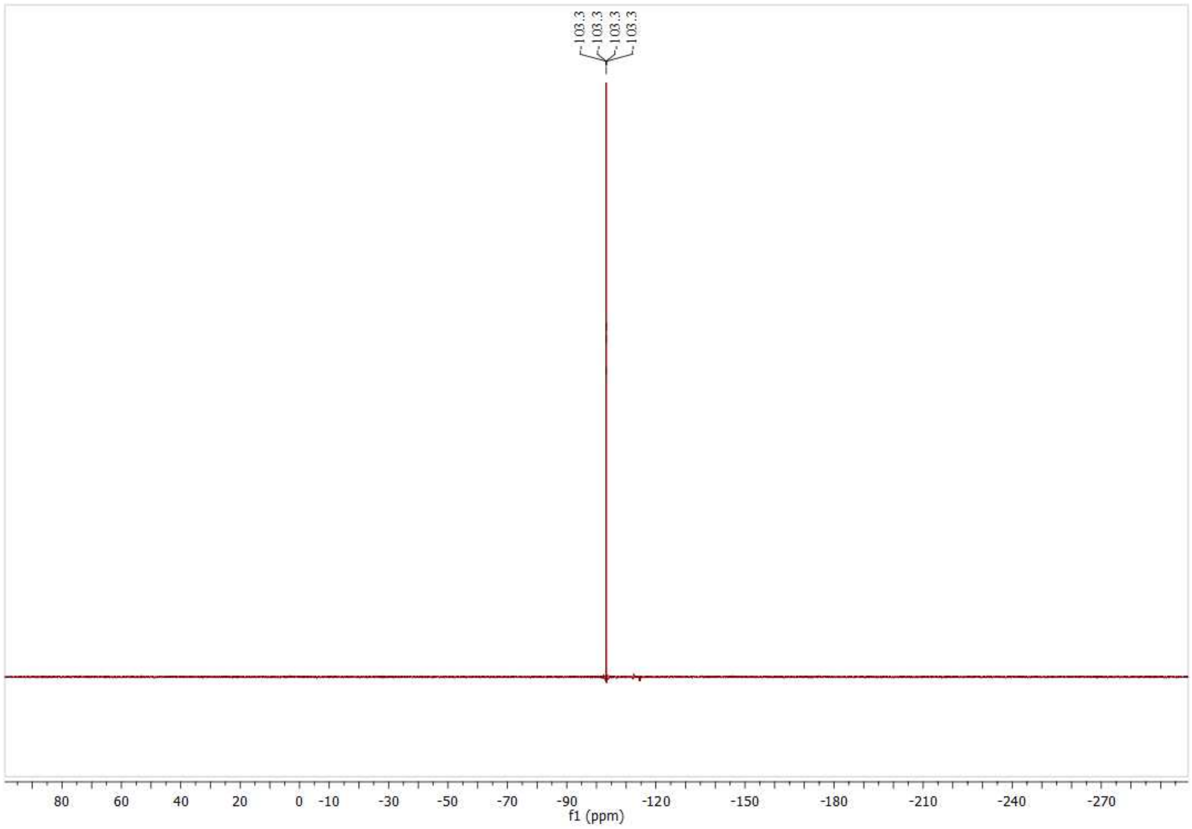

-103.3
-103.3
-103.3
-103.3
80
60
40
20
0
-10
-30
-50
-70
-90
-120
-150
-180
-210
-240
-270
f1 (ppm)

5-(4-Fluorobenzylidene)-2,2-dimethyl-1,3-dioxane-4,6-dione

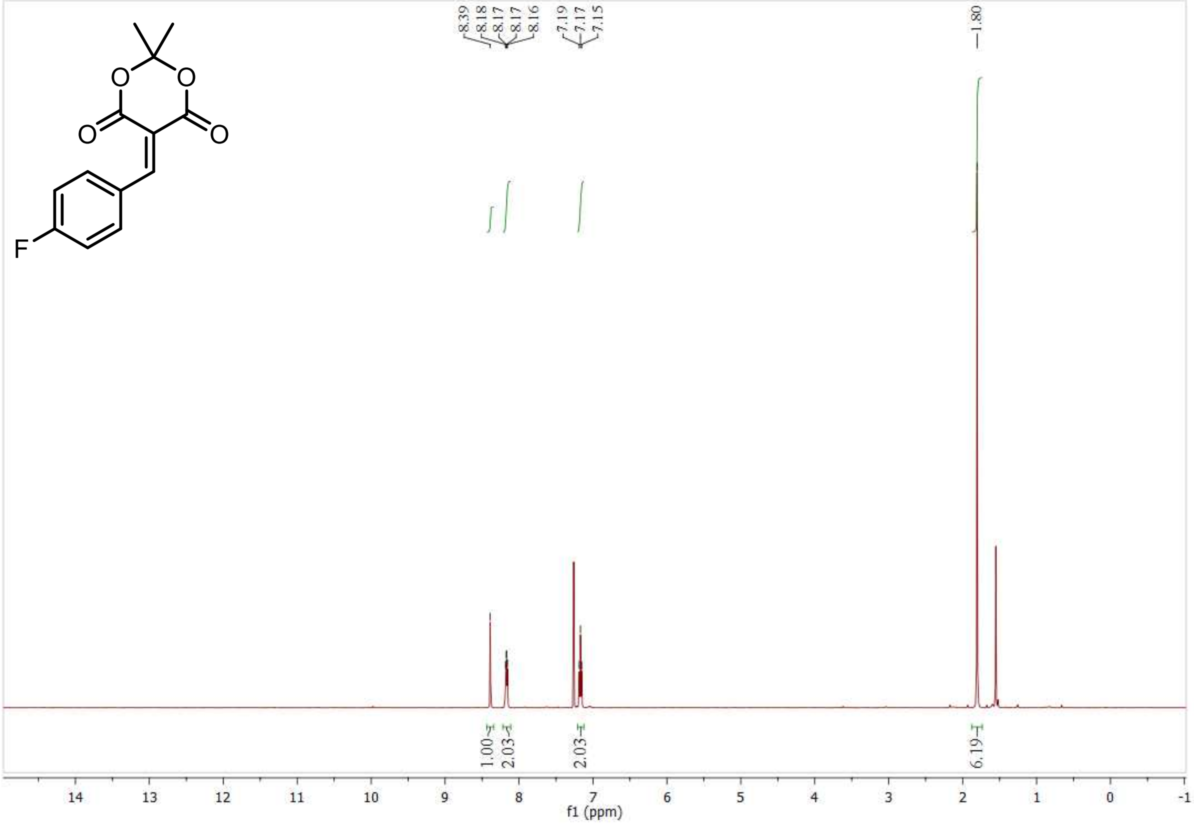

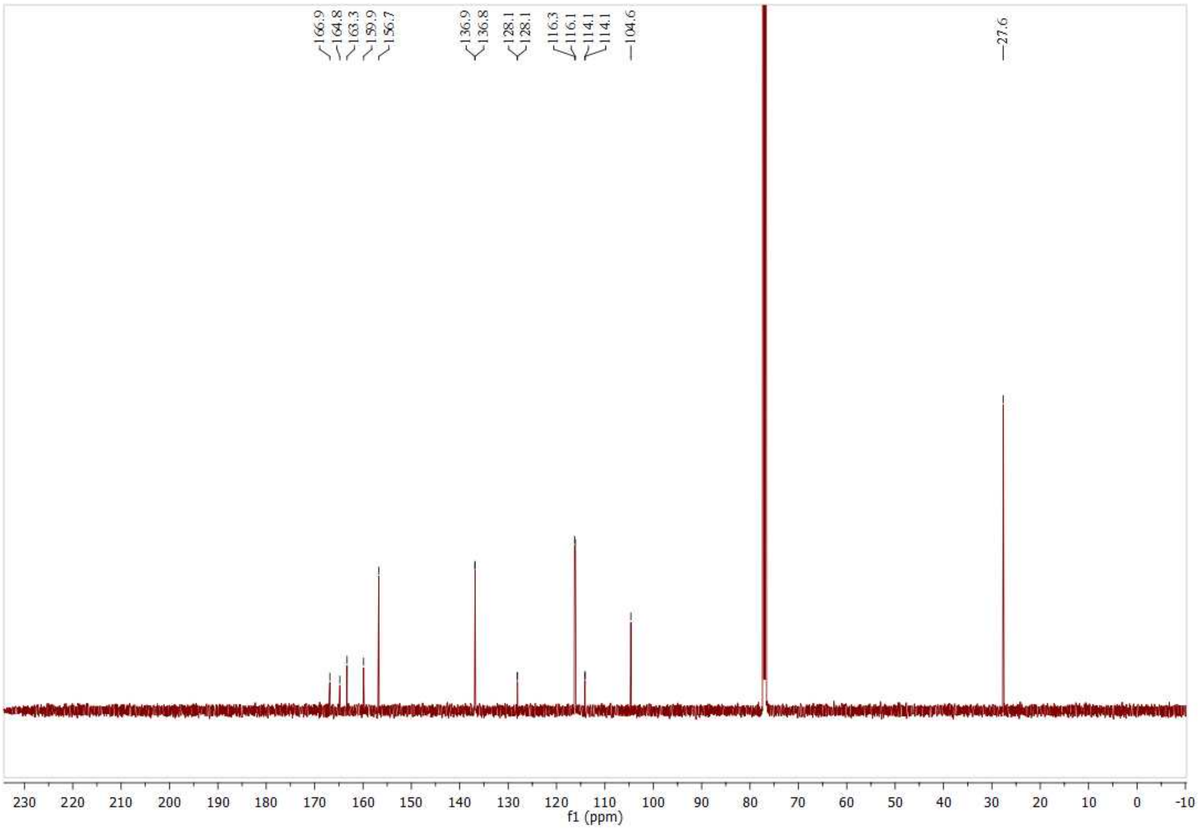

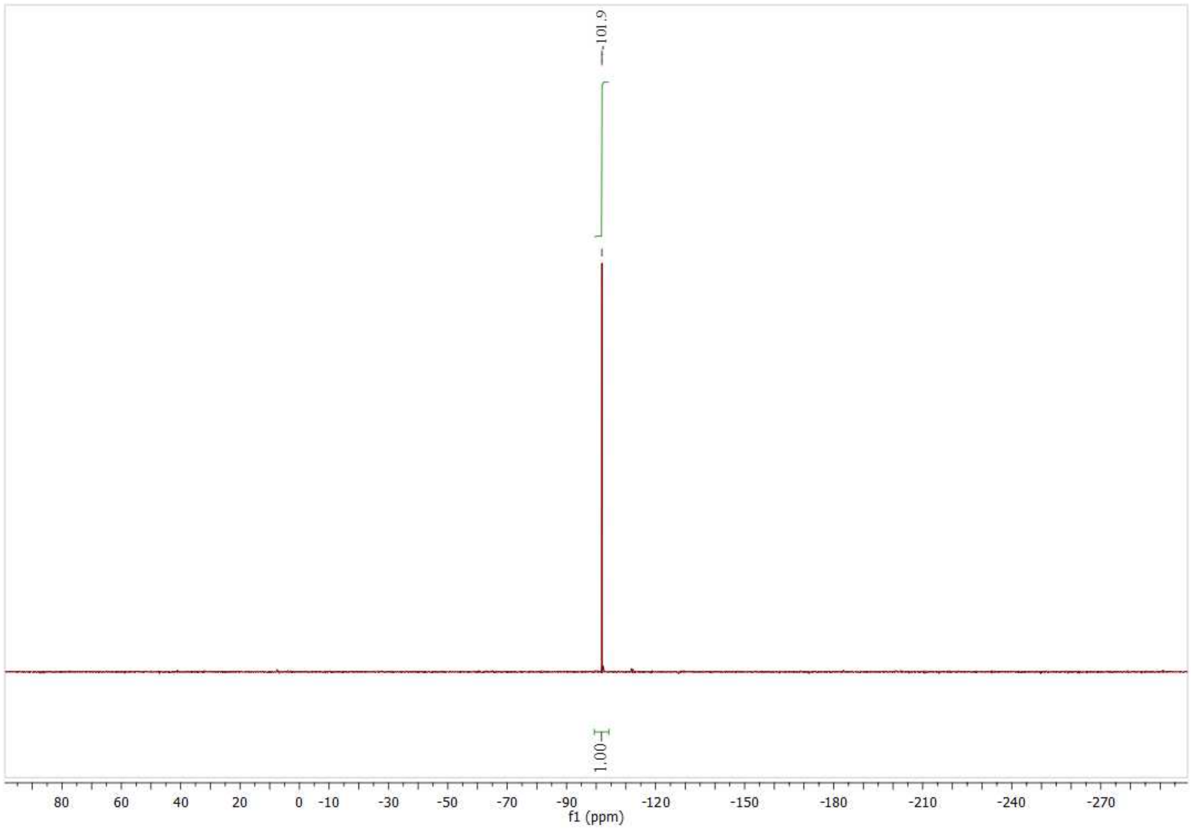
-101.9
1.00
80 60 40 20 0 -10 -30 -50 -70 -90 -120 -150 -180 -210 -240 -270
f1 (ppm)

Ethyl 2-oxo-2H-chromene-3-carboxylate

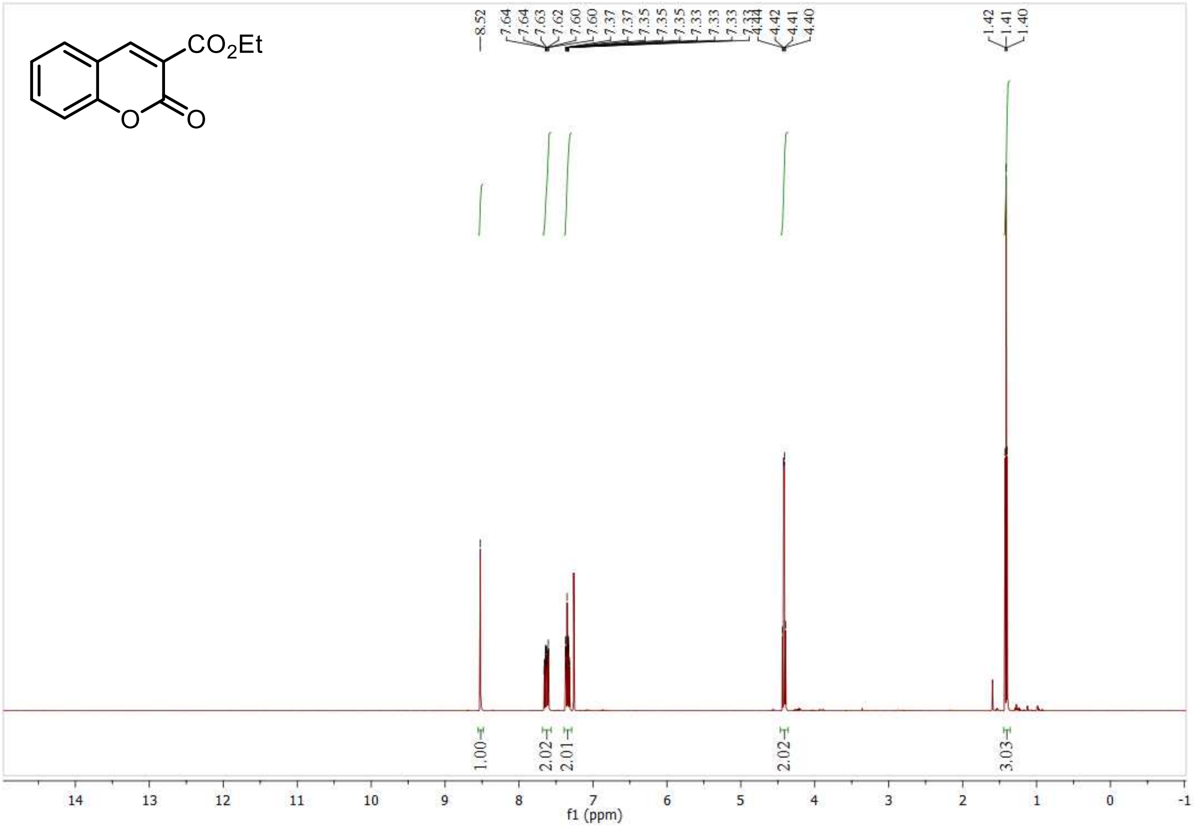

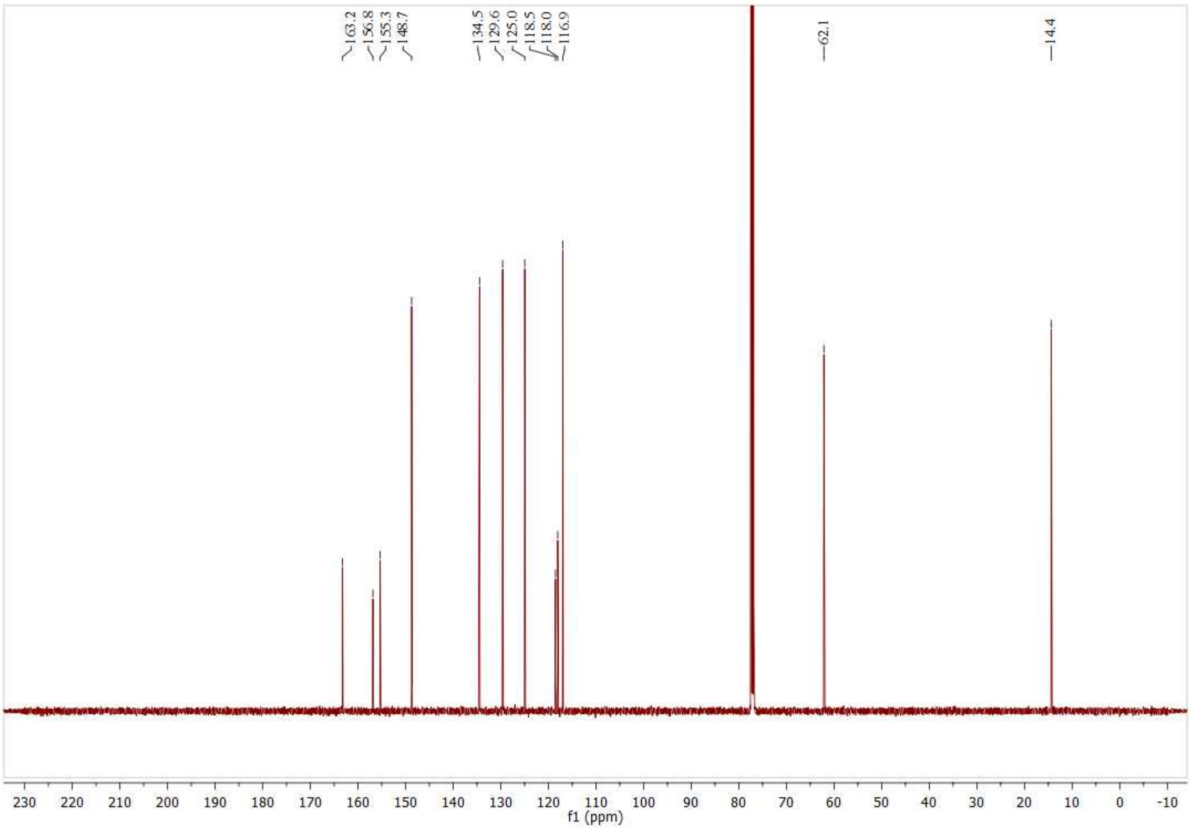

2-benzylidenemalononitrile

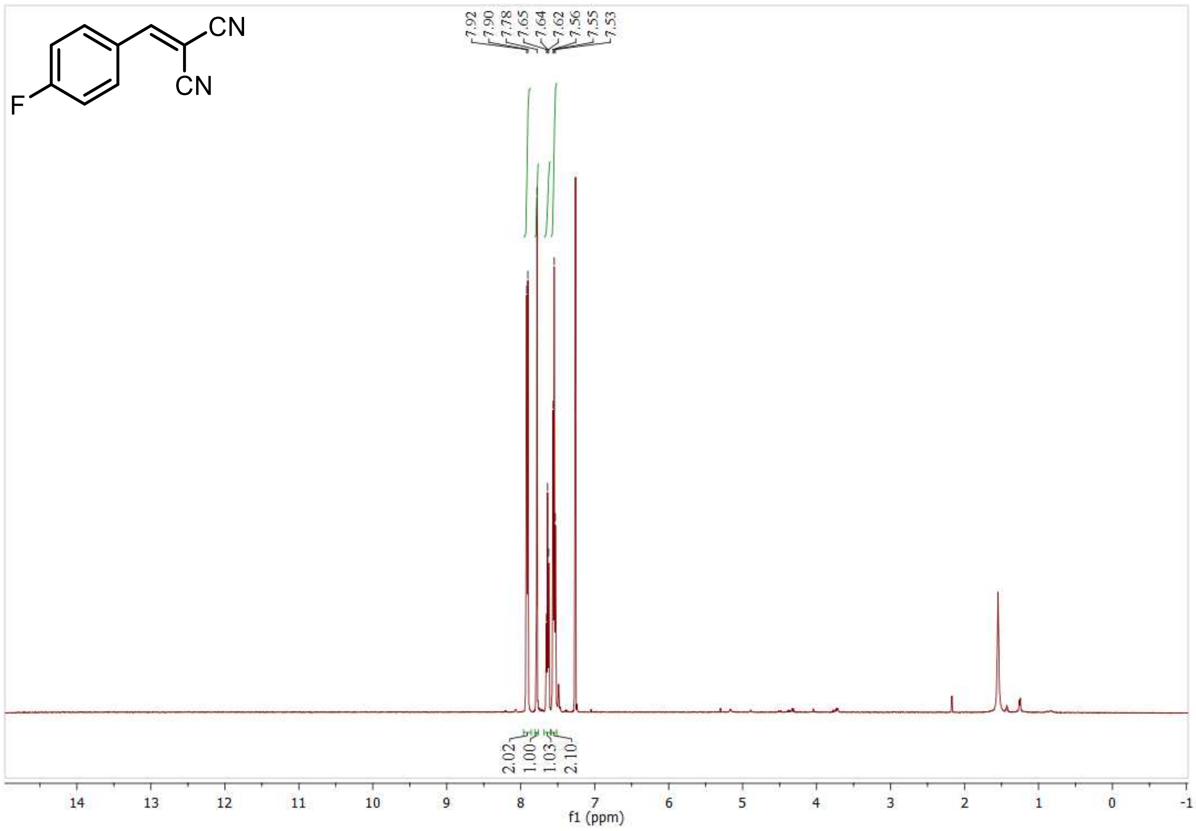

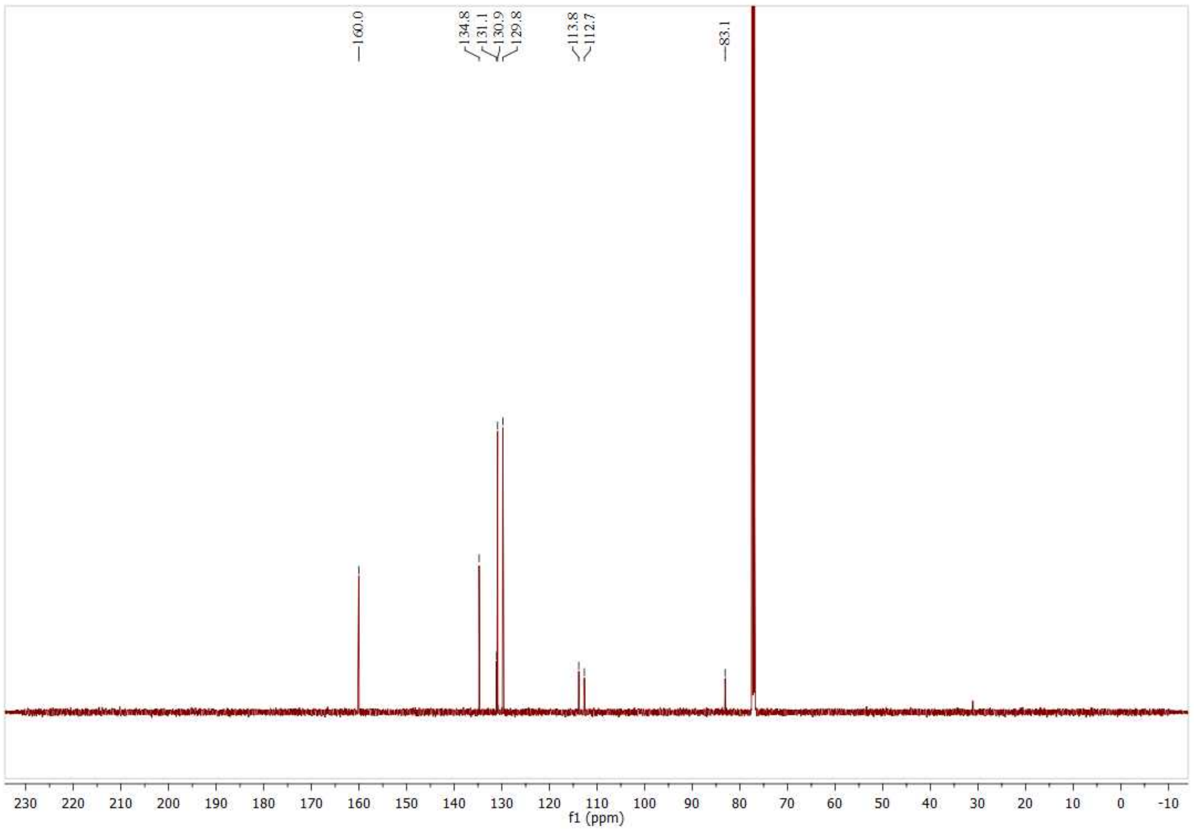

Ethyl 3-phenylpropiolate

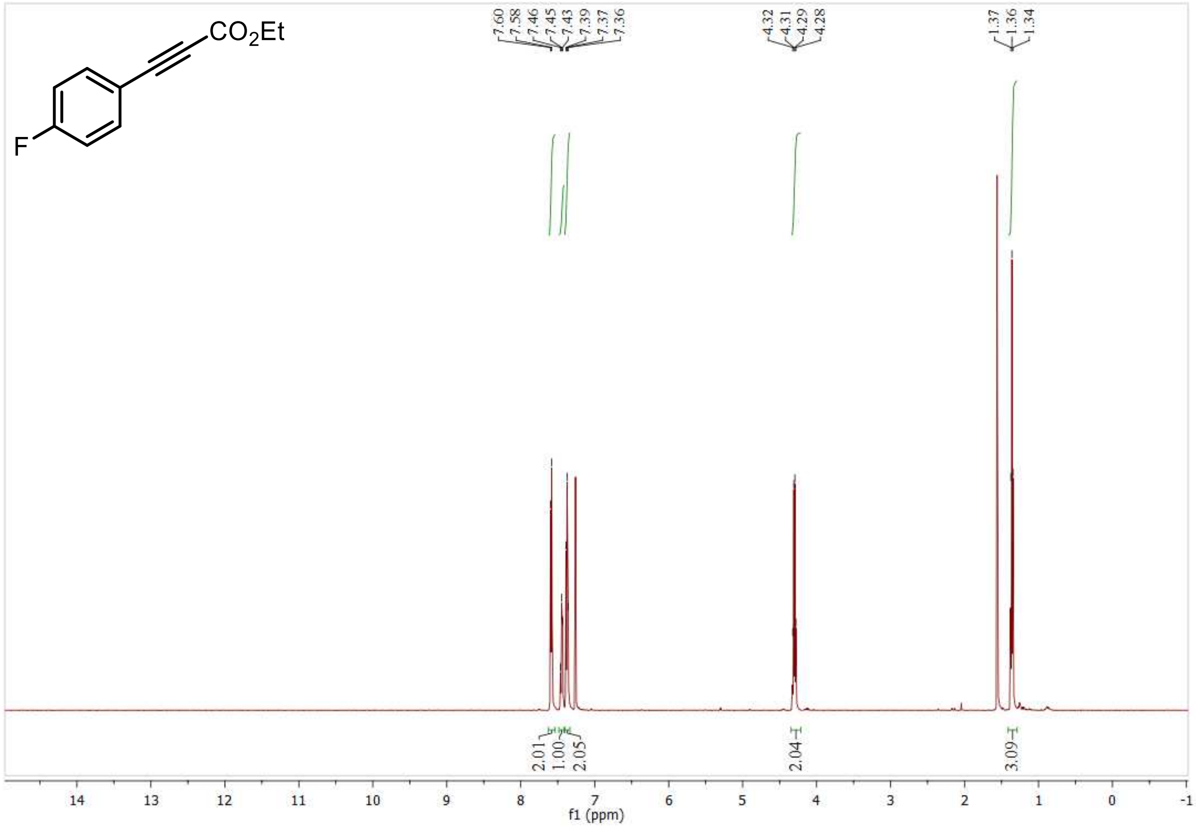

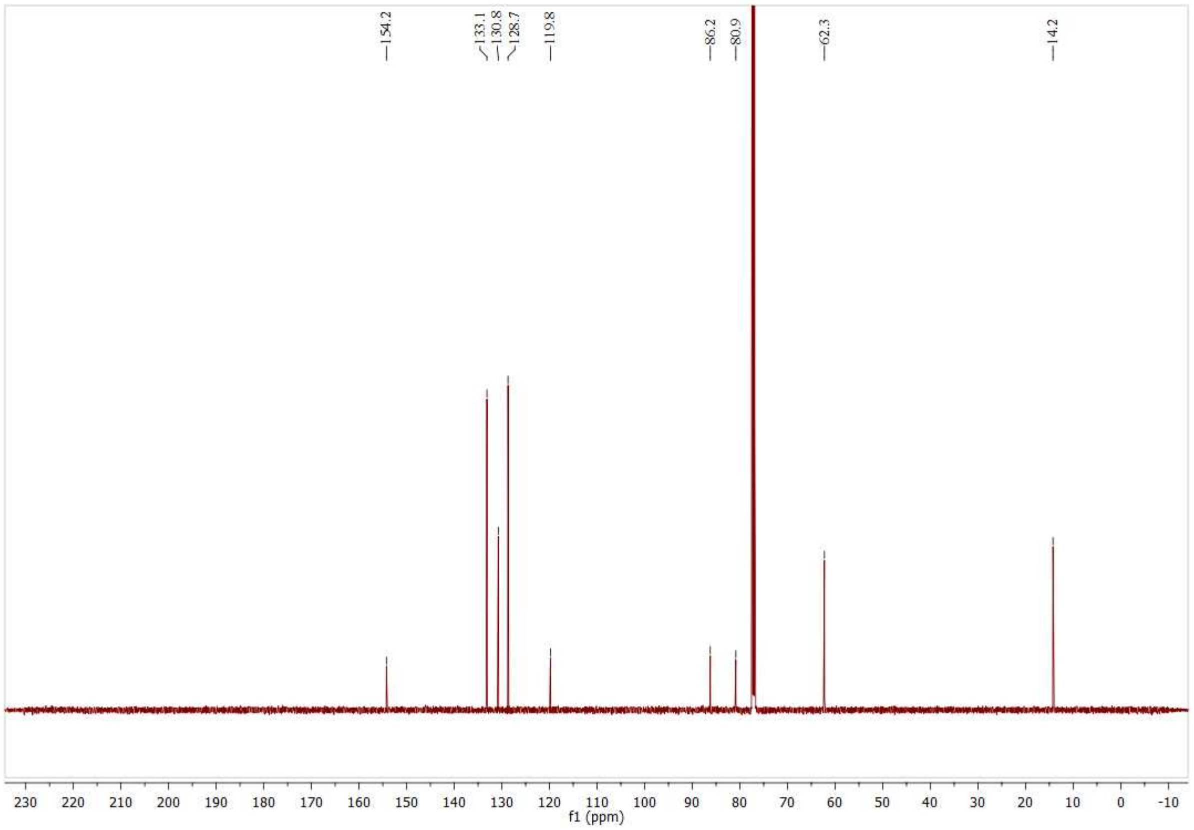

Diethyl 2-(4-fluorobenzyl)malonate

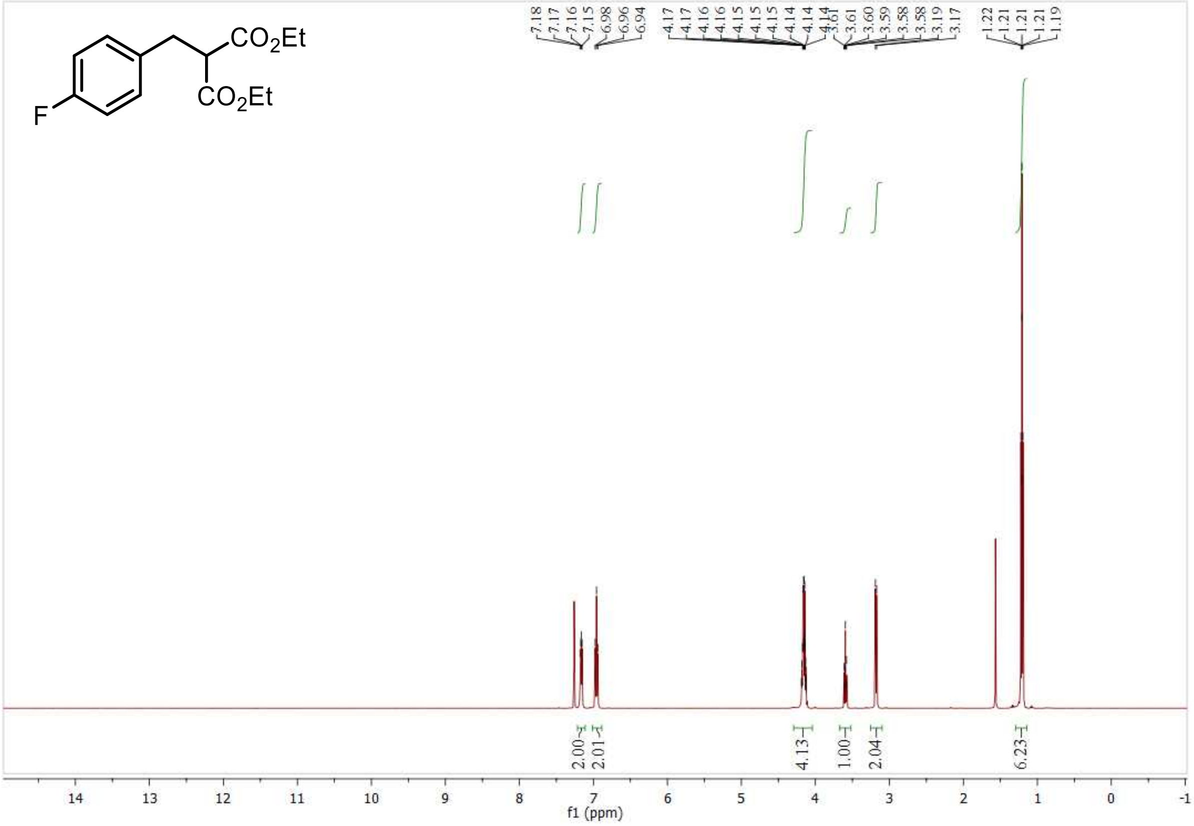

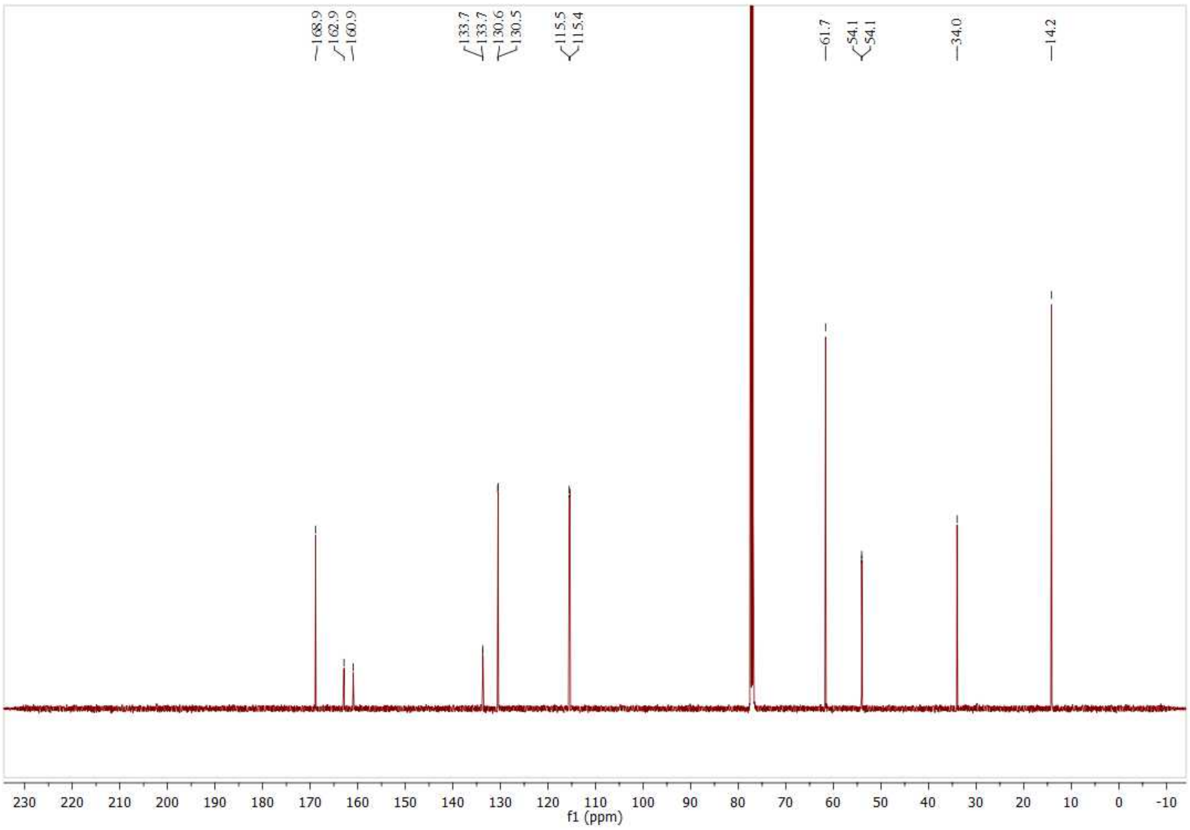

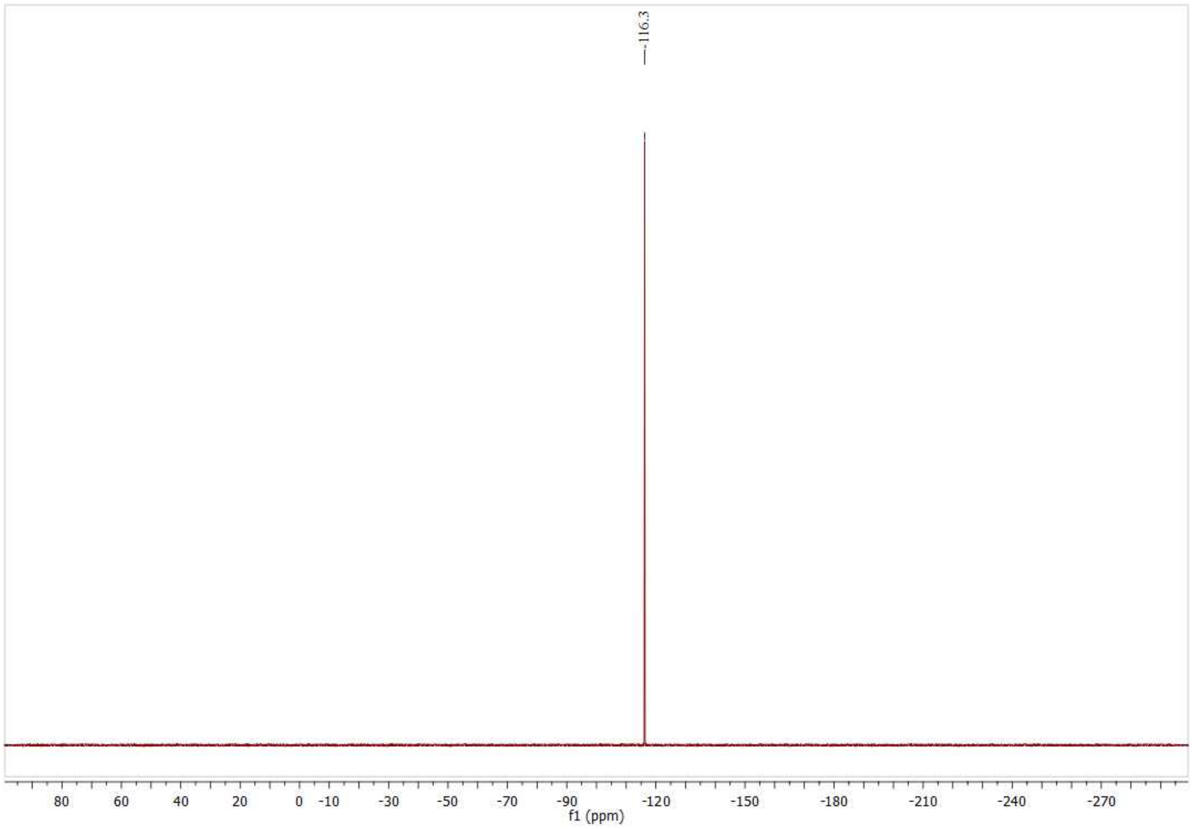
-116.3
80
60
40
20
0
-10
-30
-50
-70
-90
-120
-150
-180
-210
-240
-270
f1 (ppm)

Methyl 4-formylbenzoate

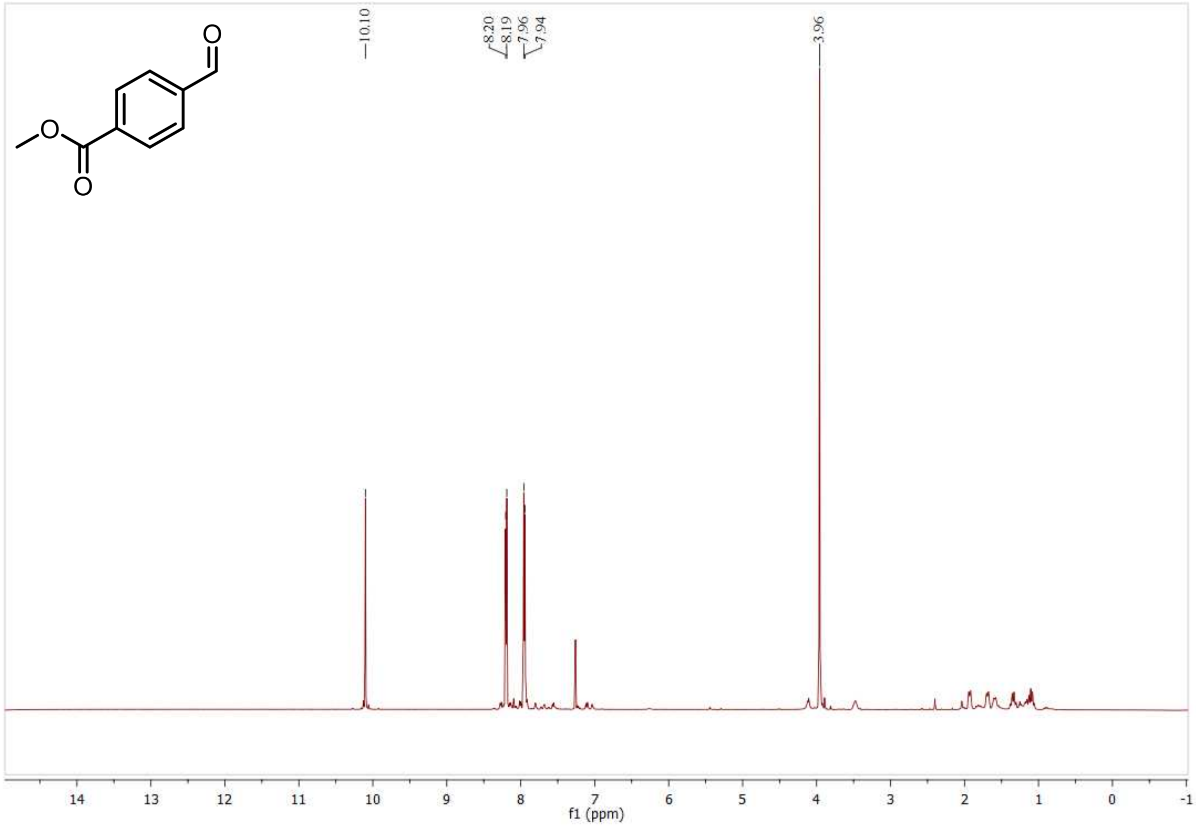

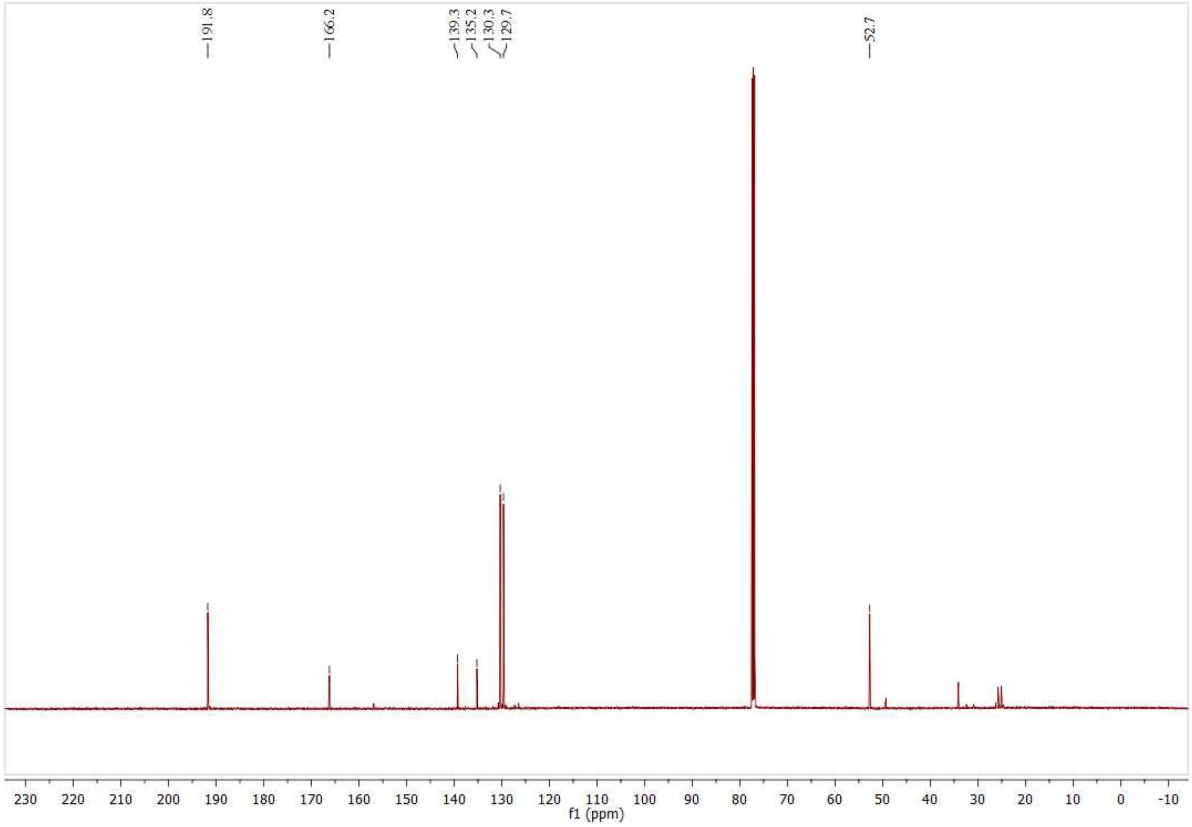

Di-*iso*-propyl malonate

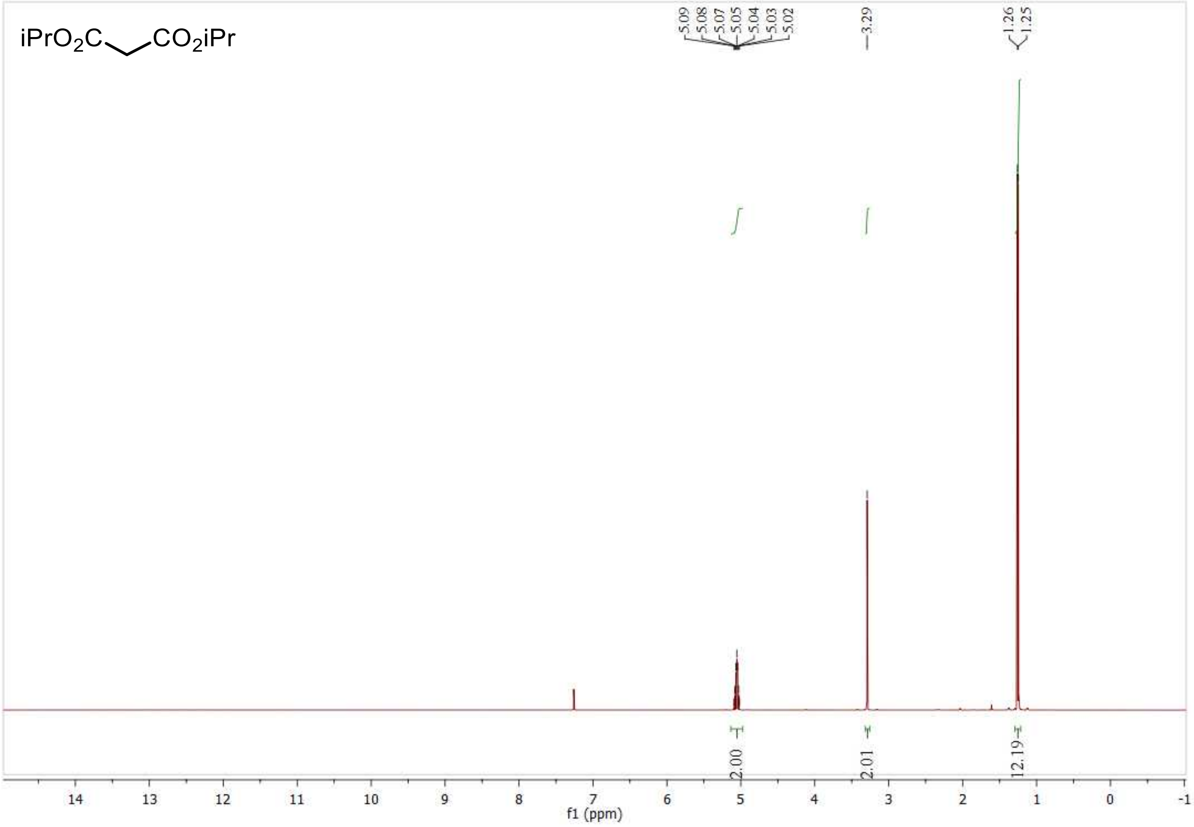

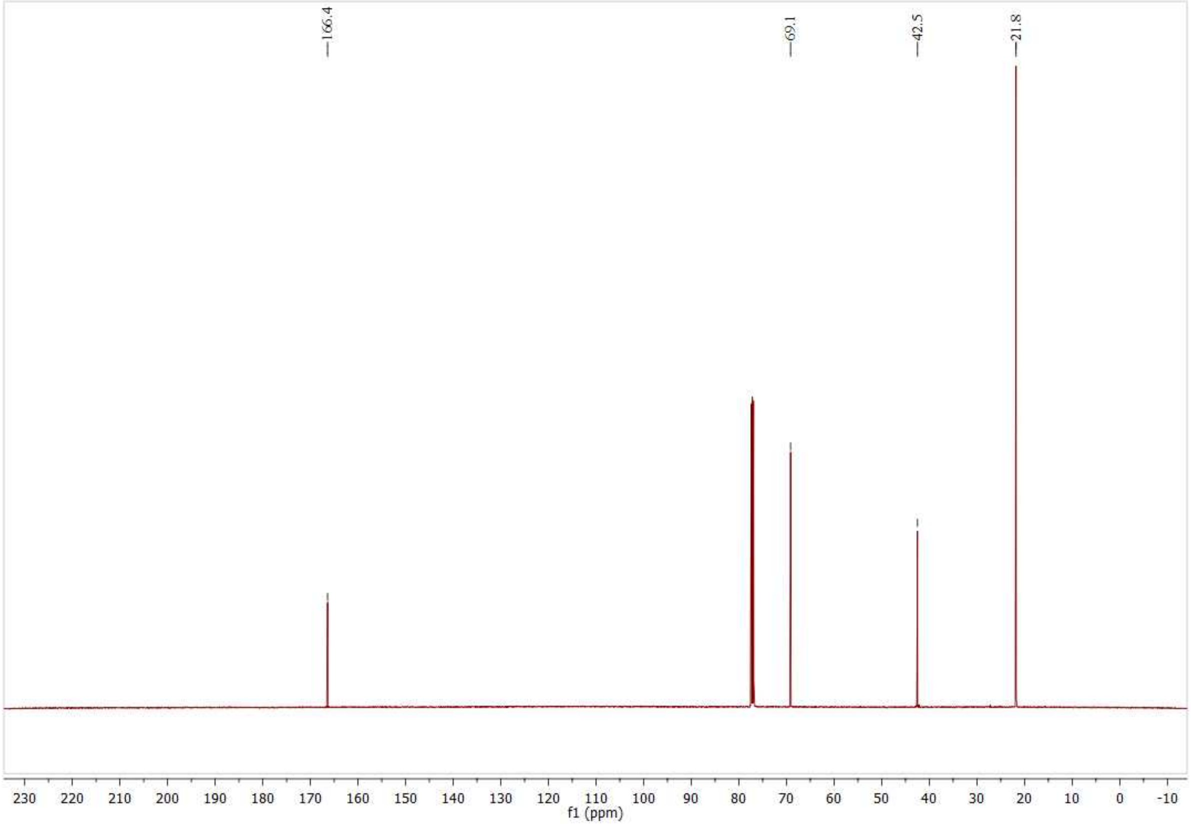

Di-*tert*-butyl malonate

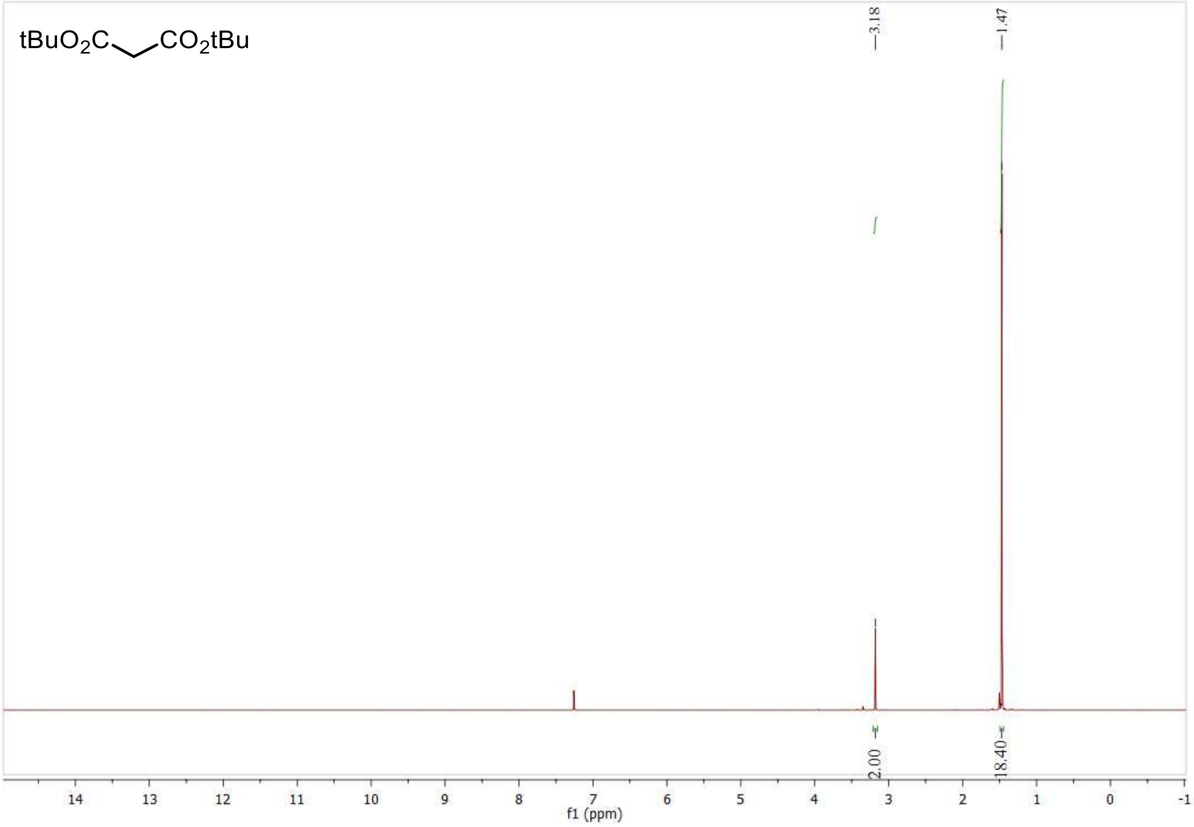

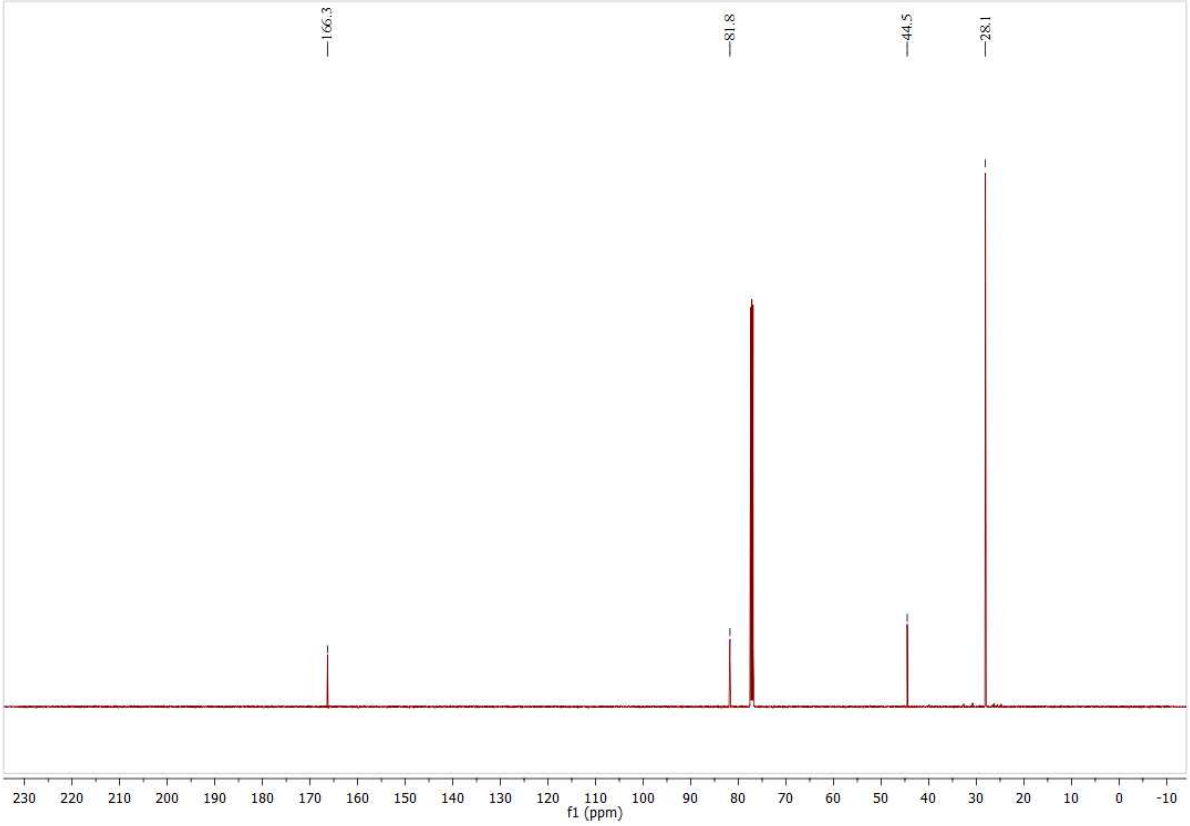

Diallyl malonate

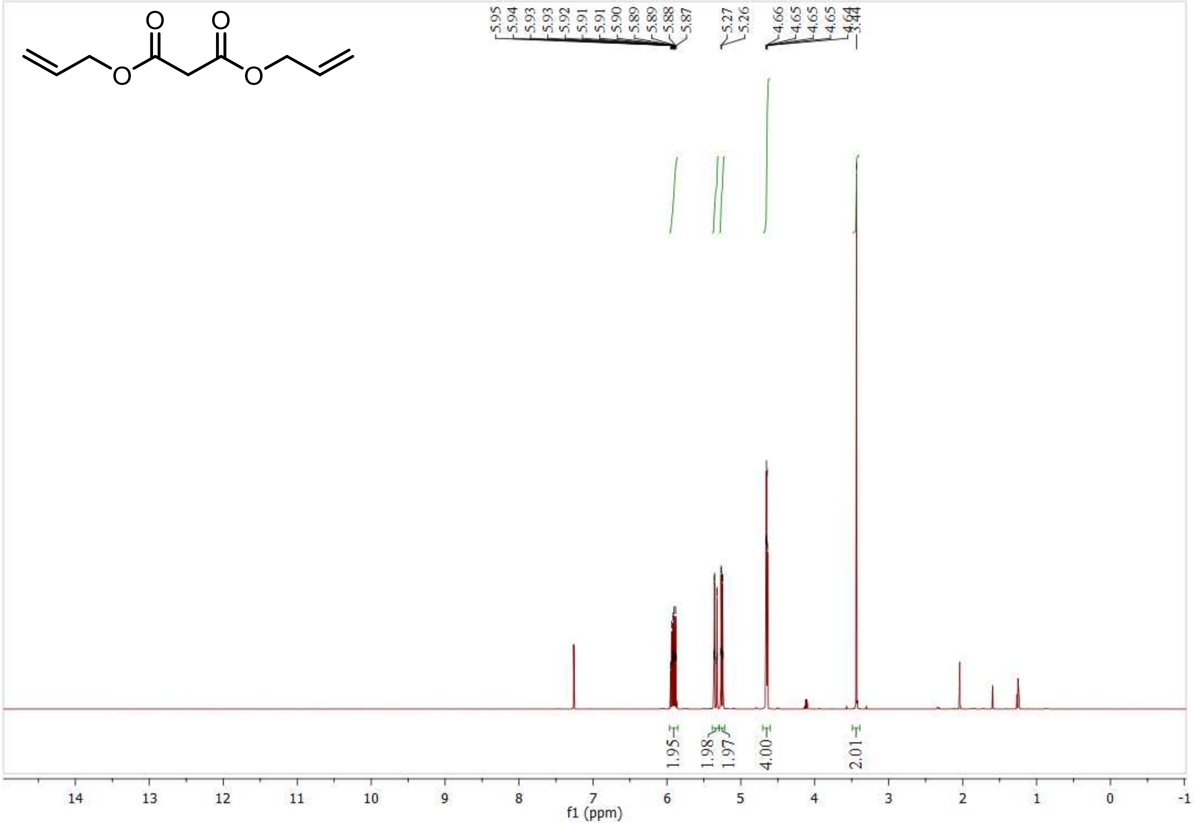

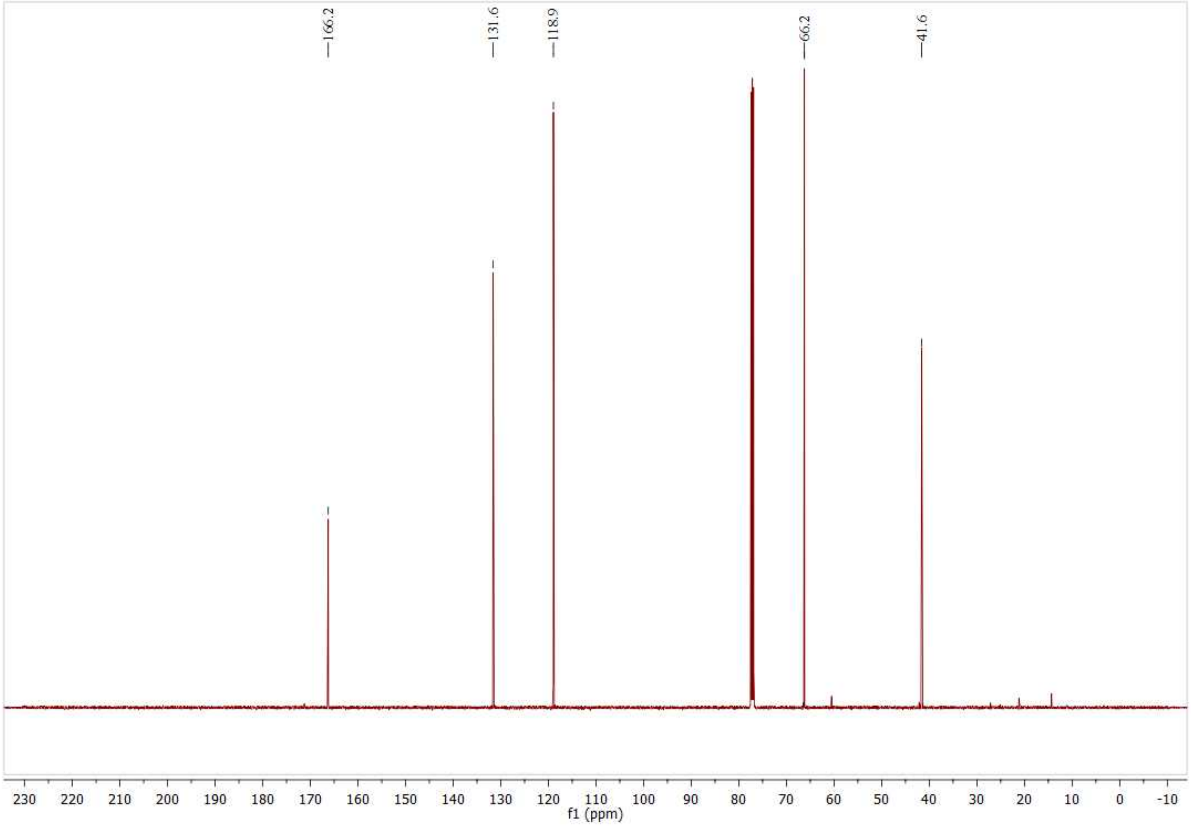

Tetraethyl 2,3-bis(4-fluorophenyl)butane-1,1,4,4-tetracarboxylate ANTI

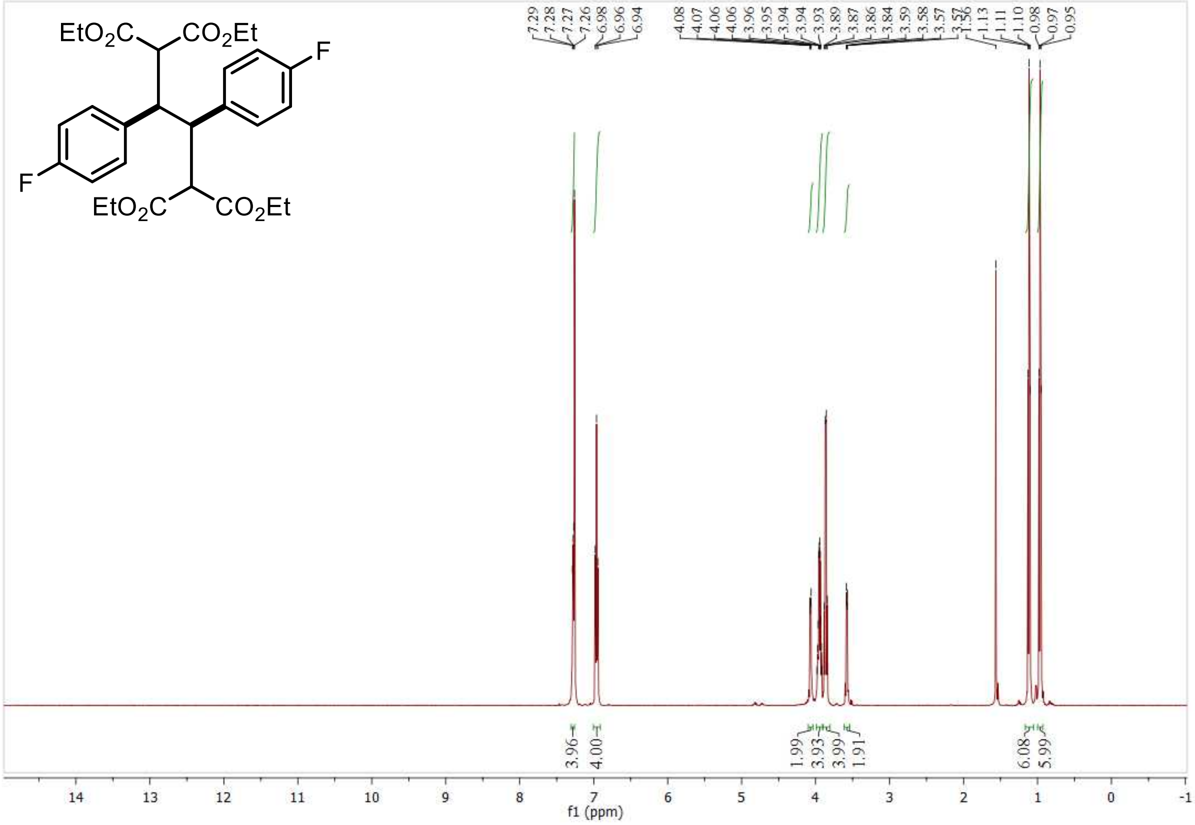

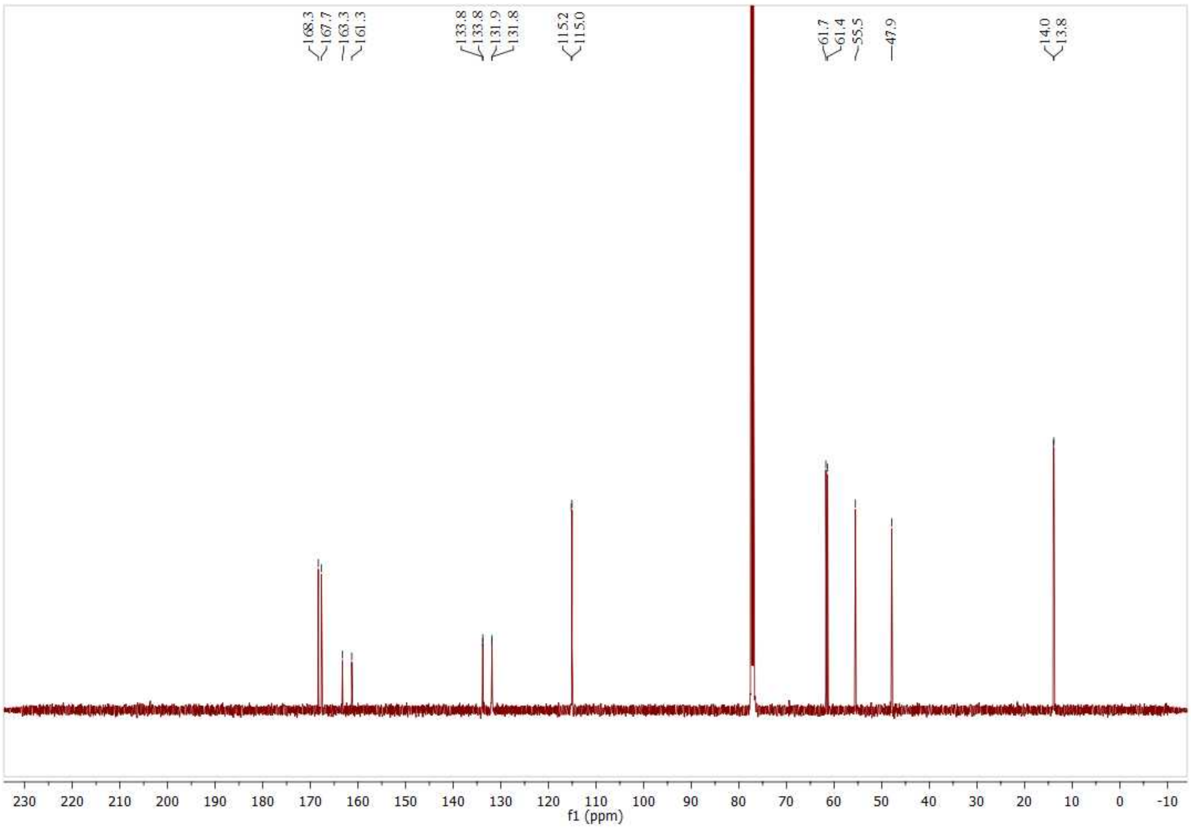

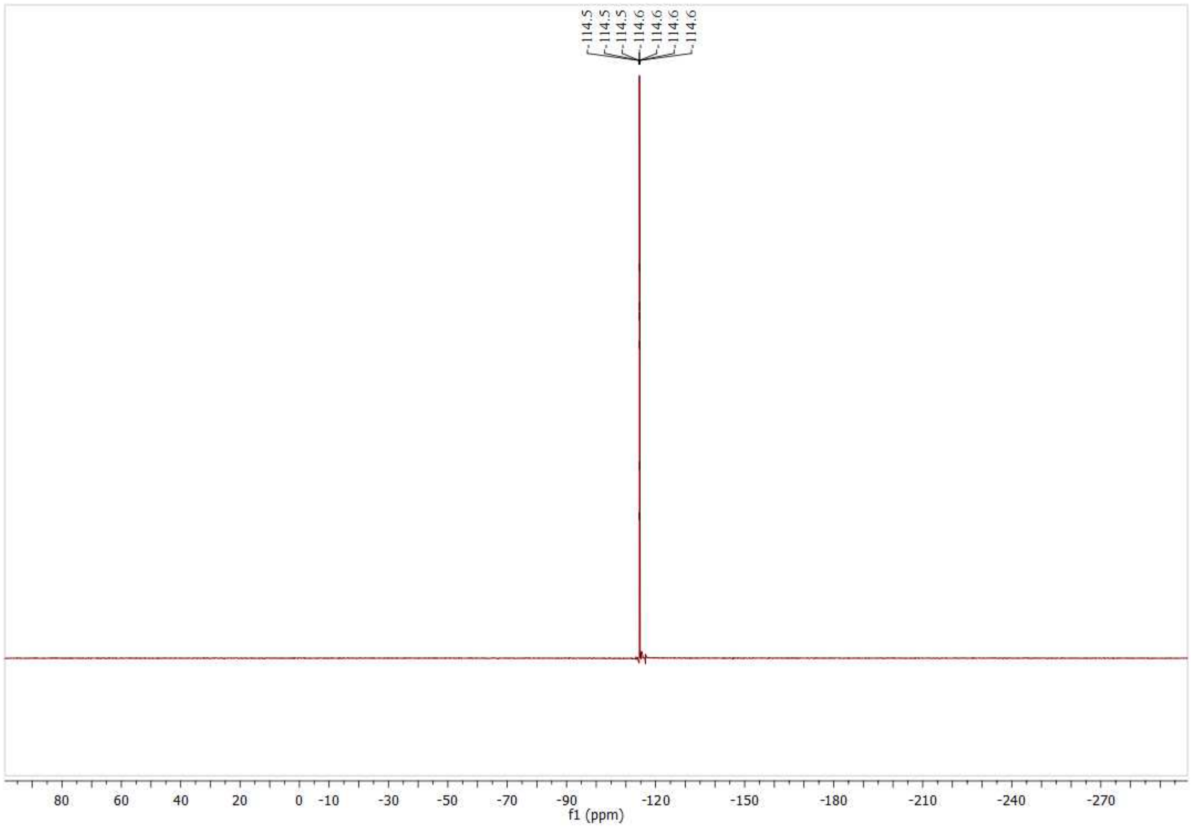
-114.5
-114.5
-114.5
-114.6
-114.6
-114.6
-114.6
80 60 40 20 0 -10 -30 -50 -70 -90 -120 -150 -180 -210 -240 -270
f1 (ppm)

Tetraethyl 2,3-bis(4-fluorophenyl)butane-1,1,4,4-tetracarboxylate SYN

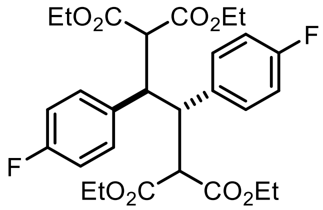

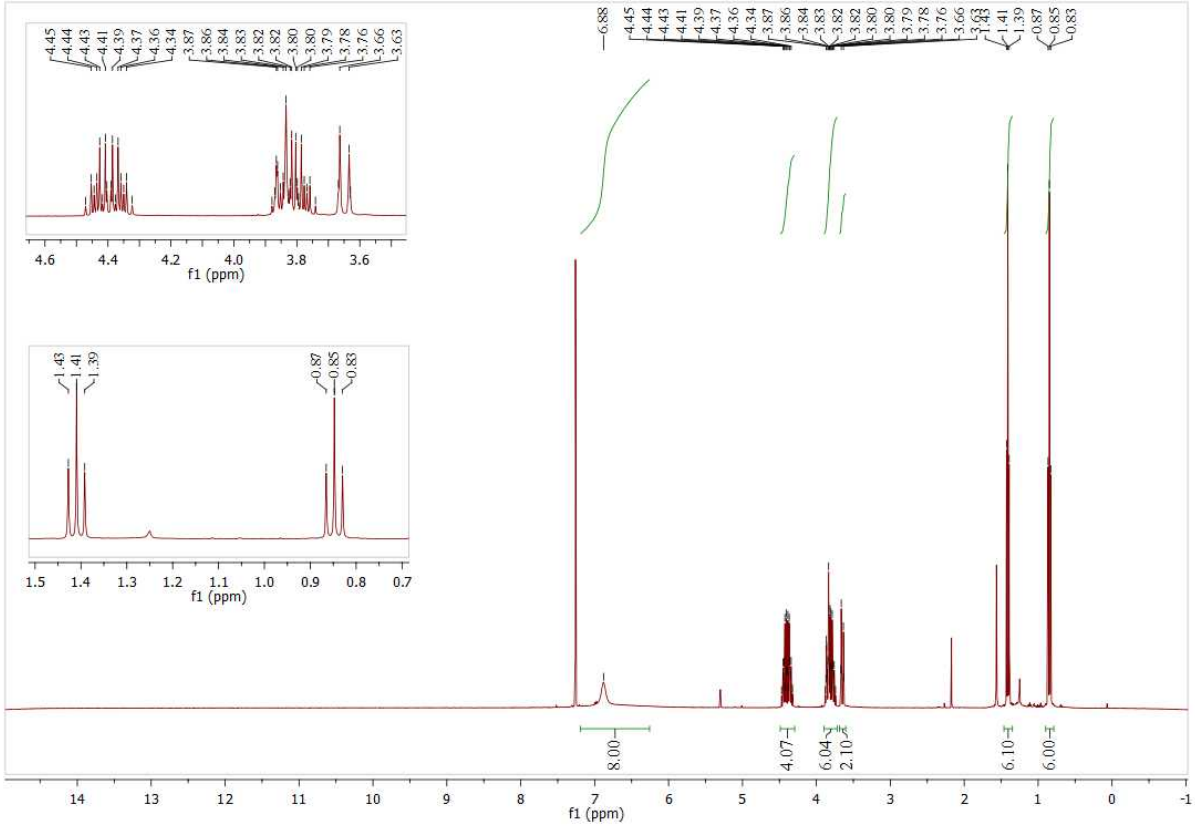

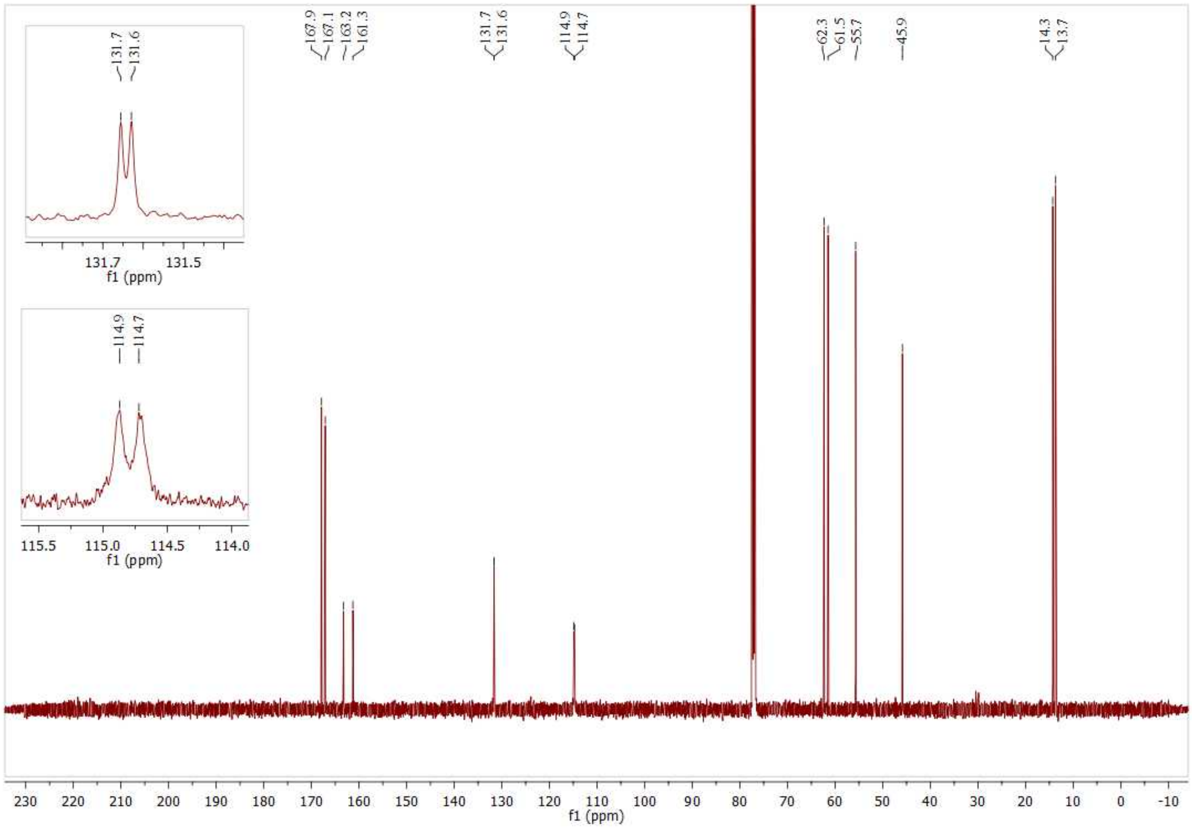
167.9
167.1
163.2
161.3
131.7
131.6
114.9
114.7
62.3
61.5
55.7
45.9
14.3
13.7
f1 (ppm)

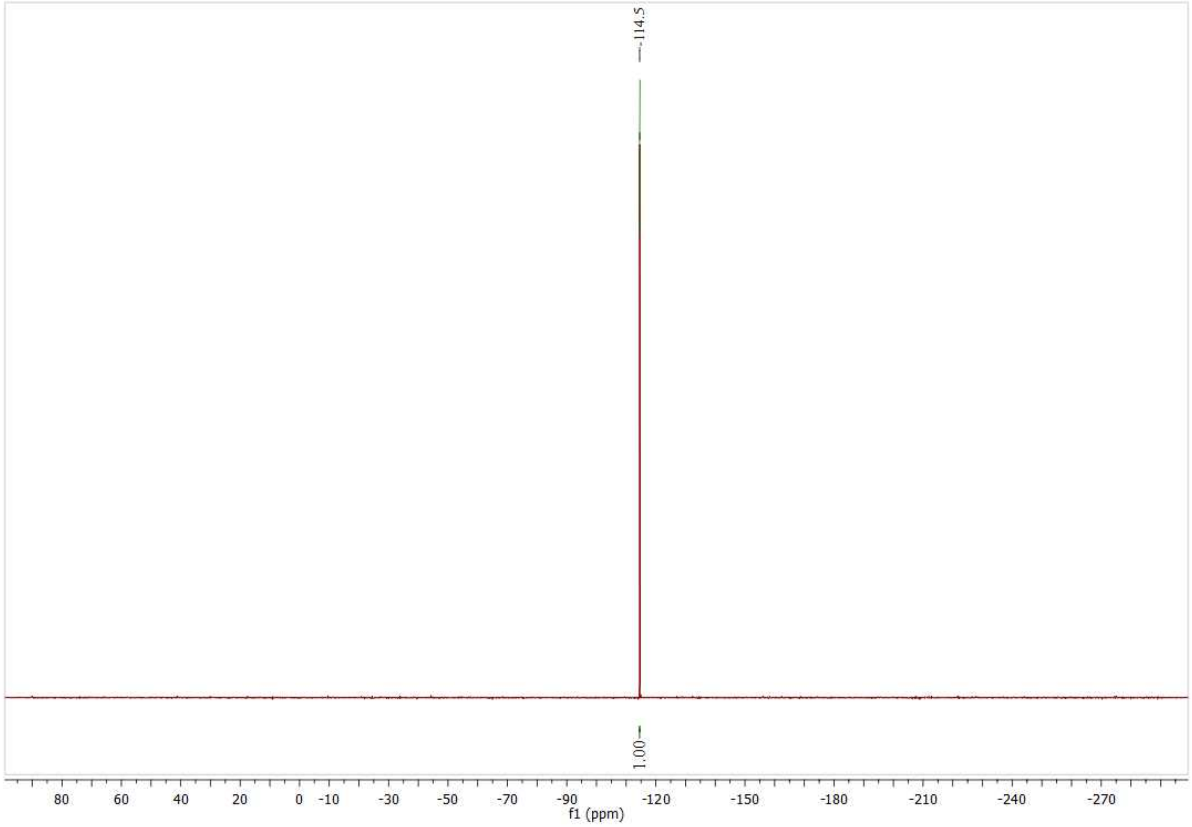
-114.5
1.00
f1 (ppm)

Tetraethyl 2,3-diphenylbutane-1,1,4,4-tetracarboxylate ANTI

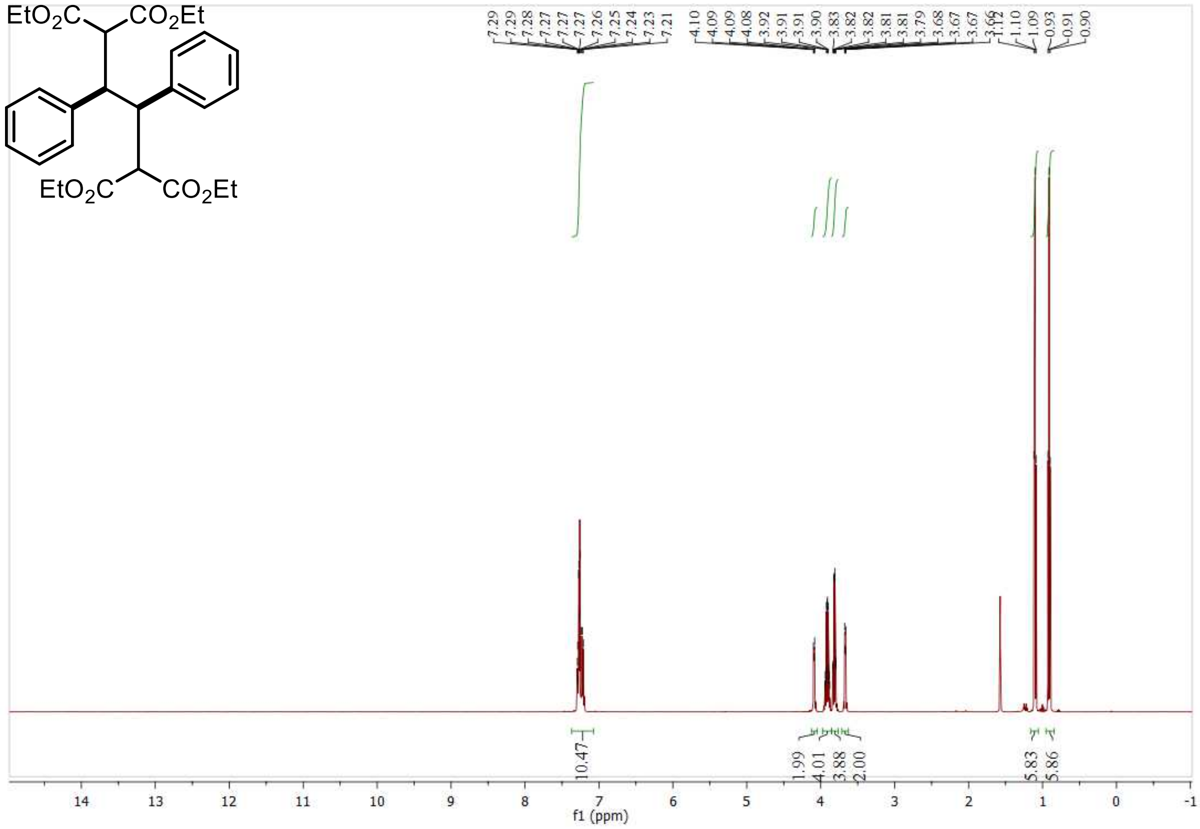

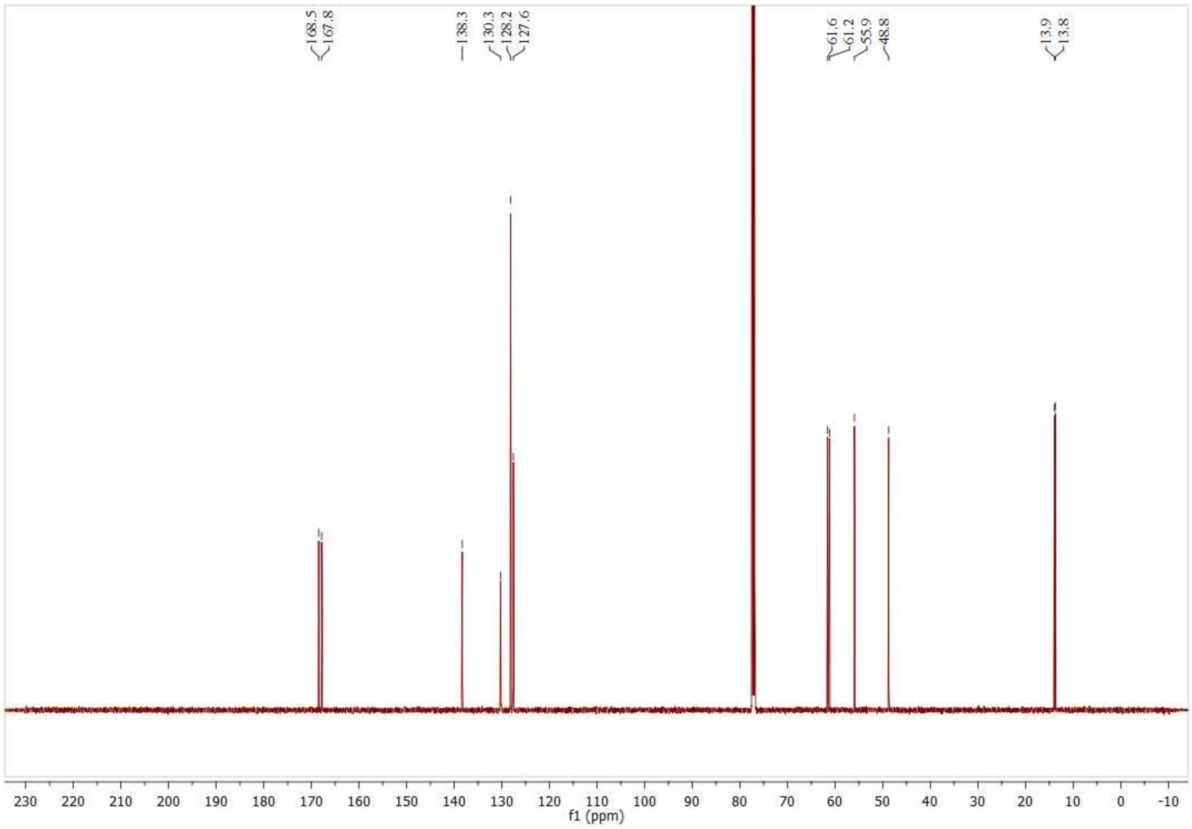

Tetraethyl 2,3-diphenylbutane-1,1,4,4-tetracarboxylate SYN

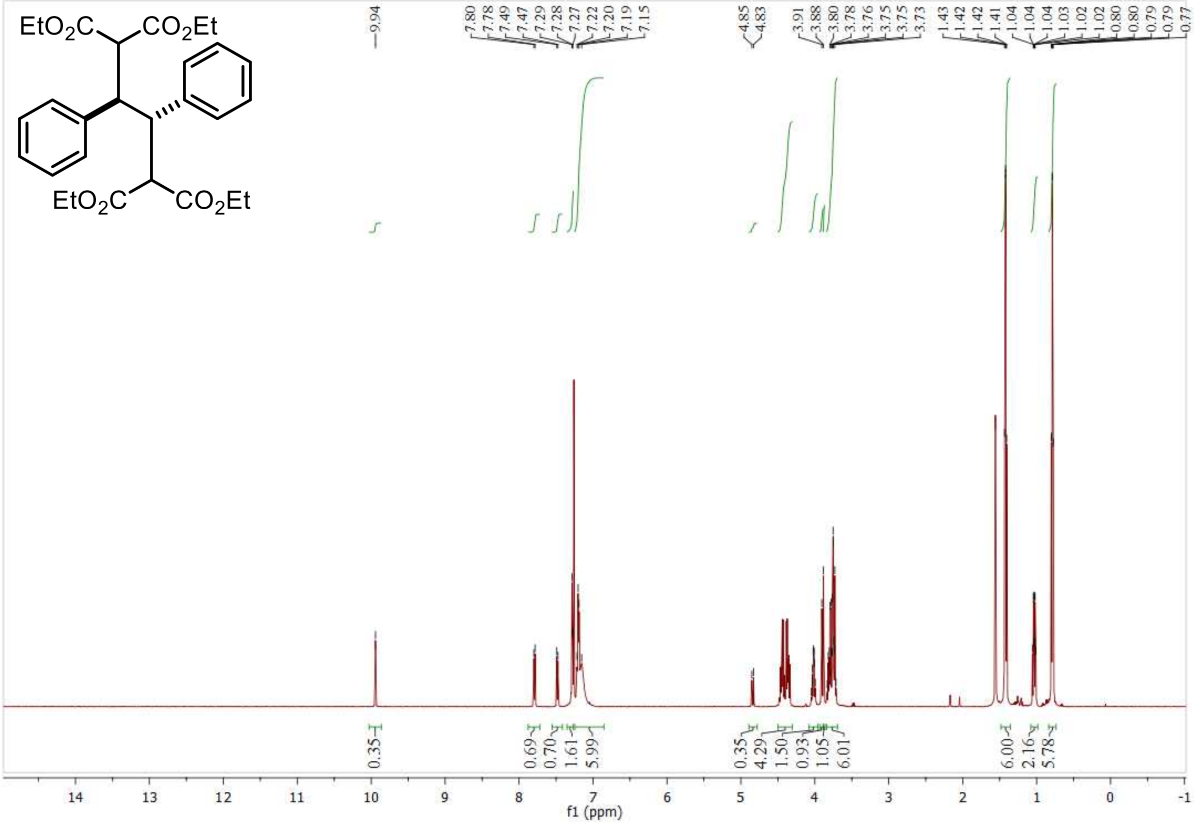

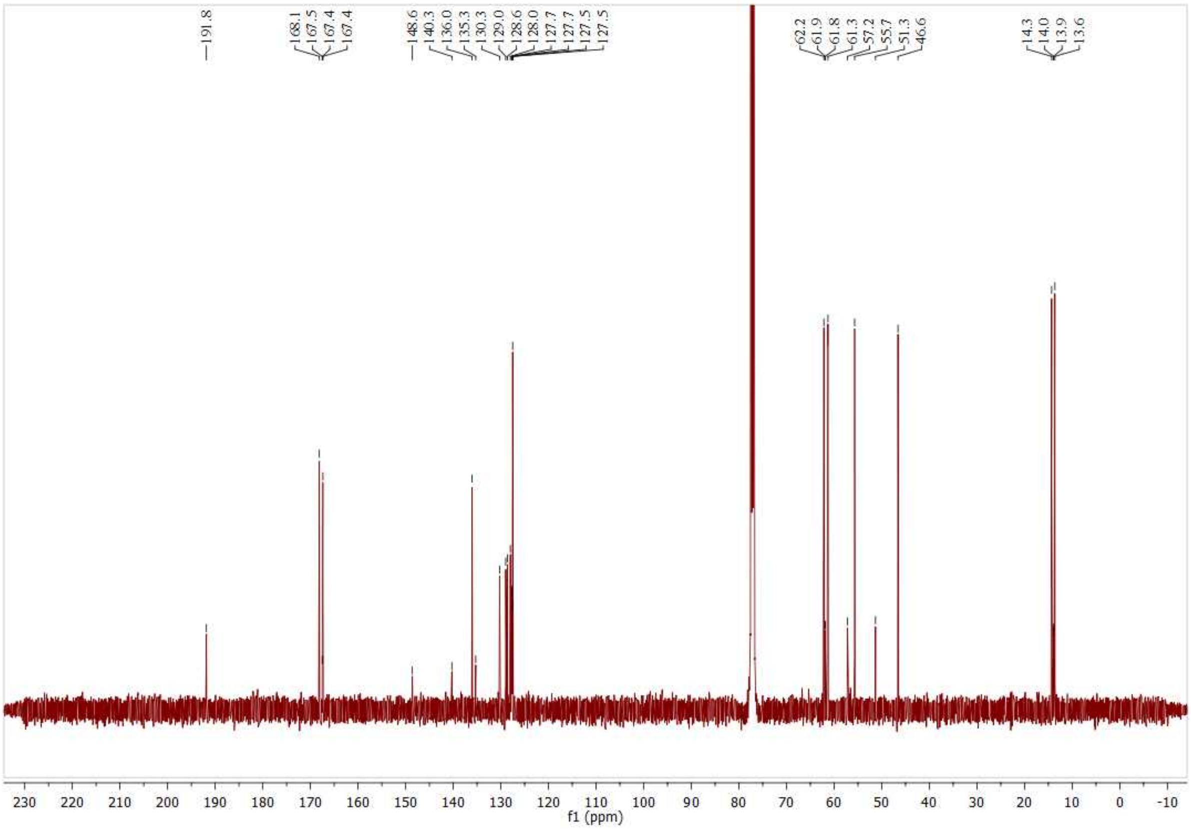

Tetraethyl (2,3-bis(4-(trifluoromethyl)phenyl)butane-1,1,4,4-tetracarboxylate ANTI

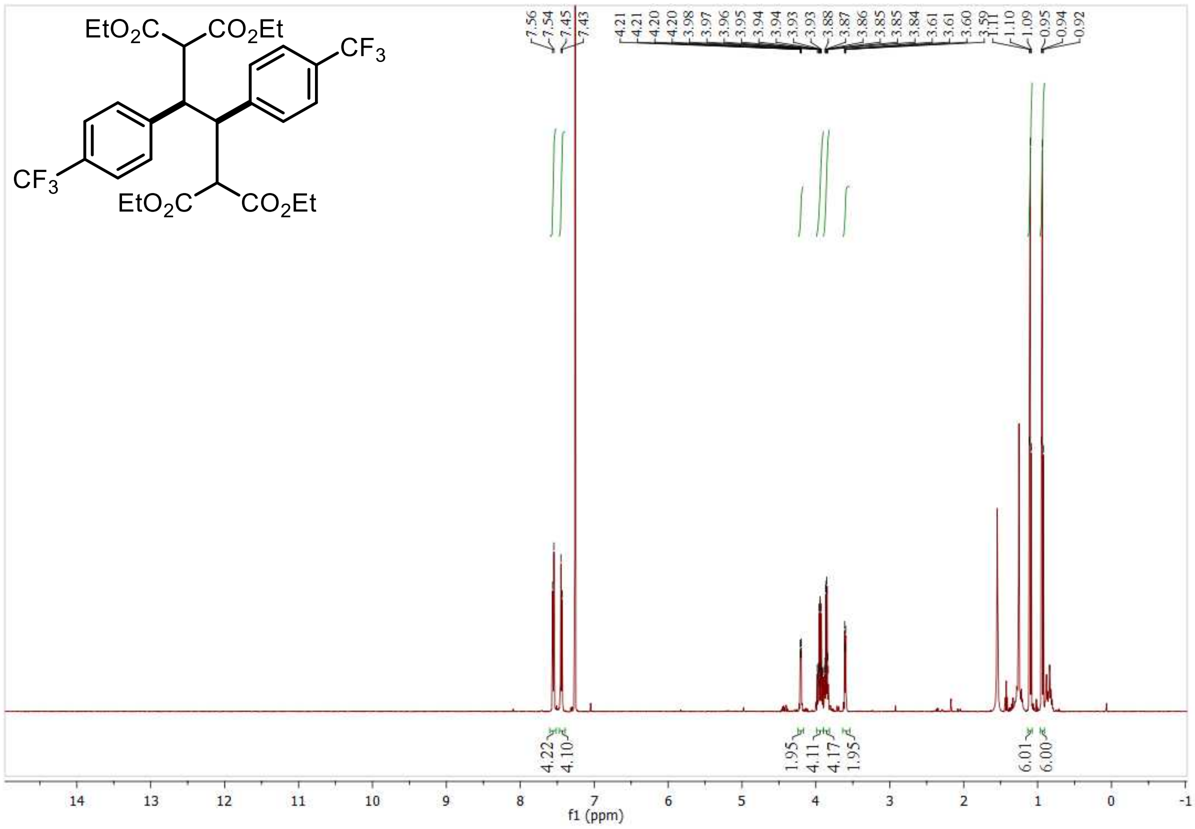

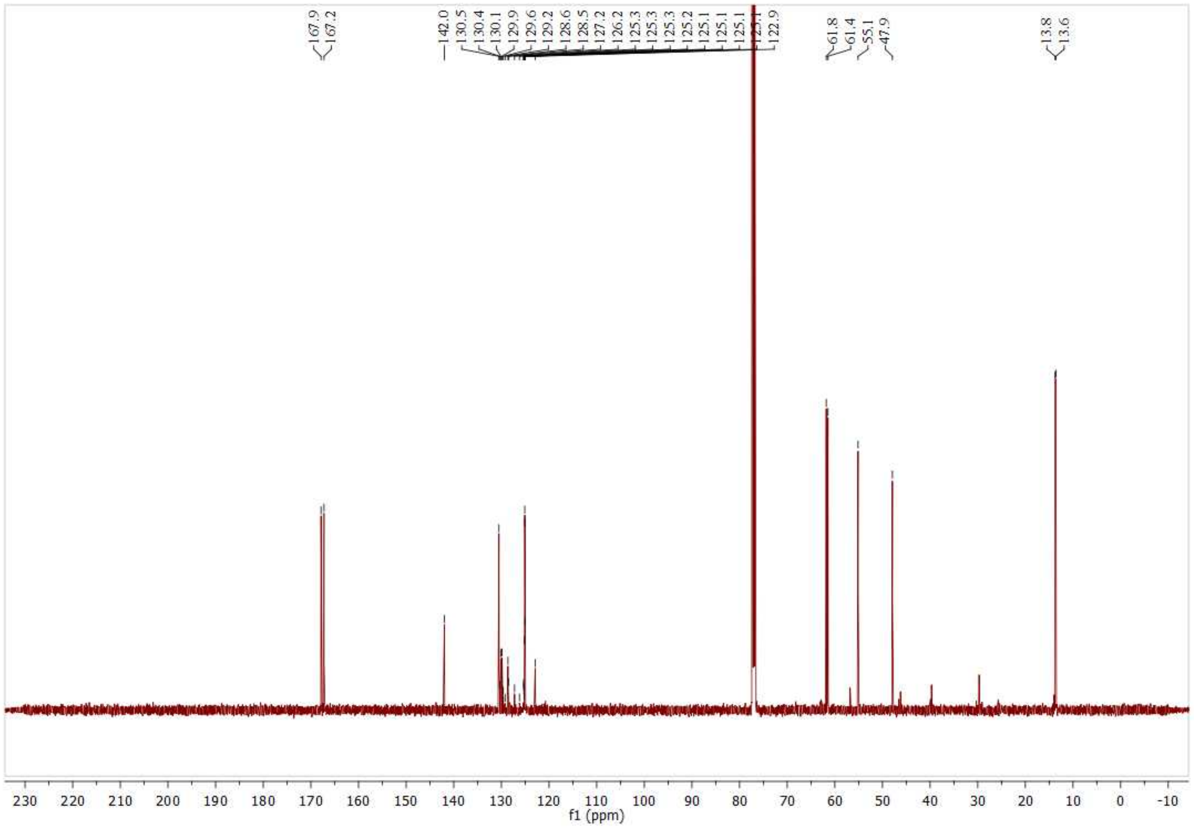

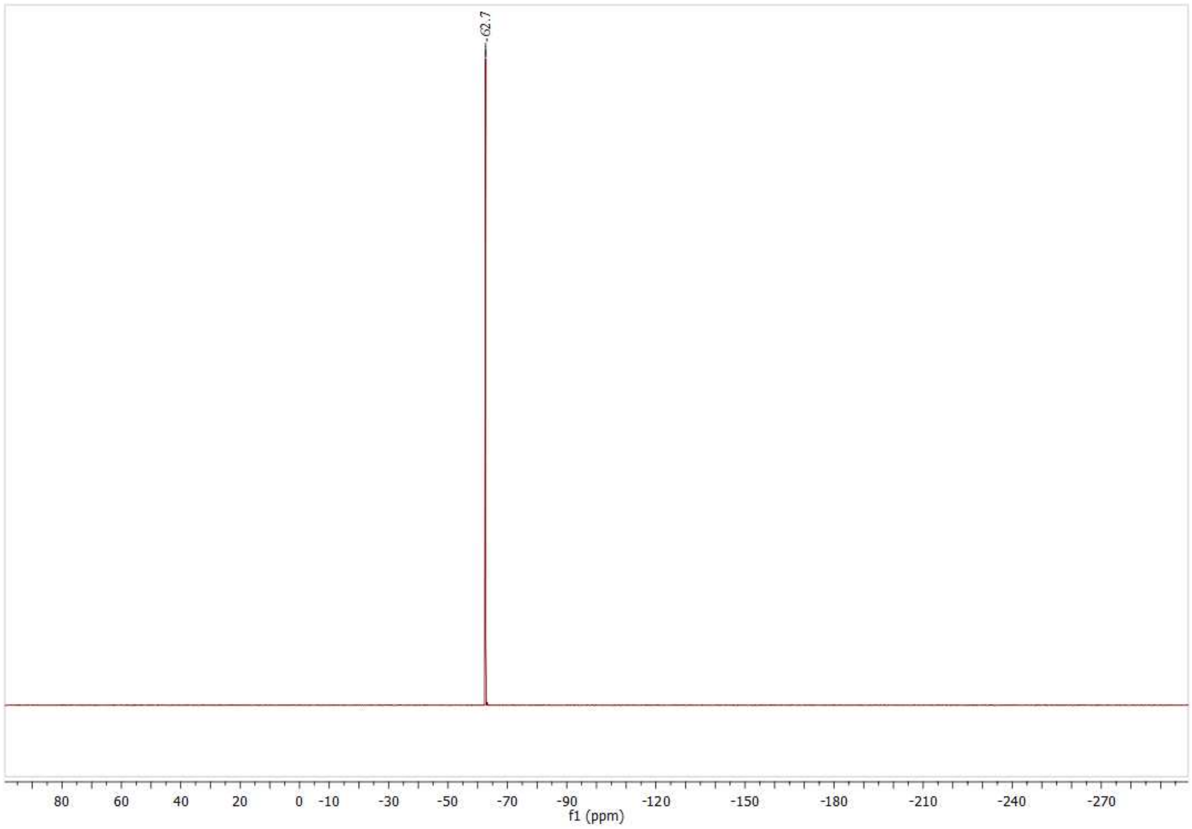
-62.7
80
60
40
20
0
-10
-30
-50
-70
-90
-120
-150
-180
-210
-240
-270
f1 (ppm)

Tetraethyl (2,3-bis(4-(trifluoromethyl)phenyl)butane-1,1,4,4-tetracarboxylate SYN

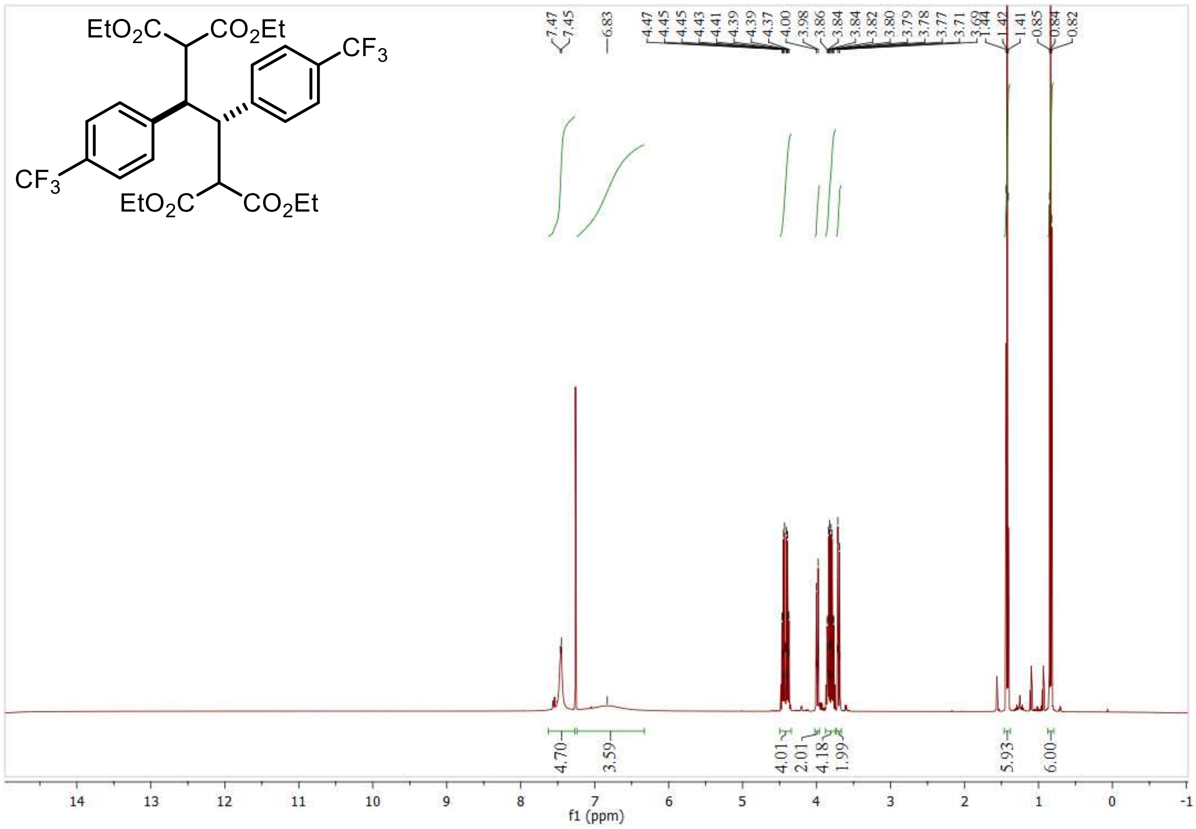

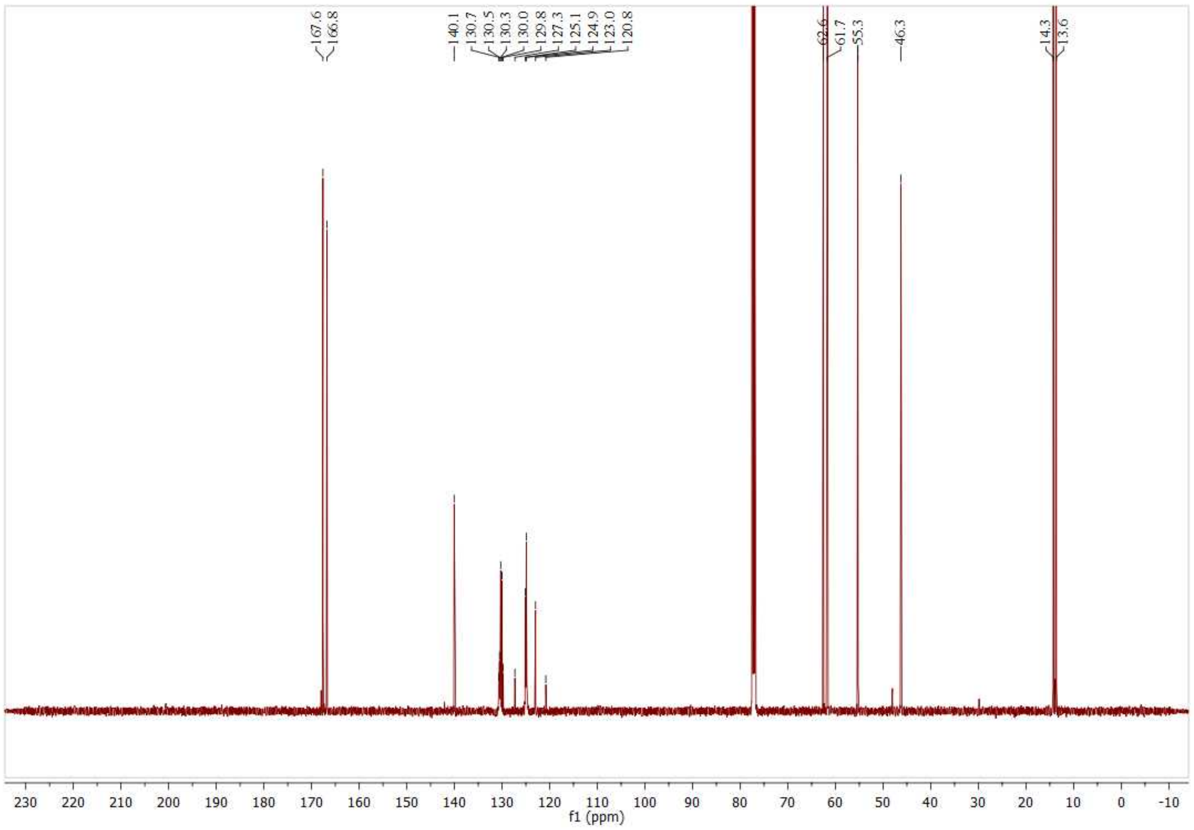

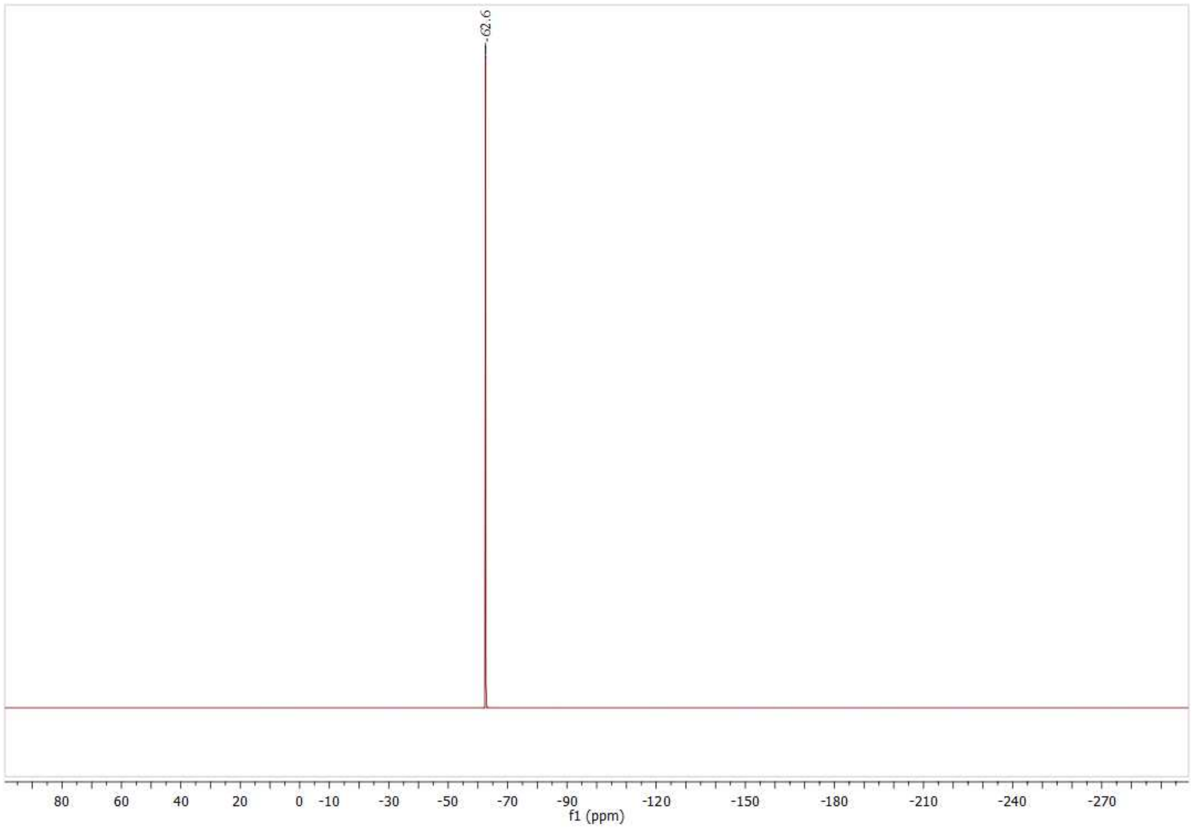

-62.6
80
60
40
20
0
-10
-30
-50
-70
-90
-120
-150
-180
-210
-240
-270
f1 (ppm)

Tetraethyl 2,3-di-p-tolylbutane-1,1,4,4-tetracarboxylate ANTI

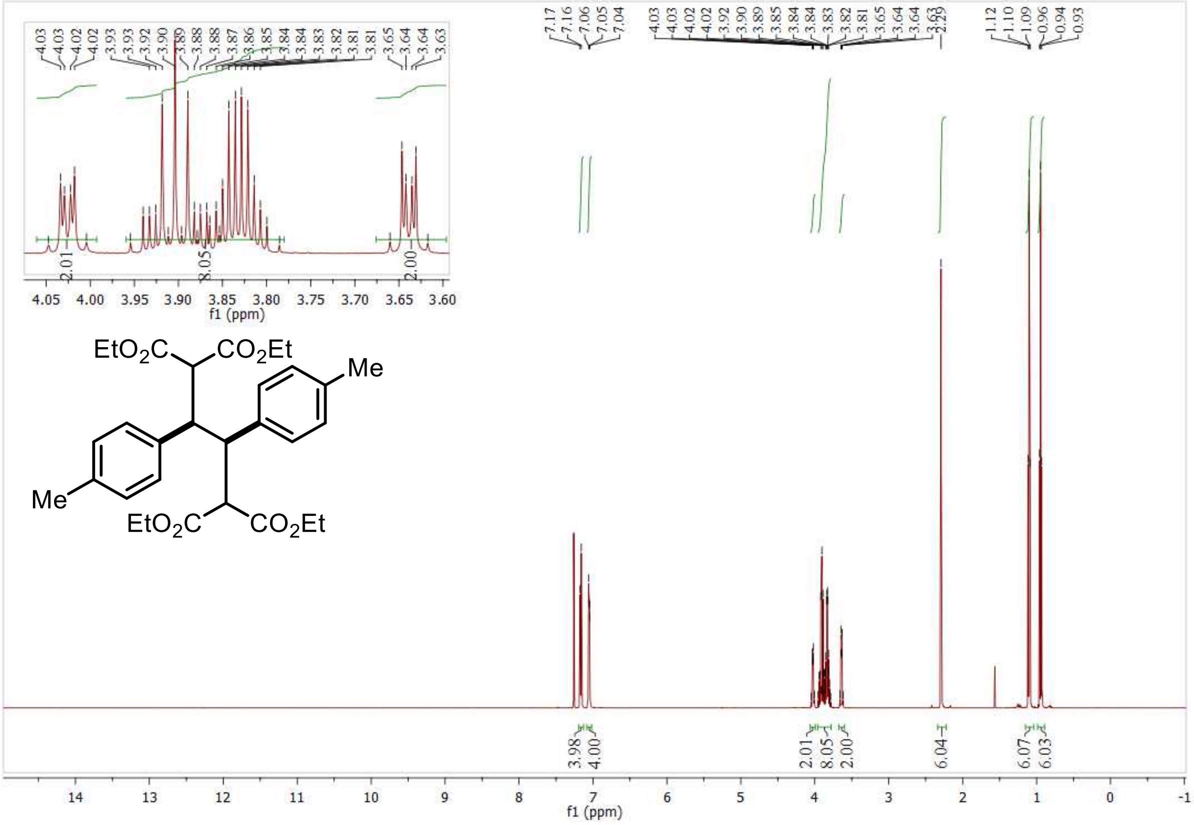

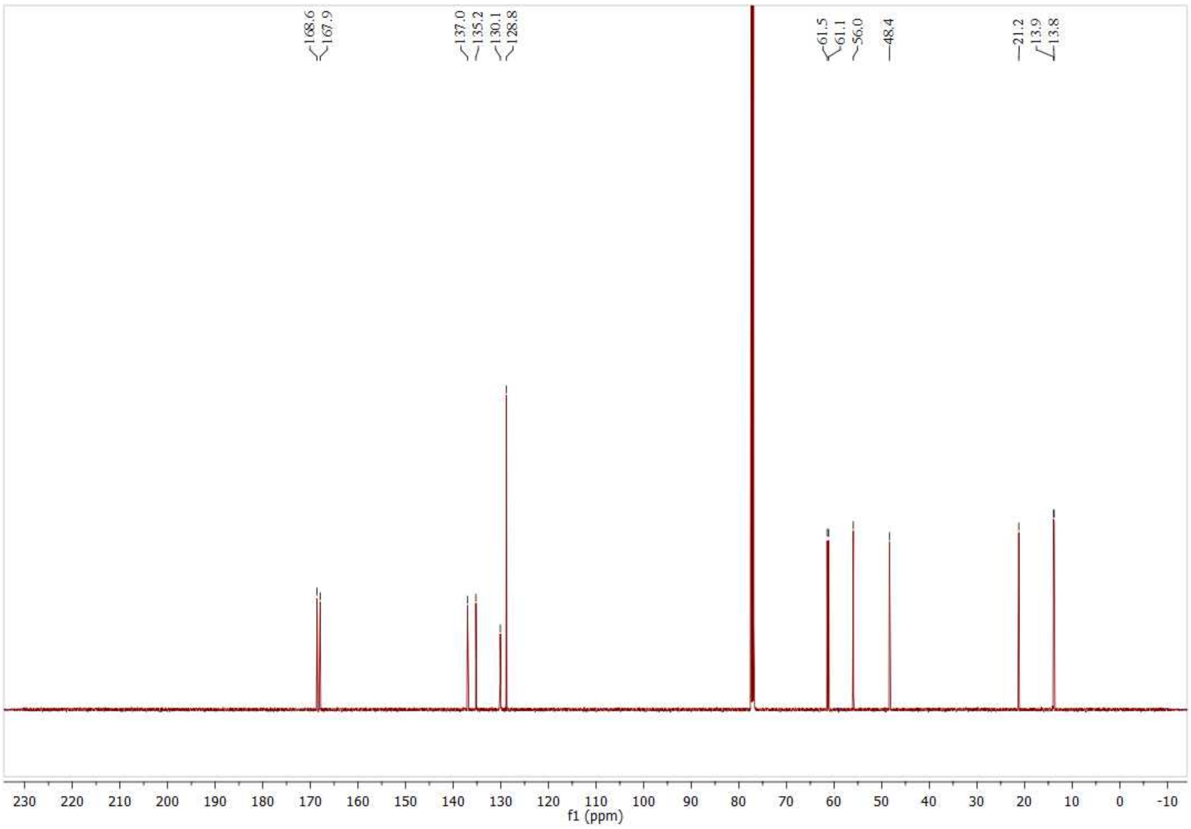

Tetraethyl 2,3-di-p-tolylbutane-1,1,4,4-tetracarboxylate SYN

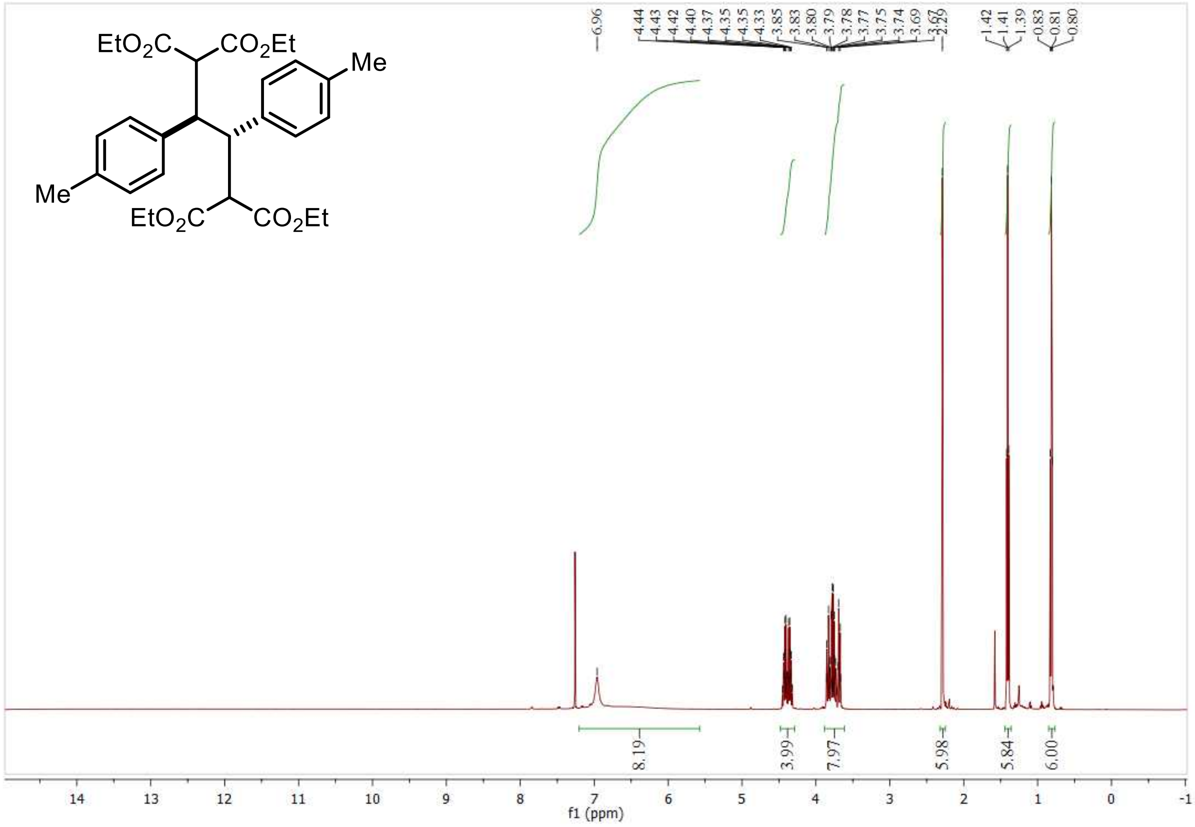

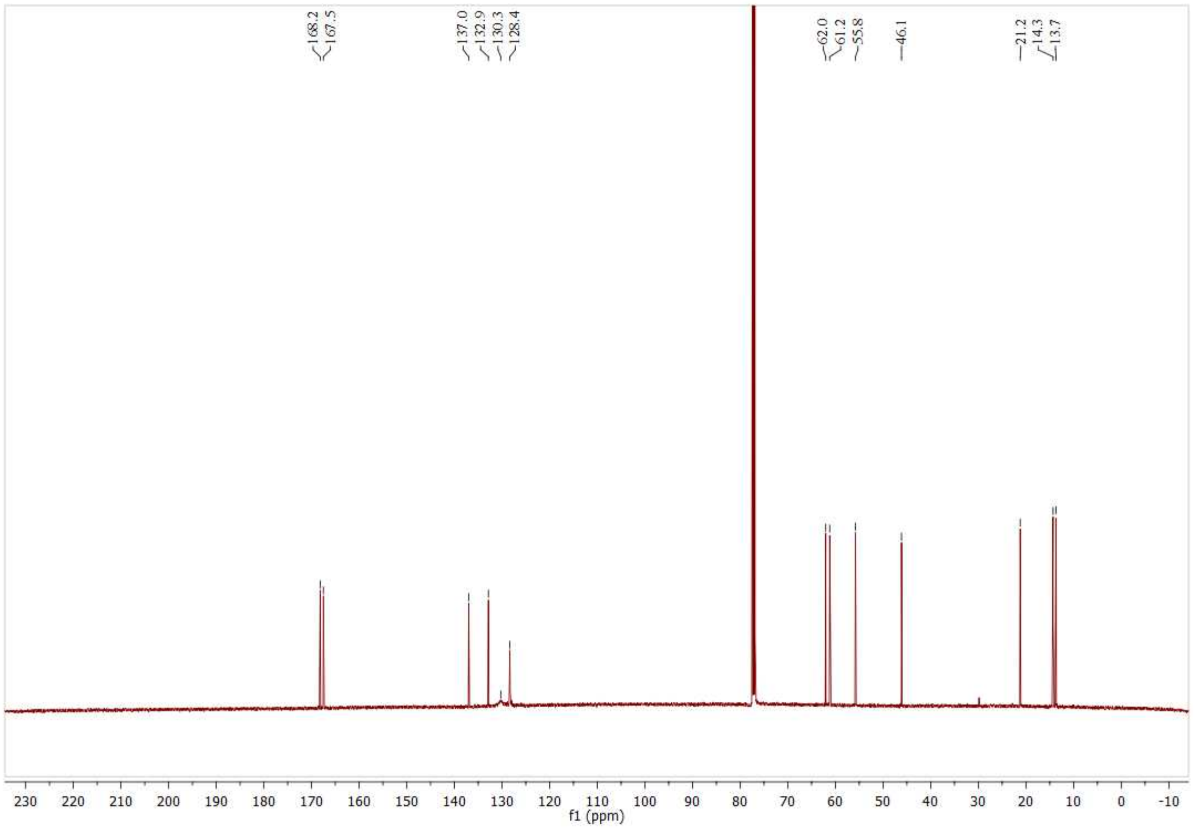

Tetraethyl 2,3-bis(4-methoxyphenyl)butane-1,1,4,4-tetracarboxylate ANTI

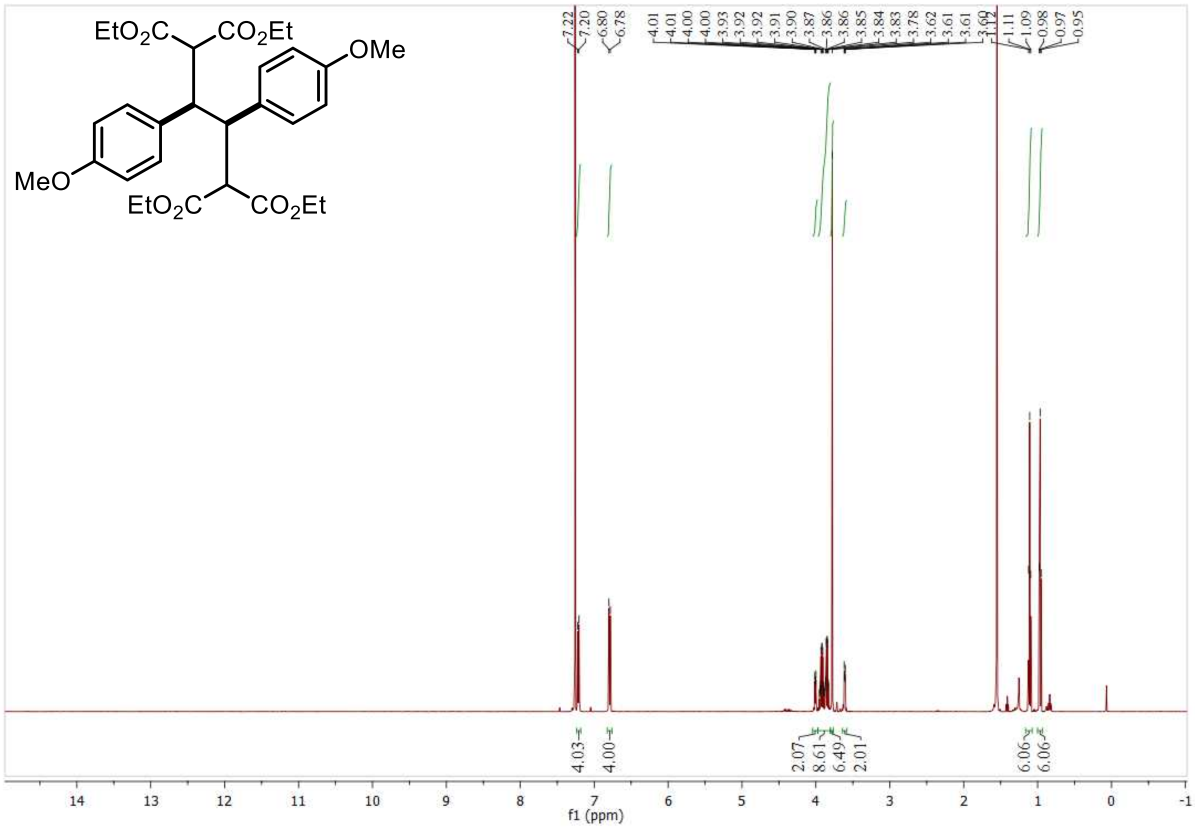

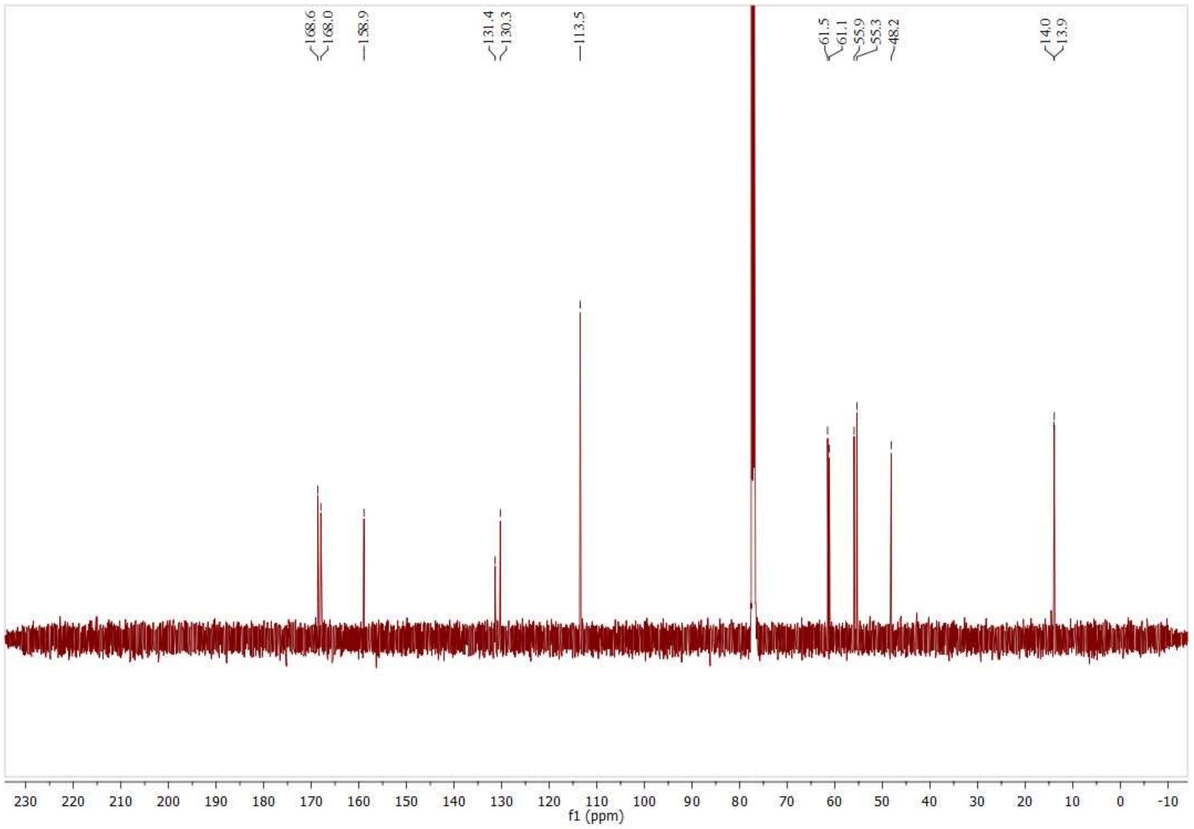

Tetraethyl 2,3-bis(4-methoxyphenyl)butane-1,1,4,4-tetracarboxylate SYN

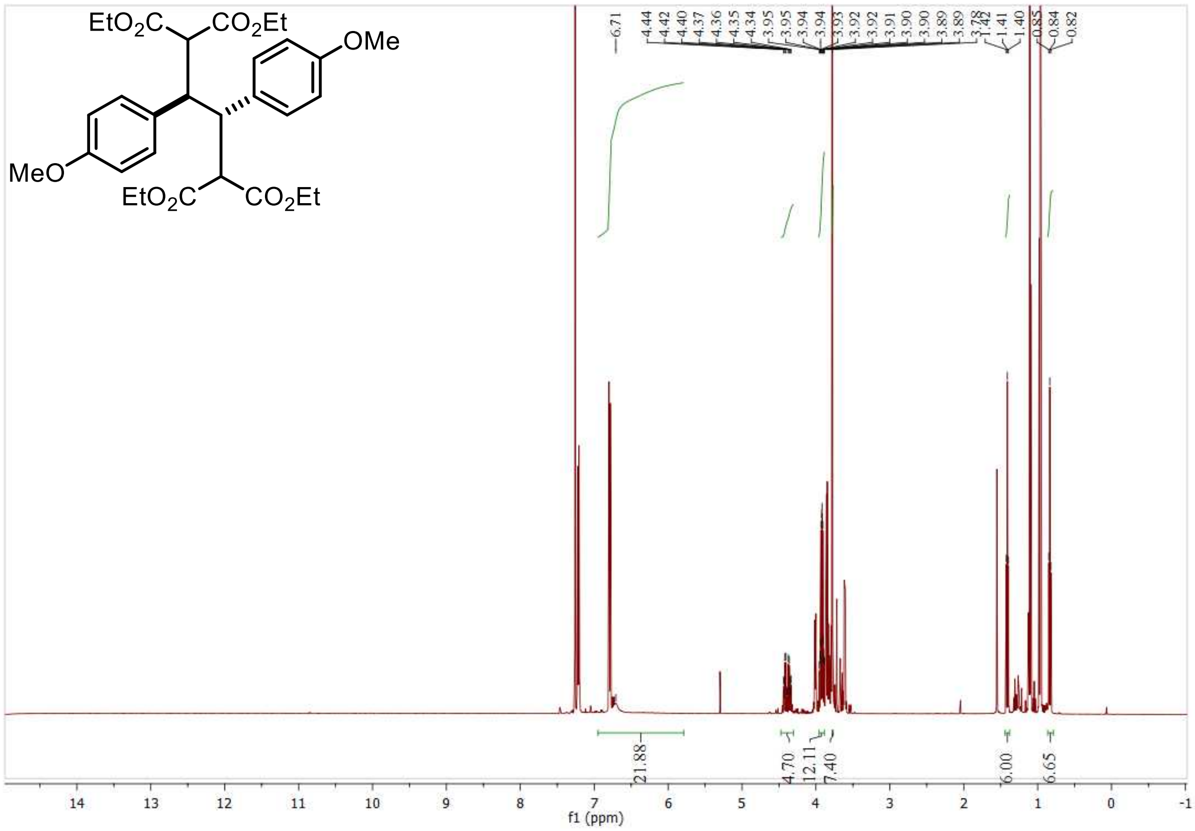

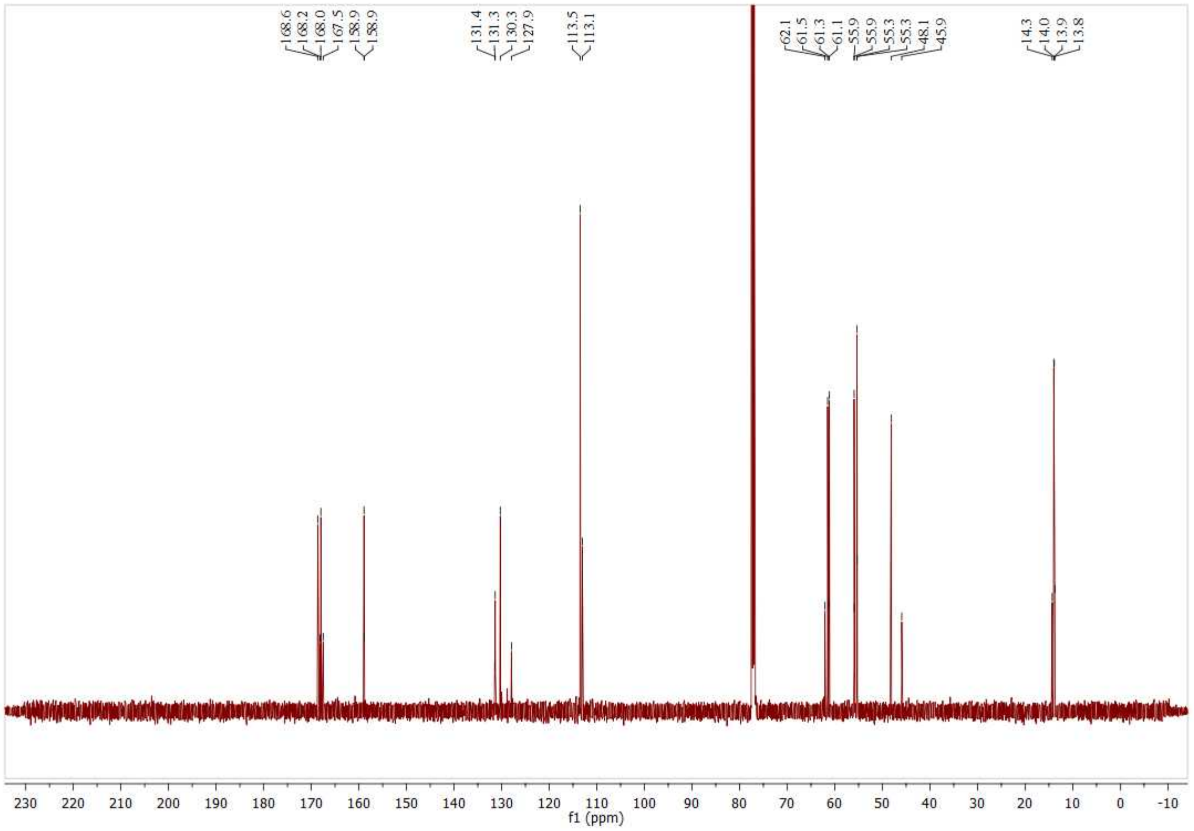

Tetraethyl 2,3-bis(4-(methoxycarbonyl)phenyl)butane-1,1,4,4-tetracarboxylate ANTI

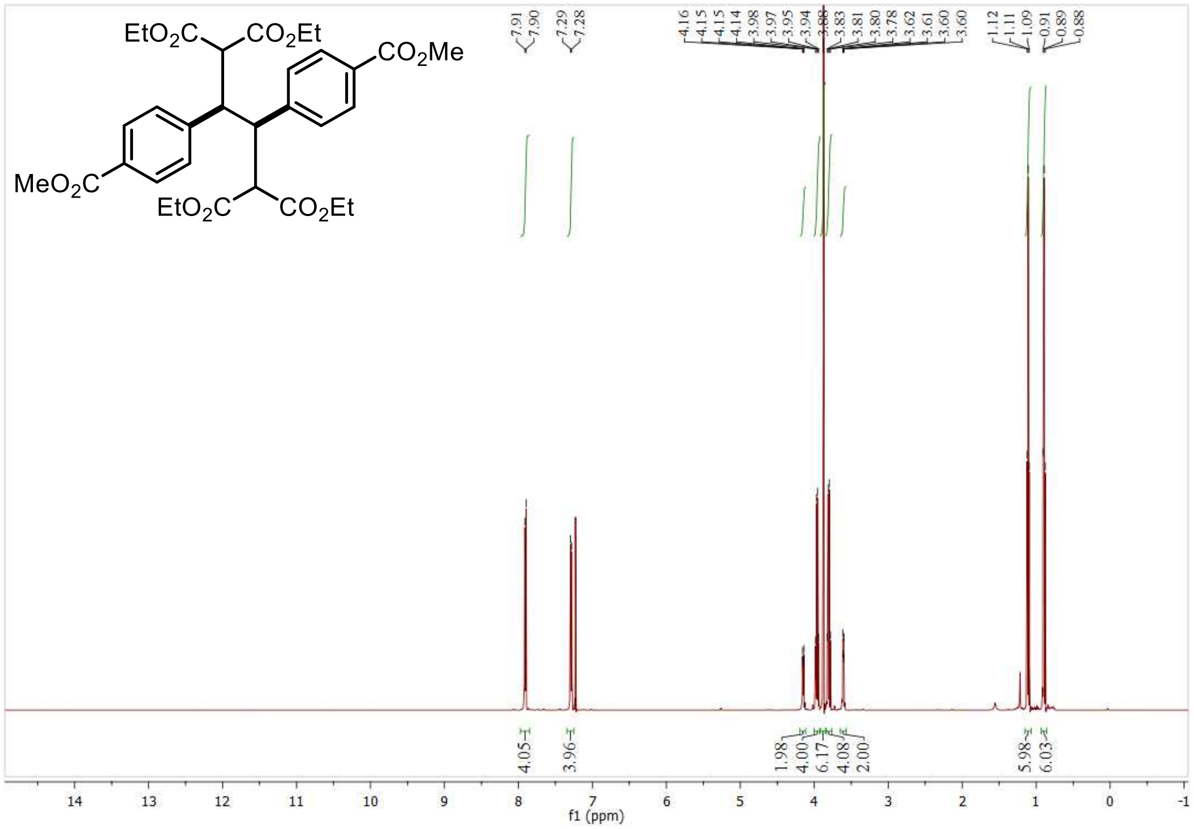

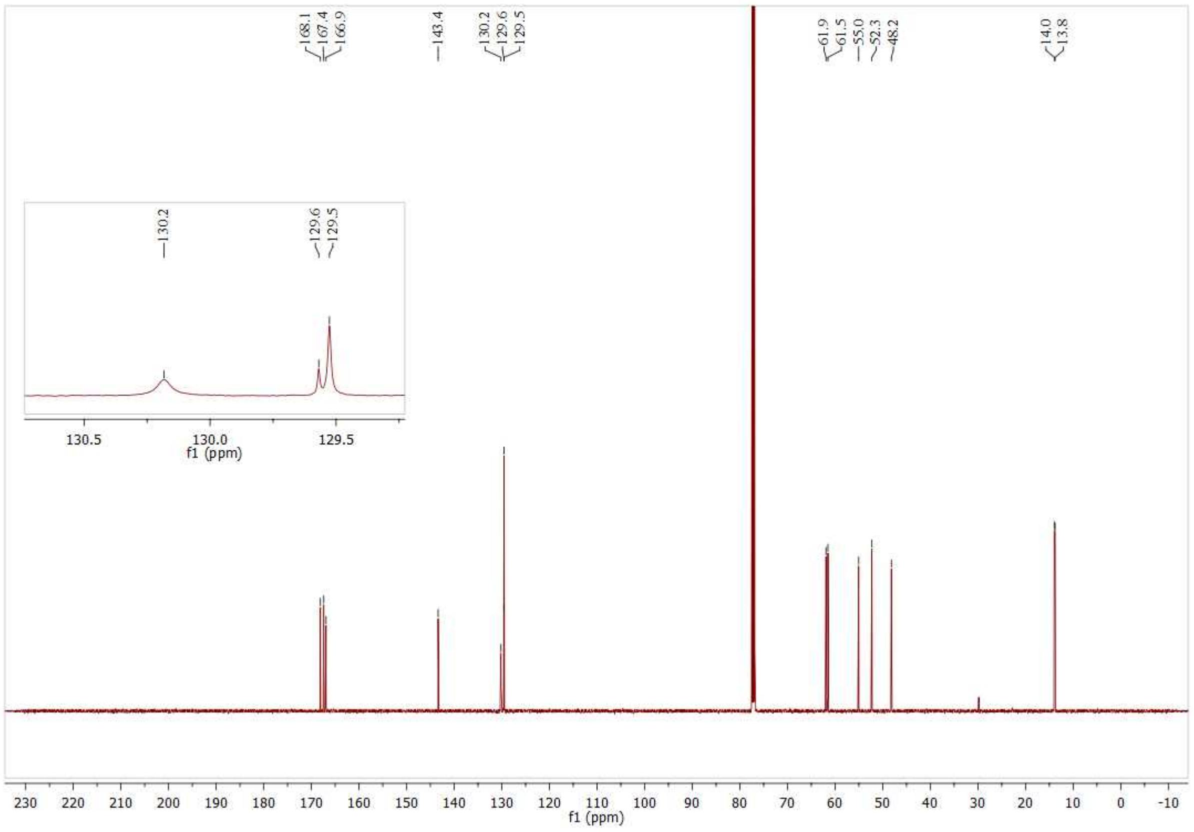

Tetraethyl 2,3-bis(4-(methoxycarbonyl)phenyl)butane-1,1,4,4-tetracarboxylate SYN

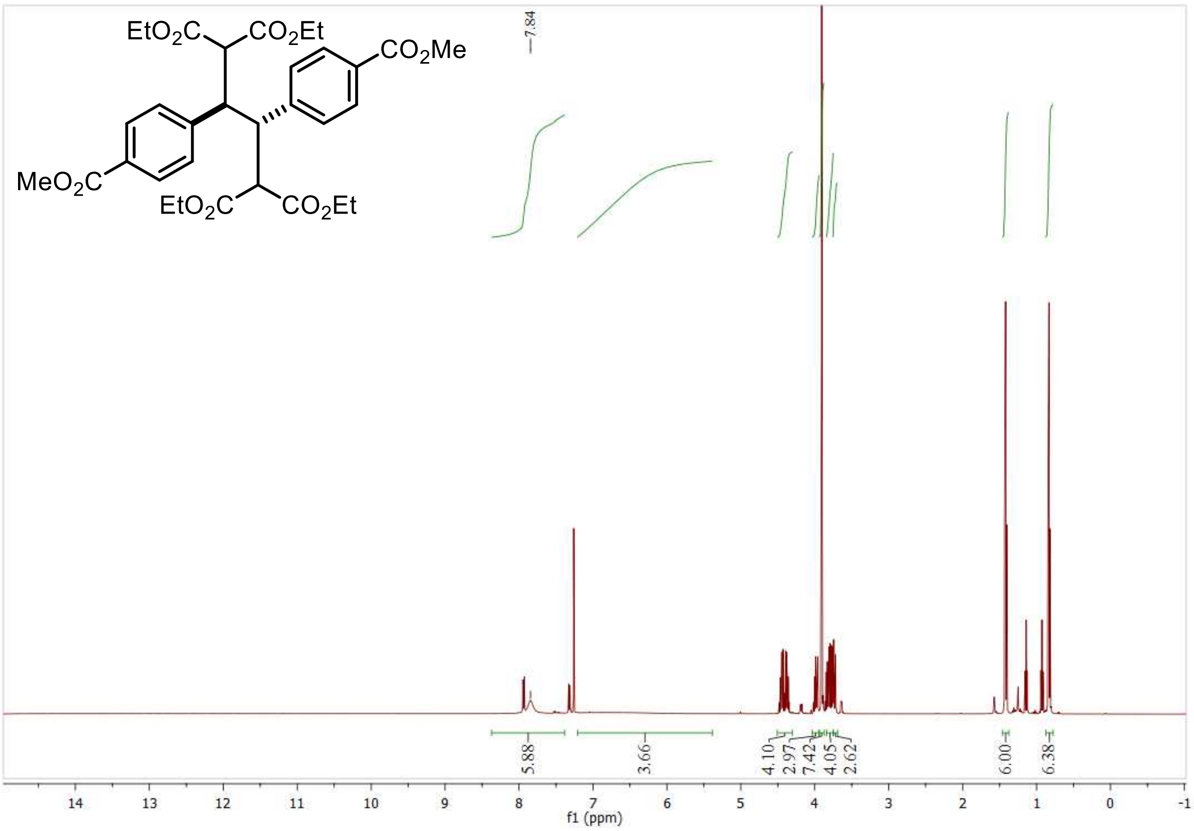

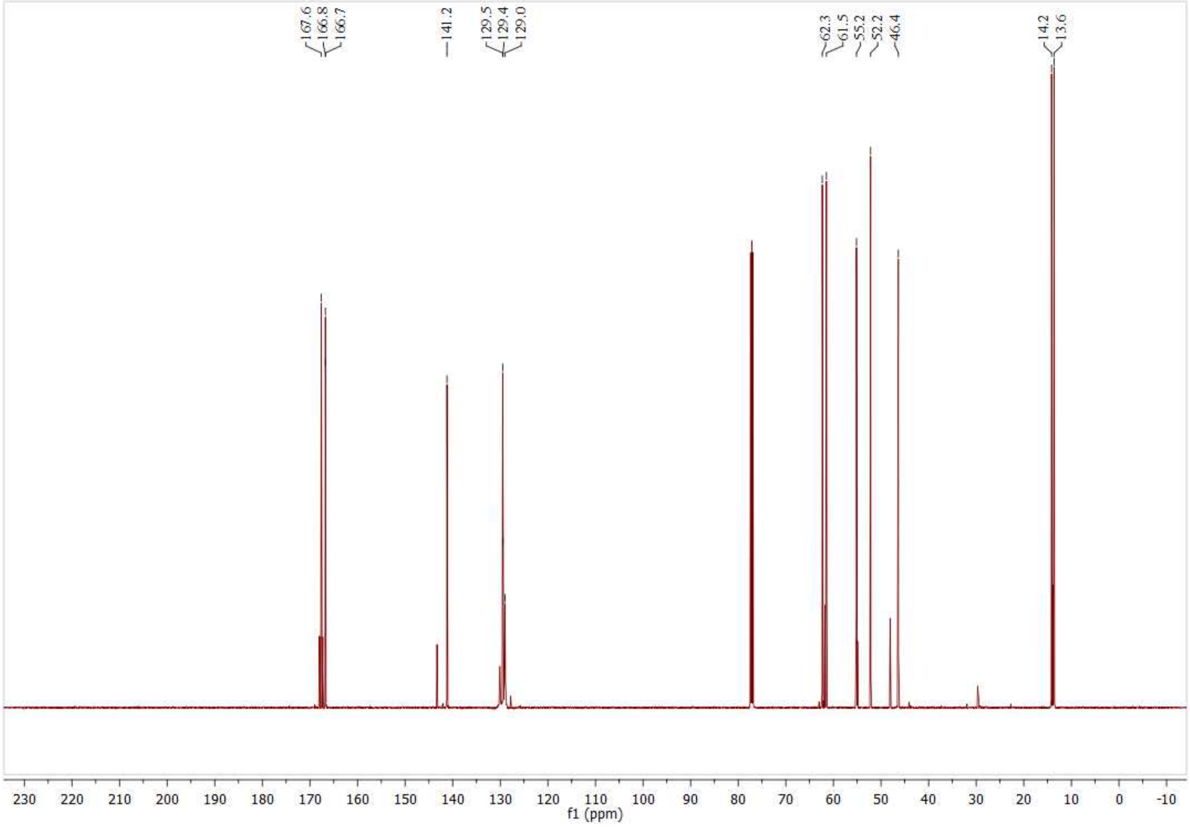

Tetraethyl bis(4-cyanophenyl)butane-1,1,4,4-tetracarboxylate ANTI

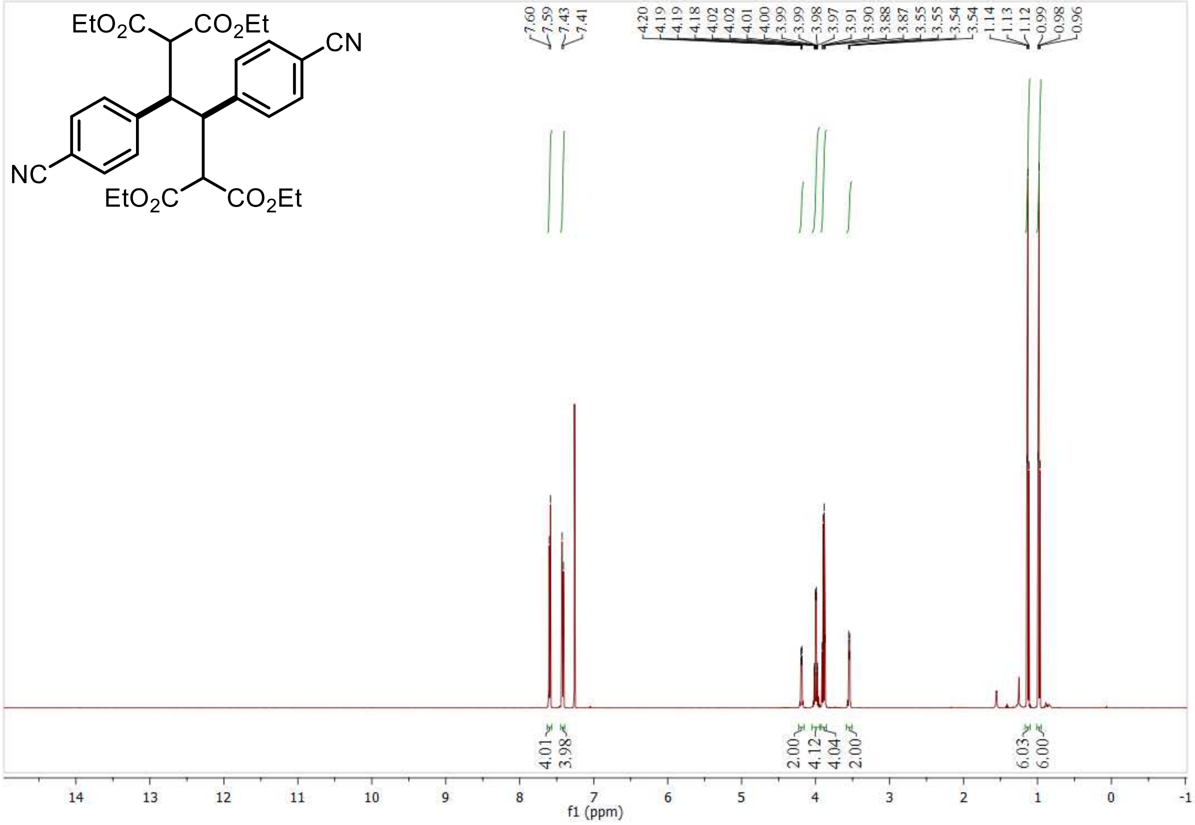

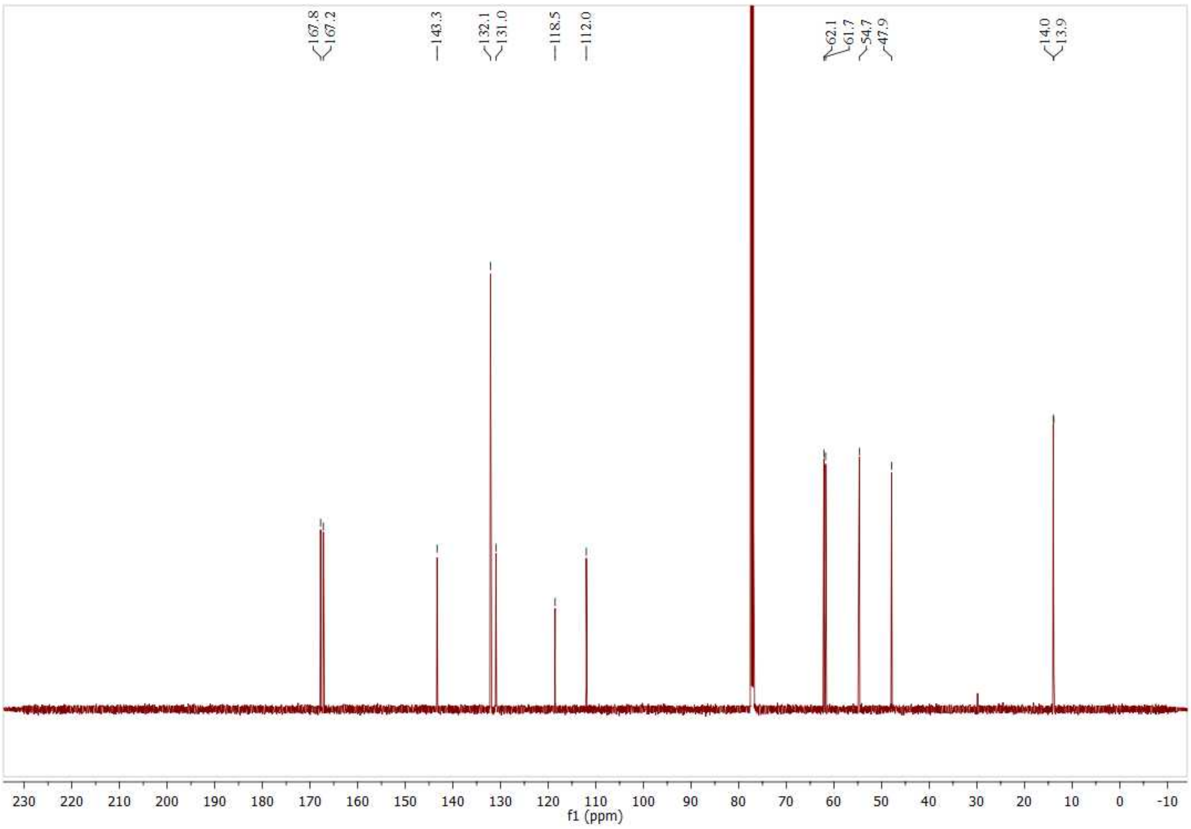

Tetraethyl bis(4-cyanophenyl)butane-1,1,4,4-tetracarboxylate SYN

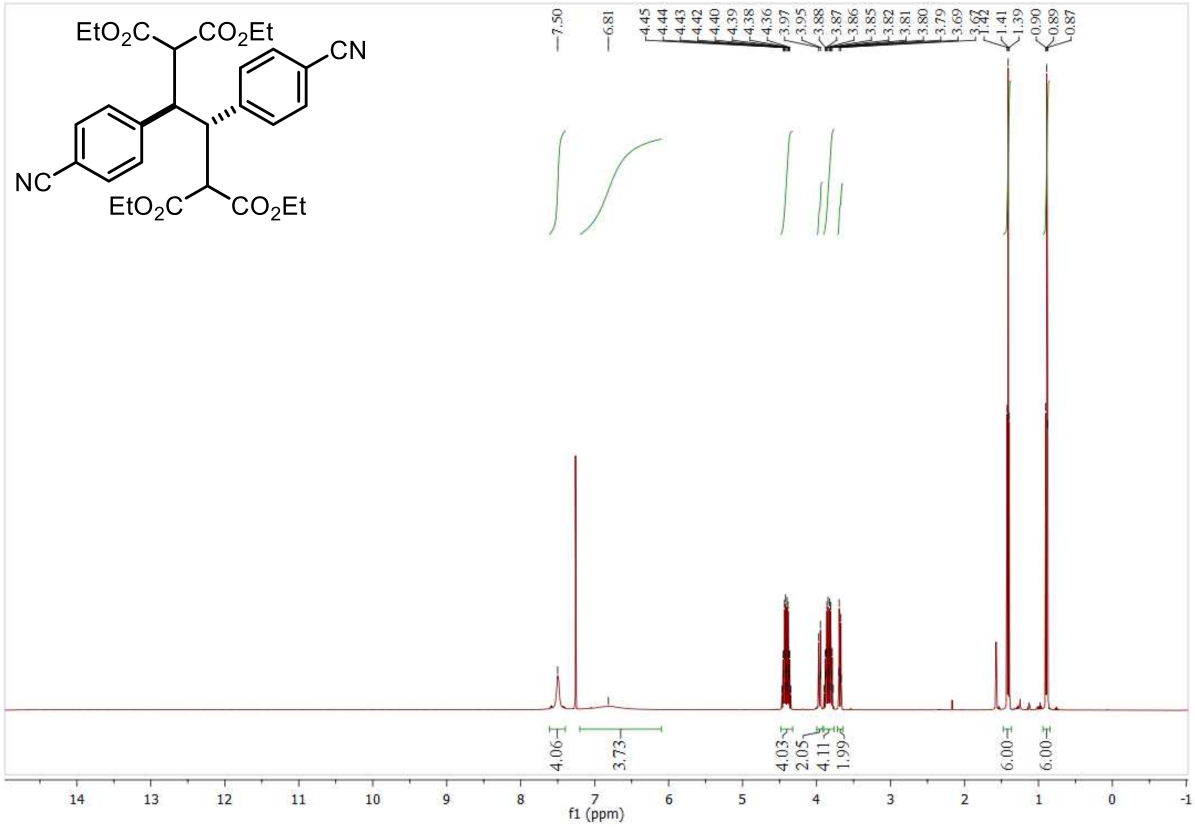

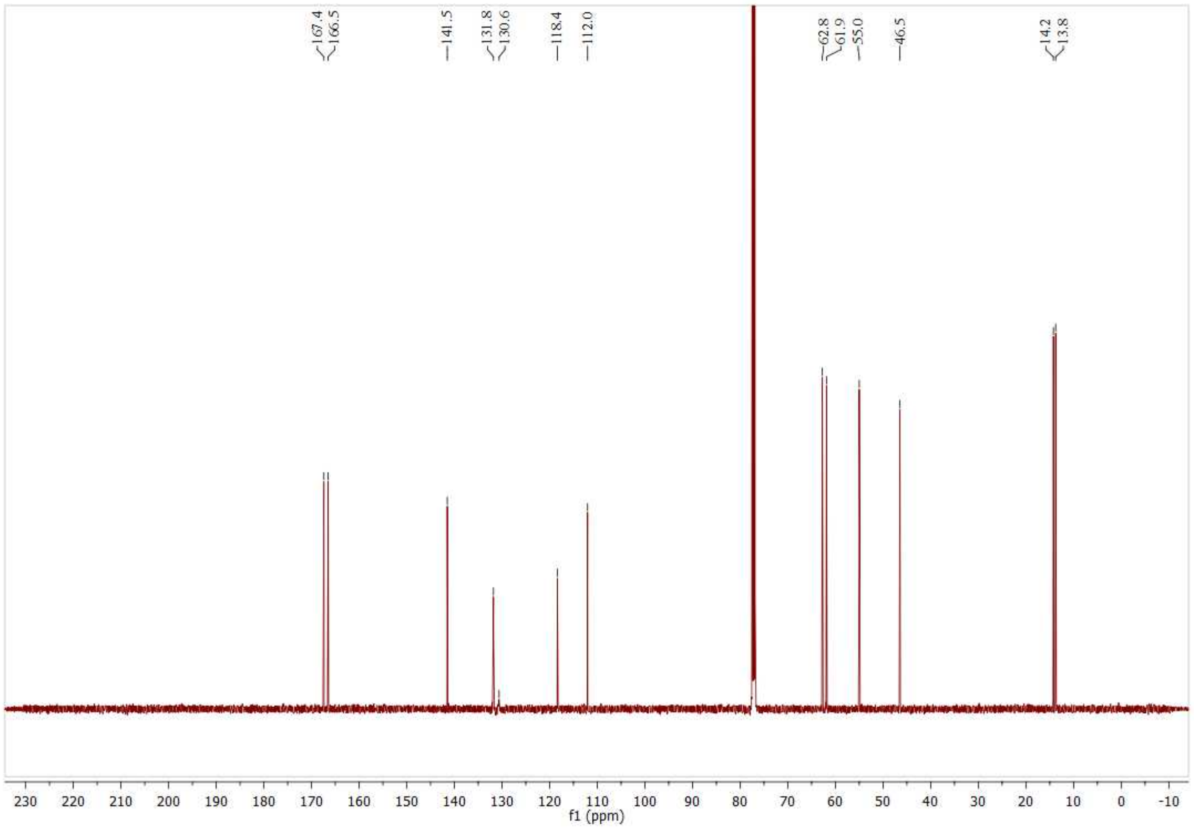

Tetraethyl 2,3-bis(4-chlorophenyl)butane-1,1,4,4-tetracarboxylate ANTI

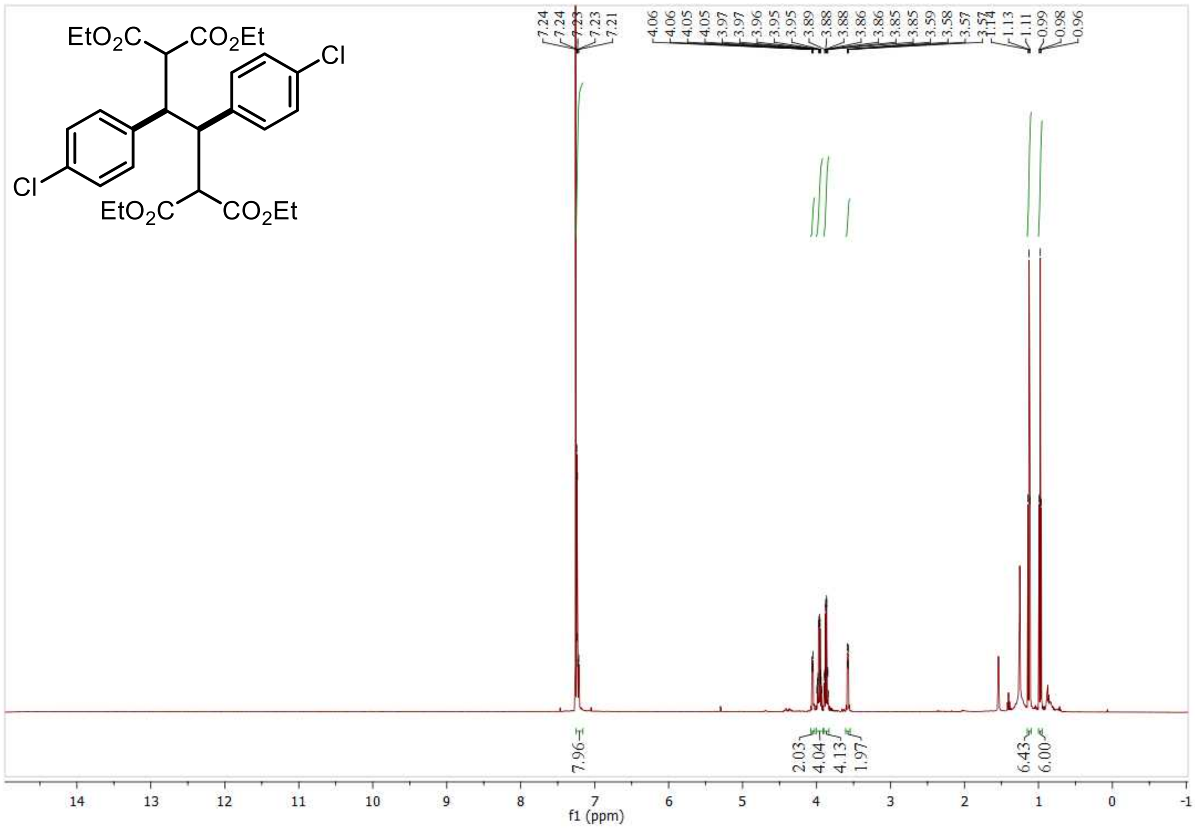

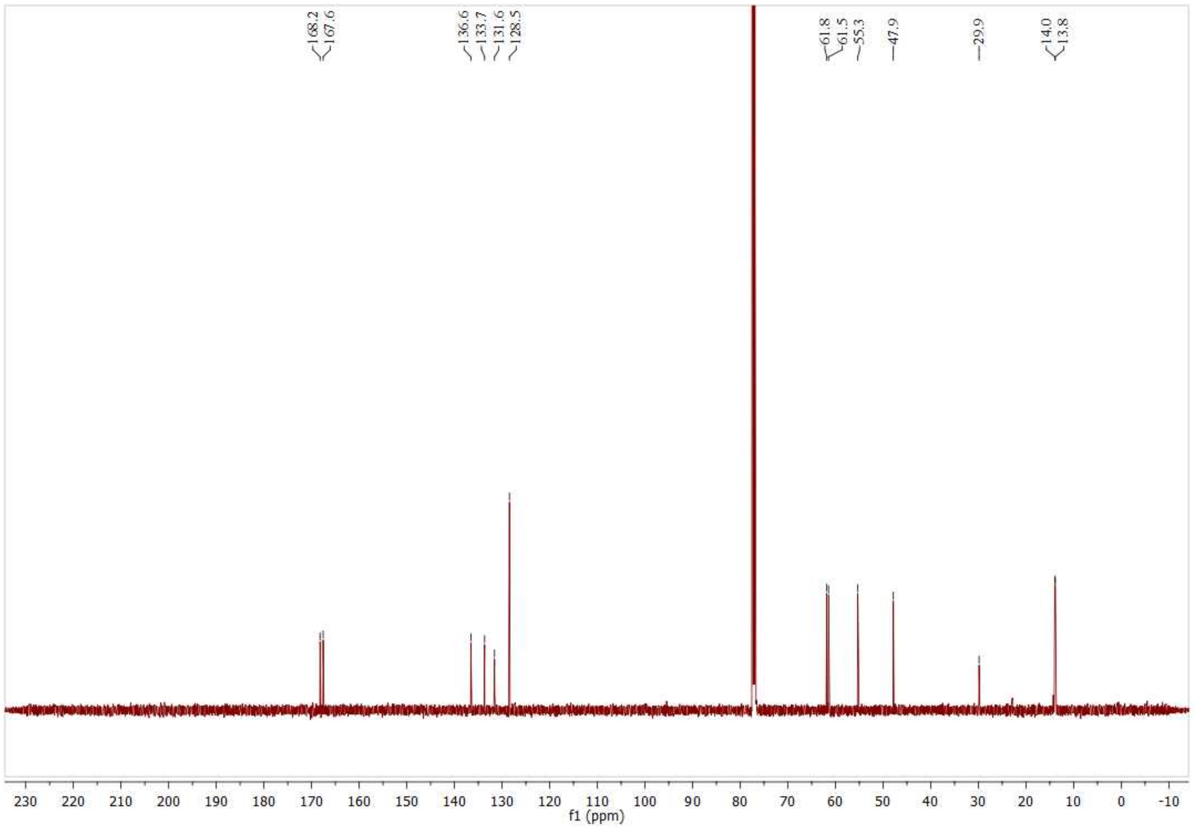

Tetraethyl 2,3-bis(4-chlorophenyl)butane-1,1,4,4-tetracarboxylate SYN

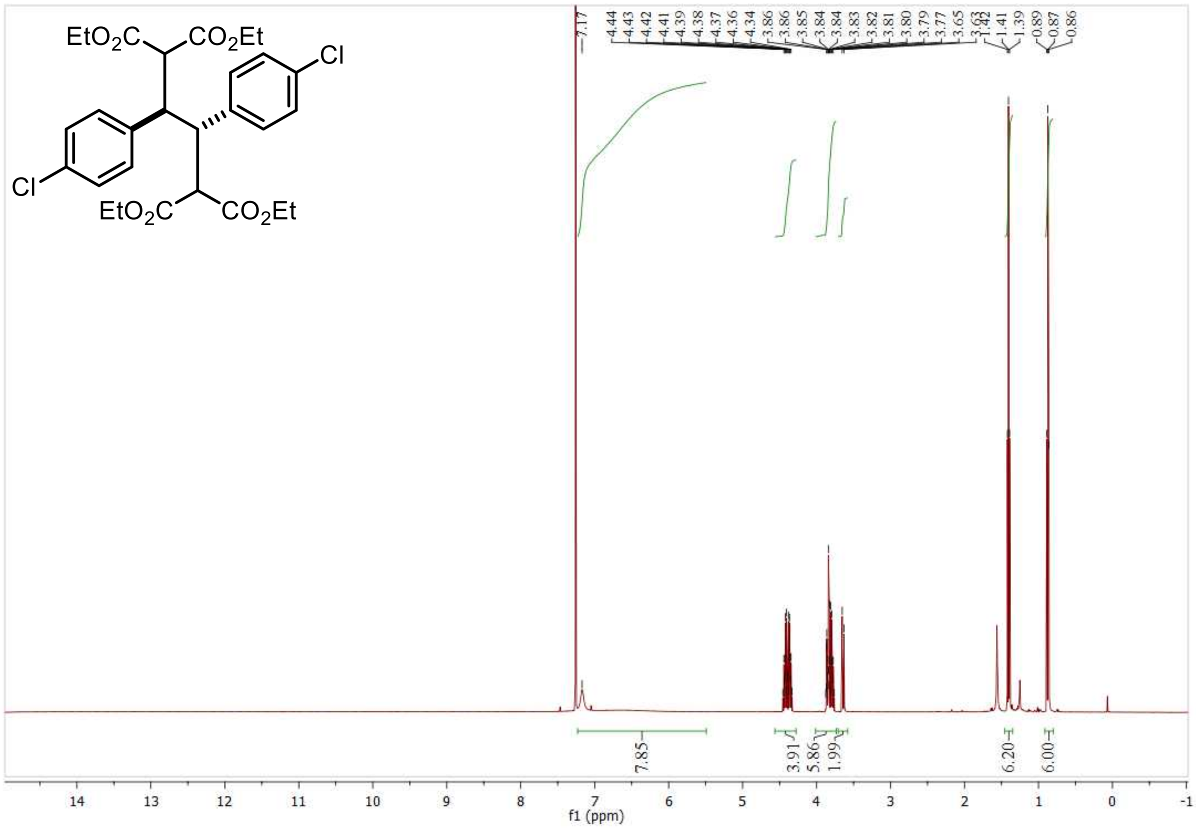

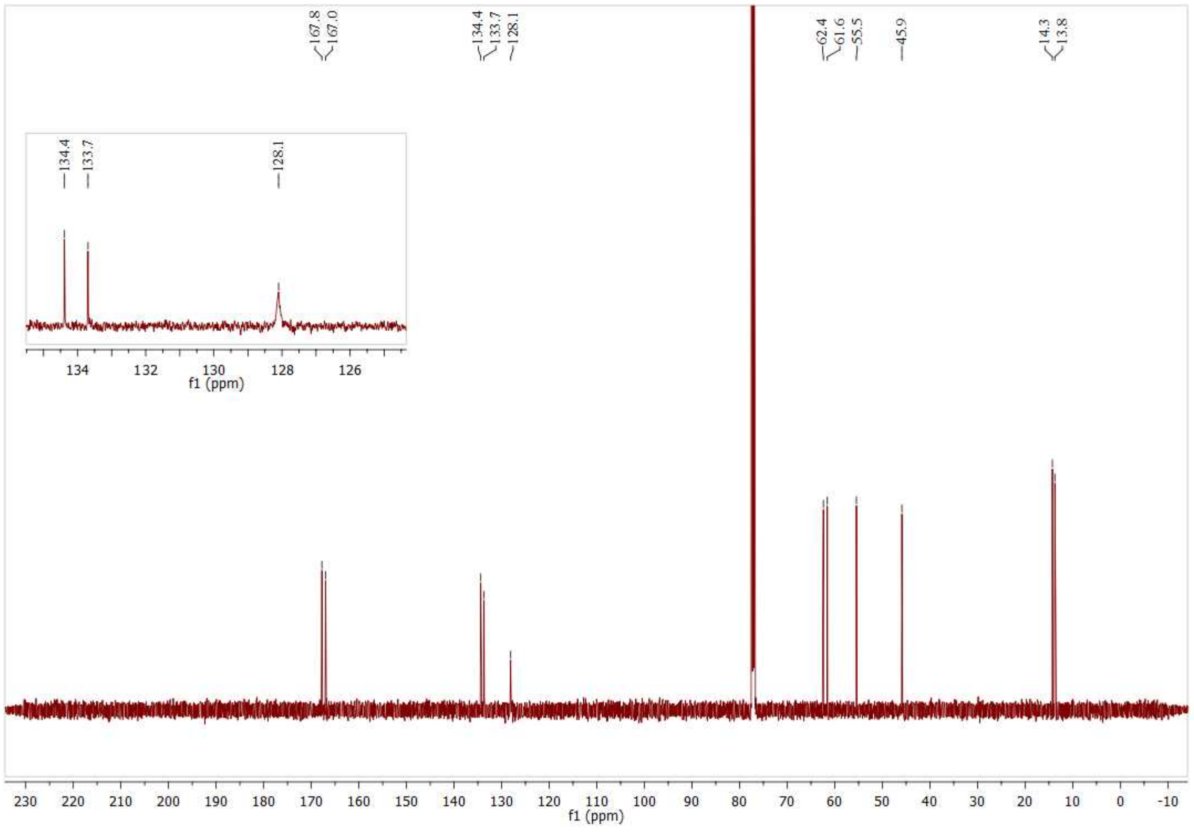

Tetramethyl 2,3-bis(4-fluorophenyl)butane-1,1,4,4-tetracarboxylate ANTI

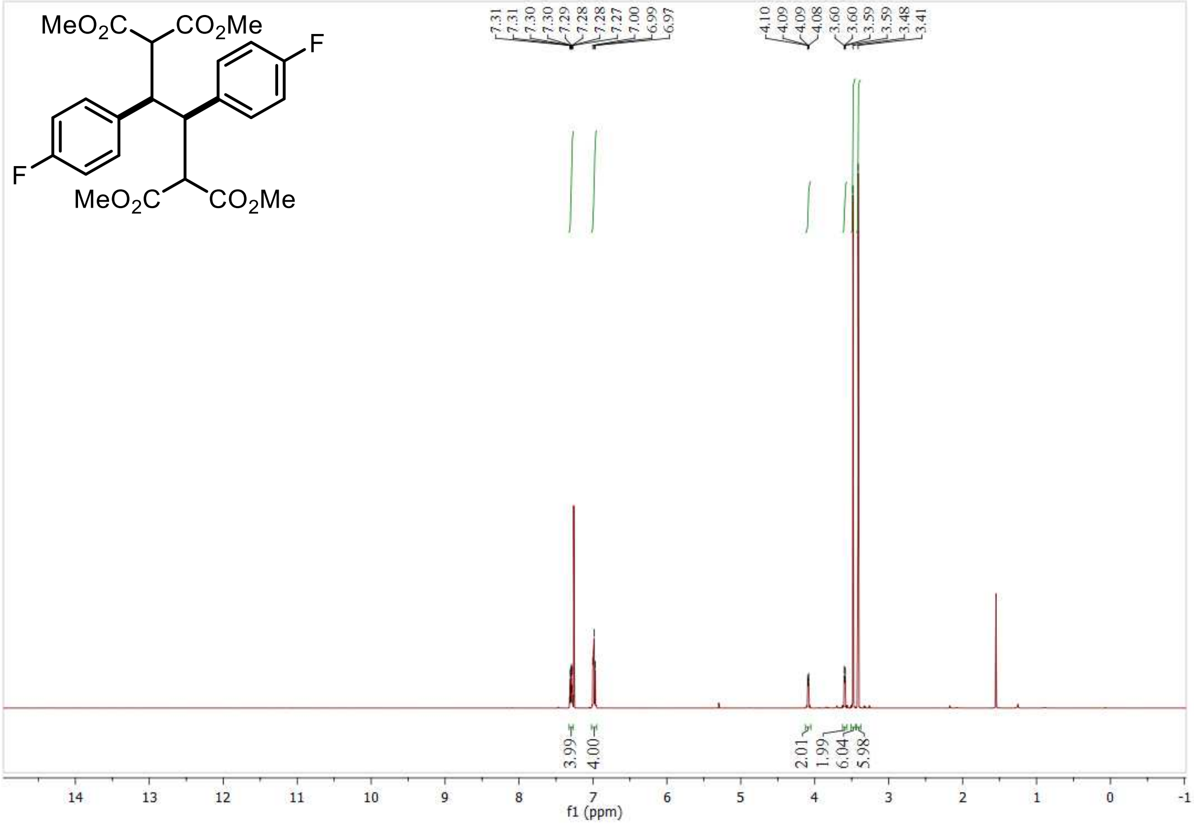

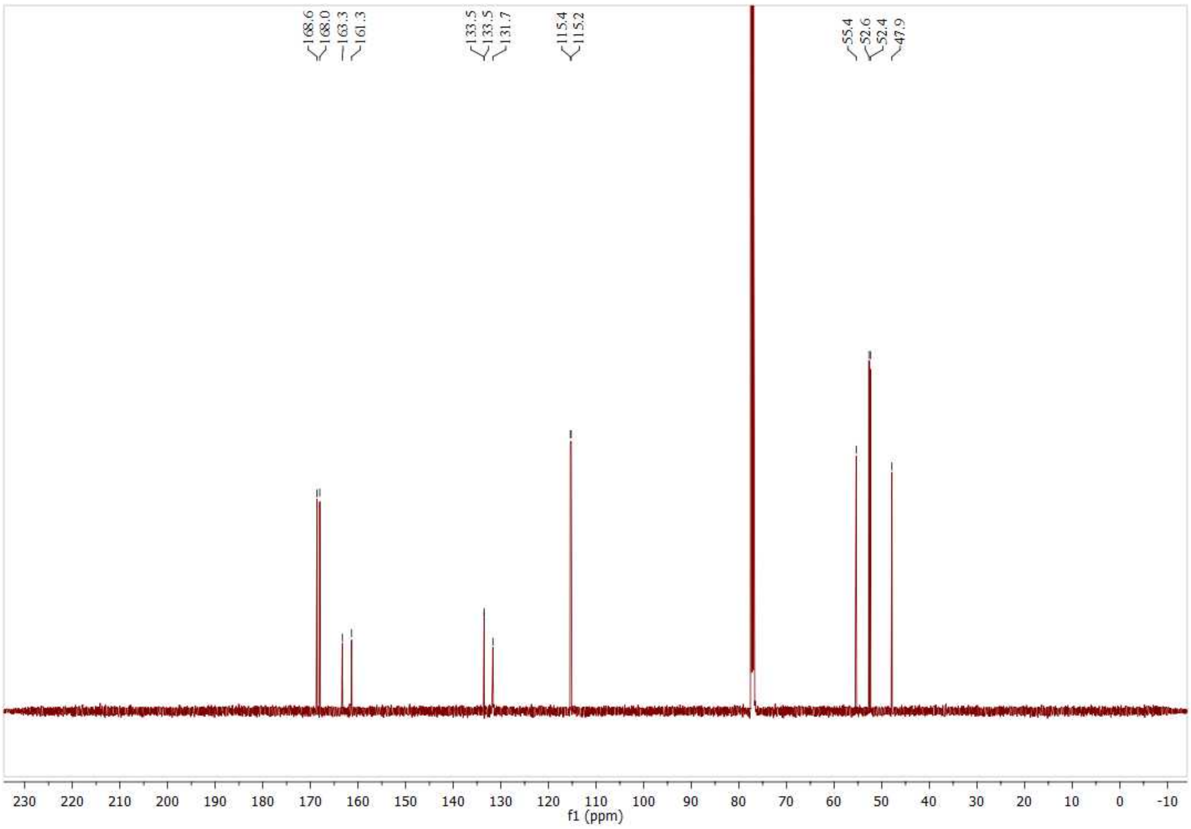

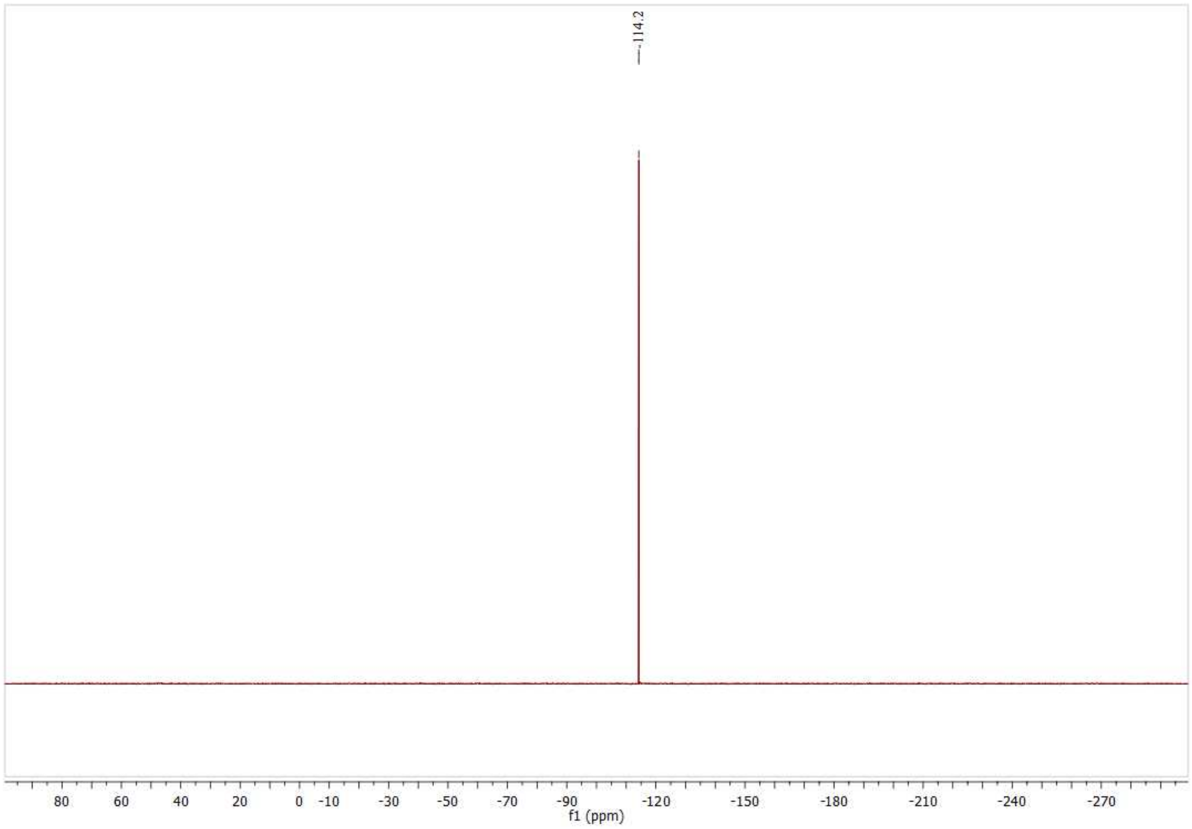
-114.2
80
60
40
20
0
-10
-30
-50
-70
-90
-120
-150
-180
-210
-240
-270
f1 (ppm)

Tetramethyl 2,3-bis(4-fluorophenyl)butane-1,1,4,4-tetracarboxylate SYN

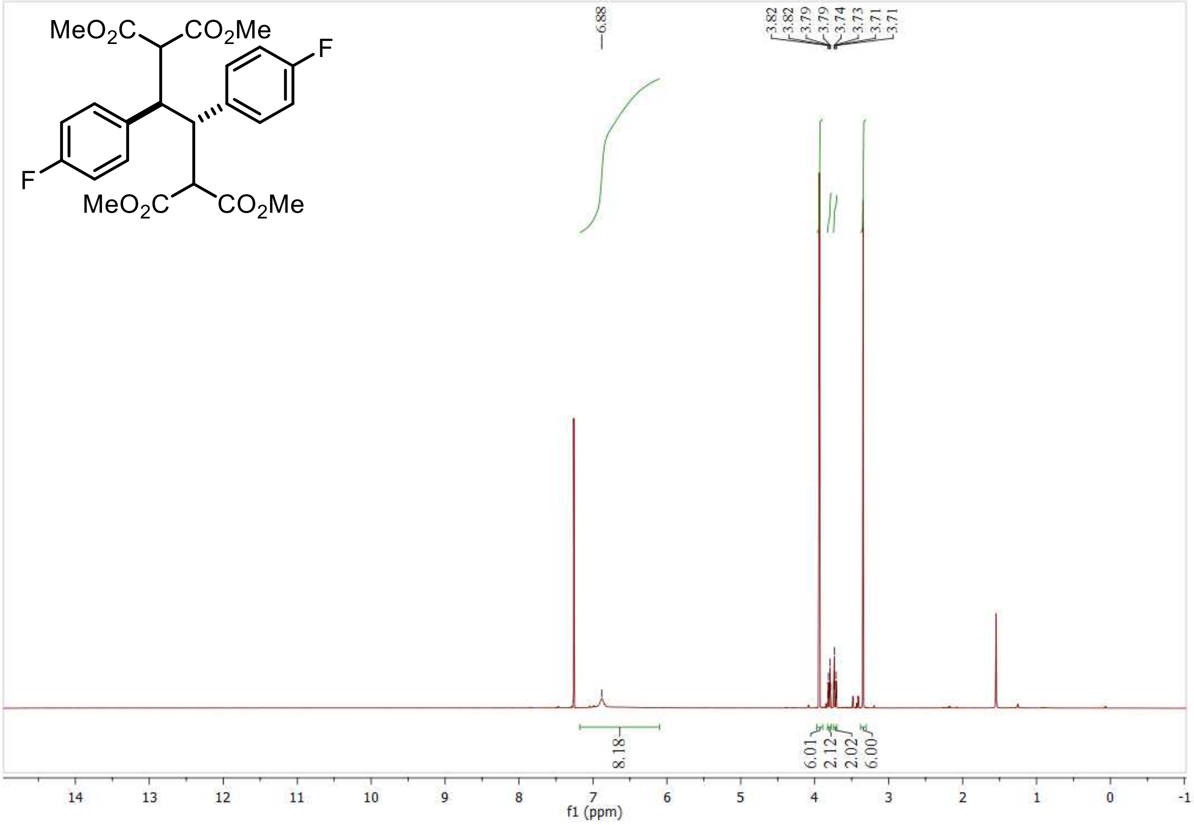

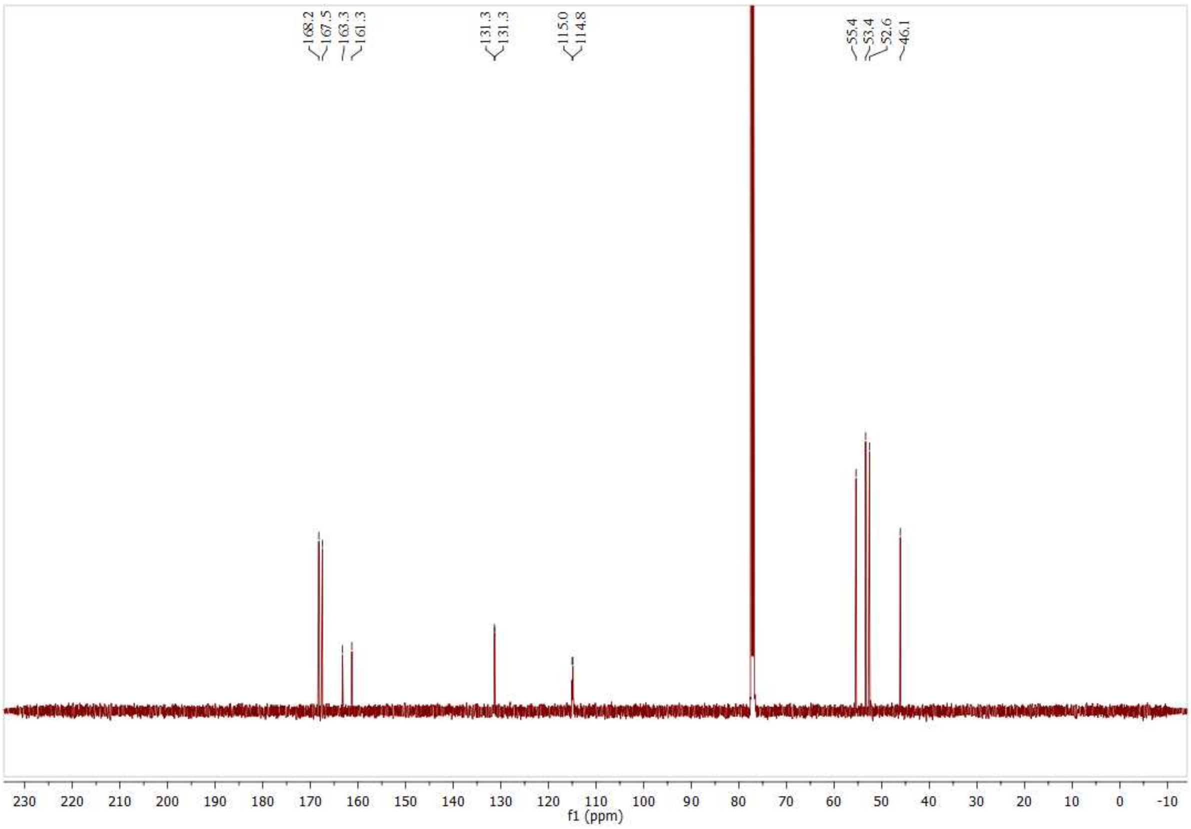

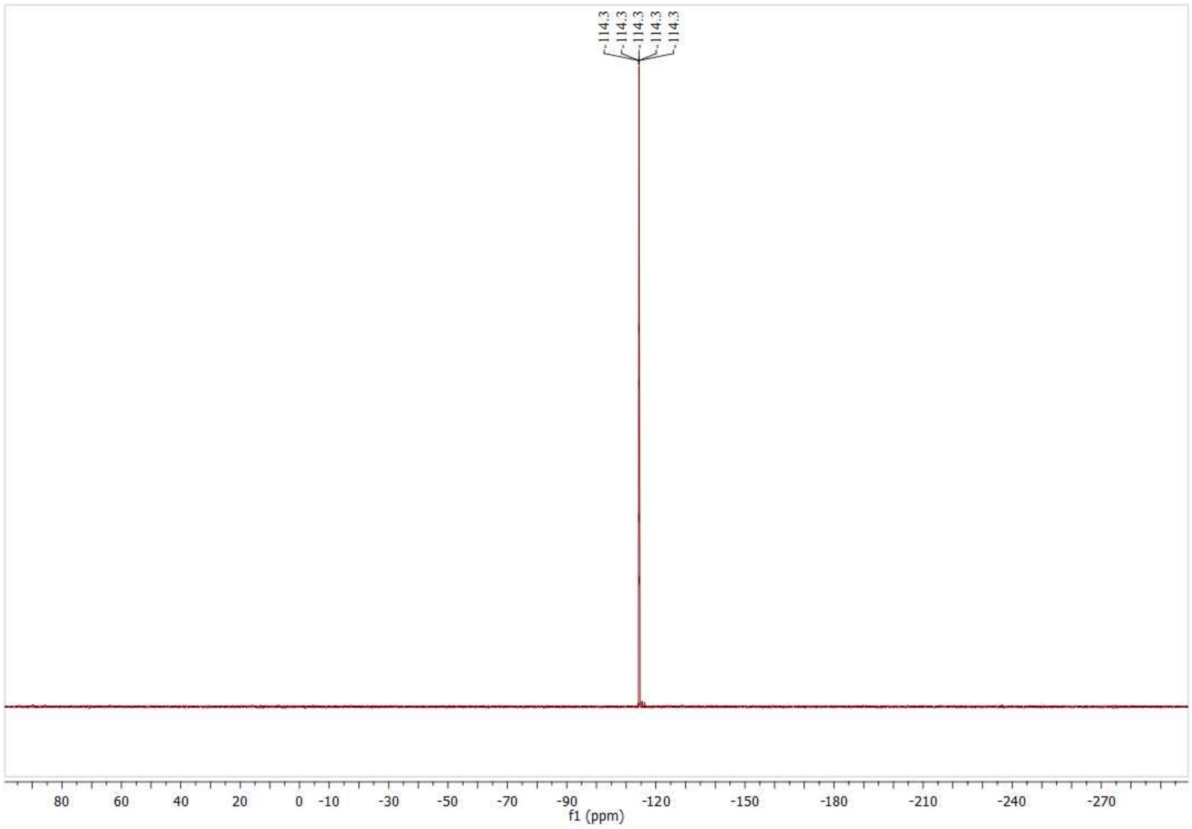
-114.3
-114.3
-114.3
-114.3
-114.3
80 60 40 20 0 -10 -30 -50 -70 -90 -120 -150 -180 -210 -240 -270
f1 (ppm)

Tetraisopropyl 2,3-bis(4-fluorophenyl)butane-1,1,4,4-tetracarboxylate ANTI

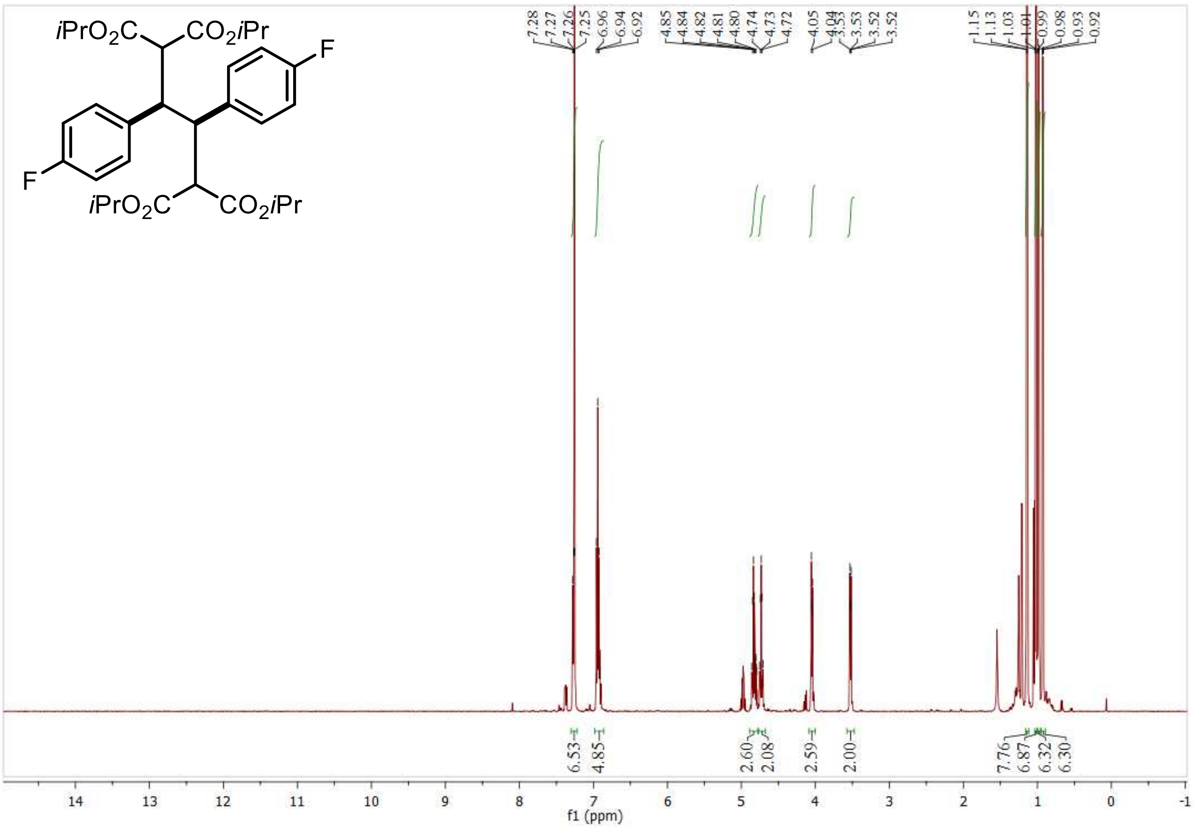

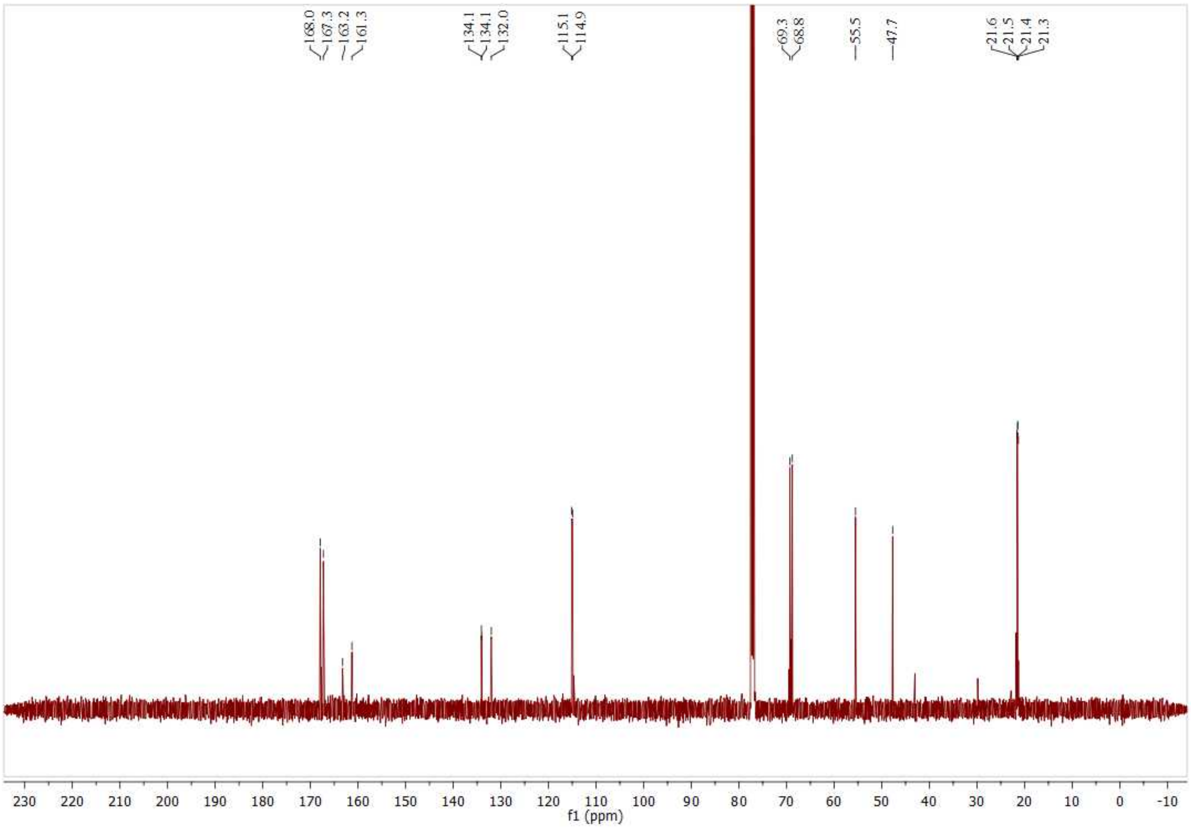

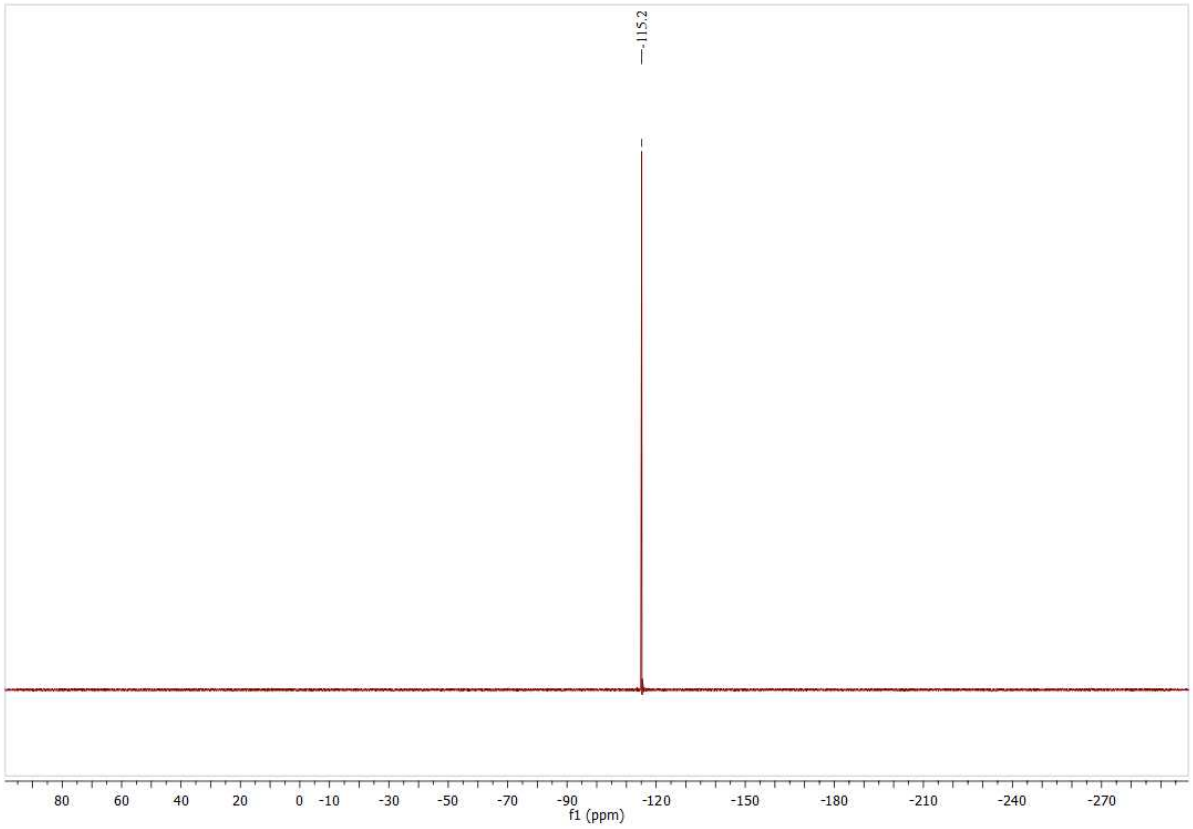
-115.2
80
60
40
20
0
-10
-30
-50
-70
-90
-120
-150
-180
-210
-240
-270
f1 (ppm)

Tetraisopropyl 2,3-bis(4-fluorophenyl)butane-1,1,4,4-tetracarboxylate SYN

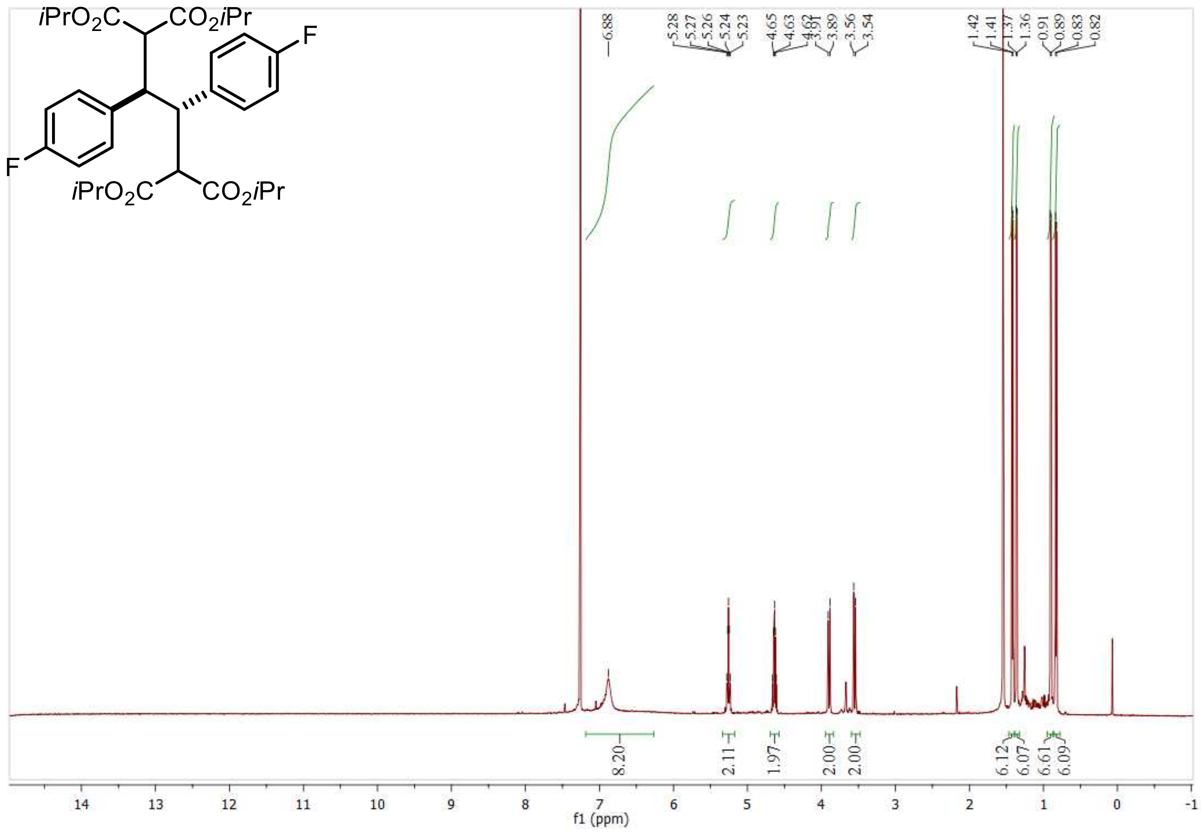

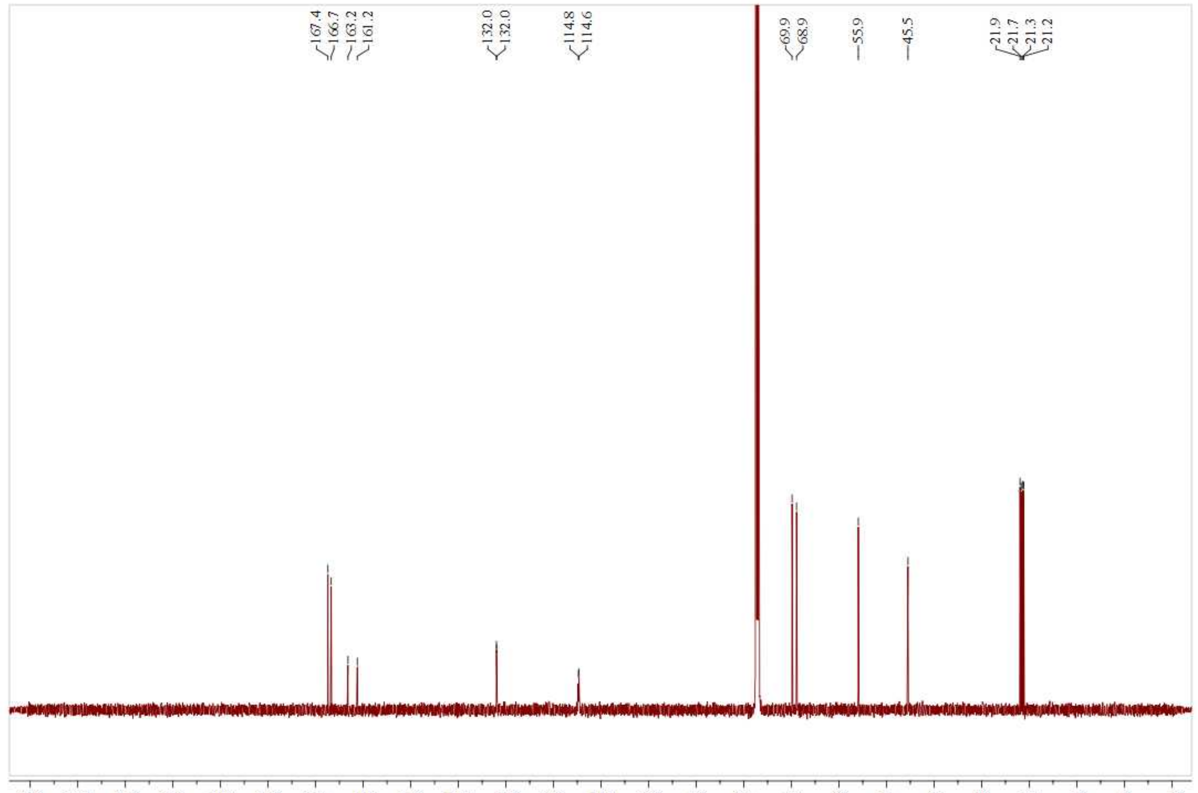

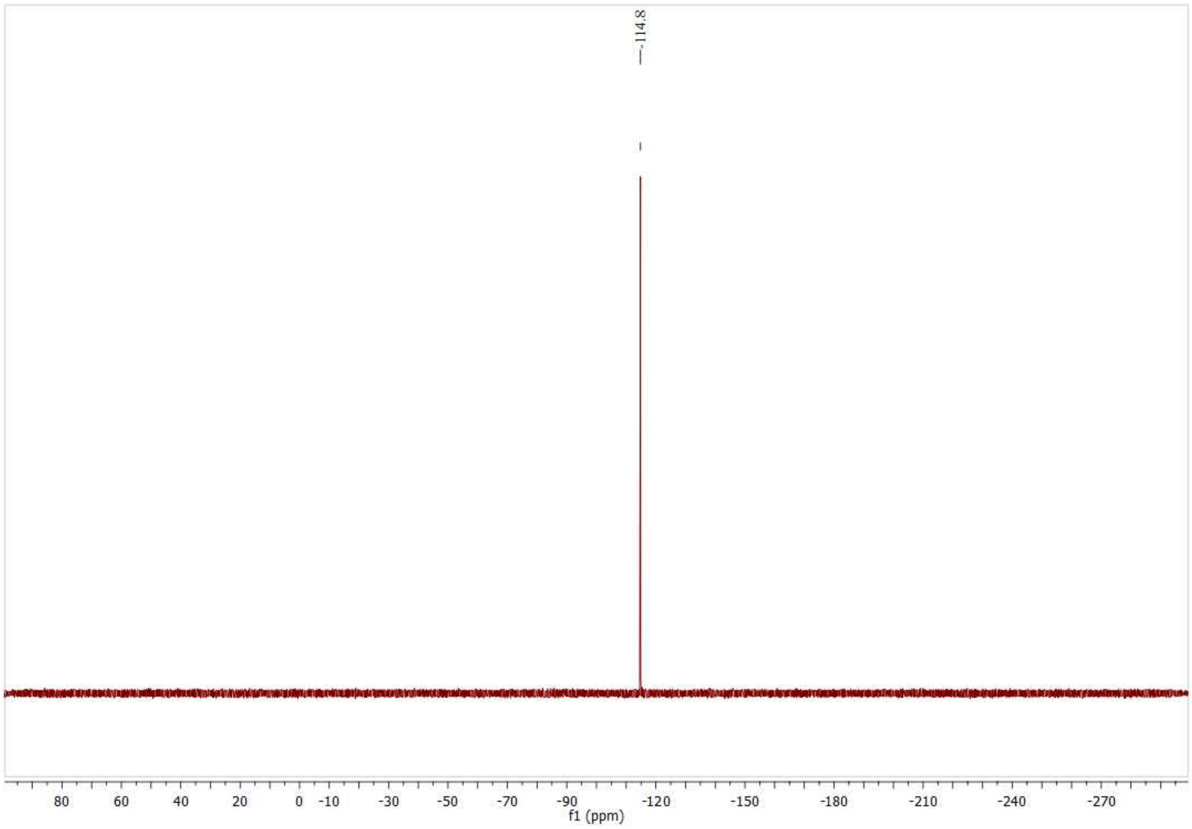
-114.8
80
60
40
20
0
-10
-30
-50
-70
-90
-120
-150
-180
-210
-240
-270
f1 (ppm)

Tetraallyl 2,3-bis(4-fluorophenyl)butane-1,1,4,4-tetracarboxylate ANTI

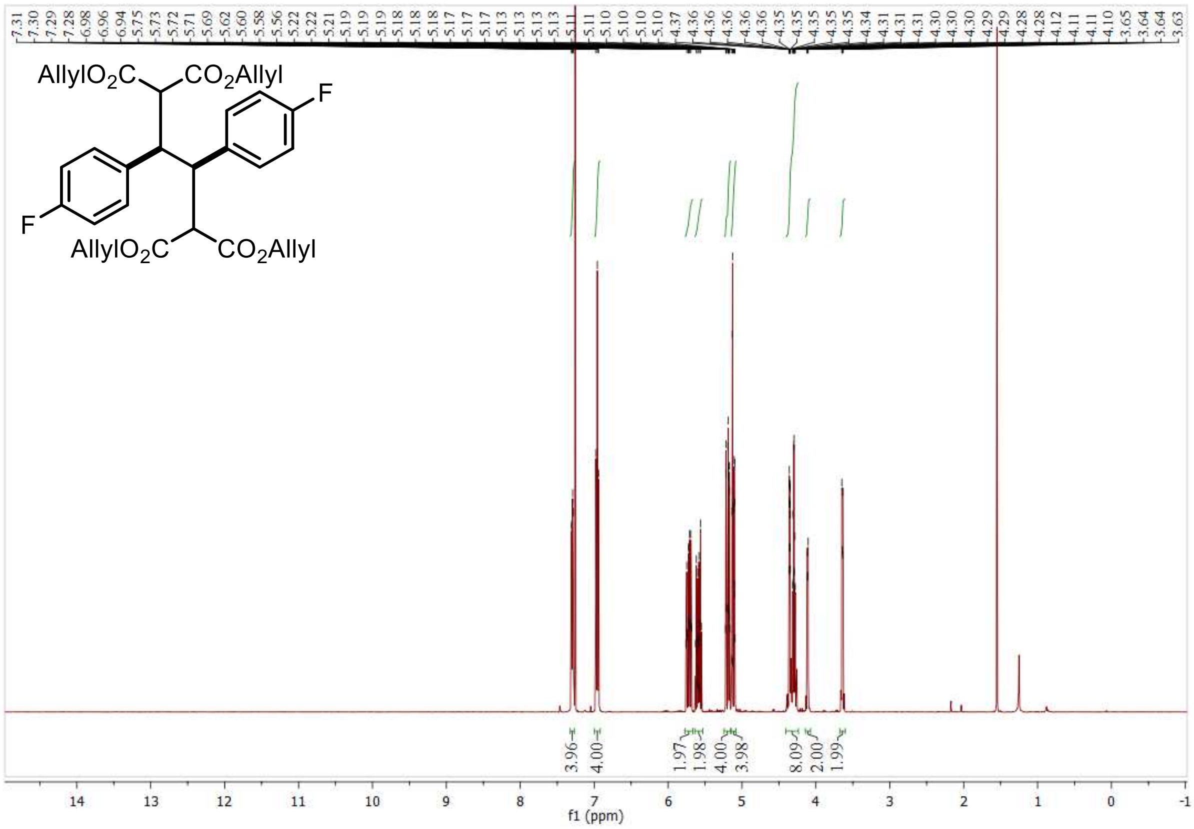

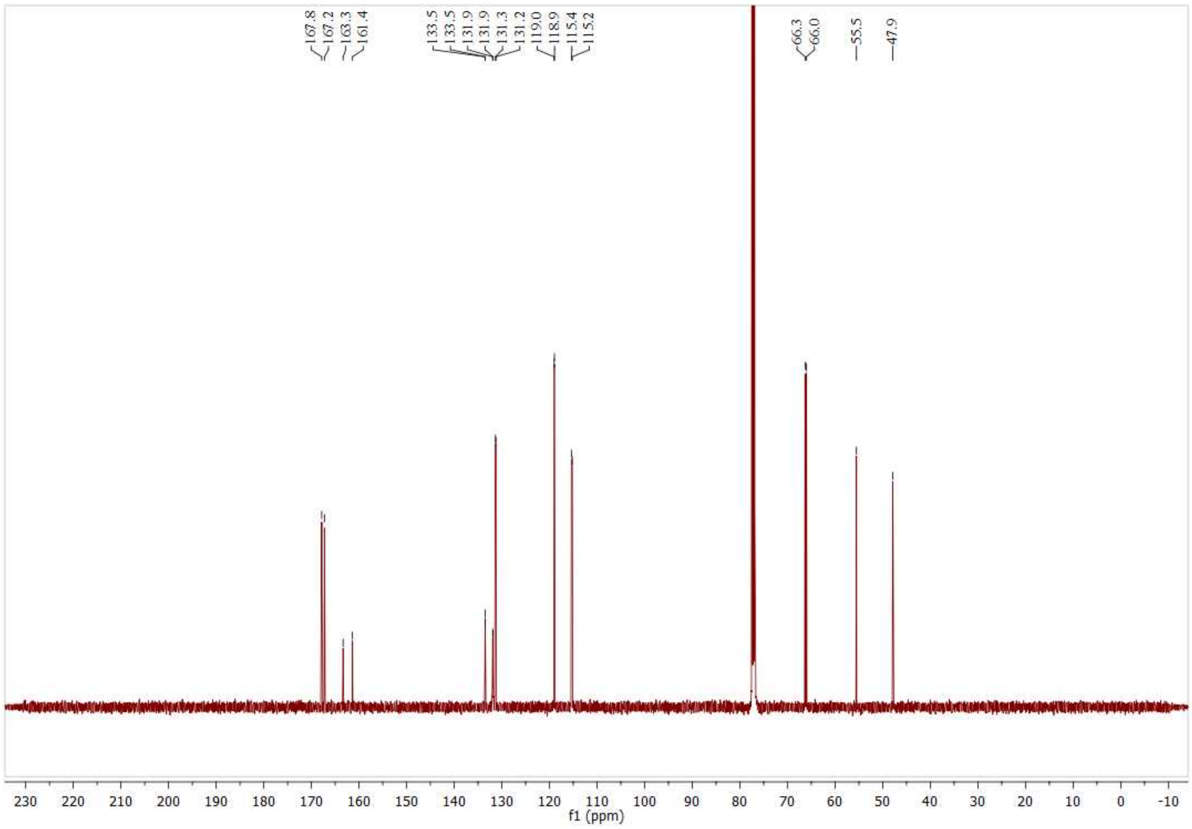

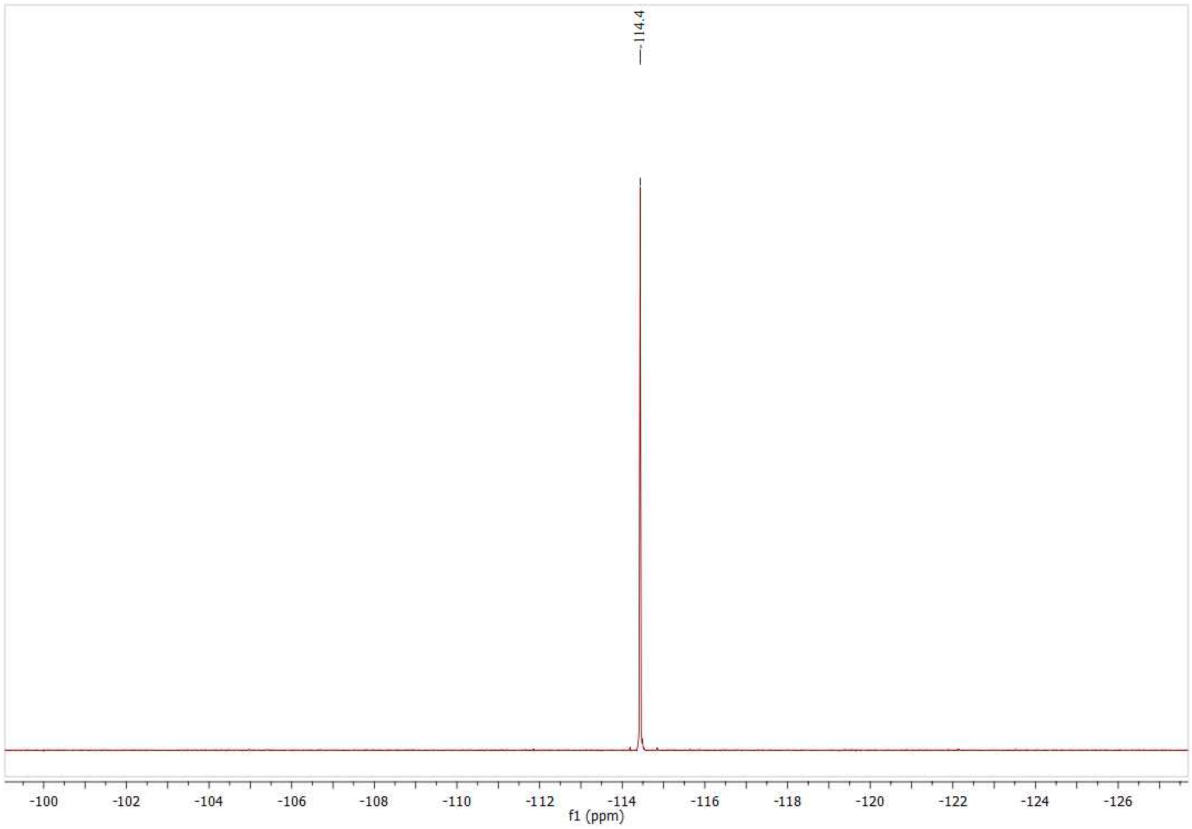

-114.4
-100
-102
-104
-106
-108
-110
-112
-114
-116
-118
-120
-122
-124
-126
f1 (ppm)

Tetraallyl 2,3-bis(4-fluorophenyl)butane-1,1,4,4-tetracarboxylate SYN

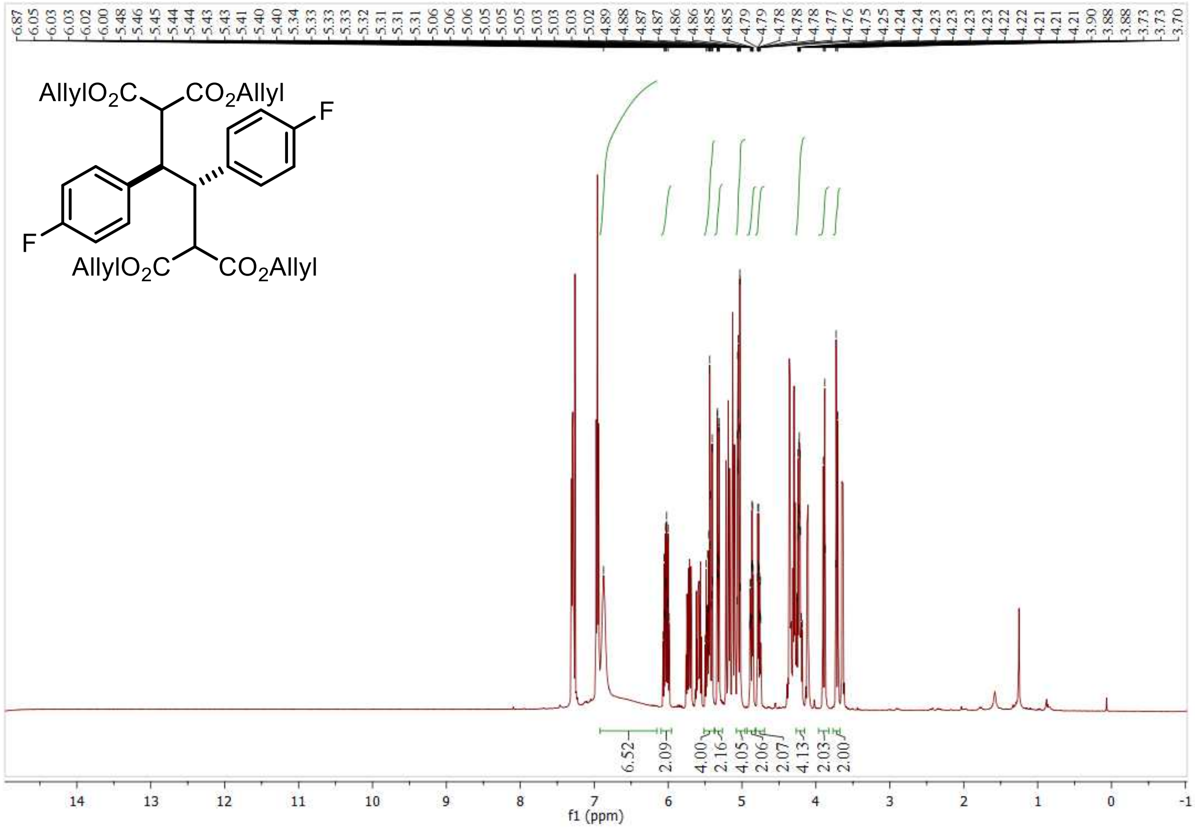

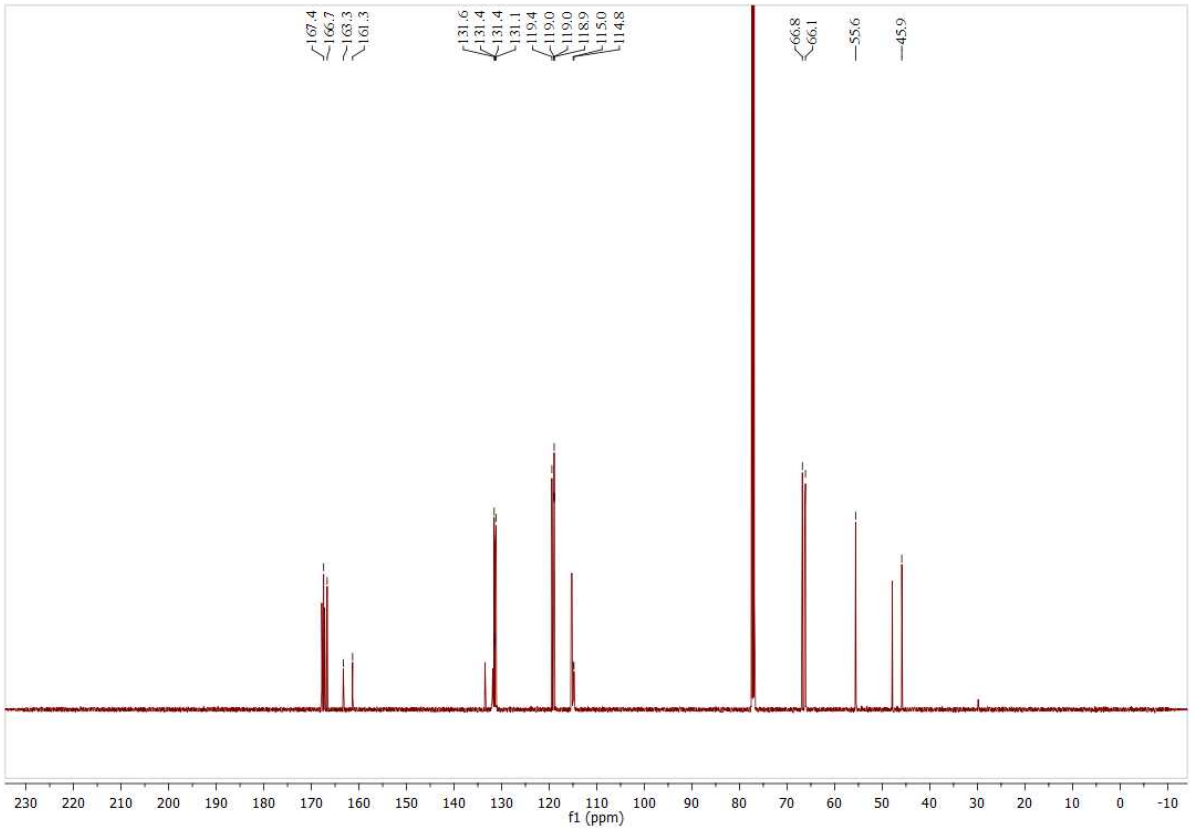

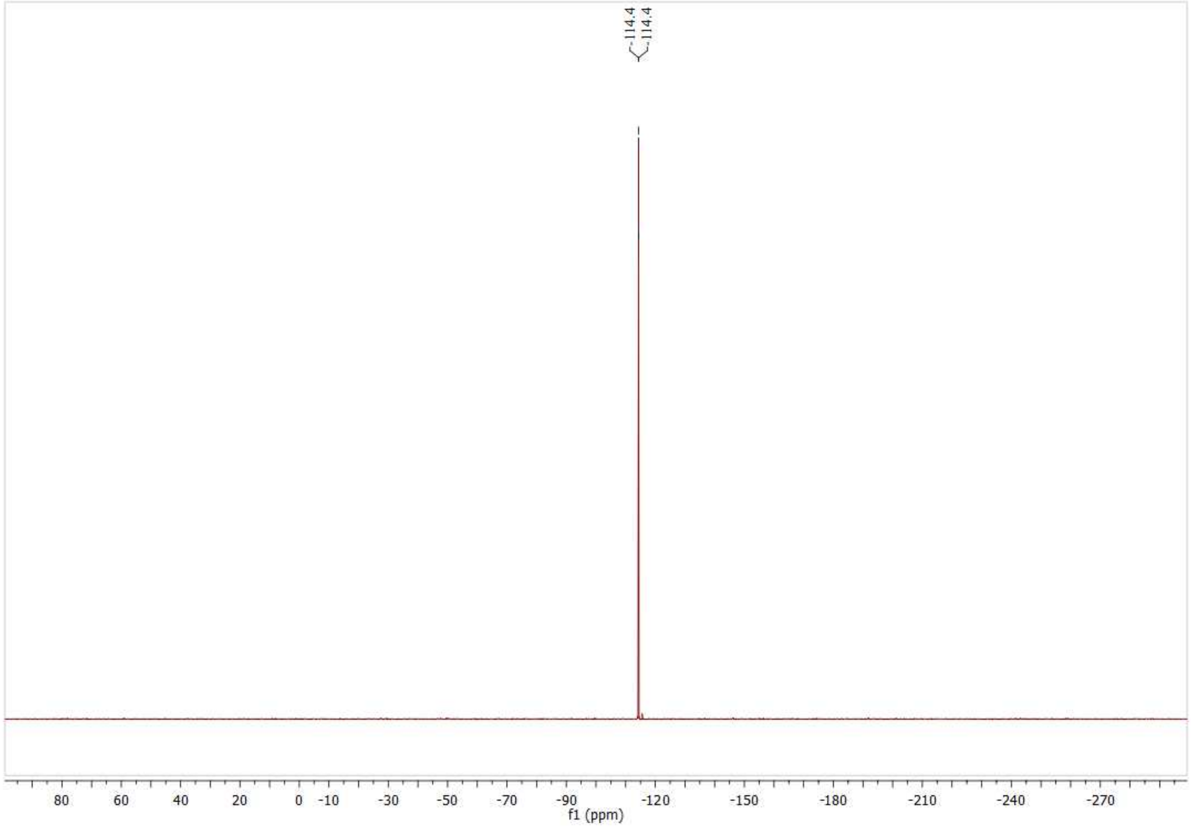
-114.4
-114.4
80
60
40
20
0
-10
-30
-50
-70
-90
-120
-150
-180
-210
-240
-270
f1 (ppm)

Diethyl 2,5-dicyano-3,4-bis(4-fluorophenyl)hexanedioate

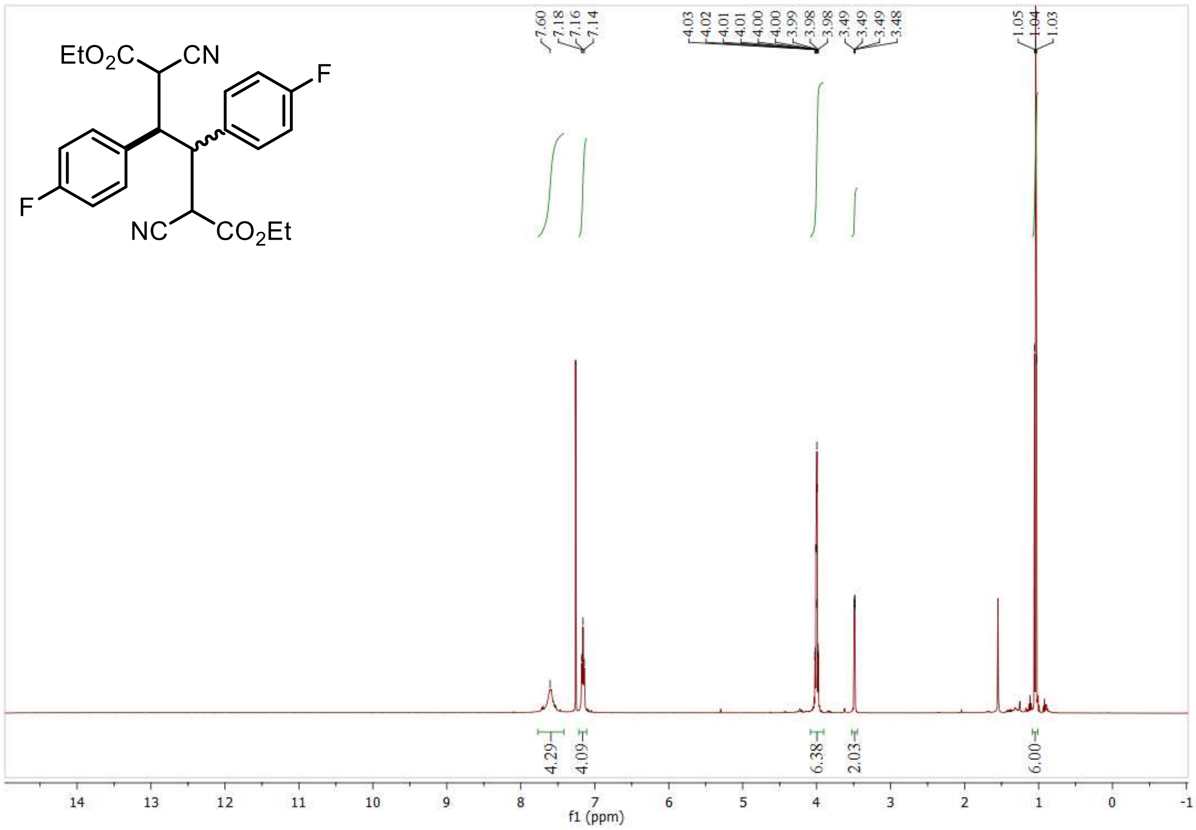

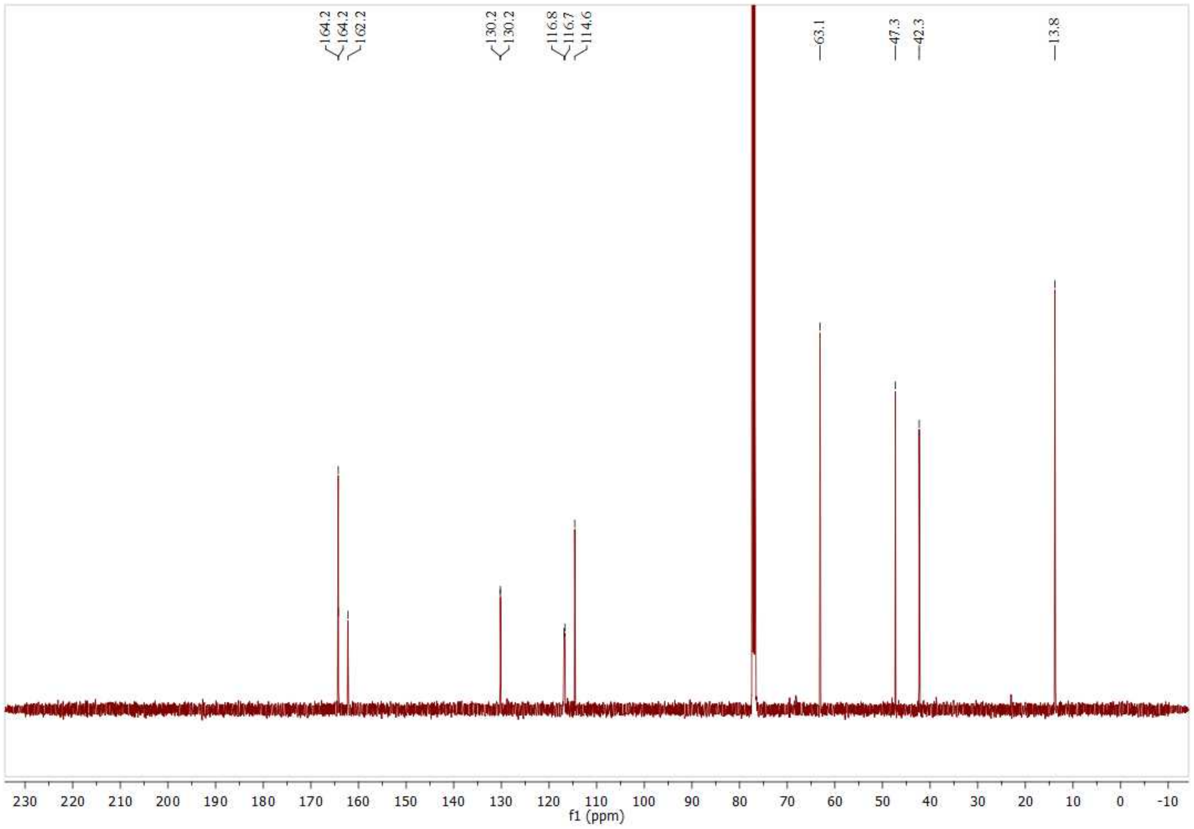

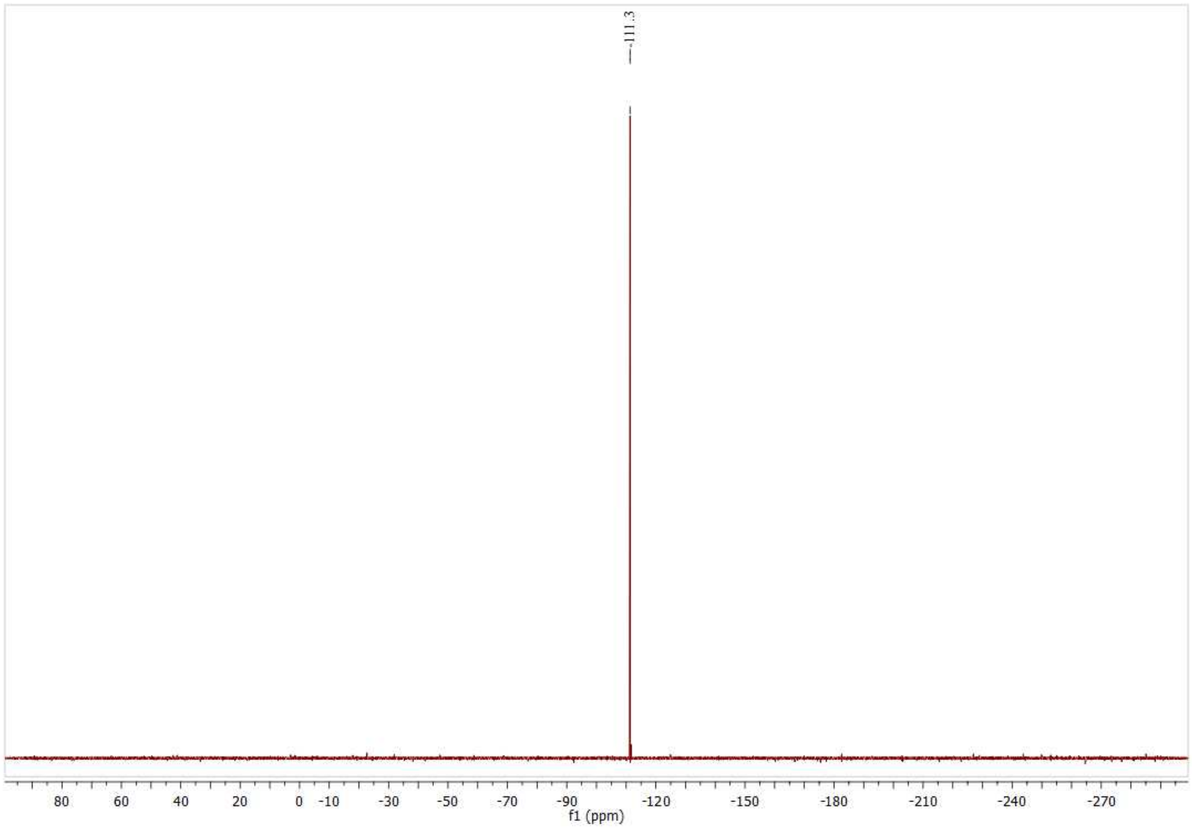

-111.3
80
60
40
20
0
-10
-30
-50
-70
-90
-120
-150
-180
-210
-240
-270
f1 (ppm)

HPLC Traces

Tetraethyl 2,3-bis(4-chlorophenyl)butane-1,1,4,4-tetracarboxylate (syn)

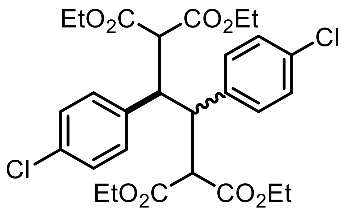

anti

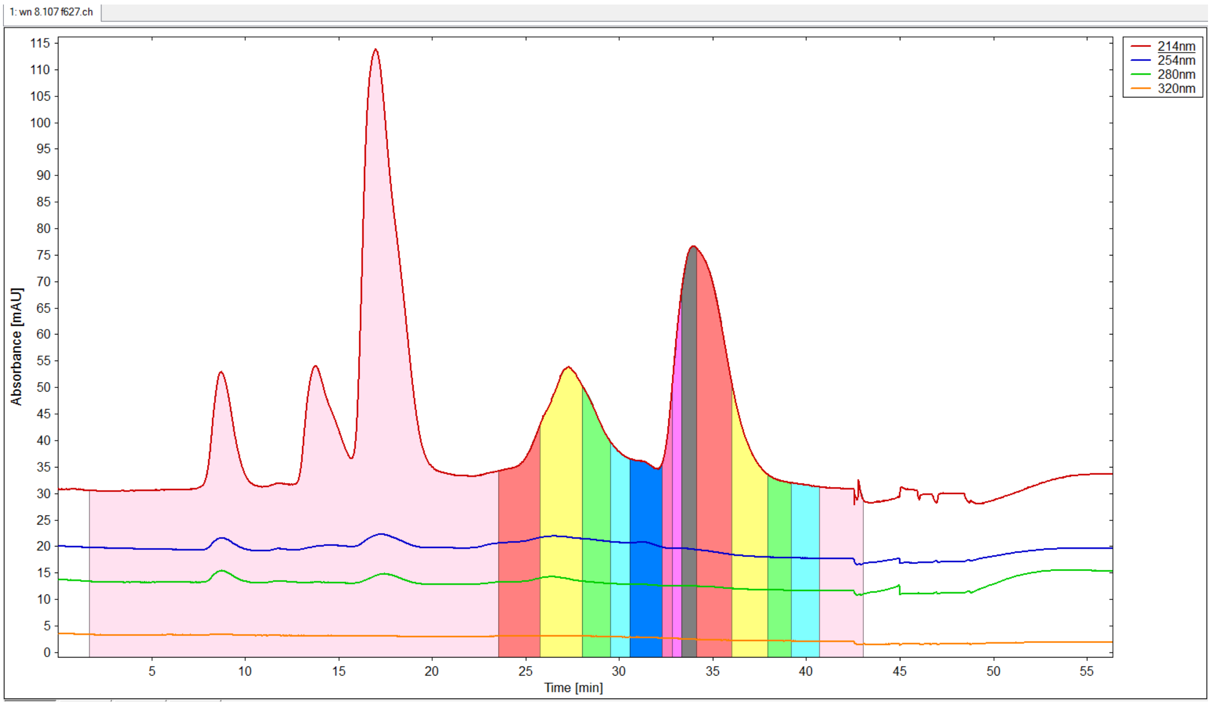

Tetraethyl 2,3-bis(4-fluorophenyl)butane-1,1,4,4-tetracarboxylate (syn)

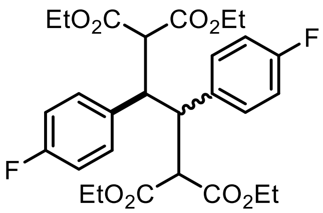

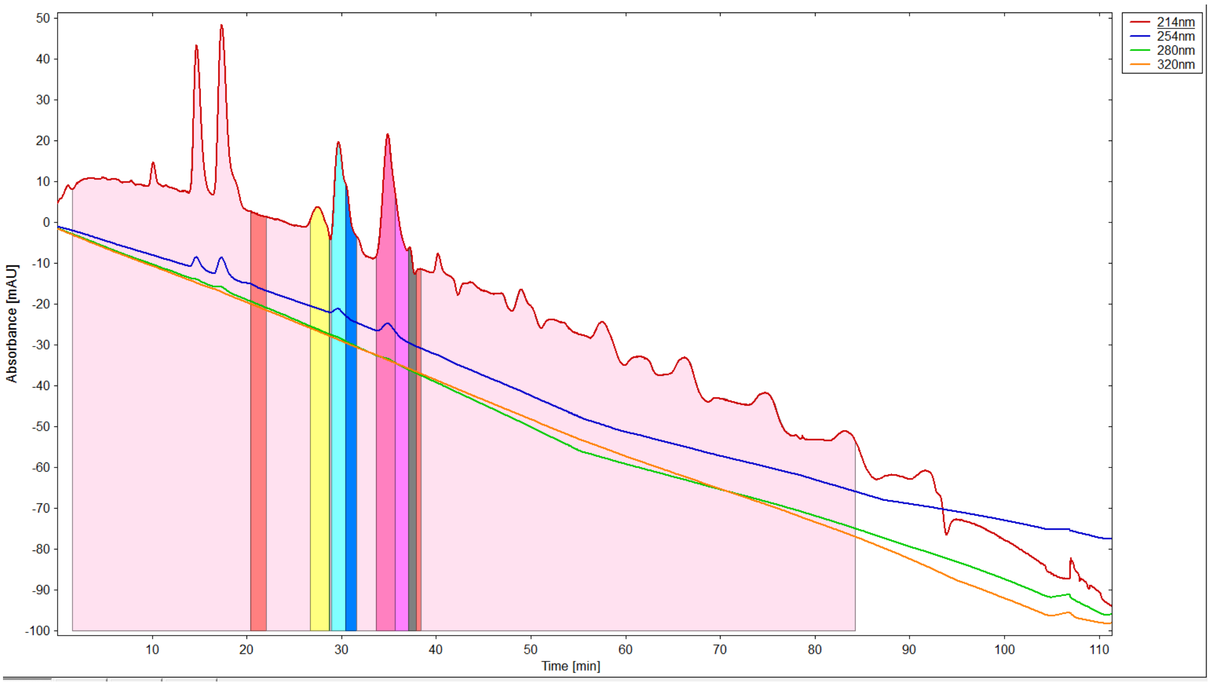

Supplement: Supplementary file 1 — Supporting Information [file ANIE-60-23128-s001.pdf]
